# Machine learning-driven alignment architecture of heterogeneous data with transient varying semantics

**DOI:** 10.1038/s41467-026-72377-w

**Published:** 2026-04-23

**Authors:** Chaofan Li, Zhichao Ma, Yangzhi Zeng, Zaizheng Yang, Jiakai Li, Zheng Yang, Junming Xiong, Shichao Niu, Zhe Wang, Hongwei Zhao, Luquan Ren

**Affiliations:** 1 School of Mechanical and Aerospace Engineering, Jilin University, Changchun, China; 2 Key Laboratory of CNC Equipment Reliability Ministry of Education, Jilin University, Changchun, China; 3 Key Laboratory of Bionic Engineering Ministry of Education, Jilin University, Changchun, China

**Keywords:** Mechanical engineering, Electrical and electronic engineering

## Abstract

Via cross-correlation algorithms or synchronized acquisition of signals, the alignment of heterogeneous data with unknown semantic time shifts and intermittent semantic variations cannot be solved. The shift is caused by different data acquisition principles of sensors, different response discrimination principles using heterogeneous data, etc. Here, we report an unsupervised alignment architecture with a supervised learning model as the kernel to overcome the limitations of brain cognition, perception, and storage in aligning complex heterogeneous data. A set of data with a time shift is input into the kernel model of the architecture to predict the semantic labels, features or continuous values corresponding to another set of data. The time shift corresponding to the maximum testing accuracy or the minimum mean squared error is the alignment parameter for the two heterogeneous datasets. This architecture is expected to serve as a preprocessing step for semantic mining of signals and for information fusion.

## Abstract (web summary)

This study presents an unsupervised alignment architecture using a supervised learning model to align heterogeneous data with unknown semantic time shifts. It demonstrates effectiveness in aligning optical and acoustic signals, enabling semantic mining and information fusion with minimal domain knowledge.

## Introduction

Artificial intelligence, widely regarded as the core driving force of the Fourth Industrial Revolution^1–3^, has been extensively applied in a variety of cutting-edge fields, including materials data factories^4,5^, materials genomics^6,7^, protein performance prediction^8,9^, customized metamaterials with tailored properties^10,11^, flexible electronic devices^12,13^, battery life prediction^14,15^, and mechanical system condition monitoring^16,17^. Artificial intelligence models with powerful expressive capacity enable researchers to establish complex correlations within and across data modalities^18–22^. Simultaneously, mapping models trained on experimental data reduces the hypothesis space for variable exploration, thereby accelerating the optimization cycle in complex mapping scenarios^6–11^. Although significant breakthroughs have yet to be achieved in the development of general artificial intelligence and superintelligence, artificial narrow intelligence is still regarded as a powerful tool for uncovering hidden patterns within data and generating novel forms of productivity^23,24^. The successful application of artificial intelligence models currently relies on an optimization function^25,26^. The optimization function guides model learning in accordance with the development objectives through the optimization process. However, whether the input and output of the model are aligned semantically is a prerequisite for the effective application of the optimization function.

The term “alignment” is commonly used in three contexts: alignment between the source and target domains in transfer learning^27–29^, alignment in multimodal transformation and fusion^30–32^, and alignment of signals along the timeline^33–36^. With respect to transfer learning, the essence of domain generalization lies in aligning the features of the source and target domains to leverage information from the source domain, thereby enhancing model performance on tasks in the target domain^29,37–42^. With respect to multimodal domains, alignment is fundamental to the effective fusion of multimodal information^31^. In multimodal learning, alignment involves projecting data from different modalities into a shared semantic space, where semantically similar concepts are placed closer together, thus facilitating the integration of diverse information sources^32^. Similarly, the goal of temporal signal alignment is to ensure semantic consistency between corresponding segments across signals. While hardware-based synchronization is the preferred approach, the differing acquisition principles of heterogeneous sensors and the varying discrimination based on heterogeneous data often limit its applicability. In such cases, cross-correlation analysis^43–45^, is considered. However, cross-correlation analysis often requires prior domain knowledge, such as understanding the positive or negative correlation between signal features. To enhance applicability, supervised learning has been employed to derive transformation functions that generate effective features for alignment^46^. A significant limitation of this approach is the reliance on manually annotated semantic time shifts between signals as the gold standard for training^46^. Aligning heterogeneous data without domain knowledge is challenging^47^, especially when semantic time shifts are unknown and semantic variations are transient^48,49^. First, the unknown semantic time shifts hinder the extraction of manually annotated semantic time shifts and effective alignment features^46^. Furthermore, transient semantic variations further hinder the establishment of associations between heterogeneous data. Consequently, a robust and unsupervised alignment method is urgently needed to overcome the associated challenges. Given the successful use of neural networks for alignment in transfer learning and multimodal domains^27–32,37–42^, a critical research question arises: can a novel neural network-driven architecture be designed to address the challenges of heterogeneous data alignment with unknown semantic time shifts and intermittent semantic variation?

Here, we present an unsupervised alignment architecture with a supervised learning model as the kernel to align heterogeneous data with unknown semantic time shifts and intermittent semantic variations. The feasibility and universality of the proposed architecture were validated through alignment experiments between optical and acoustic signals in the context of arc detection in current‒carried friction, as well as experiments based on a predefined semantic shifts. On the basis of studies of the impacts of kernel models, the number of samples, and the sample category ratio on alignment accuracy, a two-level alignment strategy was subsequently proposed to reduce the algorithmic costs of the proposed architecture. The integration of machine learning provides alignment architectures with several advantages, including adaptability to diverse data types, minimal domain knowledge requirements, and the ability to circumvent the cognitive, perceptual, and storage limitations of the human brain, thereby enabling the alignment of complex heterogeneous data. The architecture accommodates a broad range of kernel models, including classification and regression types, and supports traditional machine learning algorithms, time series models, and convolutional neural networks as kernel models. This architecture enables the alignment of multiple data types, including tabular data, time series, and image sequences, with various semantic representations from other data, such as discrete labels, continuous values, and semantic vectors. Moreover, an example of applying the architecture is provided. First, using the architecture, alignment with an accuracy of 5 ms between optical and acoustic signals under current-carrying friction arc detection conditions was achieved. On the basis of this alignment, an arc detection model with 90% accuracy was developed to enable semantic mining of acoustic signals. Finally, interpretability analysis was subsequently performed to demonstrate that the semantic sequence of the acoustic response lags behind that of the synchronous optical response, underscoring the necessity of the alignment architecture.

## Results and discussion

### Alignment requirements and architecture

**Fig. 1:**
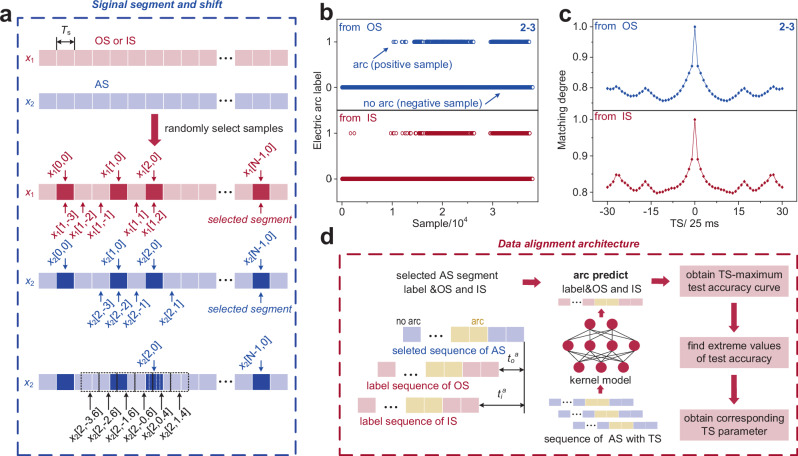
The alignment architecture for heterogeneous signals. **a** Process of random sampling and symbol definition of signal segments. **b** Label obtained from infrared signals (IS) and optical signals (OS) to determine arc damage, **c** Time shift (TS)-accuracy curve of the labels. **d** The alignment architecture and its application flow. Source data are provided as a Source Data file.

To illustrate the significance of aligning heterogeneous signals, the arc damage frequency is detected under current-carried friction conditions via optical signals (OSs), infrared signals (ISs), and acoustic signals (ASs), as presented in Supplementary Discussion 1. The abbreviations and full names of the technical terms used in this paper are provided in Supplementary Table 1 and will not be repeated in the following text. The alignment between AS and OS or IS serves as a prerequisite for extracting meaningful information from the AS. Data segmentation is essential for accurate signal alignment. OS and IS images are temporally integrated spectral information over fixed intervals, inherently limiting flexibility for temporal segmentation. In contrast, AS, which is a high-frequency one-dimensional waveform, supports fine-grained and precise temporal segmentation. Accordingly, the AS can be adaptively segmented to align with the fixed temporal windows of OS or IS images. The AS was segmented according to the acquisition time ${T}_{{{\rm{s}}}}$ of each image. The OS or IS images were subsequently randomly sampled into the $N$ group at a 1:1 ratio of the arc response to non-arc response classes, and the corresponding AS segments were extracted. The resulting signal segments are represented by Eq. (1):

1 $$\left\{\begin{array}{ccc}{x}_{1}\left[n,t\right] &=& {{{\bf{A}}}}^{n,t}\\ {x}_{2}\left[n,t\right] &=& \left\{{a}_{1}^{n,t},{a}_{2}^{n,t},\cdots,{a}_{s}^{n,t}\right\}\end{array}\right\}n\in \left\{0,2,\cdots,N-1\right\}$$

where ${x}_{1}\left[n,t\right]$ and ${x}_{2}\left[n,t\right]$ denote the *n*th two-dimensional time series signal with a time shift (TS) $t$ in the randomly sampled signal segment and the corresponding one-dimensional time series signal segment, respectively. The corresponding time length of signal segments ${x}_{1}\left[n,t\right]$ and ${x}_{2}\left[n,t\right]$ is ${T}_{{\mbox{s}}}$. In this case, ${{{\bf{A}}}}^{n,t}$ represents an OS or IS image, and ${x}_{2}\left[n,t\right]$ is a one-dimensional AS segment with amplitude ${a}_{s}^{n,t}$ and length $s$. The segmentation details of the signal segment and the symbol definition rules of Eq. (1) are shown in Fig. 1a. For signal alignment, cross-correlation algorithms are commonly employed, as shown in Eq. (2):

2 $$\left\{\begin{array}{cc}{t}_{{{\rm{c}}}} &=\\ {T}_{{{\rm{c}}}} &=\end{array}\begin{array}{c}{\max }_{t}{\sum }_{n=1}^{N}{F}_{1}^{{{\rm{tf}}}}\left({x}_{1}\left[n,0\right]\right)\cdot {F}_{2}^{{{\rm{tf}}}}\left({x}_{2}\left[n,t\right]\right)\\ {t}_{{{\rm{c}}}}\cdot {T}_{{{\rm{s}}}} \hfill \end{array}\right\}$$

where ${T}_{{{\rm{c}}}}$ and ${t}_{{{\rm{c}}}}$ denote the semantic TS and semantic alignment parameters, respectively, between the sequences of signal segments ${x}_{1}\left[n,0\right]$ and ${x}_{2}\left[n,0\right]$ obtained via the cross-correlation algorithm, ${F}_{1}^{{{\rm{tf}}}}$ and ${F}_{2}^{{{\rm{tf}}}}$ denote the corresponding transformation functions of ${x}_{1}\left[n,t\right]$ and ${x}_{2}\left[n,t\right]$, respectively; and the search range for semantics is $t\cdot {T}_{{{\rm{p}}}}$. However, in this case, defining ${F}_{1}^{{{\rm{tf}}}}$ and ${F}_{2}^{{{\rm{tf}}}}$ is challenging because of the unclear positive correlation between certain features of ${x}_{1}\left[n,t\right]$ and ${x}_{2}\left[n,t\right]$. Moreover, supervised learning is not applicable for determining ${F}_{1}^{{{\rm{tf}}}}$ and ${F}_{2}^{{{\rm{tf}}}}$^46^ because of the unknown and inherently ambiguous nature of the true semantic TS between ${x}_{1}\left[n,0\right]$ and ${x}_{2}\left[n,0\right]$. Addressing these challenges necessitates a robust unsupervised algorithm or architecture, the development of which demands further analytical and experimental investigation.

The labels (“1” denotes a positive sample corresponding to the arc, and “0” denotes negative samples corresponding to the absence of arc) obtained from the IS and OS (Fig. 1b) were subsequently subjected to a time-shift process to generate time-shifted labels. These labels were compared with the original label to construct a TS-matching degree curve (Fig. 1c). The accuracy was optimal (100%) in the absence of TS, but declined sharply with increasing TS near 0 TS. As the TS increased further, the accuracy entered a nonmonotonic, fluctuating regime. Assuming that the AS was related to arc damage and that the correlation could be captured by an artificial intelligence model, the AS segment with the TS can be used as the input of the model to monitor arc damage. For such a model, high dataset quality is achieved when the TS between AS inputs and labels derived from OS or IS closely approximates the true semantic TS, which improves the ability of the model to capture consistent patterns across training and testing sets, resulting in higher testing accuracy, similar to the trend in Fig. 1c. Therefore, during model training, the maximum testing accuracy corresponded to a specific alignment parameter between the source signal of the label and the AS. The obtained alignment parameter may correspond to, or closely approximate, the actual TS on the time axis, ensuring semantic alignment between the two signals. The implementation and application workflow of the above algorithm architecture are shown in Fig. 1d. Here, ${T}_{0}^{{{\rm{a}}}}$ denotes the semantic TS between the OS sequence and the AS sequence, whereas ${T}_{i}^{{{\rm{a}}}}$ denotes the semantic TS between the IS sequence and the AS sequence. The formal representation of the algorithm architecture is provided in Eq. (3), and the corresponding pseudocode for implementing the architecture is shown in Supplementary Algorithm 1.

3 $$\left\{\begin{array}{lcc}{\theta }_{t} &=& {min }_{{\theta }_{t}}{\sum }_{{n}_{{{\rm{tr}}}}=1}^{{N}_{{{\rm{tr}}}}}L({F}_{{\theta }_{t}}({F}_{2}^{{{\rm{stft}}}}[{n}_{{{\rm{tr}}}},t]),{y}_{1}[{n}_{{{\rm{tr}}}},0])\\ {t}_{n} &=& {max }_{t}{\sum }_{{n}_{{te}}=1}^{{N}_{{te}}}M({F}_{{\theta }_{t}}({F}_{2}^{{{\rm{stft}}}}[{n}_{{{\rm{te}}}},t]),{y}_{1}[{n}_{{{\rm{te}}}},0])/{N}_{{{\rm{te}}}}\\ N&=&{N}_{{{\rm{te}}}}+{N}_{{{\rm{tr}}}}\\ {T}_{{{\rm{n}}}}&=&{t}_{{{\rm{n}}}}\cdot {T}_{{{\rm{s}}}} \end{array}\right\} \, {y}_{1} \, \left[n,t\right]\in \{0,1\}$$

where ${\theta }_{t}$, ${F}_{2}^{{{\rm{stft}}}}$, ${y}_{1}[n,t]$, and $L$ denote the parameters of the neural network ${F}_{{\theta }_{t}}$ with a TS parameter $t$, the time-frequency spectrogram of signal ${x}_{2}\left[{n}_{{{\rm{tr}}}},t\right]$ after short-time Fourier transform, the label corresponding to signal ${x}_{1}\left[n,t\right]$, and the cross entropy loss function, respectively. ${F}_{2}^{{{\rm{stft}}}}[{n}_{{{\rm{tr}}}},t]$ and ${y}_{1}[{n}_{{{\rm{tr}}}},0]$ are used as input‒output pairs for training neural networks, whereas ${F}_{2}^{{{\rm{stft}}}}[{n}_{{{\rm{te}}}},t]$ and ${y}_{1}[{n}_{{{\rm{te}}}},0]$ are used for testing. ${N}_{{{\rm{te}}}}$ and ${N}_{{{\rm{tr}}}}$ denote the number of samples in the training and testing sets, respectively. The matching function $M$ returns a value of 1 if two elements are identical, and 0 otherwise. ${T}_{{{\rm{n}}}}$ and ${t}_{{{\rm{n}}}}$ denote the semantic TS and semantic alignment parameters, respectively, between the sequence of signal ${x}_{1}\left[n,0\right]$ and that of signal ${x}_{2}\left[n,0\right]$, as determined by the neural network-driven data alignment architecture. The search range for the semantic TS is defined by *t*$\cdot {T}_{{{\rm{s}}}}$. While initially developed for aligning OS or IS with AS, the proposed architecture is broadly applicable to a range of problems involving the alignment of two heterogeneous data streams with unknown initial semantic time shifts and intermittent semantic variations over time. The successful application of this architecture is conditional on the possession of semantic labels for one of the two heterogeneous data streams. The observed semantic TS arises from a combination of factors. The semantic TS typically arises from two main factors: the inherently distinct physical principles of the sensors (e.g., the vastly different propagation speeds of light and sound), and the data processing strategies, where optimal response identification may depend on data from before, during, or after the event. The proposed algorithm architecture aims to solve the ultimate data alignment problem, which is particularly applicable to alignments that cannot be solved through synchronous signal acquisition. Additionally, for alignment exploration under different TSs, the AS must be repeatedly segmented with different initial TSs. Choosing the length of each segment ${T}_{{{\rm{s}}}}$ as an integer multiple of the semantic TS search step size is advantageous for reducing the number of segments.

### Alignment verification and applications

**Fig. 2:**
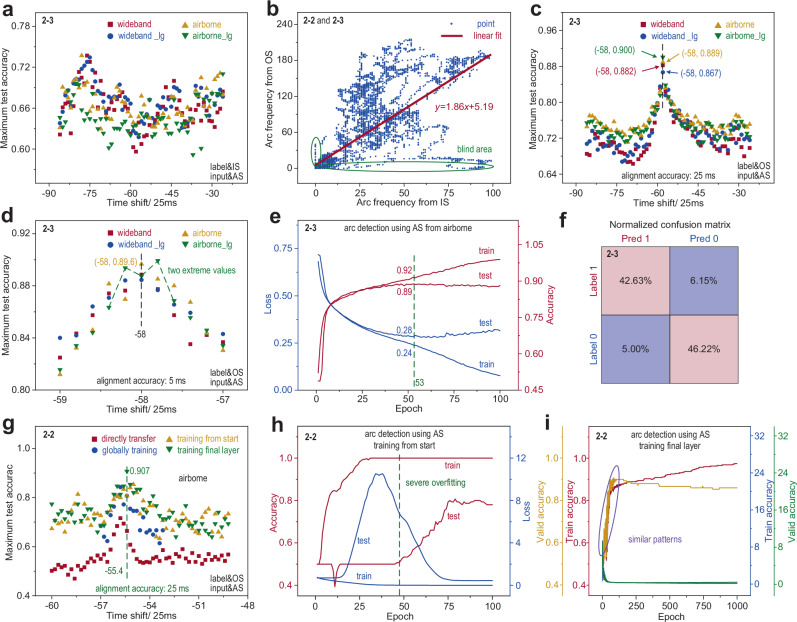
Verification and application of the data alignment architecture. **a** Alignment results (step size: 25 ms) of infrared signals (IS) and acoustic signals (AS). **b** Comparison of the arc frequency every 10 s calculated from optical signals (OS) and IS, **c**, **d** Alignment results (step size: 25 and 5 ms) of OS and AS in dataset **2-3**. **e** Epoch-accuracy and loss curves of the kernel model with aligned AS as the input and labels obtained from the IS as the output. **f** The normalized confusion matrix of the highest testing accuracy in Fig. 2e. **g** Alignment results (step size: 5 ms) of OS and AS data in dataset **2-2**. **h**, **i** Epoch-accuracy and loss curves of the model in Fig. 2g. Source data are provided as a Source Data file.

To validate the effectiveness of the proposed alignment architecture in aligning optical signals (OSs) or acoustic signals (ISs) with acoustic signals (ASs), corresponding experiments are conducted to construct datasets, as detailed in Supplementary Discussion 2. The types of collected signals and experimental conditions are summarized in Supplementary Table 3. These datasets consisted of three groups, **1-1-1** to **1-3-4,** **2-2** and **2-3**, and **3-1** to **3-3**. The details of the labels for IS and AS are shown in Supplementary Table 4. First, the ISs and ASs from datasets **2-3** were utilized for alignment analysis. A modified version of DenseNet 121 (RDNet_41) served as the kernel model within the alignment architecture to estimate the semantic time shift (TS) between signals. An overview of all heterogeneous data alignment tasks, including the number of samples used, the kernel model, and the training parameters, is provided in Supplementary Table 5. The input or output shapes and architectural details of the kernel models are summarized in Supplementary Table 6. The length of each segment ${T}_{{{\rm{s}}}}$ was set to 25 ms. These details are not reiterated in the subsequent text. The Adam optimizer was used for all training procedures. The labels obtained by the IS used for modeling corresponded to 40 Hz, which were converted from labels corresponding to 80H. To maintain class balance^50^ in the dataset, the ratio of positive to negative samples was set to 1:1. The training set and testing set are divided from the dataset at a 7:3 ratio. The learning rate and number of epochs needed to be set appropriately to achieve a training accuracy of over 90% for the kernel model to obtain the maximum testing accuracy (MTA) corresponding to the TS and draw the TS-MTA curve. Figure 2a shows the alignment results of **2-3** sets of IS and AS. The extreme values of the MTA for the four sets of input signals were low and the corresponding TS *t*_*i*_^*e*^ was -75 × 25 ms, which deviates significantly from the TS reference range. These results indicate that the proposed alignment architecture failed on this dataset, potentially because of the insufficient exposure time of the collected IS images and slight inconsistencies in the sampling time for individual IS images. The feasibility of arc detection via IS is shown in Fig. 2b and Supplementary Discussion 3.

**Fig. 3:**
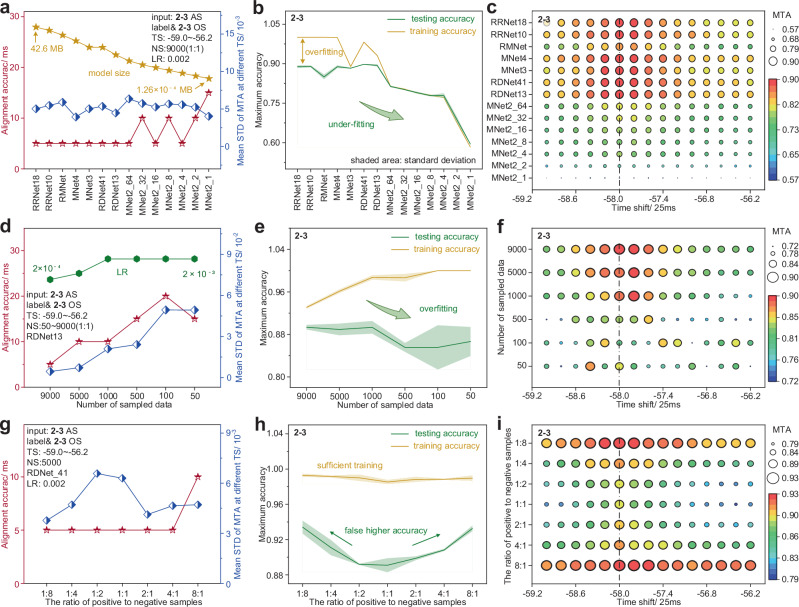
Analysis of factors affecting alignment accuracy. **a** Variance in alignment accuracy and standard deviation (STD) of the maximum testing accuracy (MTA) across different kernel models under the same learning rate (LR). **b** Maximum training and testing accuracy across different kernel models. **c** Bubble chart of the MTA across different kernel models and time shifts (TSs) (step size: 5 ms). **d** Alignment accuracy of the RDNet13 kernel models as the number of samples (NS) varies. **e** Corresponding NS–maximum accuracy curve. **f** Corresponding bubble chart of the MTA across different TSs (step size: 5 ms) and NS. **g** Variation in alignment accuracy with the proportion of positive to negative samples. **h** Maximum accuracy across different training sample proportions. **i** Corresponding bubble chart of MTA across different TSs (step size: 5 ms) and training sample proportions. Source data are provided as a Source Data file.

The alignment results (step size: 25 ms) for **2-3** sets of OS and AS data are shown in Fig. 2c. The MTAs of the 4 sets of input signals all reached extreme values at a TS of -58 × 25 ms, and the maximum MTA was 90%. At this TS, the MTA across models trained using four different forms and sources of the spectrogram as inputs was above 86%, demonstrating the effectiveness of AS in arc detection. The *t*_*o*_^*a*^ was determined to be -58 × 25 ms with an alignment accuracy of 25 ms for OS and AS in dataset **2-3**. The corresponding typical TS-accuracy curves away from the true semantic TS are shown in Supplementary Fig. 13. Additionally, the variation trend of the curves in Fig. 2c and Supplementary Fig. 13 was similar to the variation trend of the curve in Fig. 1c with increasing TS, indicating the feasibility of the proposed alignment architecture. A short TS step size (5 ms) was subsequently applied to calculate the MTA, and the corresponding fine alignment results are shown in Fig. 3d. Among the 4 sets of signals, the MTA corresponding to 3 sets of signals reached extreme values at -58 × 25 ms, indicating that the alignment parameter *t*_*o*_^*e*^ was -58 × 25 ms with an alignment accuracy of 5 ms. The epoch-accuracy and loss curve of the kernel model corresponding to this TS parameter are shown in Fig. 3e. For the aligned AS and OS, the kernel model corresponding to the AS collected by the airborne acoustic sensor, without lg transformation, achieved the maximum MTA among the four input configurations. Therefore, subsequent alignment of the AS and OS, as well as arc detection model using AS would be based on the spectrogram of the AS collected by the airborne sensor without lg transformation. The 53rd epoch kernel model with the highest validation accuracy and an acceptable degree of overfitting was selected for subsequent class activation mapping calculations and transfer learning. The confusion matrix of the model for inference on the testing set is shown in Fig. 2f. The model was subsequently applied to predict whether dataset **2-2** ASs corresponded to arc damage, as shown in Fig. 2g (direct transfer). The maximum MTA was less than 80%, indicating that varying normal loads resulted in different distributions of the AS. Therefore, the alignment of OS and AS in dataset **2-2** was reconducted, and the TS-accuracy curve is presented in Fig. 2g (training from start). However, the epoch-accuracy and loss curves (Fig. 2h) for the kernel model with respect to the alignment parameters show significant overfitting in the early training stages. Although the degree of overfitting decreased after the 75th epoch, it remains problematic and difficult to accept. Given that only 256 positive samples were available in dataset **2-2** and that the distribution of AS in dataset **2-2** may resemble that in dataset **2-3**, transfer learning was adopted for modeling. The global transfer learning strategies via minor learning rates and training only the last fully connected layer of the model were adopted for modeling, respectively, and the alignment results are shown in Fig. 2g. Compared with the global training strategy with a small learning rate, training only the last layer yielded a higher MTA. This strategy was equivalent to transforming the training convolutional neural network into a fully connected layer with reduced complexity, thereby reducing the sample demand. Additionally, when training only the last layer, the feature extraction function of the convolutional layer of the base model was fully utilized, resulting in higher reliability than that of the global training strategies. The maximum MTA of the transfer learning model peaked at a TS of -55.4 × 25 ms, which corresponded to a high accuracy of 90.7%. Moreover, the TS corresponding to the peak is close to the reference range of 52.50–60.00 × 25 ms for the *t*_*o*_^*e*^. These results indicate that the proposed alignment architecture is also applicable to transfer learning. The semantic TS parameter *t*_*o*_^*e*^ for **2-2** sets of data was -55.4 × 25 ms, with an alignment accuracy of 5 ms. Figure 2i shows the epoch-accuracy loss curve of the training model corresponding to this TS. Compared with the curve in Fig. 2h, the unified patterns in the training and testing sets emerge during the early stages of model training. In conclusion, the effectiveness of the proposed alignment architecture was validated through modeling and analysis on the basis of datasets **2-2** and **2-3**.

Additionally, we repeated the above alignment, and the alignment between the OS and AS corresponding to different friction pair materials was analyzed as detailed in Supplementary Discussion 4. The experimental results indicate that the comprehensive results of repeated alignment are more reliable than those of single alignment are, and that determining the semantic TS via the distribution variation of testing accuracy across different TSs is more reliable than relying solely on the MTA. The subsequent alignment-related figures correspond to the comprehensive results of three repeated alignments and the corresponding description will not be repeated in the future. The higher training or testing accuracy under semantic TS than under other TS conditions can be attributed to the weaker correspondence between input signals and semantic labels in non-semantic alignments, which reduces the proportion of functionally relevant components in the loss function that effectively drive model training, thereby slowing the training rate. Moreover, interpretability analysis^51^ was performed via the arc detection model with AS as the input (Fig. 2e), as detailed in Supplementary Discussion 5. The interpretability analysis results and the comparative analysis between the reference time shift range and the semantic TS in alignment between OS and AS suggest that the semantics of AS lag behind synchronized OS semantics for arc detection tasks, which underscores the value of the proposed alignment architecture. The effectiveness of arc detection on the basis of the IS or AS was demonstrated on the basis of the effectiveness of the OS. Therefore, the effectiveness of arc detection via OS is verified in Supplementary Discussion 7. The training and inference costs of the arc detection models based on OS, IS, or AS are discussed in Supplementary Discussion 8.

### Factors affecting alignment accuracy

In systems with a constant semantic time shift (TS), synchronous hardware triggering is applied to ensure the simultaneous acquisition of heterogeneous signals. Simultaneously, the TS can be estimated once during an initial calibration using the alignment architecture and subsequently reused, resulting in minimal computational cost. In contrast, in systems with a varying semantic time shift—such as those affected by time-varying delays—the alignment architecture must be further optimized to reduce the computational cost. Figure 3 illustrates the alignment accuracy between OS and AS in dataset **2-3**, evaluated across different kernel models (KMs), the number of samples (NS), and positive-to-negative sample ratios. Figure 3a presents the alignment accuracy for various KM sizes, including RRNet18 and RRNet10, with KM sizes ranging from 42.6 MB to 1.26 × 10⁻⁴ MB. The smallest model (MNet2_1) consists of only two layers and a single channel. As the KM size decreases, the alignment accuracy decreases from 5 ms to 15 ms (Fig. 3a). Contrary to the intuitive expectation that the mean standard deviation (STD) of maximum testing accuracy (MTA) across different TSs systematically increases with reduced KM size, no consistent negative correlation between these two variables is observed. Figure 3b shows the mean of the maximum training accuracy or the MTA across the KMs. Reducing the KM size corresponds to a clear transition from overfitting to underfitting. Despite the emergence of underfitting, the impact on alignment accuracy is not exponential. The corresponding bubble plot in Fig. 3c illustrates the relationships among the KMs, TS, and MTA. For each model, the MTA initially increases with TS, reaches a peak near the semantic TS, and subsequently decreases. Notably, this peak-like extremum remains pronounced even as the KM size decreases, demonstrating that alignment stability remains significantly unaffected by the KM size. The overall training process maintains relative stability despite potential underfitting tendencies in small kernel models, as corroborated by the epoch-accuracy and loss curves in Supplementary Fig. 22.

Figure 3d–f illustrate the impact of the NS on alignment accuracy. When the same kernel model is employed for alignment across varying NSs, the models trained with fewer samples exhibit more pronounced underfitting. This underfitting is reflected in the reduced testing accuracy, which in turn undermines the credibility of the alignment. Therefore, the KM was trained using learning rates that increased from 2 × 10⁻⁴ at an NS of 9000 to 2 × 10⁻³ at an NS of 50. The learning rate is negatively correlated with the NS (Fig. 3d). The alignment accuracy deteriorates from 5 ms at an NS of 9000 to 20 ms at an NS of 50. Concurrently, the average STD of the MTA across TSs increases as the NS decreases. With decreasing NS, the kernel model in the semantic TS exhibits increased overfitting and diminished generalization capability, resulting in decreased credibility of the alignment result, as shown in Fig. 3e. As shown in Fig. 3f, as the NS decreases, the trend of peak-shaped variation in the MTA near the semantic TS becomes less pronounced. In other words, the correlation—whether positive or negative—between the MTA on either side of the semantic TS and the TS weakens as the NS decreases. The typical epoch-loss and accuracy curves of the kernel model corresponding to Fig. 3f are shown in Supplementary Fig. 23. The KM exhibits an unstable training process when the NS falls below 500. Meanwhile, with reduced NS, an increased STD (Fig. 2d) indicates that the stability of alignment decreases with the decrease of NS. Additionally, when aligning the OS with the IS via the RDNet13 kernel and a limited NS of 50, an alignment accuracy of 15 ms was achieved, as shown in Fig. 3d. As illustrated in Fig. 1b, the arc events extracted from both the IS and OS demonstrate a temporally consistent distribution. The failed alignment between the IS and AS, as shown in Fig. 2a, is likely due to inaccuracies in the local time axis of the IS captured by the thermal imager, rather than the limited number of IS images (1800) associated with the arc. As presented in Supplementary Discussion 9, the effect of NS on alignment accuracy based on the lightweight kernel model Mnet2_1 is similar to that of RDNet13. Moreover, the maximum degradation in alignment resolution within the range of 50-9000 samples is within 35 ms. Therefore, in the initial stages of the large-scale search for alignment parameters, adopting a coarse alignment strategy based on a small NS and lightweight KMs is a cost-effective and practical solution.

Figure 3g–i illustrate the effect of the positive-to-negative sample ratio on alignment accuracy. Figure 3g presents the alignment accuracy and the mean STD of MTA across TSs for sample ratios ranging from 1:8 to 8:1. Stratified sampling is applied to divide the training and testing sets. The alignment accuracy is 5 ms for most sample ratios; however, an increased imbalance in the sample ratio leads to a slight degradation in alignment performance. As the sample imbalance increases, the mean STD of MTA across TSs decreases, and the alignment accuracy reduces. Figure 3h presents the relationships between the sample ratio and the maximum training accuracy and MTA. With increasing sample imbalance, the fluctuation in maximum training accuracy remains small, which is attributable to the sufficient expressive power of the KM. As the degree of sample imbalance increases, the MTA also increases. This phenomenon arises because the loss function penalizes samples from the majority class more heavily than those from the minority class do, resulting in higher predictive accuracy for overrepresented samples than for minority-class samples. Consequently, when the majority class dominates the testing set, the overall testing accuracy may appear artificially inflated, failing to reflect actual model performance and compromising the evaluation of alignment accuracy. It is therefore advisable to maintain a positive-to-negative sample ratio as close to 1:1 as practicable. In scenarios with a limited NS, transfer learning algorithms may be employed to compensate for data scarcity. As shown in Fig. 3i, owing to the relatively stable training process of the KM algorithm, the trend of mountain-shaped variation in accuracy remains well-preserved even under increasingly imbalanced sample ratios. Although, the mean STD of MTA reduces as the sample imbalance intensifies, the difference may in the MTA at different TSs diminishes. The quantitative strength of the mountain-peak trend can be characterized by a weighted mean of the rank correlation coefficients between the TSs and MTA on both sides of the maximum value of MTA. This metric serves as a direct indicator, where a larger value corresponds to a more distinct peak-shaped feature.

For scenarios that demand real-time alignment and involve a broad search range of semantic TSs, a two-level alignment strategy can be employed. At the first level, alignment is performed via a lightweight KM trained on a small NS, with coarse TS step size to search for alignment parameters and reduce the computational cost. Given the potentially limited expressive capacity of the KM relative to the data complexity, hyperparameter optimization is necessary to maximize the testing accuracy and increase the prominence of the peak-shaped variation trend of the MTA across different TSs. At the second-level alignment, a narrower search range is defined around the semantic TS identified during the first-level coarse alignment, thereby reducing the computational cost of fine-level alignment. The fine-level alignment is performed via a higher-complexity KM trained on a larger NS with a finer time shift step to search for alignment parameters. The expressive capacity of the KM should be sufficient relative to the sample complexity, and its hyperparameters should be optimized to ensure training stability and to maximize testing accuracy. Additionally, in fine alignment, the samples used in coarse alignment can be leveraged to reduce the number of preprocessing groups required in fine alignment. Meanwhile, the sampling step can be performed before the data preprocessing step, and preprocessing only the sampled samples can also reduce the time consumption of the alignment framework.

### Promotion of alignment architecture

**Fig. 4:**
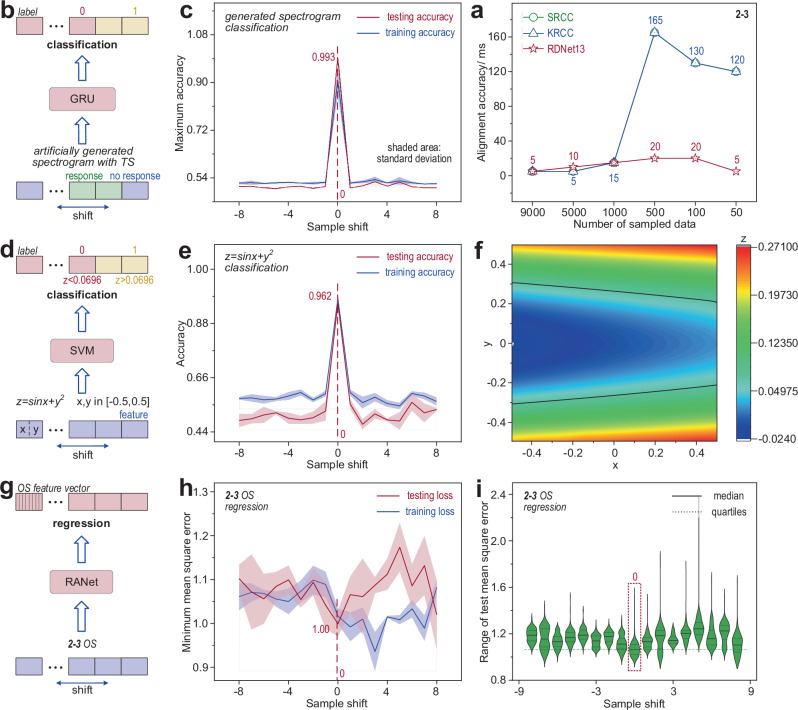
Analysis of alignment architecture promotion. **a** Comparison of alignment accuracy between the RDNet13 kernel model and frequency domain similarity, using the Spearman rank correlation coefficient (SRCC) or the Kendall rank correlation coefficient(KRCC). **b**, **c** Schematic diagrams and alignment results based on the gate recurrent unit (GRU) kernel model used to align the arc response data from the arc igniter with the corresponding labels. **d**, **e** Schematic diagrams and alignment results of aligning the input of artificially constructed functions and corresponding labels on the basis of the support vector machine (SVM) kernel model. **f** Cloud diagram of the corresponding constructed functions. **g**, **h** Schematic diagram and alignment results of aligning optical signals (OS) in dataset **2-3** and feature vectors of the OS via the RANet kernel model. **i** The corresponding distribution of testing accuracy across different sample shifts. Source data are provided as a Source Data file.

Given the limited research on algorithms closely resembling the architecture proposed in this study, an alternative alignment method based on frequency domain similarity (Supplementary Discussion 10) was developed for comparative analysis. This method was evaluated further to illustrate the relative advantages of the proposed architecture. On the basis of the same alignment parameters, a comparison was conducted between the alignment results produced by the frequency domain similarity–based method and those obtained via the neural network–based architecture (Fig. 3d–f), as summarized in Fig. 4a. Although the neural network–based architecture achieves slightly lower alignment accuracy (10 ms) than the frequency domain similarity–based method under the number of samples of 5000, the architecture remains robust under data-limited conditions. In contrast, the accuracy of the frequency domain similarity–based method substantially decreases (120–165 ms) when the number of samples is reduced to 500 or less, whereas the neural network–based architecture maintains superior precision (5–20 ms). The results indicate that, although frequency domain representations of arc responses in the AS modality exhibit measurable similarity, such patterns are not prominent in small-sample analyses and require large datasets for reliable detection. The computational costs of the respective alignment methods are reported in Supplementary Tables 17 and 18. The evolution of numerical modeling has undergone three major paradigm shifts. The first stage is defined by numerical fitting methods, such as least squares, which rely on predefined basis functions. The second stage introduces traditional machine learning algorithms, including support vector machines (SVMs), which eliminate the need for explicit basis functions but operate within limited solution spaces. The third and current stage is characterized by deep learning algorithms, which are initiated by models from the AlexNet era and are capable of learning complex patterns directly from data. Compared with earlier modeling paradigms, the training of deep learning models offers significantly expanded paths for identifying optimal solutions. Using AlexNet as a representative example, the process begins with weight initialization, followed by progressive feature transformation stages that enrich representation through dimensional expansion and spatial reduction of feature maps. Hierarchical connectivity across layers further enhances the expressive capacity of the model, enabling the learning of increasingly abstract features. During training, loss functions guide parameter updates by reinforcing features that improve task performance, thereby systematically directing the model toward accurate classification or regression outcomes. The neural network–based alignment architecture reflects the characteristics of the third modeling paradigm, which involves solving for the target kernel model (KM) through multiple adaptive strategies involving feature aggregation and enrichment, thereby achieving high-quality alignment. In contrast, the frequency domain similarity–based algorithm resembles the paradigms of the first and second stages, employing relatively fixed rules that rely solely on frequency-domain vector similarities. This fundamental limitation explains its significantly lower accuracy than neural network–based alignment when working with a limited number of samples. However, algorithms that rely on relatively fixed approaches empowered by relevant domain knowledge to find solution functions are still recommended because they consume relatively low algorithmic costs. However, in the absence of such prior knowledge, neural networks offer clear advantages. Deep learning models with strong expressive capacity can flexibly adapt to end-to-end learning across diverse data types, including tabular data, images, semantic vectors, and time series. The primary trade-offs of neural network–based approaches include increased demand for data and computational resources. Nonetheless, when domain knowledge is unavailable, the cost associated with acquiring large-scale data and supporting sufficient computational power becomes a necessary investment.

To further evaluate the applicability and reliability of the proposed alignment architecture, extensive experiments were performed on datasets with predetermined alignment parameters. For time series data, to accelerate the experimental process, both the AS spectrograms and the corresponding labels were synthetically generated on the basis of the arc response patterns of the arc igniter. The construction procedure is illustrated in Supplementary Fig. 27, and the detailed algorithm for generating data is provided in Supplementary Algorithm 3. On the basis of the gated recurrent unit (GRU) KM, alignment experiments were conducted on the generated spectrograms and corresponding labels (Fig. 4b), with the sample shift search range set from -8 to +8. In this setup, the frequency response intensity vectors at each time point in the spectrogram were used as feature vectors input into the time series model. The alignment results are shown in Fig. 4c, and the distribution of testing accuracy across different sample shifts is presented in Supplementary Fig. 28. Both the training accuracies and testing accuracies reached their maximum values at a sample shift of 0, indicating that the alignment architecture successfully achieved correct alignment between the time series data and the corresponding labels. To further validate the applicability of the proposed alignment architecture to tabular data, a synthetic dataset was constructed, referring to recent research^52^, via the function $z=\sin x+{y}^{2}$, where $x$ and $y$ were sampled within the range of −0.5 -- + 0.5. Data points with $z > 0.0696$ were assigned a label of 1, whereas those with $z\le 0.0696$ were assigned a label of 0 (Fig. 4d). The feature vectors composed of x and *y*, and the corresponding labels, were aligned via a support vector machine (SVM) KM (Fig. 4d). The alignment results shown in Fig. 4e demonstrate the favorable alignment performance of the architecture. In the multimodal era driven by transformer -based architectures, semantic vectors have become the primary medium for cross-modal information exchange. To evaluate the applicability of the proposed alignment framework in such contexts, alignment was performed between dataset **2-3** OS images and their corresponding 8-dimensional semantic feature vectors (mean, maximum, minimum, range, variance, skewness, kurtosis, and entropy) via the RANet KM (Fig. 4g). The alignment results are presented in Fig. 4h and i. While the training mean squared error (MSE) did not reach its minimum at a sample shift of 0, the testing MSE achieved its lowest value at this point. Moreover, the overall distribution of the testing MSE at a sample shift of 0 was lower than that at other shifts, indicating successful alignment between the OS images and their associated semantic representations. The sample shift–testing MSE curves corresponding to the three alignment trials (Fig. 4g–i) are presented in Supplementary Fig. 29. Among the three, one instance exhibited suboptimal alignment performance, with an alignment resolution exceeding 8 sample shifts. This discrepancy may be attributed to internal balancing mechanisms among the outputs of the multioutput neural network. To increase the robustness of alignment in such challenging scenarios, multiple alignment runs are recommended to increase the reliability of the estimated semantic time shift. Additionally, supplementary alignment experiments are presented in Supplementary Discussion 11, including the alignment between images and labels, tabular data and labels, and input vectors and output values of artificially constructed functions. All the experiments achieved favorable alignment performance. In summary, at the task level, the proposed alignment architecture is applicable not only to regression tasks of KMs but also to classification tasks. At the model level, the architecture accommodates a wide range of model architectures, including traditional machine learning models, time series models, and convolutional neural networks. At the data level, the architecture supports alignment across diverse modalities, including tabular data, time series data, and image data, with corresponding semantic labels or feature vectors. Therefore, this alignment architecture is expected to be applicable to align heterogeneous data pairs in which one modality allows the extraction of semantic labels, semantic vectors, and continuous semantic values. Finally, the time cost of alignment based on the proposed architecture is analyzed in detail in Supplementary Discussion 12.

## Methods

### In situ test of current-carried friction

The detailed configuration of the multispectral acoustic in-situ current-carrying testing system is described in Supplementary Discussion 1. In this system, a pin-on-disk configuration is employed to investigate the tribological behavior under electrical loading. The pin specimen is cylindrical, measuring 7 mm in diameter and 22 mm in length, with a 1 mm corner radius and a surface roughness of less than 0.4 μm on the contact surface. The matching disk specimen, measuring 53 mm in diameter and 8 mm in thickness, features a polished contact surface with no visible scratches. Both pin and disk specimens are prepared through precision machining and polishing processes. Details of the specimen materials, experimental parameters for the current-carried friction tests, and corresponding types of in-situ signals are provided in Supplementary Table 3. The specific current and rotational speed conditions applied to datasets from **2‑2** to **3‑3** are illustrated in Supplementary Fig. 8. To minimize optical background noise for high-fidelity arc detection and reduce acoustic interference, all the experiments were conducted in a darkened laboratory between 23:00 and 06:00 the next day (Beijing Time).

Optical signals were captured via a high-speed camera (Revealer X213) with a sampling frequency of 40 Hz, an exposure time of 25 ms, and a resolution of 1024 × 768 pixels. Simultaneously, infrared data were acquired with a thermal imager (Telops Spark M150), which recorded at 80 Hz over a temperature range of -20 to 350 °C, yielding raw images at a resolution of 640 × 556 pixels. The acoustic signals were collected via a dual-channel system synchronized for data acquisition. The first channel incorporated a wideband acoustic emission sensor (PAC WSa; 1–1000 kHz) connected to a PAC 2/4/6 amplifier. The second channel consisted of an air-coupled sensor (PAC AF62; 1–34 kHz) coupled with a PAC 1281 amplifier. Both channels shared an upper computer (PAC PCI-2) and were controlled by the software (PAC AEwin for PCI2). The signals collected by both sensors were processed through an analog bandpass filter with a frequency range of 1 kHz to 3 MHz. The system supports two acquisition modes: complete waveform acquisition and threshold-based segmented waveform acquisition. For the current-carrying friction test, both airborne and wideband sensors were employed in full waveform acquisition mode, with a sampling frequency of 2 MHz and a preamplifier gain of 40 dB. In contrast, for the acoustic signals energy comparison test (Supplementary Figs. 3, 10, and 18), to facilitate the extraction of energy information, airborne sensors were utilized in threshold-based acquisition mode, with a sampling frequency of 2 MHz, a preamplifier gain of 40 dB, a waveform length of “1k”, and a threshold of 10 dB (the minimum value supported by the system). In these experiments, optical, infrared, and acoustic signals were collected approximately synchronously via synchronized triggering software. The mechanical load signal was measured with a bidirectional force sensor (Huilizhi LZ-LW64) at a sampling frequency of 10 Hz. Current and voltage data were acquired from a programmable power supply (PSY DCPS3014) at a sampling frequency of 1 Hz. The optical, acoustic, and infrared signals were synchronized approximately via synchronous triggering software.

### Impact in-situ test

The impact testing equipment was a small mini cannon, with an accelerated component driven by an electromagnetic coil. The projectile was made of bearing steel, with a diameter of 6 mm and a mass of 6.5 g. The cylindrical projectile was accelerated to 58 m/s by the mini cannon and subsequently impacted a flat specimen made of aluminum alloy with dimensions of 80 × 80 × 4 mm. In-situ signals during the impact process were approximately synchronously collected via high-speed cameras, infrared thermal imagers, and airborne acoustic emission sensors to capture three key moments of the impact. The time shift reference range between the signals was determined by comparing these three moments (Supplementary Fig. 9c), which were subsequently used to validate the alignment effect on the basis of the signals in datasets **2-2** and **2-3**. The measurement surfaces of the optical, infrared, and acoustic sensors were adjusted to focus on the impact location collectively.

The optical, acoustic, and infrared signals were synchronized approximately via synchronous triggering software. The experiment was conducted over four trials. In the first and second experiments, the exposure time of the high-speed camera was 25 ms, with sampling frequencies of 40 Hz for the high-speed camera and 80 Hz for the infrared thermal imager. In the third experiment, the exposure time of the high-speed camera was reduced to 12.5 ms, with sampling frequencies of 80 Hz for both the high-speed camera and the infrared thermal imager. In the fourth experiment, the exposure time of the high-speed camera was further reduced to 6.25 ms, with sampling frequencies of 160 Hz for both the high-speed camera and the infrared thermal imager. In these experiments, acoustic signals were collected via the airborne acoustic emission sensor in full waveform mode, with a sampling frequency of 2 MHz and a preamplifier gain of 40 dB. The signals were processed through an analog bandpass filter with a frequency range of 1 kHz to 3 MHz.

### In situ test of the arc response of the igniter

A dedicated calibration experiment was conducted to quantify the time shift between the optical and acoustic signals of the system. An arc was generated in a darkroom with the igniter from Supplementary Fig. 9d. The synchronous triggering software coordinated the high-speed camera and acoustic emission sensor to record the event. The measured time shift between the optical and acoustic recordings of the arc ignition served as the ground-truth reference for validating the temporal alignment of the signals in datasets from **3-1** to **3-3**. The experiment was conducted seven times, with an optical signal sampling frequency of 40 Hz and an exposure time of 25 ms. Acoustic signals were collected via an air-coupled acoustic emission sensor in full waveform acquisition mode, with a sampling frequency of 2 MHz and a preamplifier gain of 40 dB. The signals were processed through an analog bandpass filter with a frequency range of 1 kHz to 3 MHz.

### Synchronize triggering software

To bypass the hardware synchronization process and accelerate the acquisition of experimental data for verifying the alignment architecture, synchronization triggering software (the software and scripts involved in the Methods section are detailed at Public repository^53^) was used on the basis of preset click steps, to approximately synchronize the activation of the corresponding start collection buttons on the user interface, enabling the approximately synchronous acquisition of heterogeneous signals. This method reduces random errors in the time shift between signals compared with manually activating the start collection buttons. Simultaneously, to verify the alignment results of the alignment architecture, via synchronization triggering software, in situ impact tests and arc response tests of the igniter were conducted to obtain the reference time shift between heterogeneous signals.

### Signal processing

The collected optical images are saved in TIFF format, and, via the script, the images are cropped to a uniform size of 672×672×3 pixels. Via scripts the cropped optical images are subsequently converted via histogram equalization and a dynamic threshold adaptive algorithm. Both the cropped and the converted image sets were archived for subsequent analysis. For the cropped raw images of the optical signal in dataset **2-3**, eight statistical features—mean, maximum, minimum, range, variance, skewness, kurtosis, and entropy—were computationally extracted via the script and exported to an Excel file for subsequent analysis. The optical images in datasets from **2-2** to **3-3** were manually annotated. Images with arcs were labeled positive samples (“1”), whereas those without arcs formed the negative class (“0”). The manually annotated image set and the corresponding arc response frequency are provided in Supplementary Table 4.

The infrared signal images in dataset **2-3** were uniformly scaled to a temperature range of 19 °C to 39 °C and exported as PNG format images for storage. Using the script, the images were cropped to 224 × 224 × 3 pixels and archived. Semantic labels were automatically annotated via a script using the criteria of unrecognized green areas. Images showing the arc damage response were labeled as 1, corresponding to positive samples, whereas infrared images without arc damage were labeled as 0, corresponding to negative samples. The corresponding arc damage frequencies are listed in Supplementary Table 4.

For the acoustic signals collected in threshold-based mode, the data from the acoustic signal energy analysis experiment (Supplementary Figs. 3, 10, and 18) were extracted via software (AEwin for PCI2) to obtain energy feature data. In contrast, for acoustic signals collected in full waveform mode, the raw data were exported as a sequence of CSV files and saved for subsequent transformation. The waveform data were converted into frequency and time‒frequency domain representations. Frequency domain representations were used to validate the alignment methods via frequency domain similarity (Fig. 4a and Supplementary Fig. 26). In contrast, time-frequency domain representations serve to validate the proposed heterogeneous data alignment architecture. For the conversion of the frequency domain representation, based on the script, the acoustic signals were segmented into 25-ms signal segments, denoted as $x[n]$ from the original CSV file. The conversion formula for obtaining the frequency domain signal ${x}^{{{\rm{f}}}}[k]$ from the time domain signal segment ${x}^{{{\rm{f}}}}[k]$ is shown in Eq. (4):

4 $${x}^{{{\rm{f}}}}[k]=\sum_{n=0}^{{N}^{s}-1}x[n]\cdot {e}^{-j\cdot \frac{2\pi }{N}\cdot {nk}}$$

where, ${x}^{{{\rm{f}}}}[k]$ denotes the frequency domain signal amplitude of the *k*th frequency component, and where *x*[*n*] denotes the amplitude of the time domain signal with length ${N}^{{{\rm{s}}}}$. The spectrum vector ${x}^{{{\rm{f}}}}[k]$ was scaled such that the sum of the elements of the spectrum vectors equals 1000. The resulting frequency domain signal vector was saved as a CSV for easy inspection, and subsequently, the CSV file was converted to a PKL file for subsequent use. For the conversion to the time-frequency domain representation, the waveform stream data were segmented into 25-ms signal segments via the script. On the basis of the obtained time-domain signal segment, $x[n]$ the conversion formula for the time-frequency domain representation ${x}^{{{\rm{tf}}}}[m,k]$ is shown in Eq. (5):

5 $${x}^{{{\rm{tf}}}}[m,k]=\sum_{n=0}^{{N}^{s}-1}x[n]\cdot w[n-{mH}]\cdot {e}^{-j\cdot \frac{2\pi }{N}\cdot {nk}}$$

where, ${x}^{{{\rm{tf}}}}[m,k]$ denotes the time-frequency domain representation intensity of the *k*th frequency component in the *m*th frame, and where $w[n-{mH}]$ denotes the window function with a frame shift of $H$. The window function used is a Hamming window, with a 50% overlap between adjacent windows. For the conversion to the time-frequency domain representation with a frequency range of 0–1 MHz, each window corresponds to 446 points of the time-domain signal, corresponding to a time length of 0.223 ms. For the conversion with a frequency range of 0–34 kHz, each window corresponds to 2382 points of the time-domain signal, with a corresponding time length of 1.191 ms. The time‒frequency domain representation was subsequently normalized and saved as a PNG image. For the conversion with spectrogram in Supplementary Fig. 27 (the time corresponding to each signal segment: 400 ms), each window corresponds to 9650 points of the time-domain signal, with a corresponding time length of 8.066 ms. Additionally, The time‒frequency domain representation was transformed via the “lg” function, normalized, and saved as a PNG image for future use.

### Other data generation

The steps for generating the spectrogram of the arc response data and the corresponding labels, for the arc igniter, are shown in Supplementary Fig. 27 and Supplementary Algorithm 3. Data generation is based on script. The input vector, output value, and corresponding category label for functions $z=\sin x+{y}^{2}$ and $z=\left|x\right|+{{\rm{step}}}\left(y\right)$ are generated via script.

### Data alignment

The mathematical expressions for data alignment via machine learning kernel models are presented in Eqs. (1) and (3), along with the implementation steps outlined in Supplementary Algorithm 1. The alignment process is implemented via the script. The parameter settings for the alignment experiment using this architecture are listed in Supplementary Table 5. Detailed information about the kernel model used is provided in Supplementary Tables 6-13. In Figs. 3a, d, g and Supplementary Fig. 24a, the alignment accuracy was calculated based on the mean of the maximum testing accuracy across different time shifts in three experiments. Regarding the mean standard deviation of the maximum testing accuracy, the standard deviation at different time shifts was first calculated based on the corresponding maximum test accuracy of three experiments, and the average standard deviation was calculated based on the obtained standard deviation at different time shifts. In Fig. 4a, the alignment accuracy obtained from alignment architecture was calculated based on the mean of the maximum testing accuracy across different time shifts in three experiments.

The mathematical expressions for the alignment method, which are based on frequency domain similarity, are presented in Eq. (1) and Supplementary Equation (1), with the implementation steps outlined in Supplementary Algorithm 2. The parameter settings for the alignment experiment, which are based on this method (Fig. 4a and Supplementary Fig. 26), are presented in Supplementary Table 5. The alignment is implemented via a script. In Fig. 4a, the alignment accuracy based on frequency domain similarity was calculated based on the mean of the rank correlation coefficient across different time shifts in three experiments.

### Arc detection model

The hyperparameter settings for the optical-based arc detection model (Supplementary Figs. 20d-f) are provided in Supplementary Table 5. The infrared signal-based arc detection is implemented via a script according to green unrecognized areas. The acoustic-based arc detection model (Figs. 2c–i) corresponds to the kernel model in the alignment architecture based on machine learning, with its trained hyperparameters listed in Supplementary Table 5.

### Class activation mapping

The class activation mapping was calculated via the last convolutional layer corresponding to the model in epoch 53 (Fig. 2e).

### Hardware and software

The operating system used in this study was Windows 10, supported by Intel Core i5, NVIDIA GeForce 3090 hardware, and 48 GB of RAM. The software packages utilized included Python 3.9.6, PyTorch 2.2.2+cu118, Scikit-learn 1.5.1, and the Sublime Text Code Editor.

## Supplementary information

Supplementary Information

Description of Additional Supplementary Files

Supplementary Movie 1

Supplementary Movie 2

Supplementary Movie 3

Supplementary Movie 4

Supplementary Movie 5

Transparent Peer Review file

## Source data

Source Data

## Data Availability

The authors declare that the main data supporting the findings of this study are available within the article and its Supplementary Information files. Source Data are provided with this paper. All other relevant data are available from the corresponding author upon request. The datasets used for data alignment, as well as training and testing of the arc detection models have been deposited in the public repository (https://www.scidb.cn/en/s/iMnaii). Source data are provided with this paper.

## Code availability

The synchronize triggering software and code for data generation, data processing, data alignment, and obtaining arc detection models have been deposited in the public repository^53^.
