# Supplementary material for: Machine learning-driven alignment architecture of heterogeneous data with transient varying semantics
Source: Nat Commun. 2026 Apr 23;17:5604. doi: 10.1038/s41467-026-72377-w (PMC13316067; doi:10.1038/s41467-026-72377-w)
Supplement: Supplementary file 1 — Supplementary Information [file 41467_2026_72377_MOESM1_ESM.pdf]

Supplementary information for

Machine Learning-driven Alignment Architecture of Heterogeneous Data with Transient Varying Semantics

Chaofan Li[1], Zhichao Ma[1,2 *], Yangzhi Zeng[1], Zaizheng Yang[1], Jiakai Li[1], Zheng Yang[1], Junming Xiong[1], Shichao Niu[3], Zhe Wang[3], Hongwei Zhao[1,2], Luquan Ren[3]

[1] *School of Mechanical and Aerospace Engineering, Jilin University, Changchun, 130025, China*

[2] *Key Laboratory of CNC Equipment Reliability, Ministry of Education, Jilin University, Changchun, 130025, China*

[3] *Key Laboratory of Bionic Engineering Ministry of Education, Jilin University, Changchun, 130025, China*

**E-mail address of the corresponding author: zcma@jlu.edu.cn*

Contents of Supplementary Tables

Supplementary Table 1. **Abbreviations and full names of the technical terms**	Page 4
Supplementary Table 2. **The estimated cost of the revised in-situ testing system**	Page 6
Supplementary Table 3. **The dataset numbering and corresponding conditions of the orthogonal experiments**	Page 12
Supplementary Table 4. **Label information of optical and infrared signals**	Page 15
Supplementary Table 5. **Training parameters of the kernel models**	Page 17
Supplementary Table 6. **The information of the revised neural networks**	Page 18
Supplementary Table 7. **The structure of the gate recurrent unit**	Page 18
Supplementary Table 8. **The structure of the multi-layer perceptron**	Page 18
Supplementary Table 9. **The structure of MiniNet2_*x***	Page 18
Supplementary Table 10. **The structure of MiniNet3**	Page 18
Supplementary Table 11. **The structure of MiniNet4**	Page 18
Supplementary Table 12. **The structure of Revised DenseNet13**	Page 19
Supplementary Table 13. **The structure of Revised DenseNet41**	Page 19
Supplementary Table 14. **The time cost of signal preprocessing**	Page 31
Supplementary Table 15. **Training time costs of machine learning models**	Page 31
Supplementary Table 16. **The inference time costs for the deployed model to detect the arc**	Page 32
Supplementary Table 17. **The time costs of alignment based on Revised DenseNet13**	Page 39
Supplementary Table 18. **The time costs of alignment on the basis of frequency domain similarity**	Page 39

Contents of Supplementary Algorithms

Supplementary Algorithm 1. **Neural network-driven data alignment algorithm architecture**	Page 11
Supplementary Algorithm 2. **The frequency domain similarity-driven data alignment method**	Page 38
Supplementary Algorithm 3. **Generate arc response label and corresponding spectrogram**	Page 40

Contents of Supplementary Figures 1-3

Supplementary Fig. 1. **The overall layout of the testing system, including a pin-disc-type friction testing instrument, a high-speed camera, an infrared thermal imager and two acoustic emission sensors.**	Page 6
Supplementary Fig. 2. **The force loading mechanism and load response corresponding to the three loading modes.**	Page 7
Supplementary Fig. 3. **The variations in the acoustic signal energy feature (obtained from an airborne acoustic emission sensor) and normal load with contact conditions controlled by micrometer heads.**	Page 7

Contents of Supplementary Figures 4-31

Supplementary Fig. 4. **The mechanism of arc generation under current-carried friction conditions.**	Page 8
Supplementary Fig. 5. **Typical signal images.**	Page 9
Supplementary Fig. 6. **Labels obtained from optical signals and infrared signals to determine arc damage.**	Page 9
Supplementary Fig. 7. **Typical acoustic signals, including the time-domain, frequency-domain, and time-frequency domain representations of the signals.**	Page 10
Supplementary Fig. 8. **The experimental conditions for datasets from 2-2 to 3-3**	Page 13
Supplementary Fig. 9. **Reference time-shift range between the signals of the testing system.**	Page 14
Supplementary Fig. 10. **Analysis of the energy of the acoustic signals.**	Page 16
Supplementary Fig. 11. **Comparison of the acoustic signal spectrograms with and without lg function transformation.**	Page 16
Supplementary Fig. 12. **The effectiveness of infrared signals in arc monitoring and detection.**	Page 20
Supplementary Fig. 13. **Typical time shift–maximum testing accuracy curve of alignment between optical signals and acoustic signals in 2-3 sets, away from the semantic time shift.**	Page 20
Supplementary Fig. 14. **Shift–maximum accuracy curves and accuracy distributions for aligning optical signals and acoustic signals in datasets 2-2 and 2-3.**	Page 22
Supplementary Fig. 15. **The time shift-maximum accuracy curve and accuracy distribution corresponding to the alignment of optical signals and acoustic signals in datasets 3-1 and 3-2.**	Page 23
Supplementary Fig. 16. **Friction form of the test specimens and alignment results of the optical signals and acoustic signals in dataset 3-3 of the experiments.**	Page 24
Supplementary Fig. 17. **Interpretability analysis of typical acoustic signals.**	Page 26
Supplementary Fig. 18. **The variations in the acoustic signal energy and normal load with varying electrification conditions, occurring in the case of slight separation of the friction pair under the constant displacement loading mode.**	Page 26
Supplementary Fig. 19. **Influence of the frequency range of the spectrogram from the acoustic signal collected by the airborne acoustic emission sensor on the alignment.**	Page 27
Supplementary Fig. 20. **The effect of optical signal preprocessing on arc detection model performance with optical images as input**.	Page 29
Supplementary Fig. 21. **Application of the arc detection model with optical images processed by a dynamic threshold adaptive as input**.	Page 30
Supplementary Fig. 22. **The typical epoch-loss and accuracy curves corresponding to the kernel model for various numbers of samples and time shifts in Fig. 3c.**	Page 33
Supplementary Fig. 23. **The typical epoch-loss and accuracy curves corresponding to the kernel model for various numbers of samples and time shifts in Fig. 3f.**	Page 34
Supplementary Fig. 24. **Analysis of the effect of the number of samples on alignment accuracy.**	Page 35
Supplementary Fig. 25. **The typical epoch-loss and accuracy curves corresponding to the kernel model for various numbers of samples and time shifts in Supplementary Fig. 24c.**	Page 36
Supplementary Fig. 26. **The alignment method on the basis of frequency domain similarity.**	Page 38
Supplementary Fig. 27. **Method for generating corresponding artificial spectrograms and labels on the basis of the spectrogram of the arc response of the arc igniter.**	Page 40
Supplementary Fig. 28. **The shift-accuracy distribution corresponding to Fig. 4c.**	Page 41
Supplementary Fig. 29. **The shift-maximum testing accuracy curve of three repeated alignments corresponding to Fig. 4h.**	Page 41
Supplementary Fig. 30. **Promotion of the alignment architecture (supplements for Figure 4).**	Page 43
Supplementary Fig. 31. **Promotion of the alignment architecture (supplements for Fig. 4).**	Page 44

Contents of Supplementary Discussion

Supplementary Discussion 1. **Instrument and alignment requirements**	Page 5
Supplementary Discussion 2. **Datasets used for the alignment study**	Page 12
Supplementary Discussion 3. **Feasibility of arc detection via infrared signals**	Page 20
Supplementary Discussion 4. **Repetitive and supplementary alignment**	Page 21
Supplementary Discussion 5. **Interpretability analysis of the acoustic signals**	Page 25
Supplementary Discussion 6. **The impact of the spectrogram frequency range on alignment**	Page 27
Supplementary Discussion 7. **Feasibility of arc detection via optical signals**	Page 28
Supplementary Discussion 8. **Cost evaluation of the arc detection model**	Page 31
Supplementary Discussion 9. **Influence of the number of samples on alignment accuracy**	Page 35
Supplementary Discussion 10. **The alignment method based on frequency domain similarity**	Page 37
Supplementary Discussion 11. **Supplement of alignment architecture promotion**	Page 42
Supplementary Discussion 12. **Cost evaluation of alignment architecture**	Page 45

Supplementary Table 1. **Abbreviations and full names of the technical terms**

Abbreviations	Meanings	Abbreviations	Meanings
AE	Acoustic emission	MSE	Mean squared error
AS	Acoustic signal	MTA	Maximum testing accuracy
C	Capacitor	NS	Number of samples
CAM	Category activation mapping	OS	Optical signal
CDL	Constant displacement loading	PKL	Pickle
CLL	Constant load loading	PNG	Portable network graphics
CMLL	Constant minor load loading	R	Resistor
CSV	Comma-separated values	RAM	Random access memory
GRU	Gate recurrent unit	RANet	Revised AlexNet
IS	Infrared signal	RDNet13	Revised DenseNet13
KM	Kernel model	RDNet41	Revised DenseNet41
KRCC	Kendall rank correlation coefficient	RMB	Renminbi
MLP	Multi-layer perceptron	RMNet	Revised MobileNet V3 small
LR	Learning rate	RRNet10	Revised ResNet10
MB	Megabyte	RRNet18	Revised ResNet18
MNet2_1	MiniNet2_1	RSNet1	Revised Squeezenet 1.0
MNet2_2	MiniNet2_2	SRCC	Spearman rank correlation coefficient
MNet2_4	MiniNet2_4	STD	Standard deviation
MNet2_8	MiniNet2_8	SVM	Support vector machines
MNet2_16	MiniNet2_16	TIFF	Tag image file format
MNet2_32	MiniNet2_32	TS	Time shift
MNet2_64	MiniNet2_64	V	Velocity
MNet3	MiniNet3	U	Voltage
MNet4	MiniNet4		

Supplementary Discussion 1. **Instrument and alignment requirements**

To clearly demonstrate the significance and applicability of the heterogeneous data alignment architecture, a case study on high-throughput detection of the arc damage frequency under current-carried friction conditions, using optical signals (OSs), infrared signals (ISs) and acoustic signals (ASs), is introduced. The abbreviations and full names of the technical terms used in this paper are provided in Supplementary Table 1 and will not be repeated in the following text. Previously, we developed a multispectral acoustic spectrum current-carrying friction in-situ testing system [1]. The testing system was used to perform current-carrying friction tests on the friction pair between pin-shaped specimens and disc-shaped specimens. The maximum speed, maximum wear radius, maximum loading current, and maximum loading voltage of the system were 150 r/min, 23 mm, 10 A, and 10 V, respectively. In addition, a high-speed camera, an infrared thermal imaging instrument, and a wideband acoustic emission (AE) sensor were integrated into the testing system, enabling the acquisition of mechanical, electrical, optical, temperature field, and acoustic information. On the basis of this system, an airborne AE sensor was integrated, and the friction pair normal load control mechanism was modified to support in-situ testing of arc damage under current carried friction conditions. The estimated cost of the complete testing system is provided in Supplementary Table 2. Supplementary Fig. 1 illustrates the modified system. Intuitively, the placement of a wideband AE sensor in wedge-shaped fixtures may hinder the collection of acoustic information corresponding to the arc response. Instead, most of the information collected by the sensor may correspond to the vibration response of the fixture caused by friction [1]. V. Pandiyan and R. Drissi-Daoudi utilized airborne AE sensors to monitor defects in the laser additive manufacturing process [2] which involves the melting of metals similar to the process during arc generation. Consequently, an airborne sensor was integrated into the system to detect the arc. The position of an airborne AE sensor was adjusted to face the friction pair. In previous versions of the testing system, the normal load of the friction pair was controlled by a pneumatic cylinder. However, the constant load performance of the system was inadequate, and the constant displacement control loading mode could not be supported. Therefore, the normal load control mechanism of the friction pair was modified. The revised control mechanism was composed mainly of springs, supports, and micrometer heads. Through this revisd mechanism, the constant displacement loading (CDL) mode, constant load loading (CLL) mode, and constant minor load loading (CMLL) mode can be achieved. The control component in Supplementary Fig. 2a corresponds to the CDL mode, where two springs are used to lift the floating component, and a micrometer head is used to precisely control the downward location of the floating component, ensuring accurate control of the absolute position of the pin-shaped specimen and the generation of the arc. The removal of the significant and minor springs in the component corresponds to the CLL mode, as shown in Supplementary Fig. 2b. In contrast, the removal of the significant spring corresponds to the CMLL mode, as shown in Supplementary Fig. 2c. The normal load variation corresponding to the CDL mode is shown in Supplementary Fig. 2d. At certain moments, the corresponding load is 0, indicating that the two specimens were not in contact. The normal load response corresponding to the CLL mode is shown in Supplementary Fig. 2e, and the normal load fluctuation range was approximately 13–23 N. Supplementary Fig. 2f shows the load response corresponding to the CMLL mode, with a range of approximately 1–11 N. In the CDL mode, the normal relative position of the two specimens can be precisely controlled via micrometer heads, enabling the accurate regulation of arc generation for the decoupling analysis of arc and friction damage. The CLL mode served as a comparative analysis, whereas the CMLL mode facilitated more efficient acquisition of in-situ signals corresponding to arc generation conditions. Supplementary Fig. 3 shows the energy variation under friction and no-friction conditions controlled by a micrometer head. First, the friction pair was separated, and the micrometer head was rotated to bring the friction pair into slight contact. The micrometer head was then rotated back to separate the current-carrying friction pair. The variation in the normal load clearly illustrates this process, with a significant increase in energy during the friction contact stage, indicating effective normal displacement control by the micrometer head and reliable monitoring by the airborne AE sensor. The settings of the CDL mode were on the basis of the principle of arc generation, as shown in Supplementary Fig. 4. The current-carrying friction pair can be modeled as an abstract system composed of a power source to provide voltage (U), a resistor (R), and a capacitor (C). The connected part of the friction pairs is modeled as a resistor, and the unconnected part can be equivalent to a capacitor. As the friction pair dynamically evolves, the equivalent R and C also dynamically vary accordingly, and arc damage corresponds to the instantaneous breakdown of the capacitance. When the distance between a specific part of the friction pair and the voltage at both ends is appropriate, the air in the middle of the friction pair is punctured, generating plasma and a large amount of heat, which leads to the melting and splashing of metal

on the surface of the friction pair and causes material transfer between the two friction pairs.

Supplementary Table 2. **The estimated cost of the revised in-situ testing system**

Modules of the testing system	The estimated cost
Self-developed current-carrying friction testing instrument	50,000 RMB
High-speed camera (Revealer X213)	400,000 RMB
Infrared thermal imaging instrument (Telops Spark M150)	600,000-800,000 RMB
AE sensor and supporting acquisition system（PAC Wsa/AF62）	150,000 RMB
Total	1,200,000-1,400,000 RMB

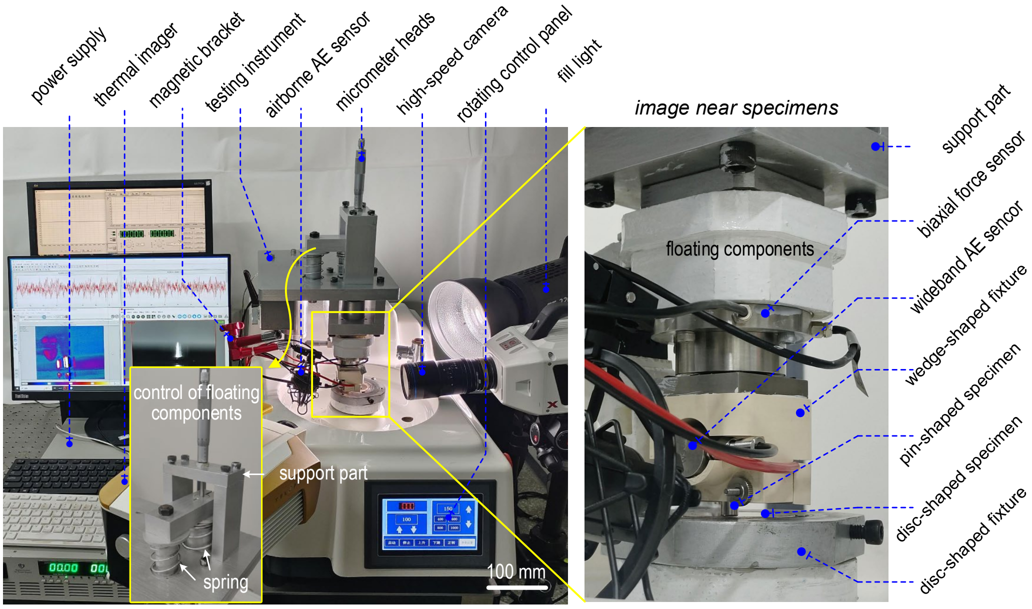

Supplementary Figure 1. **The overall layout of the testing system, including a pin-disc-type friction testing instrument, a high-speed camera, an infrared thermal imager and two acoustic emission (AE) sensors.**

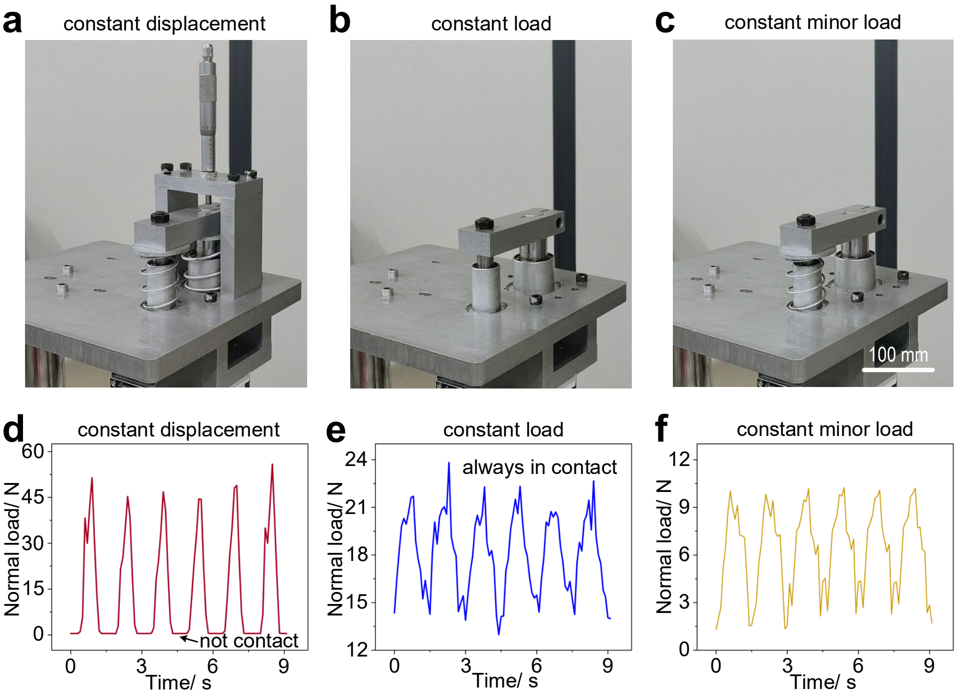

Supplementary Figure 2. **The force loading mechanism and load response corresponding to the three loading modes. a-c** Loading mechanisms corresponding to the constant displacement loading mode, constant load loading mode, and constant minor load loading mode, respectively. **d–f** Time–normal load curves corresponding to the three loading modes. Source data are provided as a Source Data file.

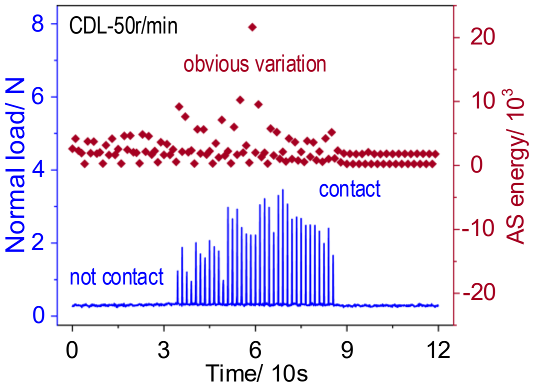

Supplementary Figure 3. **The variations in the acoustic signal (AS) energy feature (obtained from an airborne acoustic emission sensor) and normal load with contact conditions controlled by micrometer heads.** Source data are provided as a Source Data file.

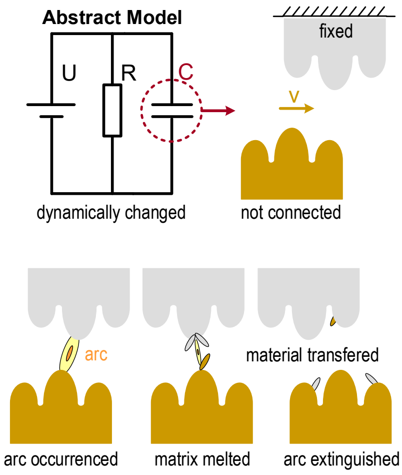

Supplementary Figure 4. **The mechanism of arc generation under current-carried friction conditions.**

In the application of artificial intelligence to specific fields, the synergy between domain knowledge and artificial intelligence modeling is often emphasized. First, the strength of the brain in comparative analysis is leveraged to analyze the OS, IS or AS data collected by the system. Typical examples of OSs and ISs are shown in Supplementary Fig. 5. Supplementary Fig. 5a illustrates typical OS captured by high-speed cameras. OS acquisition was conducted in a dark room to prevent light interference from reducing the visibility of the captured arc phenomenon. The upper left image shows the OS collected in a light environment to demonstrate the field of view, indicating the locations of the pin-shaped specimen and the disc specimen. The upper right image shows the OS in a dark room without an arc, with no bright areas visible in the image. The image in the lower right corner shows the OS corresponding to a typical, obvious arc response. The melted metal was sprayed outward from the friction pair, and the strong reflection of the arc light caused the contour of the bottom of the pin-shaped specimen to be visible. The lower left image shows the OS corresponding to the weak arc response, manifested as a small bright spot. Supplementary Fig. 5b shows a typical IS. The upper left image shows the signal collected without arc damage, whereas the upper right image shows the corresponding IS for evident arc generation. The green color in the central area of the arc indicates that the temperature at this location exceeds the selected range (350°C). The image in the lower left corner shows small bright spots at the friction pair, and further investigation is needed to determine whether these bright spots correspond to arc damage. Additional OSs and ISs are shown in Supplementary Movies 1 and 2, respectively. The consistency of labels derived from concurrent IS and OS signals was evaluated. Labeling according to small bright spots and the unidentified green area in the IS resulted in a temporal distribution of events that was inconsistent with the OS reference. However, the labels derived from the unidentified green area aligned well with the OS in terms of the overall trend (Supplementary Fig. 6), indicating that the unrecognized green area is a more reasonable criterion for detecting arc generation from the IS. The labeling rules for OS and IS are shown in Supplementary Figs. 5a and 5b. Given the large volume of data involved (tens of millions of OS and IS segments), manual analysis is infeasible due to the inherent limitations of human processing capacity. Therefore, the development of automated detection methods is essential for enabling robust, data-driven identification of arcs.

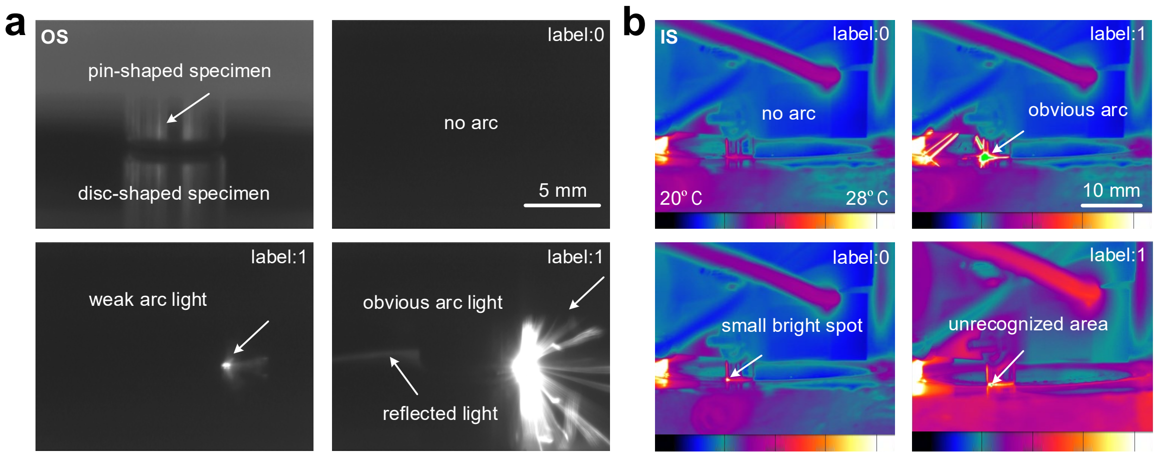

Supplementary Figure 5. **Typical signal images, a** Typical optical signal (OS). **b** Typical infrared signal (IS). Source data are provided as a Source Data file.

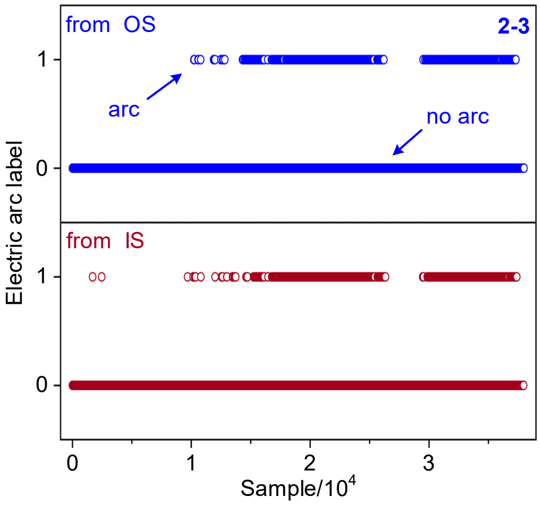

Supplementary Figure 6. **Labels obtained from optical signals (OS) and infrared signals (IS) to determine arc damage.** Source data are provided as a Source Data file.

Supplementary Fig. 7 shows the typical AS, which primarily consists of three types. For signals collected by a wideband AE sensor, the background response is evident in the low-frequency range, approximately 0 MHz (Supplementary Figure 7a). The response corresponding to conventional friction is noticeable in the frequency range from 0 to 0.1 MHz (Supplementary Figure 7b). In contrast, the response of signals corresponding to fluctuating friction was evident in the high-frequency range and fluctuated significantly along the time axis (Supplementary Figure 7c). With respect to the signals collected by the airborne AE sensor simultaneously, the response of the signal corresponding to the background is evident at approximately 20 kHz, with a relatively concentrated frequency distribution (Supplementary Figure 7d). The response corresponding to conventional friction is observed between 8 and 22 kHz, with a peak frequency at approximately 18 kHz (Supplementary Figure 7e). In contrast, the response corresponding to fluctuating friction is evident from 0--30 kHz and fluctuates significantly along the time axis (Supplementary Figure 7f). A comparison of the time-domain representations in Supplementary Fig. 7c and Supplementary Fig. 7f demonstrates that the synchronization between the signal obtained from the wideband sensor and the signal received from the airborne sensor is excellent. Additional acoustic signals are provided in Supplementary Movie 3. Additionally, through comparative analysis, the relationships between the spectrogram and the initiation and cessation of friction events can be identified. However, for the spectrogram during the friction process, intuitive perceptions of its stability or variability along the time axis remain qualitative and imprecise, lacking a definitive basis for clear differentiation. Although the attention mechanism of the brain is a product of successful evolutionary adaptation, its characteristics — such as low data perception, limited

throughput, and finite storage — create a fundamental mismatch with the demands of modern data analysis. This cognitive bottleneck is especially pronounced in the analysis of complex acoustic signals for arc detection. Therefore, it is essential to employ intelligent computational approaches, such as neural networks, to overcome these biological constraints and enable accurate, large-scale signal analysis. It is currently unclear whether ASs are related to the arc response. If a connection exists, its temporal dynamics remain elusive: is the relevant AS generated before, during, or after the arc response? This ambiguity extends to causality—whether a specific system vibration induces the arc, whether the arc generation process itself produces a unique AS, or whether the arc causes a postevent impact that generates a discernible AS signature. Therefore, employing synchronous hardware triggers to align the OS or IS with the AS may not yield the semantically precise labels required for the AS segments. Additionally, in arc damage testing of current-carried friction, unlike in bearing fault diagnosis where labels can be directly obtained, the transient and intermittent nature of arc damage further complicates the acquisition of labels. Therefore, a heterogeneous data alignment architecture is urgently needed to align OS and IS with AS, enabling the extraction of semantic labels for AS to support AS analysis and high-throughput arc testing via AS.

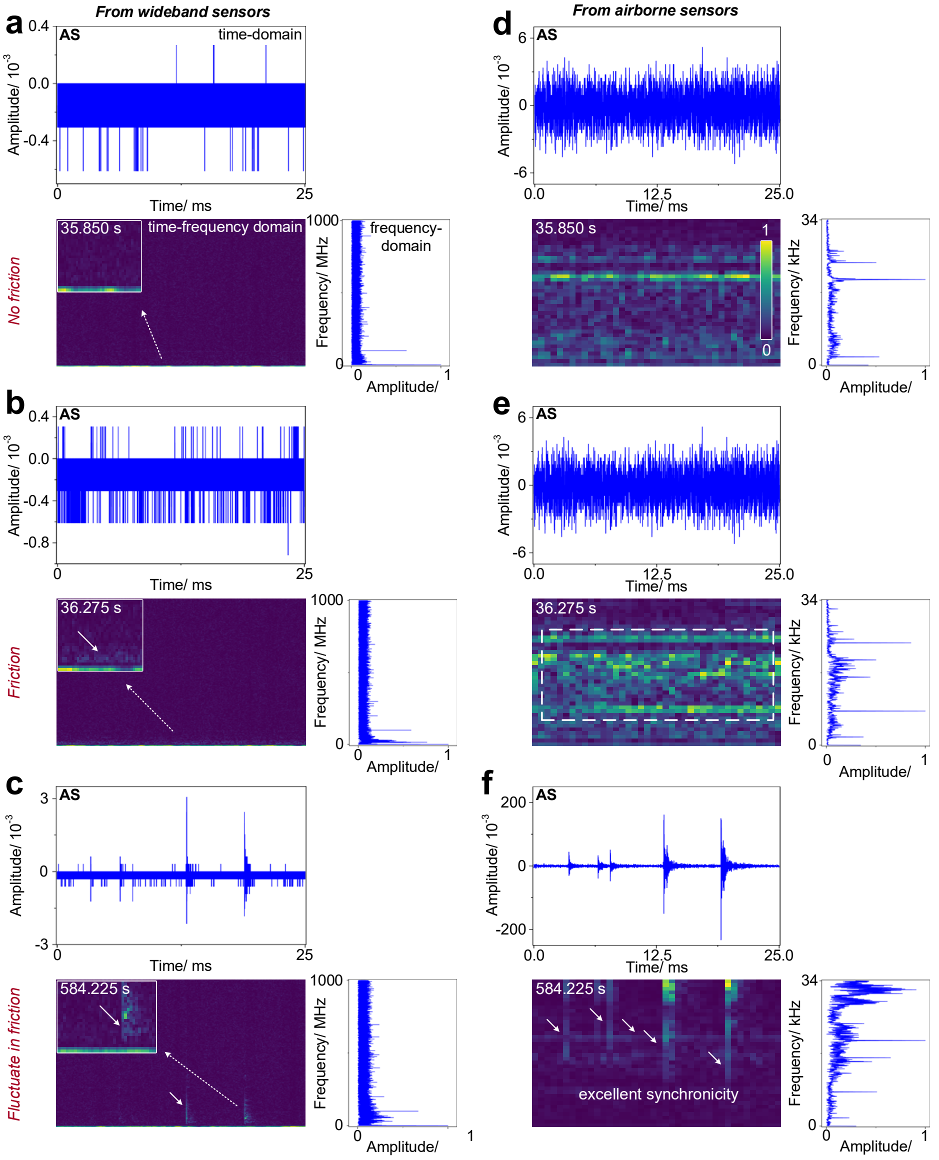

Supplementary Figure 7. **Typical acoustic signals, including the time-domain, frequency-domain, and time-frequency domain representations (spectrograms) of the signals. a-c** Typical signals collected by the wideband acoustic emission sensor. **d-f** Typical signals collected by the airborne acoustic emission sensor. (The color bar indicates the normalized intensity, scaled to the [0, 1] range for each spectrogram individually.) Source data are provided as a Source Data file.

Supplementary Algorithm 1. **Neural network-driven data alignment algorithm architecture**

**Input:** the spectrogram list of AS and label list obtained from OS or IS .	
1	initialization, including search ranges of time shift parameter t, model training parameters and number of samples N, etc.
2	**for** t in range list:
3	randomly extract equal proportional labels of “0” and “1” from the label list to form a list $\{y_1[0,0], y_1[1,0], \cdots, y_1[N-1,0]\}$.
4	extract the corresponding spectrogram list of AS $\{F_2^{\text{stft}}[0,t], F_2^{\text{stft}}[1,t], \cdots, F_2^{\text{stft}}[N-1,t]\}$.
5	establish a dataset and the dataset is divided into training and testing sets.
6	train the model F_{θ_t} based on Equation (3).
7	calculate the maximum testing accuracy of the model F_{θ_t} in training.
8	obtain the time shift parameter t in the maximum testing accuracy.
9	calculate the time shift T_{n} between signal $x_1[n,0]$ and $x_2[n,0]$ based on Equation (3).

Supplementary Discussion 2. **Datasets used for the alignment study**

To support the alignment and application research of multispectral acoustic signals, a series of orthogonal experiments were conducted with rotational speed, loading current, and loading mode as raw variables. In current-carried friction testing, the performance of friction pairs is typically evaluated through equivalent current-carried experiments. To ensure the applicability of the conclusions drawn in this study, the parameter settings for the orthogonal experiments closely matched the range supported by the developed testing instrument and were comparable to those of standard commercial systems (e.g., MS-MT9000). The wear radius of the experiment was uniformly set to 23 mm, with speeds of 50 r/min, 100 r/min, and 150 r/min. The loading voltage was uniformly set to 10 V, while the loading currents were 2 A, 5 A, and 8 A, respectively. The loading modes included constant displacement loading (CDL), constant load loading (CLL), and constant minor load loading (CMLL). As summarized in Supplementary Table 3, the experiment consisted of three groups. In the first group, optical signals (OSs) were collected to verify the reliability of the arc damage identification method based on the OS. In the second group, OSs, infrared signals (ISs), and acoustic signals (ASs) were collected for alignment research and comparative analysis of multispectral acoustic spectrum signals. In the third group, OS and AS data were collected to further validate the reliability of the proposed alignment method. To evaluate the applicability of the proposed architecture efficiently and comprehensively, orthogonal experimental conditions were applied in a set of experiments (**2-2** to **3-3**). The variations in speed and current over time for these experiments are illustrated in Supplementary Fig. 8. In experiments numbered **1-1-1** to **2-3**, 7075 Al alloy pin-shaped specimens and CuCrZr alloy disc-shaped specimens were utilized. To further assess the generalizability of the proposed architecture, the disc materials in experiments from **3-1** to **3-3** were replaced with CuBe, CuSn, and CuZn alloys. The frame rate and exposure time of the high-speed camera were set to 40 Hz and 25 ms, respectively. The acquisition frame rate of the infrared thermal imager was set to 80 Hz, and the exposure time was approximately 3.8 ms. The sampling frequency of the acoustic emission (AE) sensor was set to 2 MHz. Notably, the AS acquisition system was required to operate in full waveform acquisition mode to ensure full coverage of the entire test period. Signal segments obtained via threshold-based acquisition were deemed unsuitable, as this mode failed to capture the entire test duration and risked the loss of critical information. Although not central to the study, it is necessary to explain that the ASs for experiments **1-1-1** and **1-3-4** were acquired via threshold-based triggering rather than continuous waveform recording. As a result, the captured segments covered only approximately 10% of the total test duration, omitting substantial information and rendering the data incompatible with the alignment framework proposed in this study. Accordingly, these ASs are not included in Supplementary Table 3. To bypass the hardware-level synchronization of the in-situ monitoring module and accelerate validation of the alignment architecture, software-based synchronization triggering was employed to approximately synchronize the acquisition of the OS, IS, and AS.

Supplementary Table 3. **The dataset numbering and corresponding conditions of the orthogonal experiments**

Number	Monitor signal	Material	Load mode	Electric current / A	Rotational speed/ r/min	Time/ s
**1-1-1**	OS	CrZrCu alloy	CDL	2	50	1000
**1-1-2**	OS	CrZrCu alloy		2	150	
**1-1-3**	OS	CrZrCu alloy		8	50	
**1-1-4**	OS	CrZrCu alloy		8	150	
**1-2-1**	OS	CrZrCu alloy	CLL	2	50	
**1-2-2**	OS	CrZrCu alloy		2	150	
**1-2-3**	OS	CrZrCu alloy		8	50	
**1-2-4**	OS	CrZrCu alloy		8	150	
**1-3-1**	OS	CrZrCu alloy	CMLL	2	50	
**1-3-2**	OS	CrZrCu alloy		2	150	
**1-3-3**	OS	CrZrCu alloy		8	50	
**1-3-4**	OS	CrZrCu alloy		8	150	
**2-2**	OS,IS and AS	CrZrCu alloy	CLL	2、5、8	50、100、150	900
**2-3**	OS,IS and AS	CrZrCu alloy	CMLL			
**3-1**	OS and AS	CuBe alloy				
**3-2**	OS and AS	CuSn alloy				
**3-3**	OS and AS	CuZn alloy				

0-100s<br><br>2A<br>50r/min	100-200s<br><br>2A<br>100r/min	200-300s<br><br>2A<br>150r/min
500-600s<br><br>5A<br>50r/min	400-500s<br><br>5A<br>100r/min	300-400s<br><br>5A<br>150r/min
600-700s<br><br>8A<br>50r/min	700-800s<br><br>8A<br>100r/min	800-900s<br><br>8A<br>150r/min

Supplementary Figure 8. **The experimental conditions for datasets from 2-2 to 3-3.** Source data are provided as a Source Data file.

Moreover, the reference time shift (TS) range among the OS, IS, and AS via software-based synchronization triggering was estimated on the basis of additional photoacoustic thermal response tests (Supplementary Fig. 9), providing a reference for evaluating the alignment performance of the proposed architecture. Physical events that could be simultaneously detected by high-speed cameras, infrared thermal imagers, and AE sensors, such as the start and stop of friction disc rotation, were considered reference points for signal alignment. The fixture holding the disc-shaped specimen is visible in the IS image, as shown in Supplementary Fig. 9a. In the comparison between the upper left image and the lower left image, the disc-shaped specimens started to rotate at 37.4500 s. Similarly, the comparison between the upper right image and the lower right image indicates that the friction ceased at 933.2125 s. Therefore, according to the IS, the total duration of friction was 895.7625 s. In parallel, the AS can also be compared to distinguish the onset and cessation of disc rotation, allowing the friction duration to be identified, as shown in Supplementary Fig. 9b. The duration of friction determined from the AS was approximately 897.425 s, which was slightly longer than the 895.7625 s estimated from the IS. This discrepancy arises because the ASs were more sensitive to subtle variations and fluctuations in the friction load, whereas the low friction speed resulted in less pronounced variation between consecutive infrared frames. Four additional experiments were conducted on the basis of a more transient process to further support signal alignment. The experiments involved metal columnar projectiles impacting metal plates at a launch speed of approximately 58 m/s [3]. The impact produced clear acoustic, optical, and infrared responses, allowing for effective cross-modal alignment, as shown in Supplementary Fig. 9c. Via the impact experiments, reference ranges for the TS among the OA, AS, and IS in datesets **2-2** and **2-3** were determined. The TS t_i^a between the AS and IS ranged from -59.50×25 ms to -54.25×25 ms, whereas the TS t_o^a between the AS and OS ranged from -60.00×25 ms to -52.50×25 ms, which indicated that the TS between the signals consisted of a major systematic error and a minor random error. In addition to impact response testing, the photoacoustic transient response event of the arc generated by the arc igniter can also serve as a reference for signal alignment. The corresponding acoustic response is shown in Supplementary Fig. 9d, where the spectrogram exhibits a pronounced signal in the 17–19 Hz range. According to seven arc response tests, the TS t_o^a between OSs and ASs in datasets from **3-1** to **3-3** was determined to be within the range of -48.00×25 ms to -52.00×25 ms. Although each experiment could be approximately synchronized on the basis of other physical events such as impacts, microexplosions, or arc responses, the alignment parameters derived from these auxiliary events may not completely coincide with the semantic alignment parameters between signals of the target response.

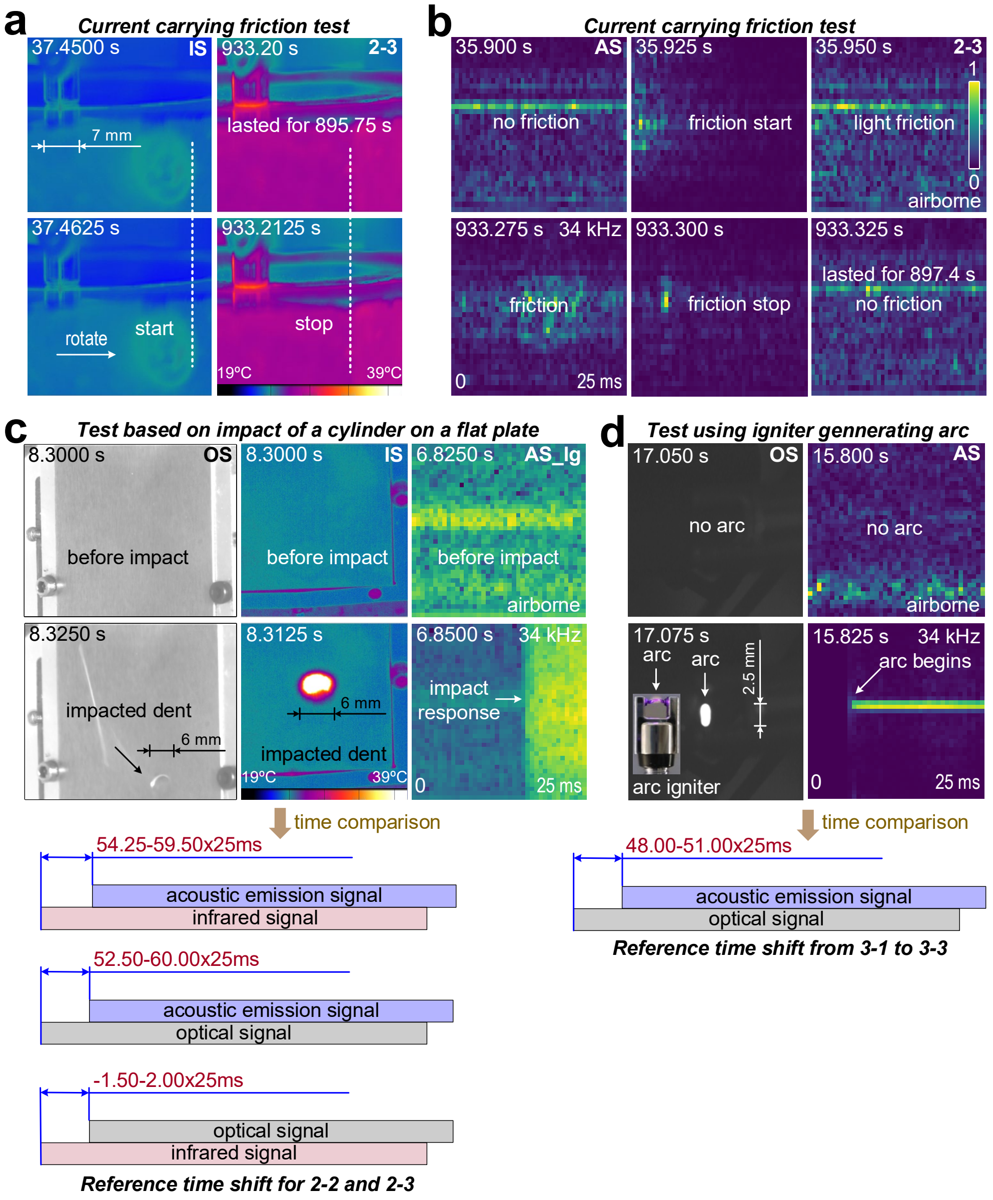

Supplementary Figure 9. **Reference time-shift range between the signals of the testing system. a** Discrimination criteria based on infrared signals (OSs) to determine whether the friction disc is rotating. **b** Spectrogram of the acoustic signals (AS) corresponding to the start and stop of the friction disk rotation. (The color bar indicates the normalized intensity, scaled to the [0, 1] range for each spectrogram individually.) **c** Approximate range of the time shift between OS, infrared signals (IS) and AS for datasets **2-2** and **2-3** of the experiments, obtained from the impact test. **d** Approximate range of the time shift of the system signal for datasets from **3-1** to **3-3** of the experiments, obtained from the arc igniter test. Source data are provided as a Source Data file.

With respect to in-situ signal processing, semantic labels were assigned to the OS from datasets from **2-2** to **3-3** via manual annotation, and to the ISs from dataset **2-3** via automated annotation. The labels were annotated by the same annotator, details of the labeling criteria are provided in Supplementary Table 4. The label "1" denotes the presence of an arc (positive sample), whereas the label "0" denotes its absence (negative sample). A total of 4832 sets of positive OS samples corresponding to arc damage were labeled from 36,000 optical signal sets in dataset **2-3**. Similarly, 2556 positive infrared signal samples were labeled from 72,000 infrared signal sets in the same datasets. The exposure time of the high-speed cameras was set to the maximum, whereas that of the IS imagers was 30.4% of the frame interval. Therefore, the lower number of positive samples in the IS data than in the OS data is reasonable. The features used for signal identification include spectrograms and statistical parameters such as the amplitude, peak value, and frequency. Although

recent studies on bearing fault diagnosis based on large language models have shown that spectrograms are superior to statistical parameters as features [4], certain statistical features contain precise physical meanings and can be analyzed to guide experiments and signal preprocessing decisions. The intensity and state of friction can be approximately inferred from the energy of the AS. The energy variations under different working conditions are compared, as shown in Supplementary Figs. 10a-c. As shown in Supplementary Fig. 10a, the energy increases with increasing rotational speed, exhibiting a stepwise trend across different speed levels. Supplementary Fig. 10b illustrates the trend in energy variation under different current conditions. As the current increases, the energy also increases without any step variation, and the energy distribution becomes dispersed. Supplementary Fig. 10c shows the signal energy under various normal loads, with more significant normal loads corresponding to higher energy. The experimental conditions described above suggest the presence of significant domain shifts in AS under varying friction conditions. Supplementary Fig. 10d presents the energy variation of background noise unrelated to the current-carried friction of the pin and disk specimens under different rotational speed conditions. As the system speed increased, the energy of the background noise exhibited a stepwise growth pattern. The energy of background noise fluctuates within the range of 1000-6000, whereas the energy associated with the current-carried friction ranges from 25000-36000. The background noise contributes no more than 25% of the total AS energy, indicating minimal interference with the frictional acoustic response. Accordingly, no additional denoising was applied to the acquired ASs. Each signal was segmented into 25-ms intervals and transformed into spectrograms via the short-time Fourier transform, followed by normalization. To further demonstrate the versatility of the proposed architecture, additional spectrograms were generated via lg transformation and normalization for subsequent analysis. As shown in Supplementary Fig. 11, spectrograms without lg transformation exhibit more pronounced high-intensity features. In contrast, applying a lg transformation suppresses these dominant responses and enhances the visibility of lower-intensity components.

Supplementary Table 4. **Label information of optical and infrared signals**

Experiment number	Signal	Number	Labeling method	Arc response frequency
**2-2**	OS	36000	Manual annotation	256
**2-2**	IS	72000	Automatic annotation<br>(Based on green unrecognized areas)	183
**2-3**	OS	36000	Manual annotation	4821 or 4713<br>(The same annotator annotates twice, inconsistency rate: 97.84%)
**2-3**	IS	72000	Automatic annotation<br>(Based on green unrecognized areas)	2556
**3-1**	OS	36000	Manual annotation	5768
**3-2**				7652
**3-3**				1944(Inclined friction)

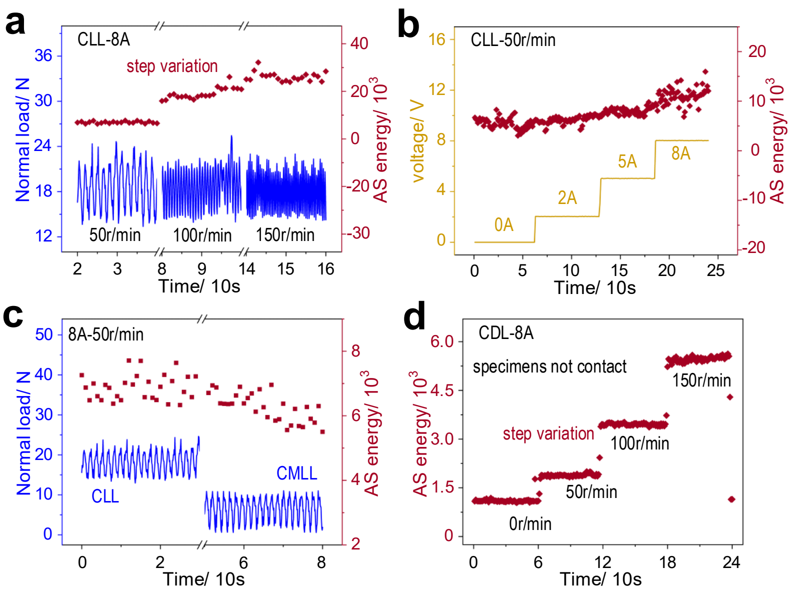

Supplementary Figure 10. **Analysis of the energy of the acoustic signals (AS). a-b** Energy variation of the AS at different speeds and currents under constant load loading (CLL) mode. **c** Energy of the AS under CLL and constant minor load loading (CMLL) modes. **d** The energy of the AS in the background of the system corresponding to different rotational speeds under constant displacement loading (CDL) mode. Source data are provided as a Source Data file.

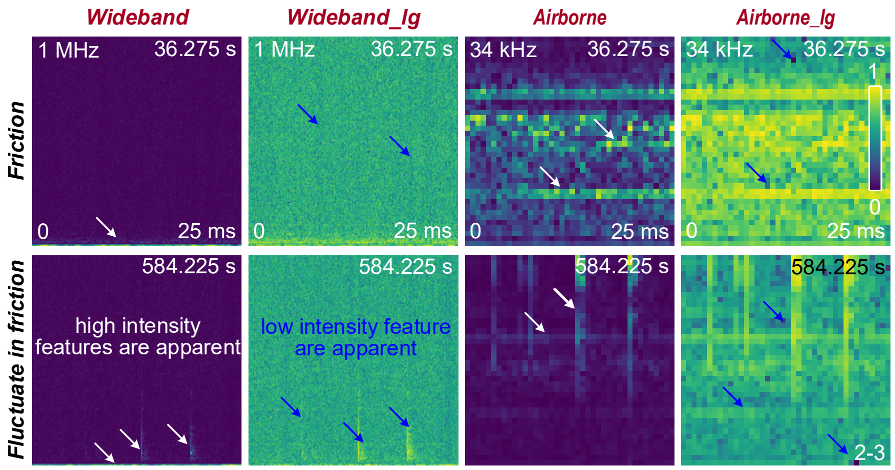

Supplementary Figure 11. **Comparison of the acoustic signal spectrograms with and without lg function transformation.** (The color bar indicates the normalized intensity, scaled to the [0, 1] range for each spectrogram individually.) Source data are provided as a Source Data file.

Supplementary Table 5. **Training parameters of the kernel models**

Illustration	Task<br>(number of repetitions)	Dataset(Proportion of positive and negative samples)	Model	Epoch	Learning rate	Batch
Fig. 2a	Alignment of label&IS and AS<br>(1)	**2-3**:3600 samples(1:1)				
Figs. 2c-f<br>Supplementary Fig. 13	Alignment of label&OS and AS<br>(1)	**2-3**:9000 samples(1:1)		100	2×10^{-4}	
Supplementary Figs. 14a-f<br>Supplementary Figs. 19	Alignment of label&OS and AS<br>(3)					
Figs. 2g, 2i	Alignment of label&OS and AS(transfer training final layer)<br>(1)			1000	8×10^{-2}	
Supplementary Figs. 11h-j	Alignment of label&OS and AS(transfer training final layer)<br>(3)	**2-2**:500 samples(1:1)	RDNet41			512
Figs. 2g, 2 h	Alignment of label&OS and AS<br>(training from start)<br>(1)			100	2×10^{-4}	
Fig. 2g	Alignment of label&OS and AS<br>(globally transfer training)<br>(1)			500	1×10^{-6}	
Supplementary Figs. 15a-e	Alignment of label&OS and AS<br>(3)	**3-1**:10000 samples(1:1)				
Supplementary Figs. 15f-j		**3-2**:14000 samples(1:1)			2×10^{-4}	
Supplementary Figs. 16c-h		**3-3**:3800 samples(1:1)				
Supplementary Fig. 20d	Arc detection based on OS<br>(1)	**2-2**:508 samples(1:1)+<br>**2-3**:9626 samples(1:1)	RSNet1		1×10^{-6}	128
Supplementary Fig. 20e					1×10^{-5}	
Supplementary Fig. 20f						256
Figs. 3a-c		**2-3**:9000 samples(1:1)	RRNet18<br>RRNet10<br>RMNet<br>MNet4<br>MNet3<br>RDNet41<br>RDNet13<br>MNet2_64<br>MNet2_32<br>MNet2_16<br>MNet2_8<br>MNet2_4<br>MNet2_2<br>MNet2_1	100	2×10^{-4}	
Figs. 3d-f<br>Fig. 4a	Alignment of label&OS and AS<br>(3)		RDNet13		2×10^{-4}<br>4×10^{-4}<br>2×10^{-3}	512
Supplementary Fig. 24		**2-3**:50-9000 samples(1:1)	MNet2_1		2×10^{-4}<br>2×10^{-2}<br>1×10^{-1}<br>5×10^{-1}	
Figs. 3g-i		**2-3**:5000 samples(1:8 - 8:1)	RDNet41		2×10^{-4}	
Supplementary Fig. 26<br>Fig. 4a		**2-3**:50-9000 samples(1:1)	/	/	/	/
Figs. 4b-c<br>Supplementary Fig. 28	Alignment of label&AS and AS<br>(3)	Generated data: 9000 samples(1:1)	GRU	200	1×10^{-2}	512
Supplementary Figs. 30a-c	Alignment of label&IS and IS<br>(3)	**2-3**: 5000 samples(1:1)			2×10^{-3}	
Supplementary Figs. 30d-f	Alignment of label&OS and OS<br>(3)		RSNet1	100	1×10^{-5}	128
Supplementary Figs. 30g-i	Alignment of label&OS and OS feature<br>(3)	**2-3**: 9000 samples(1:1)	MLP		1×10^{-3}	1024
Figs. 4d-f<br>Supplementary Figs. 30j-l	Alignment of label&function output and function input<br>(3)	Generated data: 500(1:1)		/	/	/
Supplementary Fig. 31	Alignment of function output and function input<br>(3)	Generated data: 500	SVM	/	/	/
Figs. 4g-I<br>Supplementary Fig. 29	Alignment of feature vector&OS and OS<br>(3)	**2-3**: 9000 samples(1:1)	RANet	50	1×10^{-4}	128

Supplementary Table 6. **The information of the revised neural networks**

Revised networks	Original network	Revised ranges	Input size	Input size
GRU [5] (Supplementary Table 7)	/	/	164×164	2
MLP [6] (Supplementary Table 8)	/	/	8	2
MNet (Supplementary Tables 9-11)	/	Self developed network	64×64	2
RANet	AlexNet [7]	The last few layers	224×224	8
RDNet13 (Supplementary Table 12)	DenseNet121 [8]	The first few layers, the last layer and the number of dense block	64×64	2
RDNet41 (Supplementary Table 13)	DenseNet121 [8]	The first few layers, the last layer and the number of dense block	64×64	2
RMNet	MobileNet_v3_small [9]	The first layer and the last layer	112×112	2
RRNet10	ResNet10 [10]	The first few layers and the last layer	56×56	2
RRNet18	ResNet18 [11]	The first few layers and the last layer	56×56	2
RSNet1	SqueezeNet1_1 [12]	The first layer and the last layer or the last layer	224×224	2
SVM [13] (ML model)	/	/	2	1

Supplementary Table 7. **The structure of the GRU**

Layers	Structure
GRU Layer	GRU(164,164,1)
Layer	Linear(164,2), softmax

Supplementary Table 8. **The structure of the MLP**

Layers	Structure
Layer1	Linear(8,16), ReLU
Layer2	Linear(16,8), ReLU
Layer3	Linear(8,2), softmax

Supplementary Table 9. **The structure of MNet2_*x***

Layers	Structure
Convolution_1	Conv2d(3, x,3×3,2,1), BatchNorm2d, ReLU
Classification	global avg pool2d
Layer	Linear(x,2), softmax

Supplementary Table 10. **The structure of MNet3**

Layers	Structure
Convolution_1	Conv2d(3,64,3×3,2,1),BatchNorm2d,ReLU
Convolution_2	Conv2d(64,128,3×3,2,1), BatchNorm2d,ReLU
Classification	global avg pool2d
Layer	Linear(128,2), softmax

Supplementary Table 11. **The structure of MNet4**

Layers	Structure
Convolution_1	Conv2d(3,64,3×3,2,1),BatchNorm2d,ReLU
Convolution_2	Conv2d(64,128,3×3,2,1), BatchNorm2d,ReLU
Convolution_3	Conv2d(128,256,3×3,2,1), BatchNorm2d,ReLU
Classification	global avg pool2d
Layer	Linear(128,2), softmax

Supplementary Table 12. **The structure of RDNet13**

Layers	Output Size	Structure
Convolution	66×66	1×1conv, stride 1
Dense Block_1	66×66	$\begin{bmatrix} 1 \times 1 \text{ conv} \\ 3 \times 3 \text{ conv} \end{bmatrix} \times 1$
	66×66	
Transition Layer_1	66×66	1×1 conv
	33×33	2×2average pool, stride 2
Dense Block_2	33×33	$\begin{bmatrix} 1 \times 1 \text{ conv} \\ 3 \times 3 \text{ conv} \end{bmatrix} \times 1$
Transition Layer_2	33×33	1×1 conv
	16×16	2×2average pool, stride 2
Dense Block_3	16×16	$\begin{bmatrix} 1 \times 1 \text{ conv} \\ 3 \times 3 \text{ conv} \end{bmatrix} \times 1$
Transition Layer_3	16×16	1×1 conv
	8×8	2×2average pool, stride 2
Dense Block_4	8×8	$\begin{bmatrix} 1 \times 1 \text{ conv} \\ 3 \times 3 \text{ conv} \end{bmatrix} \times 1$
Classification	1×1	8×8 adaptive avg pool2d
Layer	2	2D fully connected, softmax

Supplementary Table 13. **The structure of RDNet41**

Layers	Output Size	Structure
Convolution	66×66	1×1conv, stride 1
Dense Block_1	66×66	$\begin{bmatrix} 1 \times 1 \text{ conv} \\ 3 \times 3 \text{ conv} \end{bmatrix} \times 2$
	66×66	
Transition Layer_1	66×66	1×1 conv
	33×33	2×2average pool, stride 2
Dense Block_2	33×33	$\begin{bmatrix} 1 \times 1 \text{ conv} \\ 3 \times 3 \text{ conv} \end{bmatrix} \times 4$
Transition Layer_2	33×33	1×1 conv
	16×16	2×2average pool, stride 2
Dense Block_3	16×16	$\begin{bmatrix} 1 \times 1 \text{ conv} \\ 3 \times 3 \text{ conv} \end{bmatrix} \times 8$
Transition Layer_3	16×16	1×1 conv
	8×8	2×2average pool, stride 2
Dense Block_4	8×8	$\begin{bmatrix} 1 \times 1 \text{ conv} \\ 3 \times 3 \text{ conv} \end{bmatrix} \times 4$
Classification	1×1	8×8 adaptive avg pool2d
Layer	2	2D fully connected, softmax

Supplementary Discussion 3. **Feasibility of arc detection via infrared signals**

The effectiveness of the infrared signal (IS) in calculating the frequency of arc damage in current-carried friction is subsequently explored. The labels of dataset **2-2** (176 positive samples (40 Hz) from the IS and 256 positive samples from the optical signal) and **2-3** (2031 positive samples (40 Hz) from the IS and 4821 positive samples from the optical signal) were merged. The merged labels were cut with a duration of 10 s (10×40×25 ms) and a 5×25 ms step size to calculate the frequency of positive samples (corresponding to label 1 and arc damage) obtained from the optical signal (OS) and IS every 10 seconds. A total of 14325 data points were obtained, as shown in Fig. 2b. The points in the lower right and upper left corners of Fig. 2b indicate that there were blind regions in the fields of view captured by the high-speed cameras and infrared thermal imagers. There were more points in the lower right corner than in the upper left corner, indicating that the arc recognition performance based on the IS was inferior to that based on the OS. The arc response frequency derived from the IS was positively correlated with that from the OS. Additionally, many points in Fig. 2b may overlap. The residuals between the fitted curve and the points were calculated, as shown in Supplementary Fig. 12a. Most residuals fell within ±20, indicating that the IS was effective in identifying arcs to some extent. Furthermore, the number of pixels in the unrecognized area of the infrared image (IS) can be utilized to characterize the arc intensity, as shown in Supplementary Fig. 12b.

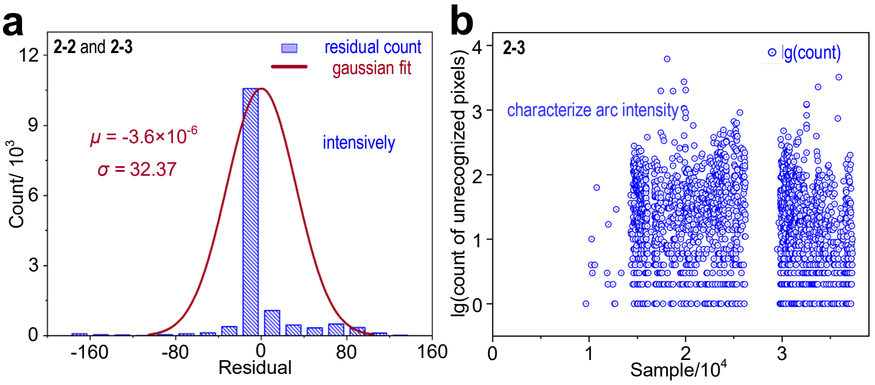

Supplementary Figure 12. **The effectiveness of infrared signals in arc monitoring and detection. a** Distribution of the residuals of the fitted curve in Fig. 2b. **b** Statistics of the number of unrecognized pixels in the infrared signals. Source data are provided as a Source Data file.

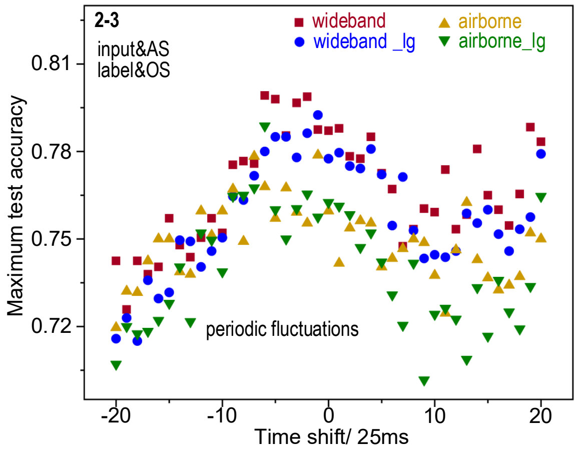

Supplementary Figure 13. **Typical time shift–maximum testing accuracy curve of alignment between optical signals (OS) and acoustic signals (AS) in 2-3 sets, away from the semantic time shift.** Source data are provided as a Source Data file.

Supplementary Discussion 4. **Repetitive and supplementary alignment**

To further assess the reliability of the alignment results produced by the proposed architecture, the alignment procedure was repeated, and the distributions of training and testing accuracy during kernel model training were analyzed. In the coarse alignment stage (step: 25 ms) of dataset **2-3** (Supplementary Fig. 14a), the alignment results from three independent replicates (data is not shown separately) were consistent with those shown in Fig. 2c. The relationship between the time shift (TS) and the mean of the maximum training and testing accuracies across the three alignment trials is shown in Supplementary Fig. 14a, which is consistent with the alignment results reported in Fig. 2c. The mean values of the maximum training and testing accuracies derived from multiple experiments provide a more reliable estimate than those from a single experiment. This is analogous to the improved accuracy observed in ensemble learning, where decision-making from multiple learners with equal weights outperforms that of a single learner. As shown in Supplementary Fig. 14b, the comprehensive distribution of testing accuracy across the three experiments follows the overall trend of mean maximum testing accuracy with TS, with testing accuracy consistently higher for semantic TSs than for other time shifts. The corresponding training accuracy distribution is shown in Supplementary Fig. 14c. Similarly, training accuracy under semantic TSs is consistently greater than that under other TS conditions. This can be attributed to the weaker correspondence between input signals and semantic labels in nonsemantic alignments, which reduces the proportion of functionally relevant components in the loss function that effectively drive model training, thereby slowing the training rate. Furthermore, although training accuracy generally remains higher than testing accuracy across all conditions, it exhibits less variability in response to TSs than testing accuracy does, which suggests that the kernel model perceives semantic alignment parameters more effectively in the testing set than in the training set. The pre-AlexNet era was defined by traditional machine learning models, such as support vector machines (SVMs). In contrast, neural networks that emerged following the AlexNet breakthrough are typically characterized by increased channel widths, which foster the development of diverse random features. This architectural approach expands the solution space and enhances representational power, enabling these networks to outperform earlier machine learning methods. While neural networks may exhibit better predictive variance than more industrially robust methods such as SVMs do, this variability can be constructively leveraged. When kernel models are trained across multiple epochs and under repetitive alignment conditions, the aggregated predictions form a stable, ensemble-like distribution at each TS. This concept parallels the idea that integrating observations from multiple perspectives leads to a more accurate interpretation. Thus, for semantic TSs, evaluations based on the full distribution of testing accuracy provide a more reliable metric than those relying solely on the maximum testing accuracy. The distribution provides a more comprehensive view of the training process, whereas the maximum testing accuracy typically reflects the credibility of the alignment results, although it is less robust. The comprehensive results of three repeated fine alignments (step size: 5 ms) for dataset **2–3** are shown in Supplementary Figs. 14d–f. Similarly, the results of three repeated coarse alignments (step size: 5 ms) for the **2–2** experiments are presented in Supplementary Figs. 14g–j. These comprehensive alignment results are consistent with previous findings. Supplementary Fig. 14i shows the TS–maximum testing accuracy curves for the three repeated alignments of the dataset **2–2** . The trends of the three curves at the semantic TS are consistent, but there are inconsistencies at other TS positions. In some cases, these inconsistencies may propagate to the semantic shift and affect the alignment outcome. Therefore, for subsequent alignments, the alignment experiment was repeated three times. To further demonstrate the effectiveness of the proposed architecture, alignment between the optical signal (OS) and acoustic signal (AS) from experiments with different current-carried friction pairs **(3–1** and **3–2**) and friction modes (**3–3**) was conducted. The friction pairs in dataset **3–1** are 7075 Al alloy and CuBe alloy, with the alignment results shown in Supplementary Figs. 15a–e. The semantic TS t_o^a for this dataset is -51.4×25 ms. In the dataset **3–2** , the friction pairs are 7075 Al alloy and CuSn alloy, with the alignment results presented in Supplementary Figs. 15f–j, and the semantic TS t_o^a is -53.0×25 ms.The alignment results of OS and AS for dataset **3-3** are shown in Supplementary Figs. 16c–h, with an alignment parameter t_o^a of -49.2×25 ms. For datasets from **3-1** to **3-3**, the obtained alignment parameter is close to the reference range from -51.00×25 ms to -49.00×25 ms derived from the photoacoustic response test. Notably, in addition to the alignment of dataset **3-3** (inclined friction in Supplementary Fig. 16a), the comparison of the alignment results from datasets **2-3**, **3-1**, and **3-2** in the four vertical friction tests (Supplementary Fig. 16b) with the corresponding reference range of TSs suggests that the semantics of the AS tend to lag behind those of the synchronous OS. Therefore, the acoustic response following the arc response may serve as a basis for detecting the arc response via the AS.

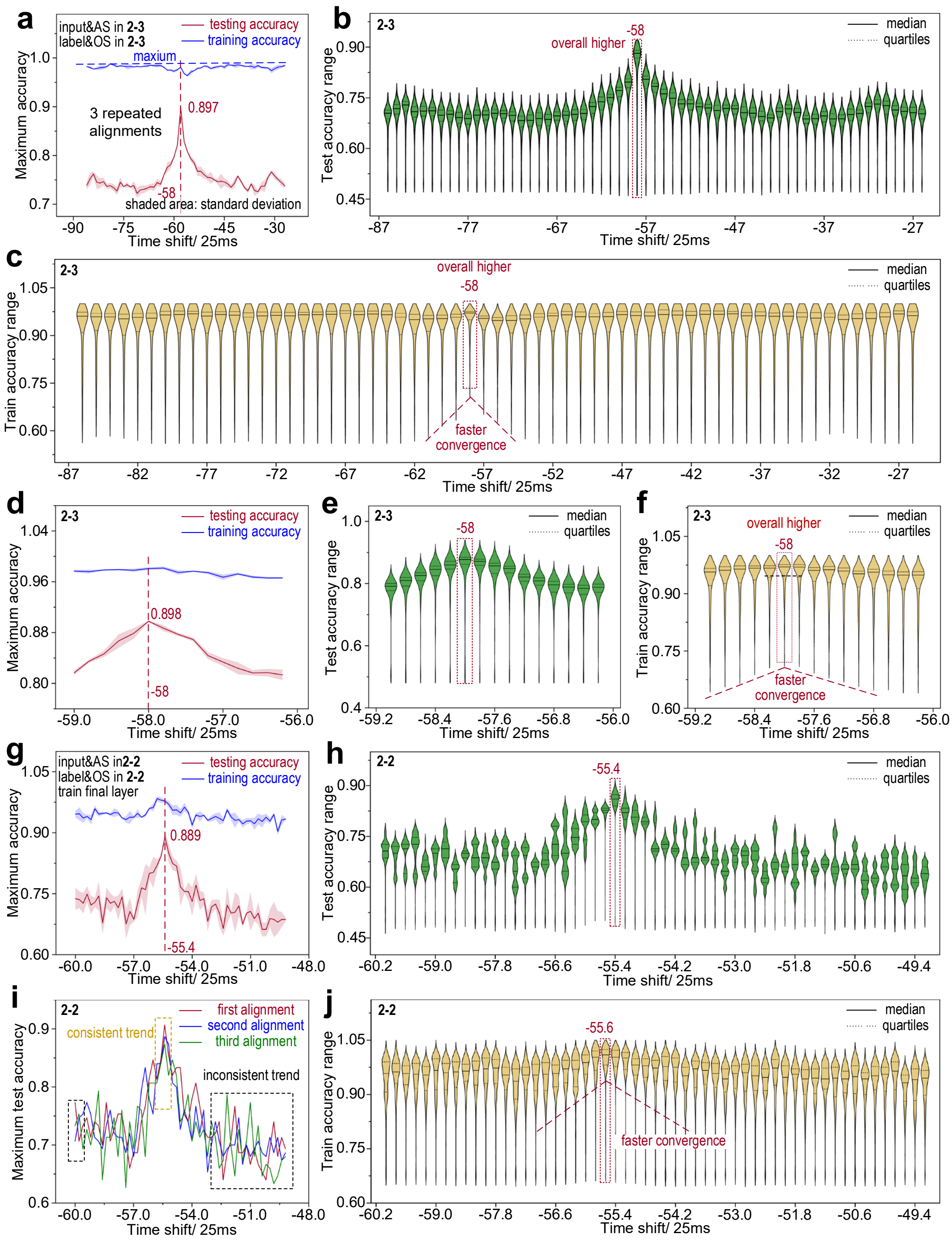

Supplementary Figure 14. **Shift–maximum accuracy curves and accuracy distributions for aligning optical signals (OS) and acoustic signals (AS) in datasets 2-2 and 2-3.** a-**b** Time shift(step size: 25 ms)-maximum training and testing accuracy curves and testing accuracy distributions corresponding to 3 alignments of **2-3** sets of OS and AS, respectively. **c** Time shift (step size: 25 ms)-train accuracy distributions in dataset **2-3**. **d** Time shift (step size: 5 ms)-maximum accuracy curves in dataset **2-3**. **e-f** Time shift (step: 5 ms)-testing accuracy distributions and training accuracy distributions, respectively, in dataset **2-3**. **g** Time shift (step: 5 ms)-maximum accuracy curves in dataset **2-3**. **h, j** Time shift (step: 5 ms)-testing accuracy distributions and training accuracy distributions, respectively, in dataset **2-2**. **i** Time shift (step: 5 ms)-maximum testing accuracy curve corresponding to the alignment repeated 3 times, in dataset **2-2**. Source data are provided as a Source Data file.

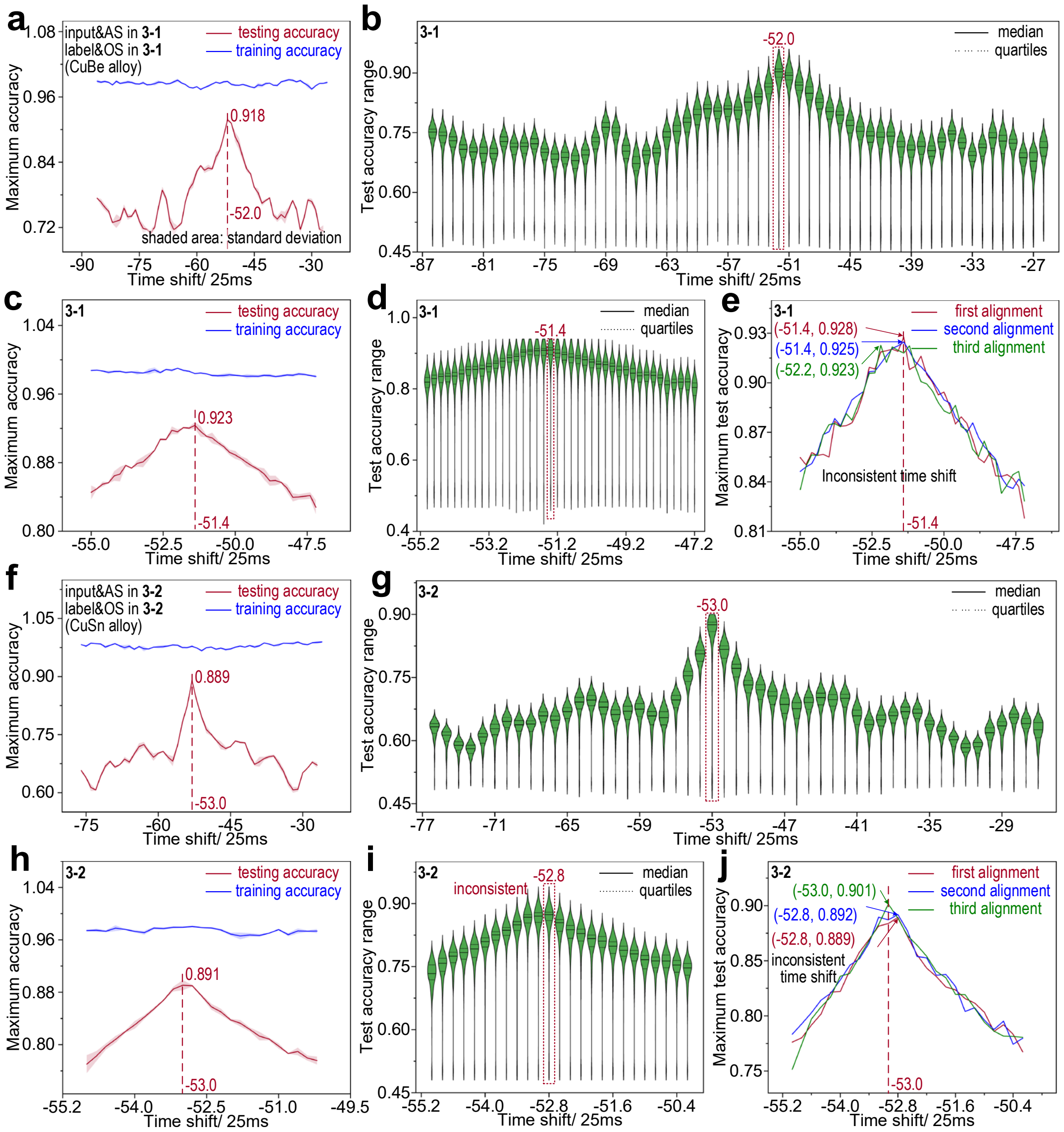

Supplementary Figure 15. **The time shift-maximum accuracy curve and accuracy distribution corresponding to the alignment of optical signals (OS) and acoustic signals (AS) in datasets 3-1 and 3-2. a-b** The time shift (step size: 25 ms)-maximum accuracy curve and time shift (step size: 25 ms)-testing accuracy distribution for dataset **3-1** alignment, respectively. **c-d** Time shift (step size: 5 ms)-maximum accuracy curve and time shift (step size: 5 ms)-testing accuracy distributions in the dataset **3-1** alignment, respectively. **e** Time shift–maximum testing accuracy curve corresponding to 3 repeated alignments in dataset **3-1**. **f-g** Time shift (step size: 25 ms)-maximum accuracy curve and time shift (step size: 25 ms)-testing accuracy distributions for dataset **3-2** alignments. **h-i** Time shift (step size: 5 ms)-maximum accuracy curve and time shift (step size: 5 ms)-testing accuracy distributions in the dataset **3-2** alignment, respectively. **j** Time shift–maximum testing accuracy curve corresponding to 3 repeated alignments in dataset **3-2**. Source data are provided as a Source Data file.

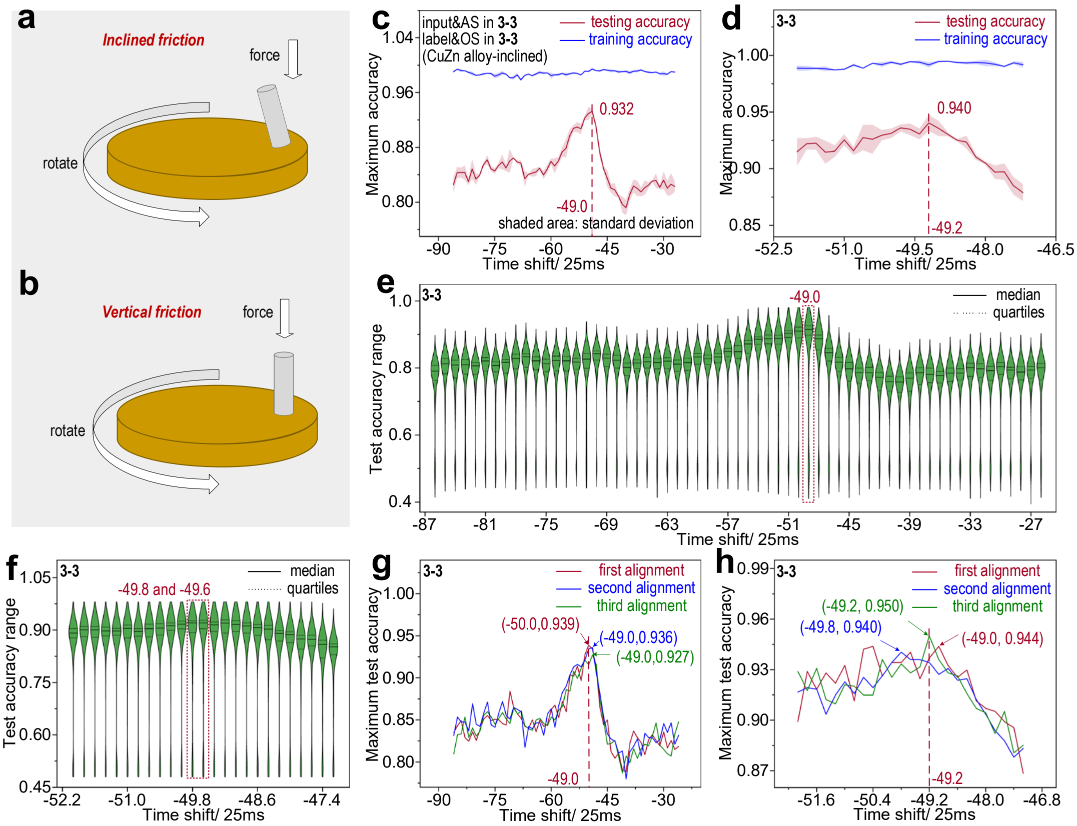

Supplementary Figure 16. **Friction form of the test specimens and alignment results of the optical signals (OS) and acoustic signals (AS) in dataset 3-3 of the experiments. a** Schematic diagram of inclined friction in dataset **3-3**. **b** Schematic diagram of vertical friction in other datasets. **c-d** Time shift (time step: 25 or 5 ms)-maximum accuracy curve for **3-3** sets of alignments. **e-f** Time shift (step: 25 or 5 ms)-testing accuracy distributions for **3-3** sets of alignments. **g-h** Time shift (step: 25 or 5 ms)-maximum testing accuracy curve corresponding to 3 repeated alignments in dataset **3-3**. Source data are provided as a Source Data file.

Supplementary Discussion 5. **Interpretability analysis of the acoustic signals**

To analyze the principle of arc detection via acoustic signals (ASs), category activation mapping (CAM) [14] was performed from the last convolutional layer of the kernel model corresponding to the 53rd epoch in Fig. 2e, as shown in Supplementary Figs. 17a-b. The CAM is related to both the activation value and gradient, and the regions with significant CAM values indicate that the regions of the input image significantly affect the class discrimination of the model. In regions with relatively significant CAM values, those corresponding to no arc damage were more extensive than those corresponding to arc damage. Therefore, the arc damage may be closely related to the local features of the spectrogram, whereas the absence of arc damage is more closely associated with the global features. The distribution of significant regions in the arc damage was related to the longitudinal texture of the spectrogram, although not entirely. In contrast, the significant areas corresponding to arc damage and no arc damage did not appear to be strongly related to frequency, unlike the clear correspondence between the arc response of the arc igniter and its corresponding spectrogram in Supplementary Fig. 9d. The arc damage in current carrying friction seems to be related to the spectrogram response in the range of 8-34 kHz, whereas the corresponding spectrogram without arc damage shows a relatively strong response in the range of 8-22 kHz. More CAM cloud maps are provided in Supplementary Movie 4 and Supplementary Movie 5. Owing to the inherent limitations of human perception, memory, and information processing, the brain, guided by evolutionary attention mechanisms, is best suited for recognizing high-contrast features, such as vertical stripes in spectrograms. Discriminating arc damage depends on coupling relationships in the AS spectrogram, which are difficult for the brain to identify. However, these complex and subtle patterns can be effectively recognized and accurately characterized by neural networks with strong expressive power. Although CAM does not provide researchers with particularly explicit domain knowledge, the proposed alignment architecture helps separate AS corresponding to arc damage from those without arc damage, which is challenging to accomplish solely through the brain. Supplementary Fig. 17c shows the normalized spectrogram corresponding to the AS in Supplementary Fig. 18, which corresponds to arc damage and slight friction(current: 8A). Supplementary Fig. 18 illustrates the energy variation when the power is switched on and off under the constant displacement loading mode. The micrometer head was rotated to slightly separate the current-carried friction pair. Initially, the power was switched off (0 A), then switched on (8 A), and finally, the power was turned off again (0 A). The variations in the voltage signal in the figure reflect this process. When voltage was applied to the friction pair, an increase in voltage fluctuation indicated arc damage. In contrast, an increase in the normal load was associated with the formation of minor surface protrusions, likely originating from transferred material, which contributed to elevated friction. Supplementary Fig. 17c presents representative spectrograms recorded under a current load of 8 A, including both time-stable patterns and those exhibiting pronounced temporal fluctuations, The relatively fluctuating spectrogram in Supplementary Fig. 17c exhibits a lower fluctuation frequency along the time axis than that in Supplementary Fig. 17a does, which may correspond to a higher frequency of collisions and frictional interactions between small surface protrusions under the constant minor load loading mode than under the slight friction condition (current: 8A) corresponding to Supplementary Fig. 18. Accordingly, the presence of vertical textures in the spectrograms corresponds to arc-induced damage on the surface of the friction pair. In contrast, the relatively temporal stable spectrograms observed in Supplementary Figs. 17b and 17c exhibit similar features, which may be indicative of friction-related damage. Moreover, in the arc generation, repeated collision and solidification of metals result in the tiny protrusions between microscopic protrusions on the friction pair surfaces, inducing unstable friction and significant fluctuations in the AS spectrogram (Supplementary Fig. 17a). In contrast, under situations without arc damage, tiny protrusions have been peeled off or flattened during friction, resulting in fine frictional contact, which effectively suppresses system fluctuations and results in a more stable AS spectrogram (Supplementary Fig.17b) along the time axis. Moreover, in this case, the delay between the OS and AS, caused by the inherent difference in the propagation speeds of light and sound, is approximately 0.5 ms, which has only a minor influence on the overall system. Consequently, the arc detection model with AS as input performs inference on the basis of the AS recorded after the arc event has occurred. This observation is consistent with the earlier finding that the semantics of AS tend to lag behind those of synchronous OS in the alignment process mentioned in Supplementary Discussion 4, further indicating the reliability of the above statement and the significance of the proposed signal alignment architecture. As shown in the confusion matrix (Fig. 2f) of the arc detection model based on the AS input, the false positive (pred 1, label 0) and false negative (pred 0, label 1) rates are relatively comparable. Since the labels for training the model are derived from the aligned OS, the false negative rate may result from arc events that cause sufficiently strong acoustic

responses detectable by the acoustic emission sensor, but are missed by the high-speed camera owing to its visual blind spots. In contrast, the false positive rate may be attributed to arc events with weak or ambiguous acoustic signatures that are captured by the high-speed camera but fail to elicit a detectable response in the acoustic emission sensor.

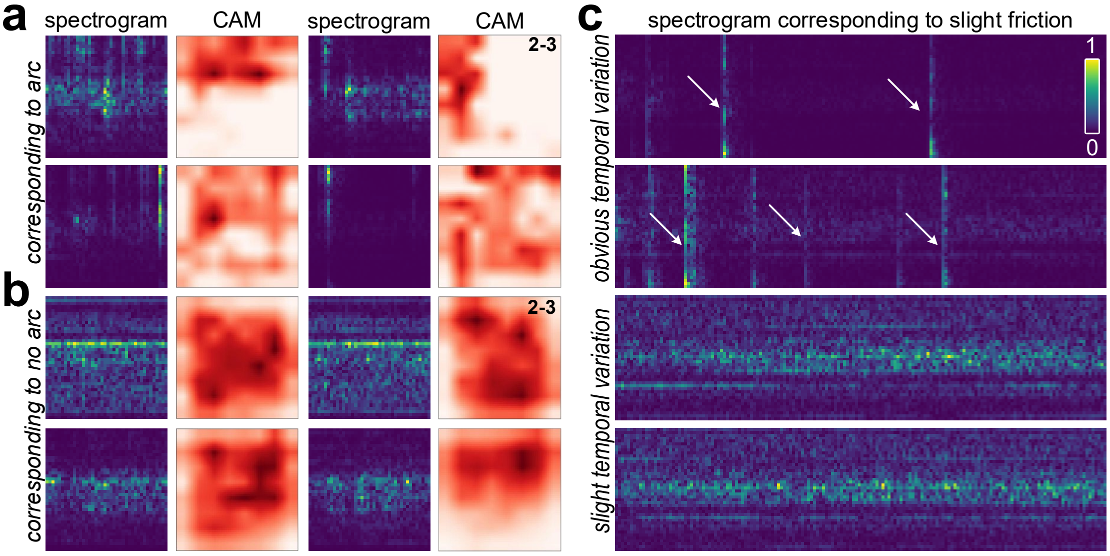

Supplementary Figure 17. **Interpretability analysis of typical acoustic signals. a-b** Normalized spectrograms of the acoustic signals selected via the signal alignment method and the calculated class activation maps (CAMs) under conditions of arc damage and no arc damage. **c** Typical normalized spectrogram of the acoustic signals under the condition of slight contact (current: 8 A) between two friction specimens (Supplementary Fig. 16.). (a, b and c show multiple sets of spectrograms with the same semantics to more fully demonstrate the common features of the signal. The color bar indicates the normalized intensity, scaled to the [0, 1] range for each spectrogram individually.) Source data are provided as a Source Data file.

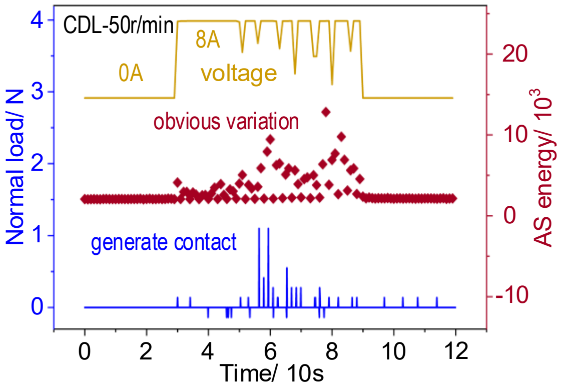

Supplementary Figure 18. **The variations in the acoustic signal (AS) energy and normal load with varying electrification conditions, occurring in the case of slight separation of the friction pair under the constant displacement loading (CDL) mode.** Source data are provided as a Source Data file.

Supplementary Discussion 6. **The impact of the spectrogram frequency range on alignment**

Additionally, during the implementation of the alignment architecture, minimizing information loss to ensure accurate signal representation is essential. The airborne acoustic emission sensor collected acoustic signals at a sampling frequency of 2 MHz. Although the measurement range of the sensor is 1–34 kHz, the highest effective frequency of its spectrogram is 1 MHz according to the Nyquist theorem. Therefore, a spectrogram of the frequency range of 0–1 MHz was generated to align the optical signals and acoustic signals, and the results were compared to those based on spectrogram of the frequency range of 0–34 kHz, as shown in Supplementary Fig. 19. As illustrated in Supplementary Figs. 19a–c, both approaches yield comparable alignment accuracies. Supplementary Fig. 19d presents the maximum testing accuracies for the kernel model achieved at the aligned time shift for both the 34 kHz and 1 MHz spectrograms. The 1 MHz spectrogram yields an accuracy of 91.7%, which exceeds the 89.8% obtained on the basis of the 34 kHz spectrogram. Therefore, effective information is included in spectrograms of 34 kHz-1 MHz. Effective information typically leads to improved alignment accuracy, as higher tesing accuracy of the kernel model under aligned time shifts may reflect more reliable alignment. Additionally, for the processing of one-dimensional signals used in alignment architectures, on the basis of ensuring that the sampling frequency of the signal is at least 2.5 times the highest frequency of the spectrogram, it is necessary to explore whether there is effective information in a wider frequency band, which may be beneficial for alignment.

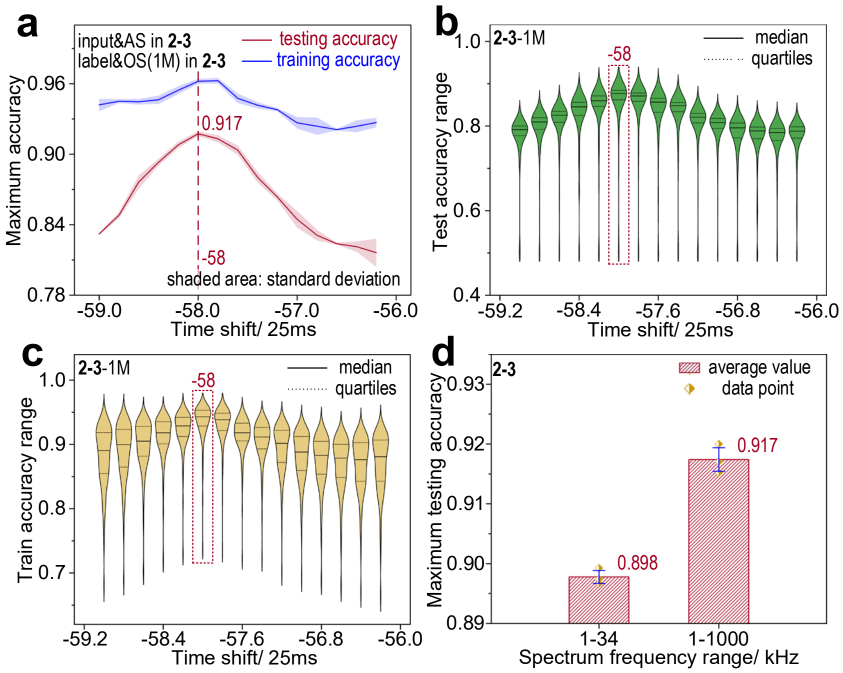

Supplementary Figure 19. **Influence of the frequency range of the spectrogram from the acoustic signal (AS) collected by the airborne acoustic emission sensor on the alignment. a** Time shift (time step: 5 ms)-highest accuracy curve of alignment between the AS spectrogram in the 1 MHz frequency range and the optical signal (OS). **b-c** Time shift (time step: 5 ms)-testing accuracy distribution and time shift (time step: 5 ms)-training accuracy distribution, respectively, under the same conditions. **d** Influence of the frequency range of the spectrogram on the maximum testing accuracy of the kernel model for the aligned parameters. Supplementary Figure 19 corresponds to the results of three repeated experiments. Source data are provided as a Source Data file.

Supplementary Discussion 7. **Feasibility of arc detection via optical signals**

The effectiveness of arc detection via infrared signals or acoustic signals was previously evaluated via optical signals (OSs) as a reference. To further verify the reliability of the OS in arc detection, arc detection models were established with OSs as inputs. The dataset for these models consisted of OS data from datasets from **2-2** to **3-3**, along with manually annotated labels derived from the OS data (Supplementary Table 4). Details of the models and their corresponding training parameters are provided in Supplementary Table 5. Supplementary Fig. 20 shows the preprocessing results for the OS data and the performance of the corresponding arc detection models. Since OS images contain both infrared and visible spectral spectrum components, variations in light intensity and background temperature may lead to domain drift. Therefore, it is essential to explore preprocessing methods to mitigate domain shifts in OS images. Supplementary Figs. 20a-c show the original OS images from datasets **2-2** and **2-3**, the images processed via histogram equalization, and those processed via dynamic threshold adaptive methods. Supplementary Figs. 20d–20f show the epoch-loss and accuracy curves, along with the corresponding confusion matrices, for arc detection models trained on original images, images preprocessed via histogram equalization, and images preprocessed via dynamic threshold adaptive methods, respectively. Among the three models, the model trained on dynamically thresholded images demonstrates the most stable training process and achieves the highest testing accuracy, reaching 97.2%, outperforming the other two preprocessing approaches. Supplementary Fig. 20g compares OS images from datasets from **3-1** to **3-3** and **1-1-1**, highlighting domain shifts caused by differences in background lighting and temperature. To evaluate the effectiveness of the three preprocessing methods in mitigating domain shift, the models that achieved the maximum testing accuracy on datasets **2-2** and **2-3** (Supplementary Fig. 20d: epoch 100, Supplementary Fig. 20e: epoch 62, Supplementary Fig. 20f: epoch 89) were tested on datasets from **3-1** to **3-3**. The test results, shown in Supplementary Fig. 20h, indicate that the model trained on histogram-equalized images exhibits the weakest generalization performance. In contrast, the models trained on raw images and dynamically thresholded images demonstrate comparable generalization capabilities. Although the model trained on raw images performed well in this case, its corresponding generalization performance may be less reliable when it is applied to optical images with more significant domain shifts. The arc temperature (3,000–30,000 °C), which is sufficient to melt metal, significantly differs from the background environment. For OS data collected in a darkroom, the dynamic threshold adaptive preprocessing method effectively preserves key features at the interface between the arc and the background. Moreover, the resulting binary images, composed solely of black and white pixels, help to substantially mitigate the domain shift. Accordingly, dynamic threshold adaptive preprocessing is recommended to increase the robustness of arc detection under varying imaging conditions. The arc detection model trained on dynamically thresholded images was subsequently applied to determine whether the OS in datasets from **1-1-1** to **1-3-4** corresponded to arc damage. The test results are shown in Supplementary Fig. 21. Supplementary Figs. 21a-c display the number of arc damage events detected in each OS set from datasets from **1-1-1** to **1-3-4** . The frequency and distribution of arc damage events on the time axis are shown in Supplementary Figs. 21d-f. For the datasets from **1-1-1** to **1-1-4** in Supplementary Figs. 21a and 21d, the number of arc damage events was not considered meaningful because of the variations in the axial runout errors of the two clamped specimens and the difficulty in maintaining a consistent load distribution for each dataset. In contrast, the spatial distribution of arc damage exhibited an alternating pattern, which was consistent with the expected behavior. In the dataset of datasets **1-1-3**, the relatively low frequency of arc events may be attributed to the minimal axial runout between the clamped specimens. Supplementary Figs. 21b and 21e show the arc numbers and temporal distributions of arc damage events under the constant load loading mode, respectively. Supplementary Figs. 21c and 21f present the corresponding results for the constant minor load loading mode. The statistical results indicate that the number of arc damage events increases with increasing current and rotational speed, and decreases with increasing load, all of which conform to the established regular pattern. The distribution of arc damage was consistent with the observation that the arc frequency in the early stages of friction was relatively low and increased in the later stages of friction. This trend can be attributed to the progressive deterioration of the frictional interface and the increased system instability caused by arc erosion and frictional damage within the friction pair. In summary, the effectiveness of the arc damage identification model based on the OS as input was validated by the observed frequency and distribution of arc damage, both of which are consistent with the established regular pattern.

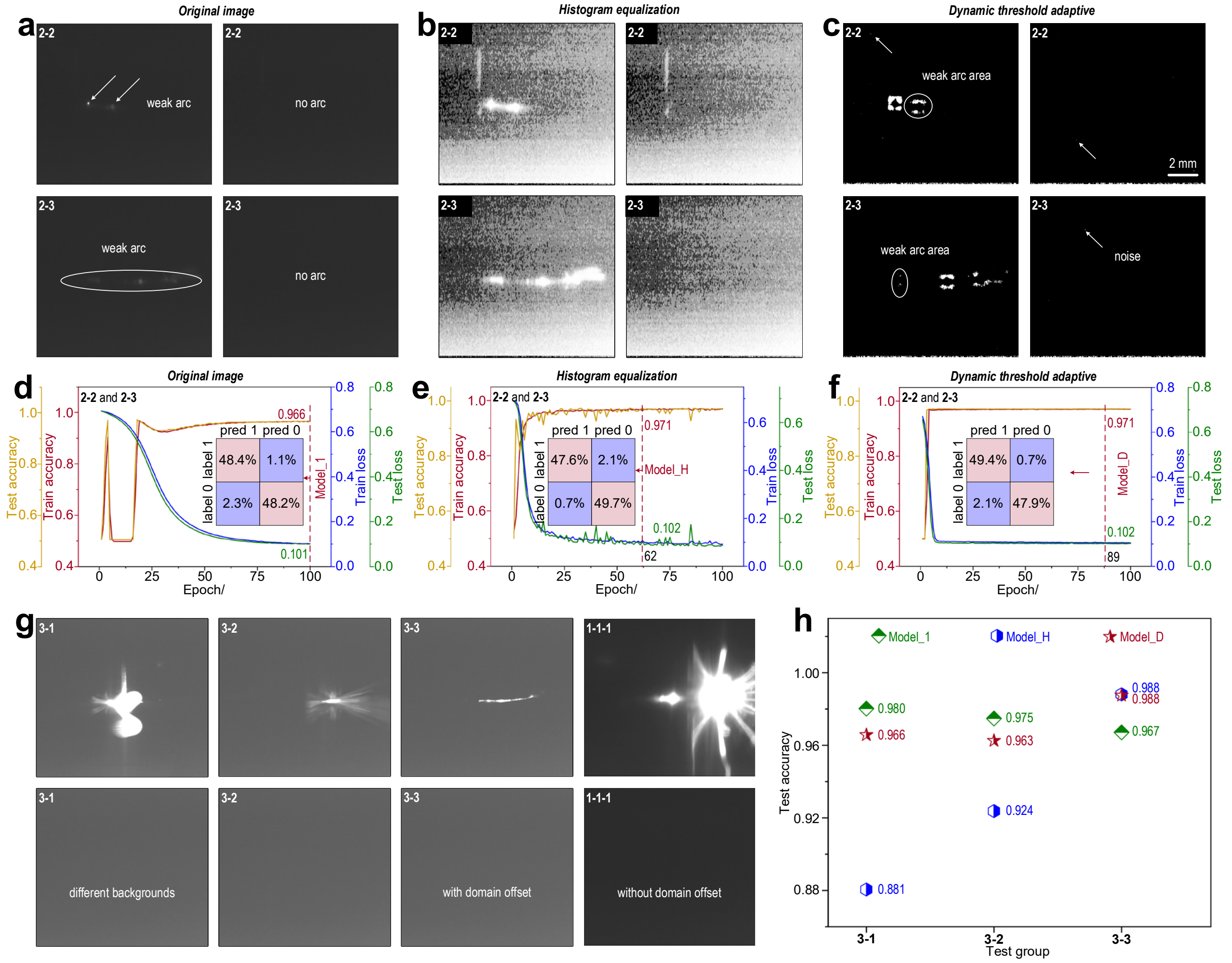

Supplementary Figure 20. **The effect of optical signal preprocessing on arc detection model performance with optical images as input**. **a** Raw images of typical optical signals from datasets **2-2** and **2-3**. **b-c** Images obtained via histogram equalization and dynamic threshold adaptive processing of these original images, respectively. **d-f** Training epoch-loss/accuracy curves of the arc detection model with the original image, the image processed via histogram equalization and the image processed via the dynamic threshold adaptive as inputs, including the corresponding normalized confusion matrix. **g** Comparison of raw images in experiments between datasets from **2-2** to **3-3** and **1-1-1**. **h** Generalization performance of the model shown in d-f for image signals in datasets from **3-1** to **3-3**. Source data are provided as a Source Data file.

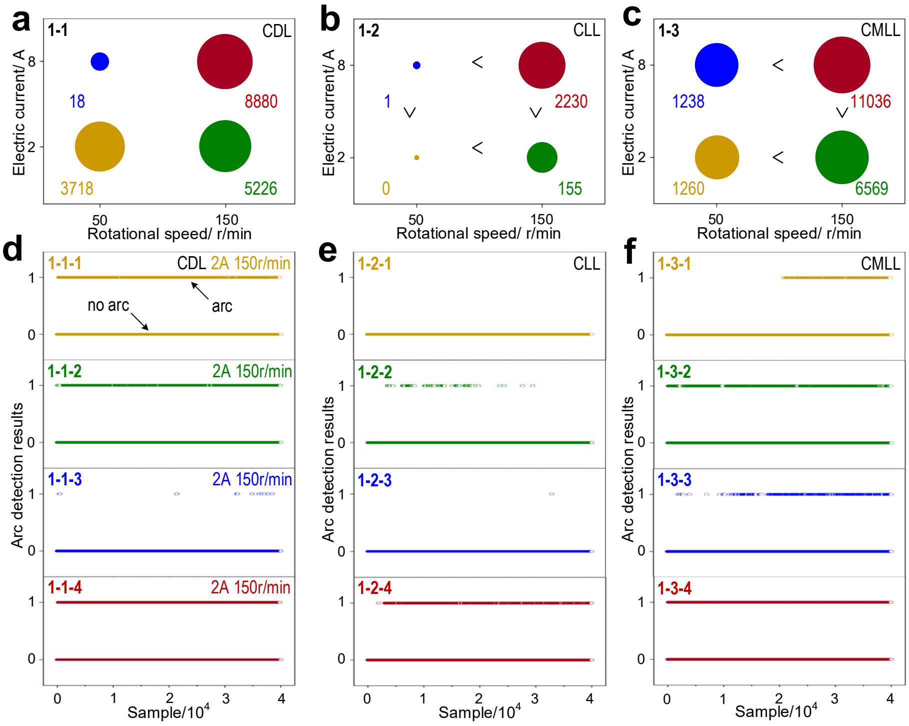

Supplementary Figure 21. **Application of the arc detection model with optical images processed by a dynamic threshold adaptive as input**. **a-c** Arc damage frequency calculated via the arc damage detection model using optical signals for datasets from **1-1-1** to **1-1-4** under constant displacement loading (CDL) mode, datasets from **1-2-1** to **1-2-4** under constant load loading (CLL) mode, and datasets from **1-3-1** to **1-3-4** under constant minor load loading (CMLL) mode. **d-f** Distributions of the corresponding arc damage on the time axis. Source data are provided as a Source Data file.

Supplementary Discussion 8. **Cost evaluation of the arc detection model**

To assess the feasibility of deploying the arc detection model based on optical signals (OSs), acoustic signals (ISs), or acoustic signals (ASs) on an in-situ testing system for real-time monitoring, the inference time was measured via Python implementation, as detailed in Supplementary Table 16. The total inference time comprises two parts: data preprocessing and model inference. For arc detection using OS as input, the total time required for dynamic threshold adaptation preprocessing and inference with the RSNet model across 36,000 samples is 88.77 seconds. In contrast, IS-based arc detection involves neither preprocessing nor deep learning model inference, resulting in a substantially lower computational cost than the OS-based approach. For AS-based arc detection via the RDNet41 model, the total processing time for 36,000 samples is 893.8 s. All three detection methods exhibit inference times that are shorter than the corresponding test duration of 900 s, thereby fulfilling the real-time requirements for arc discrimination in the in-situ testing system. Moreover, for deployment scenarios demanding greater efficiency, implementing the models in "C" language can further reduce the computational cost of arc detection. Additionally, high-quality in-situ detection modules are costly. This case is expected to simultaneously equip high-speed cameras, infrared thermal imagers, and acoustic emission sensors with the ability to generate high-throughput statistics of the arc damage frequency under current-carried friction conditions, which would enhance the flexibility and utilization efficiency of in-situ detection modules in material data factories.

Supplementary Table 14. **The time cost of signal preprocessing**

(Not included in the time for data reading, data storage, and data segmentation)

Data preprocessing method	Time cost for processing each signal segment/s
Convert AS from time domain into the 1 M spectrogram form	3.453×10^{-2}
Convert AS from time domain into the 34 kHz spectrogram form	2.434×10^{-2}
Convert AS from time domain into the 1 MHz frequency domain form	4.156×10^{-3}
Convert OS based on the dynamic threshold adaptive	1.347×10^{-3}
Convert OS based on the histogram equalization	5.155×10^{-3}

Supplementary Table 15. **Training time costs of machine learning models**

(Not included in the time for data reading, data storage, and data segmentation)

Model	Samples number	Epoch	Training time per epoch/ s	Testing time per epoch/ s	Total training time/s
GRU	9000	200	4.157	4.034	1638
MLP		100	9.855×10^{-3}	3.621×10^{-2}	13.48
MNet2_1			8.225	3.619	1184
MNet2_2			8.532	3.755	1229
MNet2_4			8.411	3.645	1206
MNet2_8			8.623	3.710	1233
MNet2_16			8.364	3.566	1193
MNet2_32			8.650	3.660	1231
MNet2_64			8.999	4.124	1312
MNet3			9.190	3.954	1314
MNet4			9.109	4.006	1312
RANet		50	64.35	26.59	4547
RDNet13		100	8.878	3.732	1261
RDNet41			9.848	4.391	1424
RMNet			9.881	4.105	1399
RRNet10			11.16	4.683	1584
RRNet18			8.921	3.728	1265
RSNet (3 channels input)	10134	100	72.24	29.89	10213
RSNet (1 channels input)			28.08	11.34	3942
SVM	600	/	/	/	1.557×10^{-2}

Supplementary Table 16. **The inference time costs for the deployed model to detect the arc**
(Inference on the basis of Supplementary Tables 14 and 15)

Input signal	Model	Samples number	Data preprocessing time/ s	Inference time of model/ s	Total inference time/s
AS	MNet2_1	36000	876.2	14.48	890.7
AS	MNet2_2			15.02	891.3
AS	MNet2_4			14.58	890.8
AS	MNet2_8			14.84	891.1
AS	MNet2_16			14.26	890.5
AS	MNet2_32			14.64	890.9
AS	MNet2_64			16.50	892.7
AS	MNet3			15.82	892.1
AS	MNet4			16.02	892.3
AS	RDNet13			14.93	891.2
AS	RDNet41			17.56	893.8
AS	RMNet			16.42	892.7
AS	RRNet10			18.73	895.0
AS	RRNet18			14.88	891.1
OS	RSNet (3 channels input)		0 or 185.6	106.2	106.0 or 291.8
OS	RSNet (1 channels input)		48.49	40.28	88.77

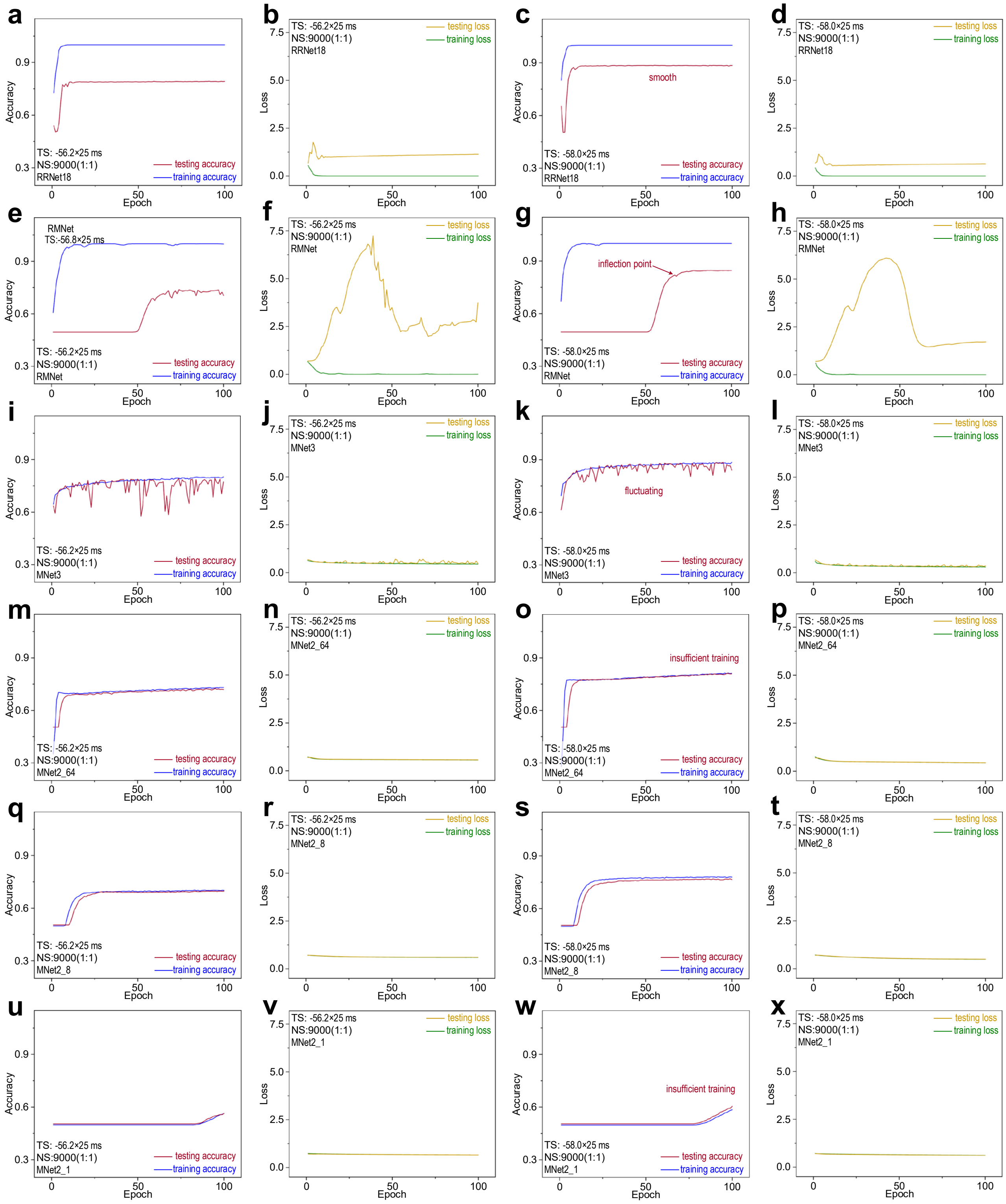

Supplementary Figure 22. **The typical epoch-loss and accuracy curves corresponding to the kernel model for various numbers of samples (NSs) and time shifts (TSs) in Fig. 3c**. Source data are provided as a Source Data file.

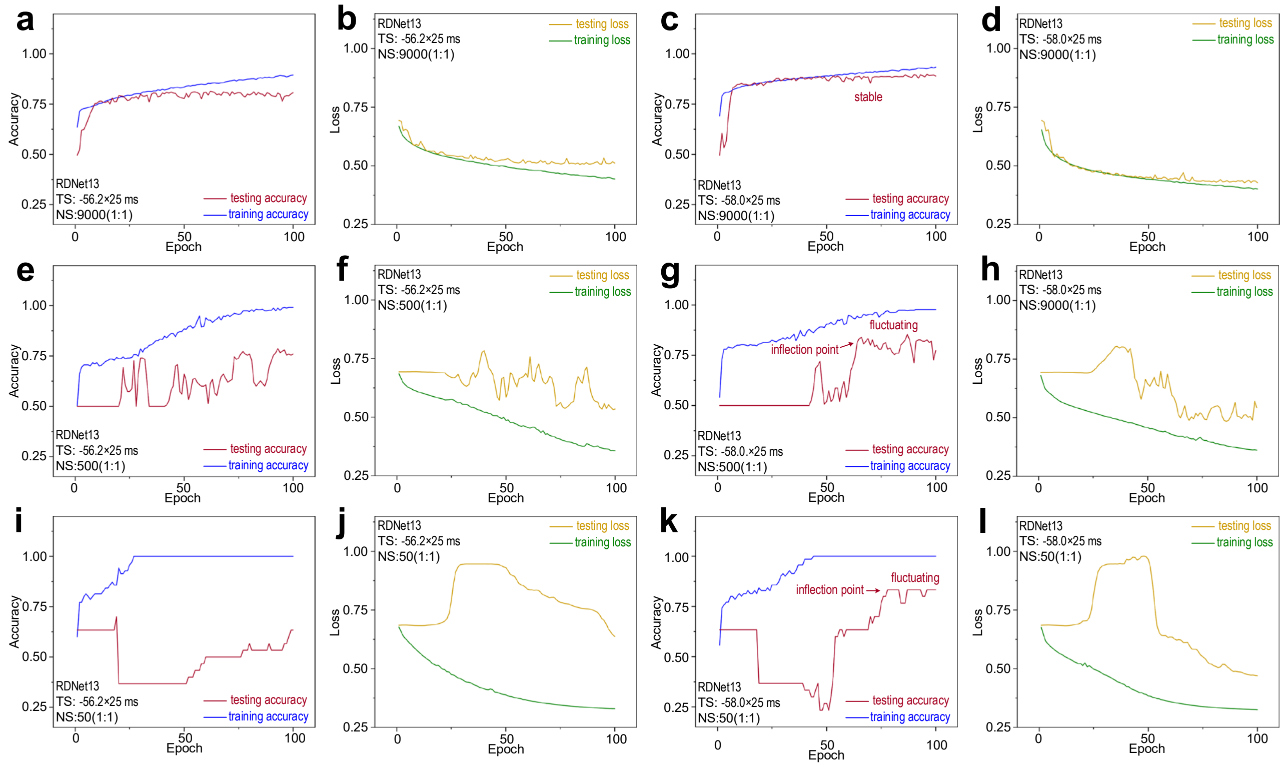

Supplementary Figure 23. **The typical epoch-loss and accuracy curves corresponding to the kernel model for various numbers of samples (NSs) and time shifts (TSs) in Fig. 3f.** Source data are provided as a Source Data file.

Supplementary Discussion 9. **Influence of the number of samples on alignment accuracy**

Supplementary Fig. 24 illustrates the effect of the number of samples (NS) on alignment accuracy on the basis of the lightweight kernel model Mnet2_1, which was trained with learning rates ranging from 2×10^{-4} to 0.5. Consistent with alignments performed via larger kernel models, the alignment accuracy generally deteriorates as the NS decreases. Furthermore, the mean standard deviation of the maximum testing accuracy across time shifts increases with increasing NS, as shown in Supplementary Fig. 24a. Referring to the corresponding typical epoch-accuracy and loss curve in Supplementary Fig. 25, the increase in standard deviation is attributed to the unstable training process of the kernel model. Moreover, owing to the limited expressive power of the kernel model, alignment based on a relatively large NS of 5000 sets of data (Supplementary Figs. 25e-h) results in an unstable training process. Even in certain instances of alignment with only 50 samples (Supplementary Figs. 25m–p), the testing accuracy exceeded the training accuracy. Although the training of the kernel model showed an unstable training process for alignment with 5,000 samples or fewer, Supplementary Fig. 24a indicates that the maximum degradation in alignment resolution within the range of 50–9000 samples is within 35 ms. The relationships between the NS and the maximum testing and training accuracies are shown in Supplementary Fig. 24b. As the NS decreases, both the training and testing accuracies of the model increase. Moreover, the gap between the testing and training accuracies remains small, suggesting that the expressive capacity of the kernel model is fully leveraged. In contrast, for the corresponding model shown in Fig. 3e, a substantial gap exists between the testing and training accuracies, indicating that the model retains unutilized expressive capacity. For the alignment task using 9,000 data samples, the kernel model results in relatively low maximum training and testing accuracy, suggesting a state of underfitting (Supplementary Figs. 25a–d). Correspondingly, the alignment accuracy has been slightly affected. Supplementary Fig. 24c presents a bubble plot illustrating the relationships among the NSs, time shifts, and maximum testing accuracies. As the NS decreases, the peak-shaped trend in maximum testing accuracy around the semantic time shift diminishes, whereas fluctuations in the maximum testing accuracy across different time shifts become more irregular, collectively leading to a reduction in alignment accuracy.

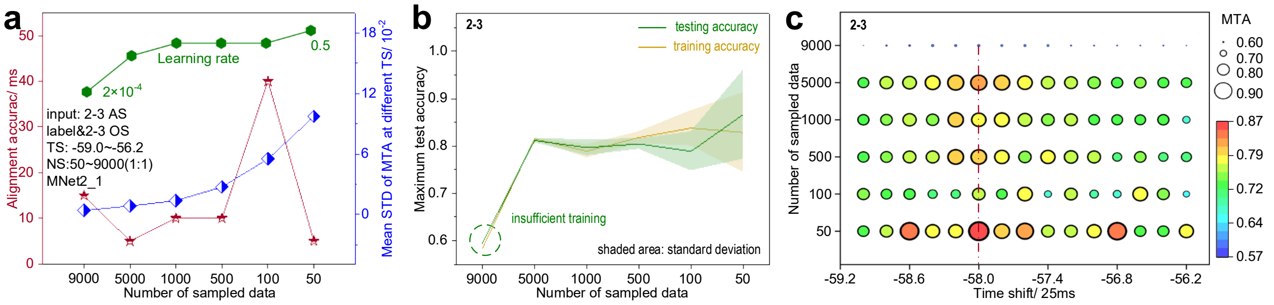

Supplementary Figure 24. **Analysis of the effect of the number of samples (NS) on alignment accuracy.** **a** The variation in alignment accuracy and standard deviation (STD) of maximum testing accuracy (MTA) using Mnet2_1 kernel models, as the NS varies. **b** Corresponding NS–maximum accuracy curve. **c** Corresponding bubble chart of the MTA across different time shifts (TSs) (step size: 5 ms). and NS. Source data are provided as a Source Data file.

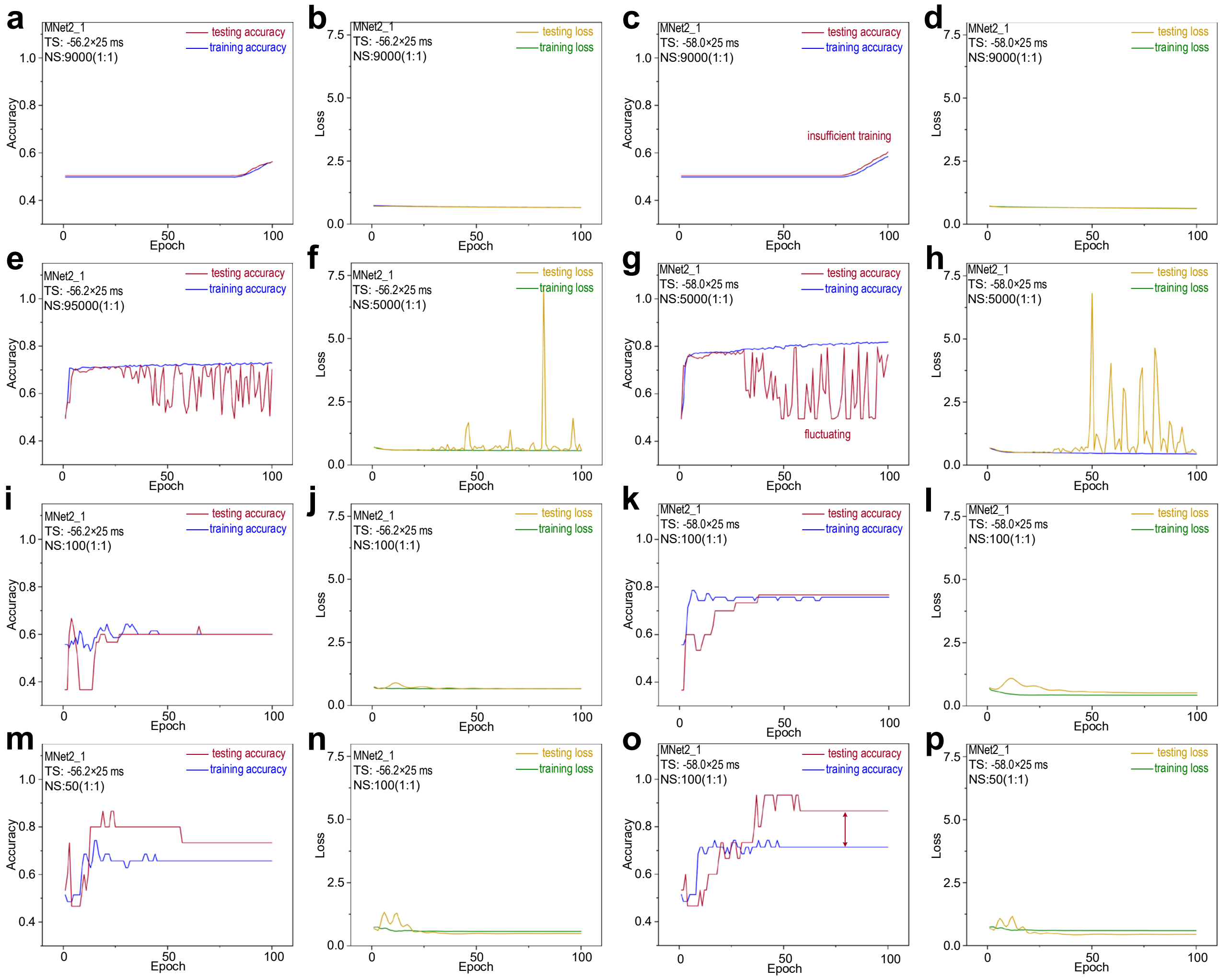

Supplementary Figure 25. **The typical epoch-loss and accuracy curves corresponding to the kernel model for various numbers of samples (NSs) and time shifts (TSs) in Supplementary Fig. 24c.** Source data are provided as a Source Data file.

Supplementary Discussion 10. **The alignment method based on frequency domain similarity**

The implementation and results of the frequency domain similarity-based alignment method are shown in Supplementary Figs. 26a–c. To address the challenge of defining explicit correlation functions between heterogeneous data modalities, an approach leveraging intramodal similarity was proposed under the assumption that spectrograms corresponding to the same label category—such as arc response and no arc response—exhibit inherent similarity. As shown in Supplementary Fig. 26a, for the optical signal (OS) modality, similarity is quantified by computing the coincidence accuracy between the OS label sequence and the offset OS label sequence. For the acoustic signal (AS) modality, the time-domain signal of each segment is first transformed into the frequency domain. To ensure comparability, the amplitude of the frequency-domain signal is scaled such that the sum of the amplitudes remains constant. Inspired by the geometric principle that the square in a rectangle with equal perimeter yields the maximum area, the similarity between two signals is quantified via the dot product between the scaled frequency-domain vectors, where an increasing dot product denotes greater similarity between two signals. For the AS segment sequence, the original AS sequence corresponds to the time shift, whereas each offset AS sequence corresponds to the superposition of the time shift and offset. The offset-label coincidence accuracy curve, derived from the OS sequence, and the offset-frequency domain similarity curve, obtained from the AS sequence across different time shifts, are shown in Supplementary Fig. 26b. Suppose that a given time shift accurately reflects accurate semantic alignment. In that case, the positive correlation between the label coincidence accuracy and frequency domain similarity at that time shift should be stronger than that observed at other time shifts. The mathematical formulation of this algorithm is shown in Supplementary Equation (1):

$$\left\{\begin{array}{l} S_y[t_{\mathrm{of}}] = \sum_{n=1}^{N} M(y_1[n,0], y_1[n,t_{\mathrm{of}}])/N \\ S_{\mathrm{fft}}[t_{of},t] = \sum_{n=1}^{N} k \cdot \mathbf{F}_{2,\mathrm{norm}}^{\mathrm{fft}}[n,t] \bullet \mathbf{F}_{2,\mathrm{norm}}^{\mathrm{fft}}[n,t_{\mathrm{of}}+t] \\ c_{\mathrm{Sr}}[t] = 1 - \frac{6\cdot\sum_{t_{\mathrm{of}}=-T_{\mathrm{of}}}^{T_{\mathrm{of}}} d_n^2(S_y[t_{\mathrm{of}}], S_{\mathrm{fft}}[t_{\mathrm{of}},t])}{N^3-N} \\ c_{\mathrm{Kr}}[t] = \frac{C(S_y[t_{\mathrm{of}}], S_{\mathrm{fft}}[t_{\mathrm{of}},t]) - D(S_y[t_{\mathrm{of}}], S_{\mathrm{fft}}[t_{\mathrm{of}},t]}{\frac{1}{2}\cdot N\cdot(N-1)} \\ t_{\mathrm{Sr}} = \max_t c_{\mathrm{Sr}}[t] \\ t_{\mathrm{Kr}} = \max_t c_{\mathrm{Kr}}[t] \\ T_{\mathrm{Sr}} = t_{\mathrm{Sr}} \cdot T_p \\ T_{\mathrm{Kr}} = t_{\mathrm{Kr}} \cdot T_p \end{array}\right\} t_{\mathrm{of}} \in \mathrm{Z}, y_1[n,t] \in \{0,1\} \quad (1)$$

where $y_1[n,0]$, $S_y[t_{\mathrm{of}}]$, $\mathbf{F}_{2,\mathrm{norm}}^{\mathrm{fft}}[n,t]$, and $S_{\mathrm{fft}}[t_{of},t]$ denote the label obtained from signal $x_1[n,0]$, the label coincidence accuracy between sequences $\{y_1[0,0], y_1[1,0],\cdots, y_1[N-1,0]\}$ and sequences $\{y_1[0,t_{\mathrm{of}}], y_1[1,t_{\mathrm{of}}],\cdots, y_1[N-1,t_{\mathrm{of}}]\}$, the scaled frequency-domain vectors of signal $x_2[n,t]$ (where the sum of the elements of each vector is equal), and the frequency-domain similarity between sequences $\{\mathbf{F}_{2,\mathrm{norm}}^{\mathrm{fft}}(0,t),\ \mathbf{F}_{2,\mathrm{norm}}^{\mathrm{fft}}(1,t),\ \cdots,\ \mathbf{F}_{2,\mathrm{norm}}^{\mathrm{fft}}(N-1,t)\}$ and $\{\mathbf{F}_{2,\mathrm{norm}}^{\mathrm{fft}}(0,t+t_{\mathrm{of}}),\ \mathbf{F}_{2,\mathrm{norm}}^{\mathrm{fft}}(1,t+t_{\mathrm{of}}),\ \cdots,\ \mathbf{F}_{2,\mathrm{norm}}^{\mathrm{fft}}(N-1,t+t_{\mathrm{of}})\}$, respectively. $c_{\mathrm{Sr}}[t]$ and $c_{\mathrm{Kr}}[t]$ denote the Spearman rank coefficient and the Kendall rank coefficient, respectively. d^n denotes the rank difference between the matching degree sequences $\{S_y[-T_{\mathrm{of}}],\ S_y[-T_{\mathrm{of}}+1],\ \cdots,\ S_y[T_{\mathrm{of}}]\}$ and the similarity sequence $\{S_{\mathrm{fft}}[-T_{\mathrm{of}},t],\ S_{\mathrm{fft}}[-T_{\mathrm{of}}+1,t],\ \ldots,\ S_{\mathrm{fft}}[T_{\mathrm{of}},t]\}$. $C(S_y[t_{\mathrm{of}}], S_{\mathrm{fft}}[t_{\mathrm{of}},t])$ and $D(S_y[t_{\mathrm{of}}], S_{\mathrm{fft}}[t_{\mathrm{of}},t])$ denote the logarithms of the consistent and inconsistent elements between sequences $\{S_y[-T_{\mathrm{of}}],\ S_y[-T_{\mathrm{of}}+1],\ \cdots,\ S_y[T_{\mathrm{of}}]\}$ and $\{S_{\mathrm{fft}}[-T_{\mathrm{of}},t],\ S_{\mathrm{fft}}[-T_{\mathrm{of}}+1,t],\ \ldots,\ S_{\mathrm{fft}}[T_{\mathrm{of}},t]\}$, respectively. Here, T_{of} is set to 8. t_{Sr} and t_{Kr} denote the semantic alignment parameters between the semantic sequences of signals $x_1[n,0]$ and $x_2[n,0]$ calculated on the basis of the Spearman rank correlation coefficient and the Kendall rank correlation coefficient, respectively. T_{Sr} and T_{Kr} are the corresponding semantic time shifts, and the search range of the semantic time shift is $\mathrm{t}\cdot T_p$. The pseudocode corresponding to the above algorithm is shown in Algorithm 2.

The sampling methods and ratios employed in Supplementary Figs. 26b-c are consistent with those utilized in Figs. 3g-i, with 9,000 samples analyzed for the former. The time shift–level correlation coefficient curve generated by the proposed algorithm is presented in Supplementary Fig. 26c. Both the Spearman and the Kendall rank correlation coefficients reach their extrema at a time shift of -58.0×25 ms, indicating that the semantic time shift t_o^a is -58.0 × 25 ms with an alignment accuracy of 5 ms. This estimated semantic time shift lies within the expected reference range and

is consistent with the outcome derived from the proposed neural network-based alignment architecture. Furthermore, large-scale data analysis confirms that spectrograms exhibit substantial similarity within the arc response category, whereas those that display clear distinctions between arc and no-arc responses.

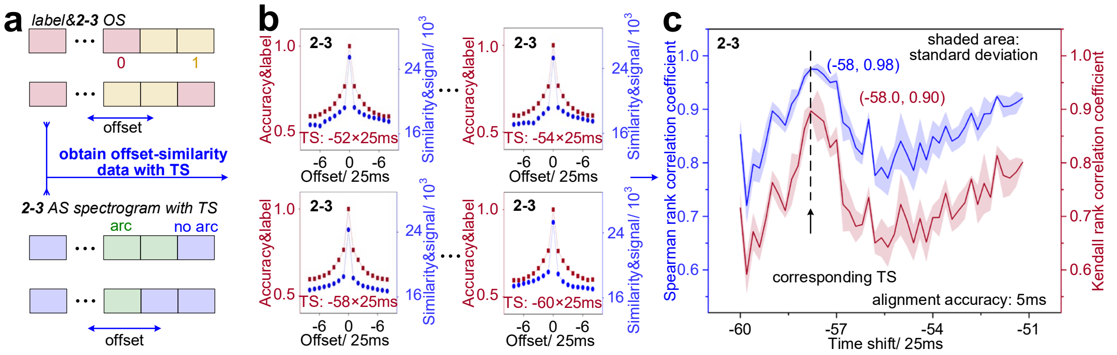

Supplementary Figure 26. **The alignment method on the basis of frequency domain similarity. a** Implement schematic of the alignment method. **b** Offset-accuracy and similarity curves under various time shifts (TSs). **c** Alignment results based on the method between optical signals (OS) and acoustic signals (AS). Source data are provided as a Source Data file.

Supplementary Algorithm 2. **The frequency domain similarity-driven data alignment method**

**Input:** the list of normalized frequency domain signal of AS and label list.	
1	initialization, including the search ranges of time shift parameter t, ranges of offset parameter t_{of} and number of samples N, etc.
2	**for** t in range list:
3	randomly extract equal proportional labels of "0" and "1" from the label list to form a list $\{y_1[0,0],\ y_1[1,0],\ \cdots,\ y_1[N-1,0]\}$.
4	extract the corresponding list of scaled frequency domain signal of AS $\{\mathbf{F}^{\text{fft}}_{2,\text{norm}}(0,t),\ \mathbf{F}^{\text{fft}}_{2,\text{norm}}(1,t),\ \cdots,\ \mathbf{F}^{\text{fft}}_{2,\text{norm}}(N-1,t)\}$.
5	**for** t_{of} in list $\{-T_{\text{of}},\ -T_{\text{of}}+1,\ \cdots,\ T_{\text{of}}\}$:
6	calculate the coincidence accuracy $S_y[t_{\text{of}}]$ between label list $\{y_1[0,0],\ y_1[1,0],\ \cdots,\ y_1[N-1,0]\}$ and offset label list $\{y_1[0,t_{\text{of}}],\ y_1[1,t_{\text{of}}],\ \cdots,\ y_1[N-1,t_{\text{of}}]\}$ according to Supplementary Equation (1).
7	**for** t_{of} in list $\{-T_{\text{of}},\ -T_{\text{of}}+1,\ \cdots,\ T_{\text{of}}\}$:
8	calculate the degree of similarity $S_{\text{fft}}[t_{\text{of}},t]$ between frequency domain signal list $\{\mathbf{F}^{\text{fft}}_{2,\text{norm}}(0,t),\ \mathbf{F}^{\text{fft}}_{2,\text{norm}}(1,t),\ \cdots,\ \mathbf{F}^{\text{fft}}_{2,\text{norm}}(N-1,t)\}$ and offset frequency domain signal list $\{\mathbf{F}^{\text{fft}}_{2,\text{norm}}(0,t+t_{of}),\ \mathbf{F}^{\text{fft}}_{2,\text{norm}}(1,t+t_{of}),\ \cdots,\ \mathbf{F}^{\text{fft}}_{2,\text{norm}}(N-1,t+t_{of})\}$ according to Supplementary Equation (1).
9	calculate the Spearman rank coefficient $c_{\text{Sr}}[t]$ and Kendall rank coefficient $c_{\text{Kr}}[t]$ for list $\{S_y[-T_{\text{of}}],\ S_y[-T_{\text{of}}+1],\ \cdots,\ S_y[T_{\text{of}}]\}$ and $\{S_{\text{fft}}[-T_{\text{of}},t],\ S_{\text{fft}}[-T_{\text{of}}+1,t],\ \ldots,\ S_{\text{fft}}[T_{\text{of}},t]\}$ based on Supplementary Equation (1).
11	calculate the semantic time shift T_{Sr} and T_{Kr} between signal $x_1[n,0]$ and $x_2[n,0]$ based on Supplementary Equation (1).

Supplementary Table 17. **The time costs of alignment based on RDNet13**

(Inference on the basis of Supplementary Tables 14 and 15)

Samples number	Data preprocessing time/ s	Training time per search steps/s	Search steps	Total time/ s
9000	1095	1261	15	20010
5000	608.5	716.5	15	11356
1000	121.7	139.2	15	2209.7
500	60.85	65.97	15	1050.4
100	12.17	12.50	15	199.67
50	6.085	7.037	15	111.64

Supplementary Table 18. **The time costs of alignment on the basis of frequency domain similarity**

(Inference on the basis of Supplementary Tables 14 and 15)

Samples number	Data preprocessing time/ s	The time for caculate rank correlation coefficient for per search step/s	Search steps	Total time/ s
9000	187.0	230.9	45	10578
5000	103.9	133.3	45	6102.4
1000	20.78	28.84	45	1318.6
500	10.39	15.34	45	700.69
100	2.078	3.162	45	144.37
50	1.039	1.806	45	82.309

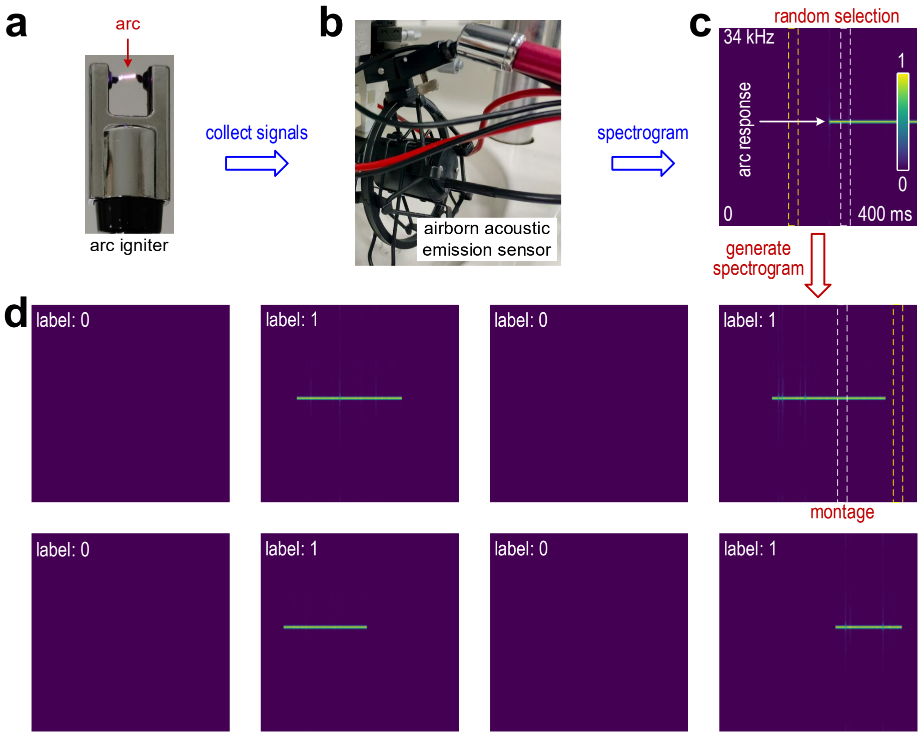

Supplementary Figure 27. **Method for generating corresponding artificial spectrograms and labels on the basis of the spectrogram of the arc response of the arc igniter. a** Schematic diagram of the arc igniter. **b** Airborne acoustic emission sensor for collecting acoustic signals corresponding to the arc response. **c** Spectrogram of the acoustic signal corresponding to the arc response of the arc igniter. (The color bar indicates the normalized intensity, scaled to the [0, 1] range for each spectrogram individually.) **d** Spectrogram and corresponding labels generated via Supplementary Algorithm 3. Source data are provided as a Source Data file.

Supplementary Algorithm 3. **Generate arc response label and corresponding spectrogram**

**Input:** original spectrogram **S** with 0-400 ms, and $[a,b]$ ms corresponds to the arc response. $a,b \in [0,400]$
randomly generate a label list composed of equal proportions of "0" and "1"
**for** element in label list:
**if** elemet = 1:
randomly select interval $[c,d]$ from 0-400 ms
regarding interval $[c,d]$, randomly select original spectrogram **S** in $[a,b]$ for stitching
regarding intervals $[0,c]$ and $[d,400]$, randomly select original spectrogram S in $[0,a]$ and $[b,25]$ for stitching
**else:**
randomly select spectrogram **S** in $[0,a]$ and $[b,400]$ for stitching
save label list and corresponding spectrogram image

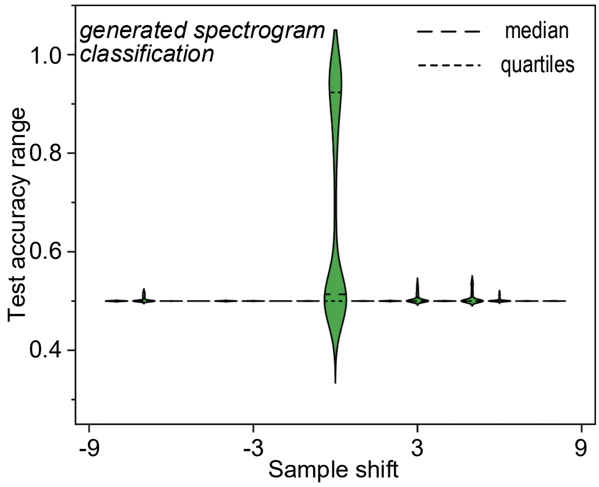

Supplementary Figure 28. **The shift-accuracy distribution corresponding to Fig. 4c.** Source data are provided as a Source Data file.

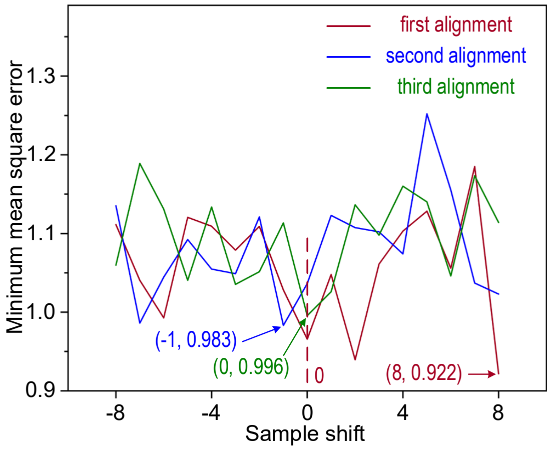

Supplementary Figure 29. **The shift-maximum testing accuracy curve of three repeated alignments corresponding to Fig. 4h.** Source data are provided as a Source Data file.

Supplementary Discussion 11. **Supplement of alignment architecture promotion**

For image data, alignment via the RSNet kernel model was performed between infrared signal images and corresponding labels in dataset **2-3** (Supplementary Figs. 30a-c), as well as between optical signal (OS) images and their corresponding labels in dataset **2-3** (Supplementary Figs. 30d-f), both of which achieved favorable alignment results. For tabular data, eight statistical features—mean, maximum, minimum, range, variance, skewness, kurtosis, and entropy—were extracted from dataset **2-3** OS images to form 8-dimensional feature vectors. The alignment between these vectors and their corresponding labels was performed via a multilayer perceptron (MLP) kernel model (Supplementary Figs. 30g–i). The model achieved maximum training and testing accuracy at a sample shift of 0, confirming the suitability of the proposed alignment architecture for structured tabular data. Additionally, the input vectors of the function $z = |x| + \mathrm{step}(y)$ and corresponding labels based on the support vector machine (SVM) were successfully aligned (Supplementary Figs. 30j-l). Subsequently, alignments between the input vectors and outputs of the above two functions were performed via a regression-based kernel model (Supplementary Fig. 31). Both models receive a two-dimensional feature vector composed of x and y as inputs and predict the corresponding z values. Both the training and testing mean squared errors reached their minimum at a sample shift of 0 for the two models, demonstrating that the proposed alignment architecture is also applicable to regression tasks.

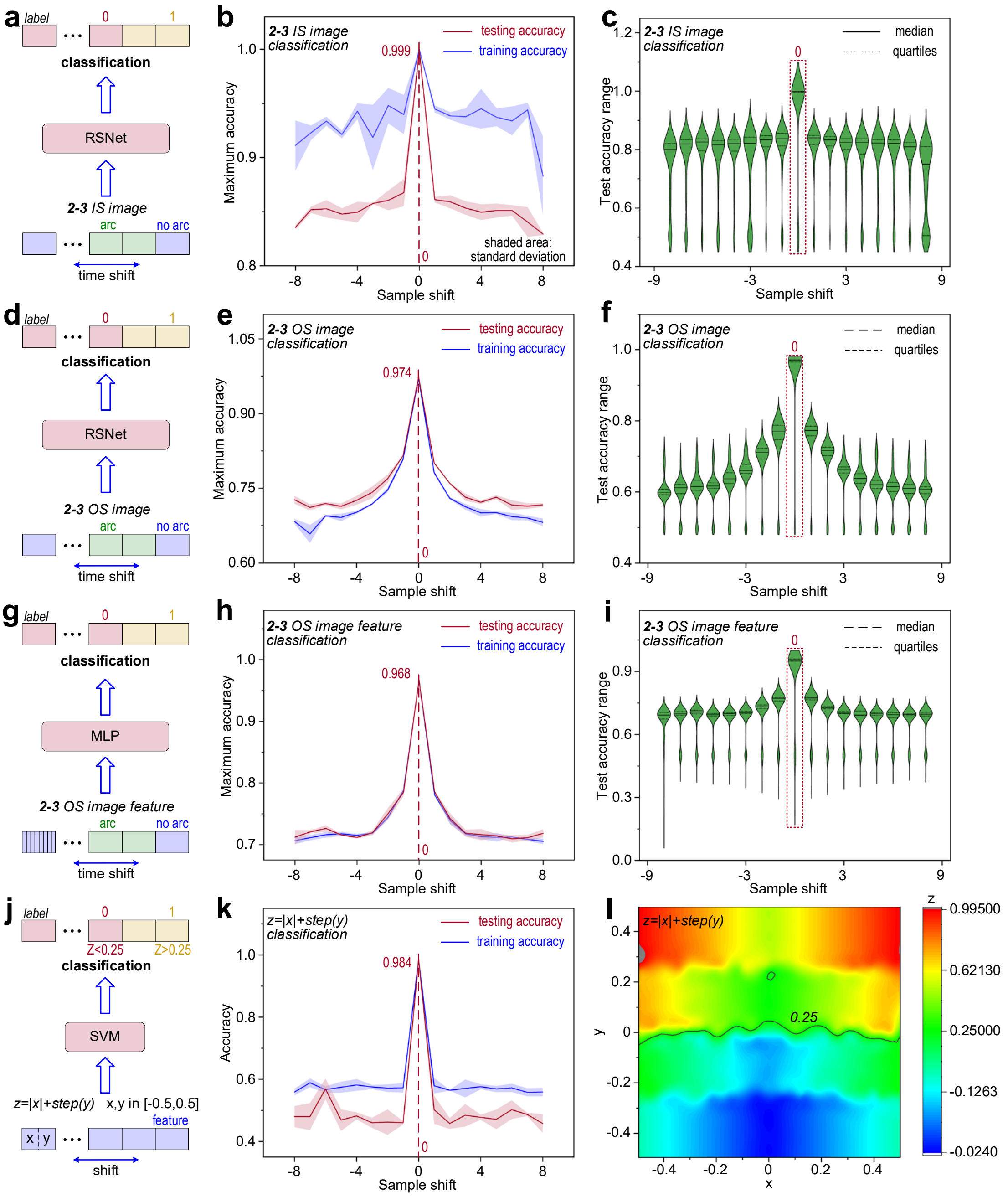

Supplementary Figure 30. **Promotion of the alignment architecture (supplements for Figure 4). a** Schematic diagram of aligning dataset **2-3** infrared signals (IS) with their labels via the RSNet kernel model. **b-c** Sample shift-maximum accuracy curve and testing accuracy distribution corresponding to a, respectively. **d** Schematic diagram of aligning dataset **2-3** optical signals (OS) with their labels via the RSNet kernel model. **e-f** Sample shift-maximum accuracy curves and accuracy distributions corresponding to d, respectively. **g** Schematic diagram of the alignment of dataset **2-3** feature vectors of the OS with their labels via multilayer perceptron (MLP) kernel model. **h-i** The shift-maximum accuracy curve and testing accuracy distribution corresponding to g, respectively. **j-k** Schematic diagram of aligning the input of artificially constructed functions with the corresponding output via the support vector machine (SVM) kernel model and corresponding alignment results. **l** Cloud diagram of the constructed functions in j. Source data are provided as a Source Data file.

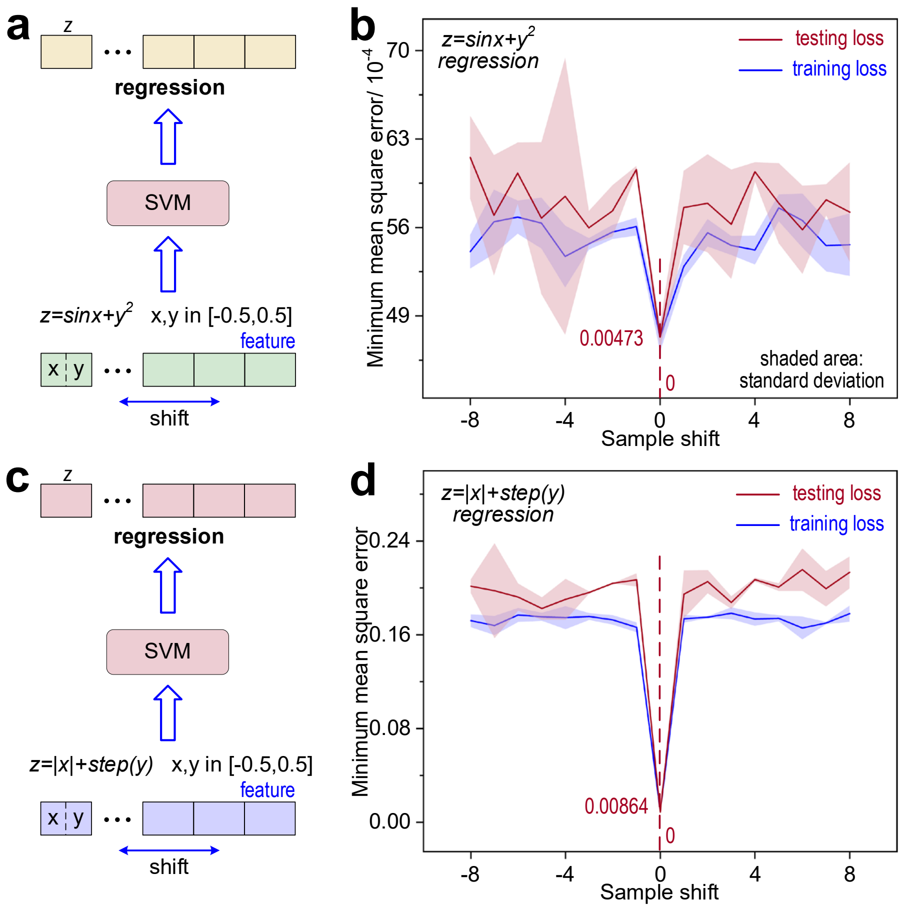

Supplementary Figure 31. **Promotion of the alignment architecture (supplements for Fig. 4). a-b** Schematic diagram of aligning the input of artificially constructed functions ($z = \sin x + y^2$) with the corresponding output via the support vector machine (SVM) kernel model and corresponding alignment results. **c-d** Schematic diagram of aligning the input of artificially constructed functions ($z = |x| + \mathrm{step}(y)$) with the corresponding output via the SVM kernel model and corresponding alignment results. Source data are provided as a Source Data file.

Supplementary Discussion 12. **Cost evaluation of alignment architecture**

The computational time required for training a kernel model is provided in Supplementary Table 15. In a fixed semantic time-shift system, the one-time alignment cost of the proposed architecture remains acceptable. In typical machine learning workflows, identifying semantic features that facilitate the extraction of critical information can enhance the performance of traditional, lightweight models. For systems that require frequent alignment, the support vector machine-based kernel model is computationally feasible when it is applied to small sample sizes within limited search ranges. Computational power is widely recognized as the cornerstone of the Fourth Industrial Revolution. In this context, the demand for quantum computing has grown increasingly urgent [15-17], driven by the application of artificial intelligence in the fields of science and industry. Similarly, for systems that require continuous alignment, deploying alignment models built upon a more complex kernel model demands substantial computational resources. The alignment requirements can vary significantly across systems. For systems with low real-time demands, fast kernel model convergence, or offline alignment needs, the applicability and efficiency of the proposed architecture should be evaluated on a case-by-case basis.

Supplementary References

[1] Li, C., & Ma, Z., Li, J., Wang, C., Zhao, H., Ren, L. Development of an in-situ current-carrying friction testing instrument and experimental analysis under the background of the Fourth Industrial Revolution. *Mech. Syst. Signal. Pr.* **223**, 111936 (2025).

[2] & Pandiyan, V., Wróbel, R., Richter, R.A., Leparoux, M., Leinenbach, C., Shevchik, S. Monitoring of Laser Powder Bed Fusion process by bridging dissimilar process maps using deep learning-based domain adaptation on acoustic emissions. Addit. Manuf. **80**, 103974 (2024).

[3] Zhang, W., Kuang, Y., Xie, H., Li, C. &Ma, Z., Zhao, H., Ren, L. A novel high-velocity impact testing system based on multi-stage electromagnetic coil accelerator and in-situ monitoring signals. *Mech. Syst. Signal. Pr.* 242, 113627 (2026).

[4] Tao, L., Liu, H., Ning, G., Cao, W., Huang, B., & Lu, C. LLM-based framework for bearing fault diagnosis. Mech. Syst. Signal. Pr. **224**, 112127 (2025).

[5] & Kyunghyun, C., Bart, V.M., Caglar. G., Dzmitry B., Fethi, B., Holger, S., Yoshua, B. Learning Phrase Representations using RNN Encoder–Decoder for Statistical Machine Translation. Preprint at https://arxiv.org/abs/1406.1078 (2104).

[6] Rumelhart, D.E., & Hinton, G.E., &Williams, R.J. Learning Internal Representations by Error Propagation. Nature. **323**, 533–536 (1986).

[7] & Krizhevsky, A., Sutskever, I., Hinton, G.E. ImageNet Classification with Deep Convolutional Neural Networks. Commun. ACM. **60**, 84-90 (2017).

[8] & Huang, G., Liu, Z., Van Der Maaten, L., Weinberger, K.Q. Densely Connected Convolutional Networks. Preprint at https://arxiv.org/pdf/1608.06993v3 (2017).

[9] Howard, A.G. & Zhu, M., Chen, B., Kalenichenko, D., Wang, W. Weyand, T., Andreetto, M. Adam. H. MobileNets: Efficient Convolutional Neural Networks for Mobile Vision Applications. Preprint at https://arxiv.org/abs/1704.04861 (2017).

[10] & Liang, H., Zhang, Q., Dai, P. Lu, J. Boosting the Generalization Capability in Cross-Domain Few-shot Learning via Noise-enhanced Supervised Autoencoder. Preprint at https://arxiv.org/abs/2108.05028 (2021).

[11] & He, K., Zhang, X., Ren, S., Sun, J. Deep Residual Learning for Image Recognition. Preprint at https://arxiv.org/abs/1512.03385 (2015).

[12] & Iandola, F.N., Han, S., Moskewicz, M.W., Ashraf, K., Dally, W.J., Keutzer, K. SqueezeNet: AlexNet-level accuracy with 50x fewer parameters and <0.5MB model size. Preprint at https://arxiv.org/abs/1602.07360 (2016).

[13] Cortes, C., & Vapnik, V. Support-vector networks. Mach. Learn. **20**, 273-297 (1995).

[14] & Zhou, B., Khosla, A., Lapedriza, A., Oliva, A., Torralba, A., Learning Deep Features for Discriminative Localization. Preprint at https://arxiv.org/abs/1512.04150 (2016).

[15] Zuo, P., Wang, Q. Luo, Y., Xie. R., Wang, S. Cheng, Z., Bao, L., Wang, Z., Cai, Y. Huang, R., & Sun, Z. Precise and scalable analogue matrix equation solving using resistive random-access memory chips. Nat. Electron. (2025).

[16] Chiu, N., Trapp, E.C., Guo, J., Abobeih, M.H., Stewart, L.M., Hollerith, S., Stroganov, P.L. Kalinowski, M., Geim, A.A., Evered, S.J., Li, S.H., Lyu, X.J., Peters, L.M. Bluvstein, D., Wang, T.T., Greiner, M., Vuletic, V., & Lukin, M.D. Continuous operation of a coherent 3,000-qubit system. Nature. (2025).

[17] Evered, S.J., Kalinowski, M., Geim, A.A. Manovitz, T., Bluvstein, D., Li, S.H. Maskara, N., Zhou, H., Ebadi, S., Xu, M., Campo, J., Cain, M., Ostermann, S., Yelin, S.F., Sachdev, S., Greiner, M., Vuletic, V., Lukin, M.D. Probing the Kitaev honeycomb model on a neutral-atom quantum computer. Nature. **645**, 341-347 (2025).
