# Supplementary material for: Machine learning-driven alignment architecture of heterogeneous data with transient varying semantics
Source: Nat Commun. 2026 Apr 23;17:5604. doi: 10.1038/s41467-026-72377-w (PMC13316067; doi:10.1038/s41467-026-72377-w)
Supplement: Supplementary file 2 — Description of Additional Supplementary Files [file 41467_2026_72377_MOESM2_ESM.pdf]

Description of Additional Supplementary Files

File name: Supplementary Movie 1
Description: Optical signals of dataset 2-3.

File name: Supplementary Movie 2
Description: Infrared signal signals of dataset 2-3.

File name: Supplementary Movie 3
Description: Spectrogram of acoustic signals of dataset 2-3.

File name: Supplementary Movie 4
Description: Class activation mapping of acoustic signal spectrogram corresponding to arc damage.

File name: Supplementary Movie 5
Description: Class activation mapping of acoustic signal spectrogram corresponding to the absence of arc.
