# Supplementary material for: Machine learning-driven alignment architecture of heterogeneous data with transient varying semantics
Source: Nat Commun. 2026 Apr 23;17:5604. doi: 10.1038/s41467-026-72377-w (PMC13316067; doi:10.1038/s41467-026-72377-w)
Supplement: Supplementary file 8 — Transparent Peer Review file [file 41467_2026_72377_MOESM8_ESM.pdf]

nature portfolio

Peer Review File

Machine Learning-driven Alignment Architecture of Heterogeneous Data with Transient Varying Semantics

Corresponding Author: Professor Zhichao Ma

Parts of this Peer Review File have been redacted as indicated to remove third-party material.

This file contains all reviewer reports in order by version, followed by all author rebuttals in order by version.

Version 0:

Reviewer comments:

Reviewer #1

(Remarks to the Author)
The manuscript requires following revisions to be incorporated:
1. Explain why you chose to subtract and divide by min/max pixel values rather than, for example, histogram equalization or adaptive thresholding. Discuss how sensitive your alignment results are to changes in illumination or background.
2. Provide the specific window function $w(t)$ you used (e.g. Hanning, Hamming), the overlap between adjacent 25 ms frames, and the FFT length—this matters for frequency resolution and alignment precision.
3. You mention 4,832 positive optical labels vs. 2,556 infrared labels—discuss how this class imbalance might bias model training and validation. Consider augmenting underrepresented classes (e.g., via synthetic acoustic events) or using stratified sampling.
4. Report inter-rater or intra-rater agreement if labels were assigned by multiple annotators, to quantify label noise.
5. Compare your neural-network alignment method against a classical technique (e.g., cross-correlation or dynamic time warping) on the same 2-3 dataset. Show how much accuracy or precision is gained.
6. Provide timing benchmarks (training/inference cost) so readers can assess practicality for high-throughput in-situ tests.
7. You use a simplified DenseNet-121 for shift estimation. Include an ablation that:
Varies network depth (e.g., DenseBlock counts).
Swaps in a lightweight CNN (e.g., MobileNet or ResNet-18) to show that DenseNet's dense connections are necessary.
Report how alignment precision (in ms) degrades with smaller models.

Reviewer #2

(Remarks to the Author)
The manuscript, "Neural network-driven alignment of ultra-fast acoustic and optical signals," introduces a temporal alignment method for multimodal signals, including optical images, infrared images, and acoustic signals. The authors claim that the proposed method achieves 5 ms alignment accuracy and 90% discrimination accuracy. Although the proposed method may work appropriately for the specific experimental setup presented in the paper, the impact and significance of the manuscript are limited for the reasons below. Therefore, my recommendation is to reject the manuscript.

1) The authors propose a temporal alignment method meticulously crafted for a measurement system they built in [3]. However, timing synchronization across different measurement signals can, in general, be easily achieved using hardware triggers, which are versatile for off-the-shelf measurement devices. It seems that the authors raise an unnecessary problem by not synchronizing the sensors in hardware and trying to solve it. Although it would be effective for their specific measurement system in which the sensors are not synchronized, I doubt the necessity and significance of the proposed method in a general sense.

2) This paper demonstrates the effectiveness of the proposed method only for the one measurement system. There is no discussion about how this method could be used for other systems, so the method is missing generality. If the proposed method is only valid for the specific measurement system, it may not be appropriate for publication in Nature Communications.

3) The authors train the DenseNet model for different time shifts and compare the validation accuracy to determine the time shift. This approach can be interpreted as a grid search with the neural network as a black box discriminator. I believe that the technical novelty and academic contribution from the ML perspective are quite low.

Reviewer #3

(Remarks to the Author)
• The novelty of the manuscript is not clearly established. Further clarification needed how this approach is fundamentally different from existing neural network-based alignment and multimodal fusion methods.
• The paper does not clearly specify the threshold criteria for identifying arc-related acoustic events. Clear information on the amplitude, energy, or statistical thresholds used to distinguish arc events from background signals, and how these thresholds were determined.
• The details of the synchronization process across acoustic, optical, and infrared sensors are not clearly described. A description of the sensor synchronization procedure and the process used to validate the ground truth time shift independently of the neural network would improve clarity.
• According to Nyquist theory, the sampling rate should be at least 2.5 times higher than the maximum AE frequency. The authors used a 2 MHz sampling rate, while the effective frequency goes up to 1 MHz. This may cause loss of important high-frequency information. It is recommended to perform tests with higher sampling rates to confirm whether any data was lost.
• The rationale behind the chosen combinations of load mode, current, and speed is not explained. Justification for selecting these specific parameters and their relevance to realistic or challenging test conditions would strengthen the study.
• The stability of background reference images over time is not addressed. It would be useful to explain how drift in background illumination or temperature was controlled during preprocessing.
• It is not clear whether additional filtering was applied to the acoustic signals beyond bandpass filtering. Details on any temporal or spectral denoising methods used to handle cantilever vibrations or other noise sources would be important.
• No information is provided about the repeatability of alignment results across multiple training runs. Reporting the variance in estimated time shifts across repeated model trainings would help assess consistency.
• There is no direct comparison to conventional alignment techniques such as cross-correlation or dynamic time warping. Including baseline performance comparisons would help demonstrate the advantage of the proposed method.
• All experiments are limited to aluminum and copper alloy friction pairs. Information on whether the model was evaluated on other materials or geometries would be useful to understand generalization.
• The paper does not address whether the measured time shifts include physical delays such as acoustic propagation time. Clarification on whether these delays were measured and accounted for would improve interpretation.
• Class activation maps are shown qualitatively without detailed analysis. Quantification of which frequency bands contributed most to arc detection would improve interpretability.
• Computational demands and real-time feasibility are not discussed. Information on the computational time required for alignment and prediction, and whether the approach is feasible for real-time monitoring, would be relevant.

Reviewer #4

(Remarks to the Author)
This article proposes a model for the current carrying friction scenario, which is still meaningful for this scenario. The experimental design, application of neural network methods, and final result verification of the article are all quite rigorous. The overall writing and structural arrangement are also relatively reasonable, but there are still the following issues that need further consideration. Overall, this manuscript is well-written and the proposed method is clearly implemented.
1. In the current field of current carrying friction for monitoring acousto-optic electromagnetic signals, is there a method to detect the signal, or is there any problem if it is not aligned? This should be supplemented in the introduction.
Since it is aimed at the current carrying friction scenario, the title of the paper should be limited, otherwise it is unknown whether this data alignment method is applicable or its generalization performance can be extended in other scenarios.
3. The article mentions that the proposed method requires "relatively high-quality training data" to work effectively, which poses a challenge to the engineering practicality of the proposed method. It is suggested to supplement or explain this part slightly.
4. The current method seems to have no technical issues in achieving monitoring data alignment, but how economical is it? Should this be discussed in the article, such as the cost of the entire monitoring system.

Reviewer #5

(Remarks to the Author)
The manuscript introduces a neural network-based method for aligning ultra-fast optical signals (images) with one-dimensional acoustic signals, applied in the context of arc damage detection during current-carrying friction tests. The authors integrate a high-speed optical imaging system, an infrared (IR) thermal camera, and acoustic emission (AE) sensors into a tribological test rig and conduct orthogonal experiments varying load, current, and speed. The proposed alignment approach uses a deep convolutional network (a modified DenseNet-121) to determine the time offset between optical and acoustic signal streams. By feeding time-shifted acoustic data into the network and using optical-signal-derived labels, they identify the time shift that yields the highest model validation accuracy as the correct alignment parameter. Using this method, the authors report that they can align the optical and acoustic signals with ~5 ms accuracy.

Comments

• While the manuscript presents an interesting multimodal alignment framework, the overall presentation suffers from a lack of clarity. Key concepts such as the alignment challenge, deep learning workflow, and result interpretation are not introduced or developed in a systematic and reproducible manner. Figures are inconsistently labelled, and several abbreviations are used without prior definition. For improved readability and reproducibility, I strongly recommend the authors revise the structure to include: (i) a clearly defined problem statement early on; (ii) a step-by-step methodology section including model architecture, training procedure, and data labelling; and (iii) consistently labelled and explained figures with defined acronyms. Further details are provided in the specific comments below.

• The manuscript should more clearly articulate the motivation and technical challenge of aligning acoustic signals with optical/infrared images. It would help readers unfamiliar with sensor fusion to understand why alignment is necessary despite simultaneous data acquisition, and what specific temporal or physical desynchronizations this method aims to resolve.

• The manuscript lacks an explanation for why a DenseNet-121-based CNN was chosen for the alignment and classification tasks. This leaves the impression that the choice might be arbitrary. The authors should clarify why this architecture is suitable or discuss comparisons to other models. Without this, readers may question whether a simpler or traditional method could have achieved similar results. Given the novelty of the alignment idea, it would strengthen the paper to justify the use of a deep CNN.

• While the authors suggest that the optimal time shift is determined by identifying the maximum classification accuracy of a DenseNet model, the methodology is under-specified. It remains unclear how acoustic data are pre-processed into segments, how labels are assigned across shifts, and whether DenseNet is trained separately for each shift or evaluated using a sliding window strategy. Furthermore, the rationale and validity behind using peak classification accuracy as a surrogate for signal alignment require clearer justification and empirical support. I recommend the authors provide a structured description of: (i) training data generation, (ii) model input/output, (iii) shift-wise evaluation protocol, and (iv) how maximum accuracy maps to optimal alignment.

• The manuscript could be more explicit about how exactly the labels were extracted. For example, was this done by manual frame inspection or via an automated threshold algorithm? It is implied that the “unrecognized green area” in IR images was used as the arc indicator.

• It would be helpful to include a brief discussion of the computational cost. Training multiple CNNs for different shifts (or training one and sliding the input) can be computationally expensive. Is the method feasible for real-time or large-scale use?

• The paper does not explicitly state which sensor’s data was ultimately used for the alignment and detection models. One may infer that the airborne sensor was used for arc detection, as it would better capture arc sounds, but this should be clearly stated.

• Table 1 presents a well-designed set of 14 orthogonal experiments, yet only results from experiments 2-2 and 2-3 are presented and discussed. The rationale for selecting these specific experiments should be explicitly stated. Are they representative cases or particularly informative for alignment validation? If space constraints limit full reporting, a brief summary of results across all 14 conditions (e.g., in supplementary files) would enhance the transparency and reproducibility of the work.

• The terms “positive sample” and “negative sample” are used when describing the dataset and model training, but the manuscript does not explicitly define them. I infer that “positive” refers to an acoustic signal segment during which an arc was occurring, and “negative” refers to the absence of arc activity. This should be explicitly defined in the manuscript.

• Several undefined abbreviations are used in figures without definition (e.g., OS, AS, IS, AES, AE, SFT, CAM, etc.). While domain experts might infer some of these, it burdens the reader and can lead to misinterpretation. This is especially problematic in figures meant to stand on their own. All abbreviations should be defined either in the legend, the caption, or in the main text.

• The manuscript uses some vague terms that require more precise definition. For instance, the terms “relatively stationary” and “relatively non-stationary” appear as axis labels in Fig. 4, but are not introduced or explained anywhere in the manuscript.

• While the manuscript touches briefly on limitations (e.g., IR camera issues), the discussion could be expanded. It would be helpful to mention the scope and limitations of the proposed method more explicitly in the discussion or conclusion section.

• From page 5, line 7 onward, several figure references appear incorrect. For example, Fig. 2f and 2g should likely be Fig. 1f and 1g based on the surrounding discussion. Similarly, Fig. 3h (page 9, line 13) appears to be mislabeled and should be Fig. 3i. These should be corrected to ensure accurate cross-referencing.

• The vertical axes in both Figures 4a and 4b are labelled as “corresponding to no arc,” yet the text suggests that these plots include arc-related events. This inconsistency should be resolved by aligning the figure axes labels with the textual descriptions or clarifying exceptions.

• Figures 4a, 4b, and 4c contain two rows of images, but the distinction between the rows is not clearly explained in the captions or main text. This makes interpretation difficult. The authors should clarify the meaning and purpose of the two rows to improve reader understanding.

• The figure references on Page 14 are inconsistent and confusing. Subfigures labelled as Fig. 6e, 6f, 6h, and 6i are mentioned, but Fig. 6 itself does not appear to exist. Similarly, Fig. 5 is never referenced, making it unclear whether it has been omitted or misnumbered. The entire figure sequence should be checked for consistency.

Overall Assessment
The manuscript presents strong scientific content and proposes a novel method for multimodal signal alignment using deep learning. However, its clarity is hindered by structural and presentation issues. Revising key elements such as inconsistent terminology, unclear figure labelling, and missing methodological context will significantly enhance the manuscript's readability. At present, the need to interpret undefined terms or locate missing figure components detracts from the core contributions. Addressing these concerns will not only improve the manuscript's quality but also allow readers and reviewers to better engage with and appreciate the work.

Reviewer #6

(Remarks to the Author)
Signal/image alignment is an very important and challenging issue in the community of AI applications.
This manuscript attmepts to tackle the issue of aligning 1D acoustic signal and 2D images. However, a few concerns need to be addressed before its publication.

1) The big issue is lack of novelty. Section '4. Signal alignment method' should be the key part for this research work in terms of novelty. However, the main idea of the proposed method is an dedicated experiment design for data acquisition. Then a deep learning network is used to further improve the alignment accuracy of signals. The novelty is minor.

2) The manuscript title is not good reflection of the research work presented in this manuscript. For me, the main contribution of this manusript is the experimental work, and the knowledge contribution on alignment of acoustic signals and optical images is minor.

3) The writing of the manuscript needs to be improved significantly. A more structural way will help audiences to capture the key information.

Version 1:

Reviewer comments:

Reviewer #2

(Remarks to the Author)
I appreciate the author's careful and detailed responses. The authors' replies to Comments 1 and 2 are clear and sufficiently address my concerns.

However, I do not agree with the authors' response to Comment 3. In my view, the proposed method is essentially a "brute-force alignment" approach, one of the most fundamental and inefficient approaches in sequence alignment. The authors' method treats the time shift as an unknown variable and searches for the optimal shift using a neural network as a predictor. In addition, dividing the measurement data into training and test sets to evaluate performance is a similar concept to cross-validation. While the experimental results may demonstrate a certain level of practical usefulness for the specific measurement scenarios considered, I believe that the contribution is incremental. Therefore, this work should be published in an application-focused journal rather than Nature Communications.

(Remarks on code availability)

Reviewer #4

(Remarks to the Author)
The authors have clearly addressed all my concerns, I have no further comments.

(Remarks on code availability)

Reviewer #5

(Remarks to the Author)

(Remarks on code availability)
The repository provides the essential code components needed to reproduce the paper's main results, including scripts for data alignment, arc detection modeling, class activation mapping, and data preprocessing. A README file is included with hardware and software requirements clearly listed (Python 3.9.6, PyTorch 2.2.2, etc.), and the authors have linked the corresponding dataset on an external repository. The code is organized into logical folders, and a demo case is provided that allows users to quickly verify the core alignment methodology.
The installation instructions are somewhat minimal—there's no requirements.txt file provided, and the guidance essentially tells users to "use pip" without specific commands. The reliance on Sublime Text for running scripts is a bit unusual compared to standard command-line approaches. The authors note that more detailed documentation will be added after publication, which suggests the current state is preliminary.

Reviewer #6

(Remarks to the Author)
The issues raised in my previous review have been addressed.

(Remarks on code availability)

Open Access This Peer Review File is licensed under a Creative Commons Attribution 4.0 International License, which permits use, sharing, adaptation, distribution and reproduction in any medium or format, as long as you give appropriate credit to the original author(s) and the source, provide a link to the Creative Commons license, and indicate if changes were made.
In cases where reviewers are anonymous, credit should be given to 'Anonymous Referee' and the source.
The images or other third party material in this Peer Review File are included in the article's Creative Commons license, unless indicated otherwise in a credit line to the material. If material is not included in the article's Creative Commons license and your intended use is not permitted by statutory regulation or exceeds the permitted use, you will need to obtain permission directly from the copyright holder.
To view a copy of this license, visit https://creativecommons.org/licenses/by/4.0/

Response Letter

Dear reviewers,

We declare that the **submitted manuscript is the revised and resubmitted version of NCOMMS-25-26757.** Thank you very much for your review and valuable comments on our manuscript. We also appreciate the opportunity of resubmission and revision provided by the editor. As noted in the decision letter, except for reviewer 2, the editor and the other 5 reviewers suggested revisions. We made our best effort to revise our manuscript accordingly. The initial version of our response letter exceeded 200 pages and was highly detailed. Editor Gustavo Tontini highly acknowledged the effort we've dedicated to comprehensively addressing the reviewers' comments, and the Editor acknowledged the clear organization of our point-by-point response letter to reviewers. However, the Editor also pointed out that an excessively lengthy reply could hinder readability. Under the guidance of the Editor and requirements of the journal, we have condensed the response letter from 200 to 96 pages to facilitate the review process. In this revision, we have supplemented the repeated experiments on the alignment of the previous version, the alignment experiments conducted with the predetermined time shift, the datasets of optical and acoustic signals corresponding to different current-carrying friction pairs and friction modes, and the corresponding alignment experiments to demonstrate the universality of the algorithm architecture proposed in this paper. We have supplemented a comparative analysis between the cross-correlation algorithm and the algorithm architecture proposed in this paper to illustrate the advantages and application scenarios of the proposed algorithm architecture. We have supplemented the research on the impact of different models, positive and negative sample ratios, and sample sizes on alignment accuracy, based on which we have supplemented a two-level alignment strategy to reduce the computational power consumption of the alignment architecture. We have supplemented research on the impact of different preprocessing methods on eliminating optical signal domain shift. We have supplemented the estimated time costs for alignment architecture, model training, and inference, respectively. Additionally, we have supplemented the quantification of label errors and the research about the impact of the frequency range of the spectrogram on data alignment. The structure and format of the entire text have been thoroughly revised in accordance with the reviewers' suggestions and the journal's official requirements to showcase the innovative points and enhance readability clearly. The code and dataset have been uploaded in accordance with official requirements for easy review by reviewers and future use by other researchers. Finally, we have also corrected some errors discovered during the revision process and polished the full text. Twenty figures, four equations, nineteen tables and twenty-three references have also been included to support the feasibility. For your guidance, itemised responses to your comments are appended below. The "blue font" represents the modified and supplemented content in the manuscript. The "red font", "yellow marking" and "green marking" are used to assist reviewers in reviewing. **The full name of the abbreviation of the technical terms is shown in Supplementary Table 1 of the Supplementary Information.**

Contents

Response to the reviewer 1	Page 2-14
Response to the reviewer 2	Page 15-27
Response to the reviewer 3	Page 28-53
Response to the reviewer 4	Page 54-60
Response to the reviewer 5	Page 61-82
Response to the reviewer 6	Page 83-95

Reviewer #1

The manuscript requires following revisions to be incorporated:

Comment 1:

Explain why you chose to subtract and divide by min/max pixel values rather than, for example, histogram equalization or adaptive thresholding. Discuss how sensitive your alignment results are to changes in illumination or background.

Answer of comment 1:

Thanks very much for your valuable comment. Based on your comment, we have supplemented experiments with different lighting and background temperatures (datasets 3-1 and 3-3). We have adopted your suggestion, and the comparison of the generalization performance (in different lighting and background temperatures) of image detection models trained based on original images, dynamically threshold adaptive processing images, and histogram equalization processing results has been supplemented in the "Supplementary Discussion 7. Feasibility of arc detection via optical signals" section. The image processing scheme used in the previous version of the paper aimed to eliminate domain shifts in the image. However, we presently found that dynamic threshold adaptation is more suitable for optical image preprocessing in arc detection tasks. The impact of light on alignment is mainly reflected in the number of labels "1" extracted from the optical signal, which affects alignment. The study on the impact of number of samples on alignment accuracy has been supplemented in sections "2.3. Factors affecting alignment accuracy" and "Supplementary Discussion 9. Influence of the number of samples on alignment accuracy".

The supplemented contents are in "2.3. Factors affecting alignment accuracy" section are shown as:

Figs. 3d–3f illustrate the impact of the NS on alignment accuracy. When the same kernel model is employed for alignment across varying NSs, the models trained with fewer samples exhibit more pronounced underfitting. This underfitting is reflected in the reduced testing accuracy, which in turn undermines the credibility of the alignment. Therefore, the KM was trained using learning rates that increased from 2×10^{-4} at an NS of 9000 to 2×10^{-3} at an NS of 50. The learning rate is negatively correlated with the NS (Fig. 3d). The alignment accuracy deteriorates from 5 ms at an NS of 9000 to 20 ms at an NS of 50. Concurrently, the average STD of the MTA across TSs increases as the NS decreases. With decreasing NS, the kernel model in the semantic TS exhibits increased overfitting and diminished generalization capability, resulting in decreased credibility of the alignment result, as shown in Fig. 3e. As shown in Fig. 3f, as the NS decreases, the trend of peak-shaped variation in the MTA near the semantic TS becomes less pronounced. In other words, the correlation—whether positive or negative—between the MTA on either side of the semantic TS and the TS weakens as the NS decreases. The typical epoch-loss and accuracy curves of the kernel model corresponding to Fig. 3f are shown in Supplementary Fig. 23. The KM exhibits an unstable training process when the NS falls below 500. Under stable training conditions, a higher STD in the MTA across TSs reflects a greater sensitivity of accuracy to temporal variation, which is advantageous for alignment. However, in the case of unstable training, an increased STD (Fig. 2d) does not contribute to improved alignment, because the variability primarily arises from training instability rather than from meaningful temporal sensitivity. Additionally, when aligning the OS with the IS via the RDNet13 kernel and a limited NS of 50, an alignment accuracy of 15 ms was achieved, as shown in Fig. 3d. As illustrated in Fig. 1f, the arc events extracted from both the IS and OS demonstrate a temporally consistent distribution. The failed alignment between the IS and AS, as shown in Fig. 2a, is likely due to inaccuracies in the local time axis of the IS captured by the thermal imager, rather than the limited number of IS images (1800) associated with the arc. The effect of NS on alignment accuracy based on the extremely lightweight kernel model Mnet2_1 is similar to that of RDNet13, as presented in Supplementary Discussion 9. Moreover, the maximum degradation in alignment resolution within the range of 50-9000

samples is limited to just 15 ms. Therefore, in the initial stages of the large-scale search for alignment parameters, adopting a coarse alignment strategy based on a small NS and lightweight KMs is a cost-effective and practical solution.

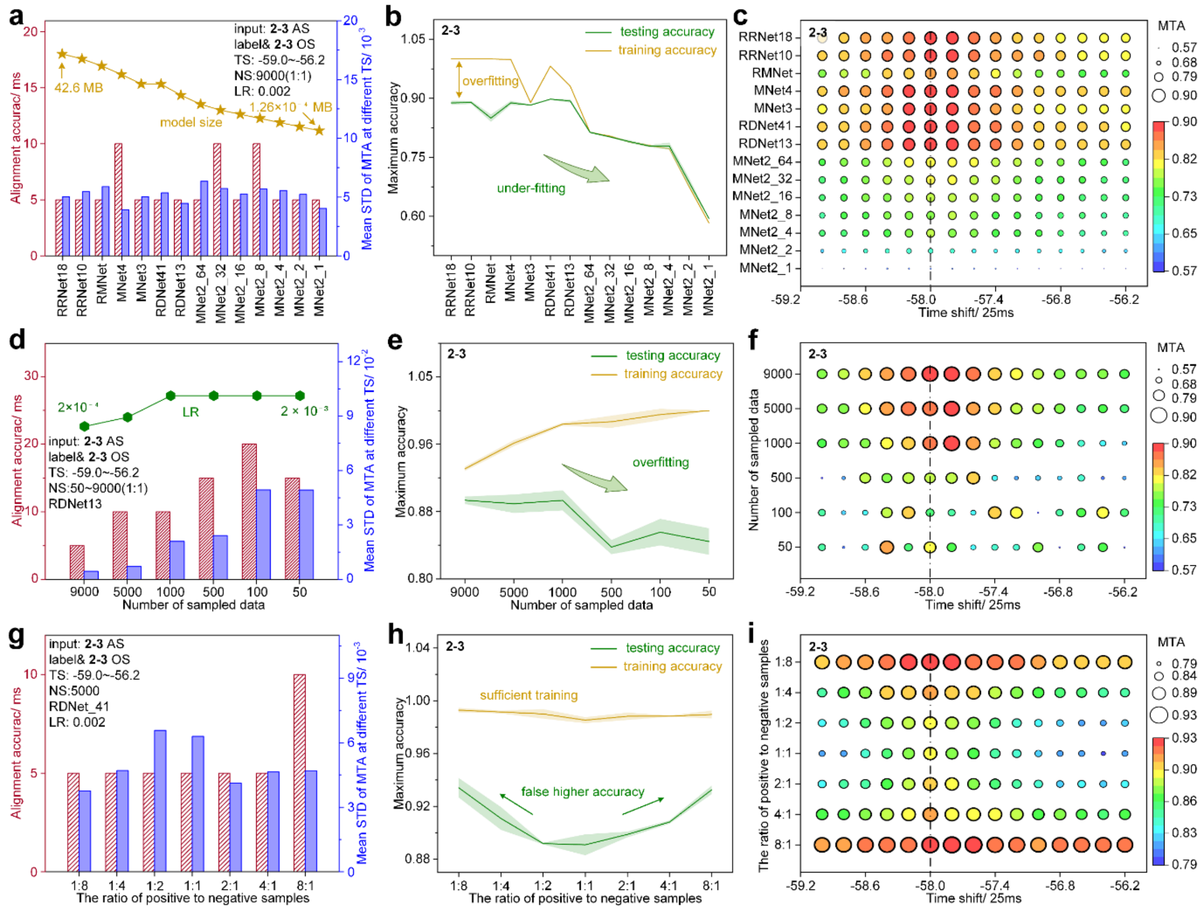

Figure 3. **Analysis of factors affecting alignment accuracy. a** Variance in alignment accuracy and standard deviation (STD) of the maximum testing accuracy (MTA) across different kernel models under the same learning rate (LR). **b** Maximum training and testing accuracy across different kernel models. **c** Bubble chart of the MTA across different kernel models and time shifts (TSs) (step size: 5 ms). **d** Alignment accuracy of the RDNet13 kernel models as the number of samples (NS) varies. **e** Corresponding NS–maximum accuracy curve. **f** Corresponding bubble chart of the MTA across different TSs (step size: 5 ms). and NS. **g** Variation in alignment accuracy with the proportion of positive to negative samples. **h** Maximum accuracy across different training sample proportions. **i** Corresponding bubble chart of MTA across different TSs (step size: 5 ms) and training sample proportions.

The supplemented and revised contents are in "Supplementary Discussion 7. Feasibility of arc detection via optical signals" section are shown as:

Line 496, Supplementary Information: Supplementary Fig. 20 shows the preprocessing results for the OS data and the performance of the corresponding arc detection models. Since OS images contain both infrared and visible spectral spectrum components, variations in light intensity and background temperature may lead to domain drift. Therefore, it is essential to explore preprocessing methods to mitigate domain shifts in OS images. Supplementary Figs. 20a-c show the original OS images from datasets **2-2** and **2-3**, the images processed via histogram equalization, and those processed via dynamic threshold adaptive methods. Supplementary Figs. 20d–20f show the epoch-loss and accuracy curves, along with the corresponding confusion matrices, for arc detection models trained on original images, images preprocessed via histogram equalization, and images preprocessed via dynamic threshold adaptive methods, respectively. Among the three models, the model

trained on dynamically thresholded images demonstrates the most stable training process and achieves the highest testing accuracy, reaching 97.2%, outperforming the other two preprocessing approaches. Supplementary Fig. 20g compares OS images from datasets from **3-1** to **3-3** and **1-1-1**, highlighting domain shifts caused by differences in background lighting and temperature. To evaluate the effectiveness of the three preprocessing methods in mitigating domain shift, the models that achieved the maximum testing accuracy on datasets **2-2** and **2-3** (Supplementary Fig. 20d: epoch 100, Supplementary Fig. 20e: epoch 62, Supplementary Fig. 20f: epoch 89) were tested on datasets from **3-1** to **3-3**. The test results, shown in Supplementary Fig. 20h, indicate that the model trained on histogram-equalized images exhibits the weakest generalization performance. In contrast, the models trained on raw images and dynamically thresholded images demonstrate comparable generalization capabilities. Although the model trained on raw images performed well in this case, its corresponding generalization performance may be less reliable when it is applied to optical images with more significant domain shifts. The arc temperature (3,000–30,000 °C), which is sufficient to melt metal, significantly differs from the background environment. For OS data collected in a darkroom, the dynamic threshold adaptive preprocessing method effectively preserves key features at the interface between the arc and the background. Moreover, the resulting binary images, composed solely of black and white pixels, help to substantially mitigate the domain shift. Accordingly, dynamic threshold adaptive preprocessing is recommended to increase the robustness of arc detection under varying imaging conditions.

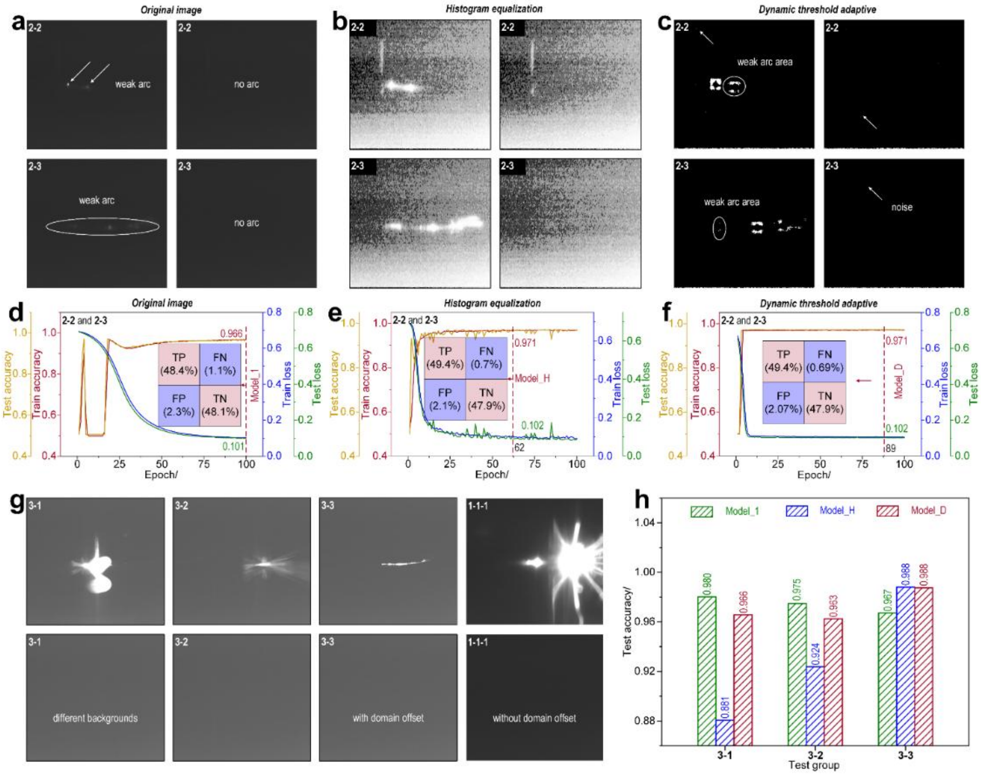

Supplementary Figure 20. **The effect of optical signal preprocessing on arc detection model performance with optical images as input**. **a** Raw images of typical optical signals from datasets **2-2** and **2-3**. **b-c** Images obtained via histogram equalization and dynamic threshold adaptive processing of these original images, respectively. **d-f** Training epoch-loss/accuracy curves of the arc detection model with the original image, the image processed via histogram equalization and the image processed via the dynamic threshold adaptive as inputs. The corresponding confusion matrix includes the true positive (TP) rate, false positive (FP) rate, true negative (TN) rate, and false negative (FN) rate. **g** Comparison of raw images in experiments between datasets from **2-2** to **3-3** and **1-1-1**. **h** Generalization performance of the model shown in d-f for image signals in datasets from **3-1** to **3-3**.

Comment 2:

Provide the specific window function w(t) you used (e.g. Hanning, Hamming), the overlap between adjacent 25 ms frames, and the FFT length—this matters for frequency resolution and alignment precision.

Answer of comment 2:

Thanks very much for your valuable comment. Based on your comment, we have supplemented related content at the end of the "Signal processing" section in the "Methods" section.

The supplemented and revised contents are in "Methods" section are shown as:

where, $x^{\mathrm{tf}}[m,k]$ denotes the time-frequency domain representation intensity of the kth frequency component in the mth frame, and where $w[n-mH]$ denotes the window function with a frame shift of H. The window function used is a Hamming window, with a 50% overlap between adjacent windows. For the conversion to the time-frequency domain representation with a frequency range of 0–1 MHz, each window corresponds to 446 points of the time-domain signal, corresponding to a time length of 0.223 ms. For the conversion with a frequency range of 0–34 kHz, each window corresponds to 2382 points of the time-domain signal, with a corresponding time length of 1.191 ms.

Comment 3:

You mention 4,832 positive optical labels vs. 2,556 infrared labels—discuss how this class imbalance might bias model training and validation. Consider augmenting underrepresented classes (e.g., via synthetic acoustic events) or using stratified sampling.

Answer of comment 3:

Thanks very much for your valuable comment. Based on your comment, we have supplemented the research on the impact of imbalanced positive and negative sample ratios on model training and alignment accuracy in the "2.3. Factors affecting alignment accuracy" section. The aforementioned study on the impact of the number of samples on the alignment accuracy of optical and acoustic signals shows that even with 50 sets of samples, an alignment accuracy of 15ms can still be achieved. Therefore, the reason for the failure of alignment between infrared and acoustic signal is the inaccurate timeline of infrared thermal imaging, which supports a maximum sampling frequency much lower than that of high-speed cameras.

The supplemented contents are in "2.3. Factors affecting alignment accuracy" section are shown as:

Figs. 3g–i illustrate the effect of the positive-to-negative sample ratio on alignment accuracy. Fig. 3g presents the alignment accuracy and the mean STD of MTA across TSs for sample ratios ranging from 1:8 to 8:1. Stratified sampling is applied to divide the training and testing sets. The alignment accuracy is 5 ms for most sample ratios; however, an increased imbalance in the sample ratio leads to a slight degradation in alignment performance. As the sample imbalance increases, the mean STD of MTA across TSs decreases, corresponding to a reduction in alignment accuracy. Fig. 3h presents the relationships between the sample ratio and the maximum training accuracy and MTA. With increasing sample imbalance, the fluctuation in maximum training accuracy remains small, which is attributable to the sufficient expressive power of the KM. As the degree of sample imbalance increases, the MTA also increases. This phenomenon arises because the loss function penalizes samples from the majority class more heavily than those from the minority class do, resulting in higher predictive accuracy for overrepresented samples than for minority-class samples. Consequently, when the majority class dominates the testing set, the overall testing accuracy may appear artificially inflated, failing to reflect actual model performance and compromising the evaluation of alignment accuracy. It is therefore advisable to maintain a positive-to-negative sample ratio as close to 1:1 as practicable. In scenarios with a limited NS, transfer learning algorithms may be employed to compensate for data scarcity.

As shown in Fig. 3i, owing to the relatively stable training process of the KM algorithm, the trend of mountain-shaped variation in accuracy remains well-preserved even under increasingly imbalanced sample ratios. However, as the sample imbalance intensifies, the difference in the MTA at different TSs diminishes, which is consistent with the trend in the STD of MTA observed in Fig. 3g. The quantitative strength of the mountain-peak trend can be characterized by a weighted mean of the rank correlation coefficients between the TSs and MTA on both sides of the maximum value of MTA. This metric serves as a direct indicator, where a larger value corresponds to a more distinct peak-shaped feature.

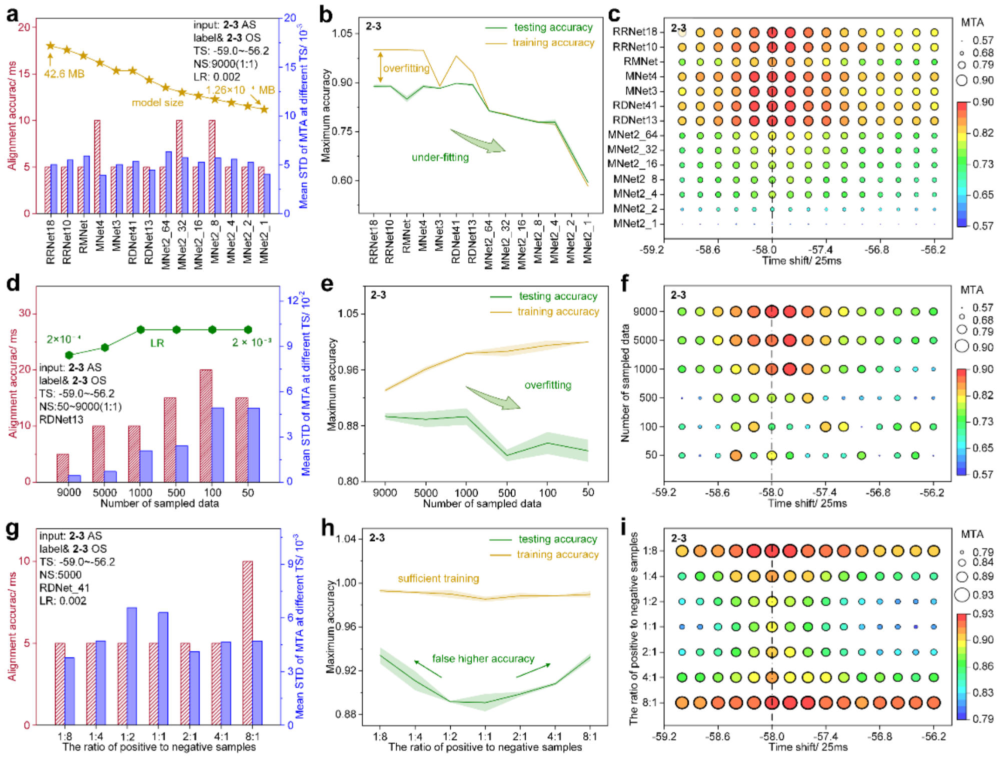

Figure 3. **Analysis of factors affecting alignment accuracy. a** Variance in alignment accuracy and standard deviation (STD) of the maximum testing accuracy (MTA) across different kernel models under the same learning rate (LR). **b** Maximum training and testing accuracy across different kernel models. **c** Bubble chart of the MTA across different kernel models and time shifts (TSs) (step size: 5 ms). **d** Alignment accuracy of the RDNet13 kernel models as the number of samples (NS) varies. **e** Corresponding NS–maximum accuracy curve. **f** Corresponding bubble chart of the MTA across different TSs (step size: 5 ms). and NS. **g** Variation in alignment accuracy with the proportion of positive to negative samples. **h** Maximum accuracy across different training sample proportions. **i** Corresponding bubble chart of MTA across different TSs (step size: 5 ms) and training sample proportions.

Comment 4:

.Report inter‑rater or intra‑rater agreement if labels were assigned by multiple annotators, to quantify label noise.

Answer of comment 4:

Thanks very much for your valuable comment. Based on your comment, we have supplemented the quantification of label noise in the "Supplementary Table 4. Label information of optical and infrared signals".

The supplemented contents are in "Supplementary Table 4. Label information of optical and infrared signals" section are shown as:

Supplementary Table 4. **Label information of optical and infrared signals**

Experiment number	Signal	Number	Labeling method	Arc response frequency
**2-2**	OS	36000	Manual annotation	256
**2-2**	IS	72000	Automatic annotation (Based on green unrecognized areas)	183
**2-3**	OS	36000	Manual annotation	4821 or 4713 (The same annotator annotates twice, inconsistency rate: 97.84%)
**2-3**	IS	72000	Automatic annotation (Based on green unrecognized areas)	2556
**3-1**	OS	36000	Manual annotation	5768
**3-2**				7652
**3-3**				1944(Inclined friction)

Line 237, Supplementary Information: The labels were annotated by the same annotator, details of the labeling criteria are provided in Supplementary Table 4.

Comment 5:

Compare your neural‑network alignment method against a classical technique (e.g., cross‑correlation or dynamic time warping) on the same 2-3 dataset. Show how much accuracy or precision is gained.

Answer of comment 5:

Thanks very much for your valuable comment. Based on your comment, we have supplemented the description and discussion of traditional cross-correlation algorithms and alignment methods based on cross-correlation supervised learning in the “2.1. Alignment requirements and architecture” section. Meanwhile, we have not yet read any heterogeneous data alignment methods is applicable to this paper scenario. Therefore, in sections “2.4. Promotion of alignment architecture” and “Supplementary Discussion 10. The alignment method based on frequency domain similarity”, we have supplemented the proposed alignment method based on frequency domain signal similarity, and a comparison between this method and the alignment architecture to highlight the advantages of the proposed architecture.

The supplemented contents are in “2.1. Alignment requirements and architecture” section are shown as: where $x_1[n,t]$ and $x_2[n,t]$ denote the nth two-dimensional time series signal with a time shift (TS) t in the randomly sampled signal segment and the corresponding one-dimensional time series signal segment, respectively. The corresponding time length of signal segments $x_1[n,t]$ and $x_2[n,t]$ is T_s. In this case, $\mathbf{A}^{n,t}$ represents an OS or IS image, and $x_2[n,t]$ is a one-dimensional AS segment with amplitude $a_s^{n,t}$ and length s. The segmentation details of the signal segment and the symbol definition rules of Equation (1) are shown in Fig. 1a. For signal alignment, cross-correlation algorithms are commonly employed, as shown in Equation (2):

$$\begin{cases} t_{\mathrm{c}} = \max_{t} \sum_{n=1}^{N} F_1^{\mathrm{tf}}(x_1[n,0]) \cdot F_2^{\mathrm{tf}}(x_2[n,t]) \\ T_{\mathrm{c}} = t_{\mathrm{c}} \cdot T_{\mathrm{s}} \end{cases} \quad (2)$$

where T_{c} and t_{c} denote the semantic TS and semantic alignment parameters, respectively, between the sequences of signal segments $x_1[n,0]$ and $x_2[n,0]$ obtained via the cross-correlation algorithm, F_1^{tf} and F_2^{tf} denote the corresponding transformation functions of $x_1[n,t]$ and $x_2[n,t]$, respectively; and the search range for semantics is $t \cdot T_{\mathrm{p}}$. However, in this case, defining F_1^{tf} and F_2^{tf} is challenging because of the unclear positive correlation between certain features of $x_1[n,t]$ and $x_2[n,t]$. Moreover, supervised learning is not applicable for determining F_1^{tf} and F_2^{tf} [46] because of the unknown and inherently ambiguous nature of the true semantic TS between $x_1[n,0]$ and $x_2[n,0]$. Addressing these challenges necessitates a robust unsupervised algorithm or architecture, the development of which demands further analytical and experimental investigation.

The supplemented contents are in "2.4. Promotion of alignment architecture" section are shown as:

Given the limited research on algorithms closely resembling the architecture proposed in this study, an alternative alignment method based on frequency domain similarity (Supplementary Discussion 10) was developed for comparative analysis. This method was evaluated further to illustrate the relative advantages of the proposed architecture. On the basis of the same alignment parameters, a comparison was conducted between the alignment results produced by the frequency domain similarity–based method and those obtained via the neural network–based architecture (Figs. 3d–f), as summarized in Fig 4a. Although the neural network–based architecture achieves slightly lower alignment accuracy (10 ms) than the frequency domain similarity–based method under the number of samples of 5,000, the architecture remains robust under data-limited conditions. In contrast, the accuracy of the frequency domain similarity–based method substantially decreases (120–165 ms) when the number of samples is reduced to 500 or less, whereas the neural network–based architecture maintains superior precision (5–20 ms). The results indicate that, although frequency domain representations of arc responses in the AS modality exhibit measurable similarity, such patterns are not prominent in small-sample analyses and require large datasets for reliable detection. The computational costs of the respective alignment methods are reported in Supplementary Tables 17 and 18. The evolution of numerical modeling has undergone three major paradigm shifts. The first stage is defined by numerical fitting methods, such as least squares, which rely on predefined basis functions. The second stage introduces traditional machine learning algorithms, including support vector machines (SVMs), which eliminate the need for explicit basis functions but operate within limited solution spaces. The third and current stage is characterized by deep learning algorithms, which are initiated by models from the AlexNet era and are capable of learning complex patterns directly from data. Compared with earlier modeling paradigms, the training of deep learning models offers significantly expanded paths for identifying optimal solutions. Using AlexNet as a representative example, the process begins with weight initialization, followed by progressive feature transformation stages that enrich representation through dimensional expansion and spatial reduction of feature maps. Hierarchical connectivity across layers further enhances the expressive capacity of the model, enabling the learning of increasingly abstract features. During training, loss functions guide parameter updates by reinforcing features that improve task performance, thereby systematically directing the model toward accurate classification or regression outcomes. The neural network–based alignment architecture reflects the characteristics of the third modeling paradigm, which involves solving for the target kernel model (KM) through multiple adaptive strategies involving feature aggregation and enrichment, thereby achieving high-quality alignment. In contrast, the frequency domain similarity–based algorithm resembles the paradigms of

the first and second stages, employing relatively fixed rules that rely solely on frequency-domain vector similarities. This fundamental limitation explains its significantly lower accuracy than neural network–based alignment when working with a limited number of samples. However, algorithms that rely on relatively fixed approaches empowered by relevant domain knowledge to find solution functions are still recommended because they consume relatively low algorithmic costs. However, in the absence of such prior knowledge, neural networks offer clear advantages. Deep learning models with strong expressive capacity can flexibly adapt to end-to-end learning across diverse data types, including tabular data, images, semantic vectors, and time series. The primary trade-offs of neural network–based approaches include increased demand for data and computational resources. Nonetheless, when domain knowledge is unavailable, the cost associated with acquiring large-scale data and supporting sufficient computational power becomes a necessary investment.

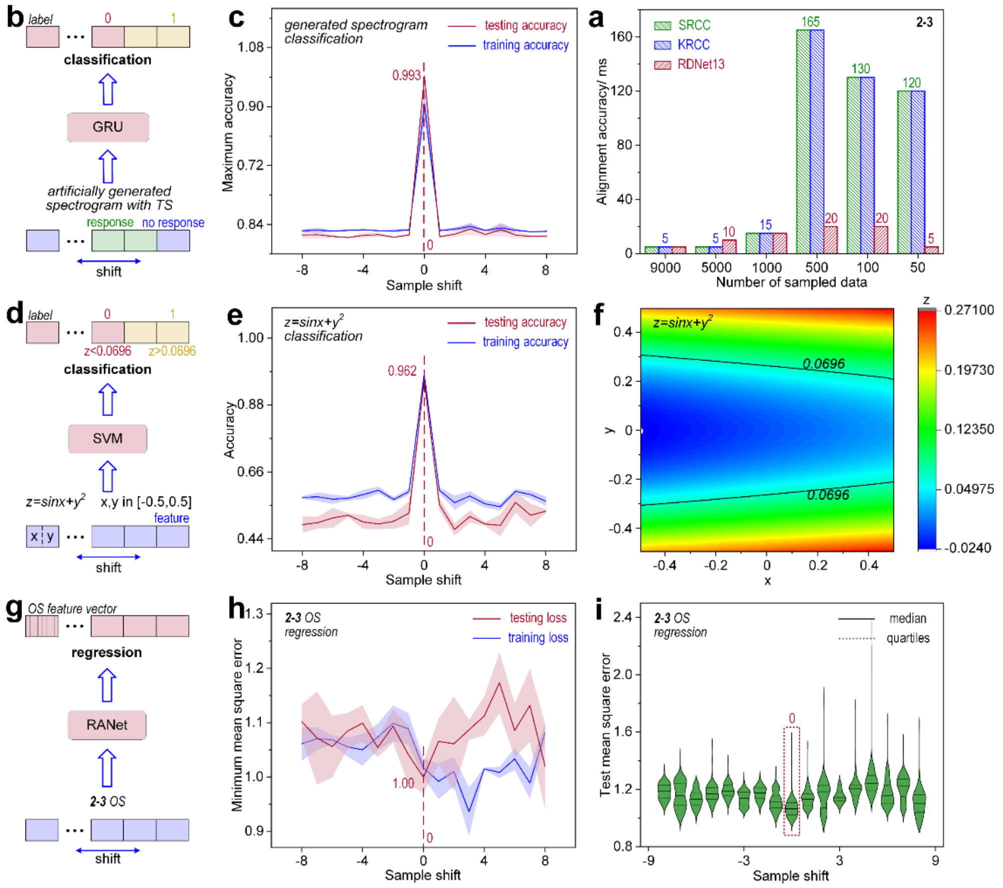

Figure 4. **Analysis of alignment architecture promotion.** **a** Comparison of alignment accuracy between the RDNet13 kernel model and frequency domain similarity, using the Spearman rank correlation coefficient (SRCC) or the Kendall rank correlation coefficient(KRCC).

The supplemented contents are in “Supplementary Discussion 10. The alignment method based on frequency domain similarity” section are shown as:

The implementation and results of the frequency domain similarity-based alignment method are shown in Supplementary Figs. 26a–c. To address the challenge of defining explicit correlation functions between heterogeneous data modalities, an approach leveraging intramodal similarity was proposed under the assumption that spectrograms corresponding to the same label category—such as arc response and no arc response—exhibit inherent similarity. As shown in Supplementary Fig. 26a, for the optical signal (OS) modality, similarity is quantified by computing the coincidence accuracy between the OS label sequence and the offset OS label sequence. For the acoustic signal (AS) modality, the time-domain signal of each segment

is first transformed into the frequency domain. To ensure comparability, the amplitude of the frequency-domain signal is scaled such that the sum of the amplitudes remains constant. Inspired by the geometric principle that the square in a rectangle with equal perimeter yields the maximum area, the similarity between two signals is quantified via the dot product between the scaled frequency-domain vectors, where an increasing dot product denotes greater similarity between two signals. For the AS segment sequence, the original AS sequence corresponds to the time shift, whereas each offset AS sequence corresponds to the superposition of the time shift and offset. The offset-label coincidence accuracy curve, derived from the OS sequence, and the offset-frequency domain similarity curve, obtained from the AS sequence across different time shifts, are shown in Supplementary Fig. 26b. Suppose that a given time shift accurately reflects accurate semantic alignment. In that case, the positive correlation between the label coincidence accuracy and frequency domain similarity at that time shift should be stronger than that observed at other time shifts. The mathematical formulation of this algorithm is shown in Supplementary Equation (1):

$$\left\{\begin{aligned} S_y[t_{\mathrm{of}}] &= \sum\nolimits_{n=1}^{N} M(y_1[n,0], y_1[n,t_{\mathrm{of}}])/N \\ S_{\mathrm{fft}}[t_{of},t] &= \sum\nolimits_{n=1}^{N} k\cdot \mathbf{F}_{2,\mathrm{norm}}^{\mathrm{fft}}[n,t] \bullet \mathbf{F}_{2,\mathrm{norm}}^{\mathrm{fft}}[n,t_{\mathrm{of}}+t] \\ c_{\mathrm{Sr}}[t] &= 1-\frac{6\cdot\sum_{t_{\mathrm{of}}=-T_{\mathrm{of}}}^{T_{\mathrm{of}}} d_n^2(S_y[t_{\mathrm{of}}],S_{\mathrm{fft}}[t_{\mathrm{of}},t])}{N^3-N} \\ c_{\mathrm{Kr}}[t] &= \frac{C(S_y[t_{\mathrm{of}}],S_{\mathrm{fft}}[t_{\mathrm{of}},t])-D(S_y[t_{\mathrm{of}}],S_{\mathrm{fft}}[t_{\mathrm{of}},t]}{\frac{1}{2}N\cdot(N-1)} \\ t_{\mathrm{Sr}} &= \max_t c_{\mathrm{Sr}}[t] \\ t_{\mathrm{Kr}} &= \max_t c_{\mathrm{Kr}}[t] \\ T_{\mathrm{Sr}} &= t_{\mathrm{Sr}}\cdot T_p \\ T_{\mathrm{Kr}} &= t_{\mathrm{Kr}}\cdot T_p \end{aligned}\right\} t_{\mathrm{of}}\in \mathrm{Z},\ y_1[n,t]\in\{0,1\} \qquad (1)$$

where $y_1[n,0]$, $S_y[t_{\mathrm{of}}]$, $\mathbf{F}_{2,\mathrm{norm}}^{\mathrm{fft}}[n,t]$, and $S_{\mathrm{fft}}[t_{of},t]$ denote the label obtained from signal $x_1[n,0]$, the label coincidence accuracy between sequences $\{y_1[0,0], y_1[1,0],\cdots, y_1[N-1,0]\}$ and sequences $\{y_1[0,t_{\mathrm{of}}], y_1[1,t_{\mathrm{of}}],\cdots, y_1[N-1,t_{\mathrm{of}}]\}$, the scaled frequency-domain vectors of signal $x_2[n,t]$ (where the sum of the elements of each vector is equal), and the frequency-domain similarity between sequences $\{\mathbf{F}_{2,\mathrm{norm}}^{\mathrm{fft}}(0,t),\ \mathbf{F}_{2,\mathrm{norm}}^{\mathrm{fft}}(1,t),\ \cdots,\ \mathbf{F}_{2,\mathrm{norm}}^{\mathrm{fft}}(N-1,t)\}$ and $\{\mathbf{F}_{2,\mathrm{norm}}^{\mathrm{fft}}(0,t+t_{\mathrm{of}}),\ \mathbf{F}_{2,\mathrm{norm}}^{\mathrm{fft}}(1,t+t_{\mathrm{of}}),\ \cdots,\ \mathbf{F}_{2,\mathrm{norm}}^{\mathrm{fft}}(N-1,t+t_{\mathrm{of}})\}$, respectively. $c_{\mathrm{Sr}}[t]$ and $c_{\mathrm{Kr}}[t]$ denote the Spearman rank coefficient and the Kendall rank coefficient, respectively. d^n denotes the rank difference between the matching degree sequences $\{S_y[-T_{\mathrm{of}}],\ S_y[-T_{\mathrm{of}}+1],\ \cdots,\ S_y[T_{\mathrm{of}}]\}$ and the similarity sequence $\{S_{\mathrm{fft}}[-T_{\mathrm{of}},t],\ S_{\mathrm{fft}}[-T_{\mathrm{of}}+1,t],\ \ldots,\ S_{\mathrm{fft}}[T_{\mathrm{of}},t]\}$. $C(S_y[t_{\mathrm{of}}],S_{\mathrm{fft}}[t_{\mathrm{of}},t])$ and $D(S_y[t_{\mathrm{of}}],S_{\mathrm{fft}}[t_{\mathrm{of}},t])$ denote the logarithms of the consistent and inconsistent elements between sequences $\{S_y[-T_{\mathrm{of}}],\ S_y[-T_{\mathrm{of}}+1],\ \cdots,\ S_y[T_{\mathrm{of}}]\}$ and $\{S_{\mathrm{fft}}[-T_{\mathrm{of}},t],\ S_{\mathrm{fft}}[-T_{\mathrm{of}}+1,t],\ \ldots,\ S_{\mathrm{fft}}[T_{\mathrm{of}},t]\}$, respectively. Here, T_{of} is set to 8. t_{Sr} and t_{Kr} denote the semantic alignment parameters between the semantic sequences of signals $x_1[n,0]$ and $x_2[n,0]$ calculated on the basis of the Spearman rank correlation coefficient and the Kendall rank correlation coefficient, respectively. T_{Sr} and T_{Kr} are the corresponding semantic time shifts, and the search range of the semantic time shift is $\mathrm{t}\cdot T_p$. The pseudocode corresponding to the above algorithm is shown in Algorithm 2.

The sampling methods and ratios employed in Supplementary Figs. 26b-c are consistent with those utilized in Figs. 3g-i, with 9,000 samples analyzed for the former. The time shift–level correlation coefficient curve generated by the proposed algorithm is presented in Supplementary Fig. 26c. Both the Spearman and the Kendall rank correlation coefficients reach their extrema at a time shift of -58.0×25 ms, indicating that the semantic time shift t_o^a is -58.0 × 25 ms with an alignment accuracy of 5 ms. This estimated semantic time shift lies within the expected reference range and is consistent with the outcome derived from the proposed neural network-based alignment architecture. Furthermore, large-scale data analysis confirms that

spectrograms exhibit substantial similarity within the arc response category, whereas those that display clear distinctions between arc and no-arc responses.

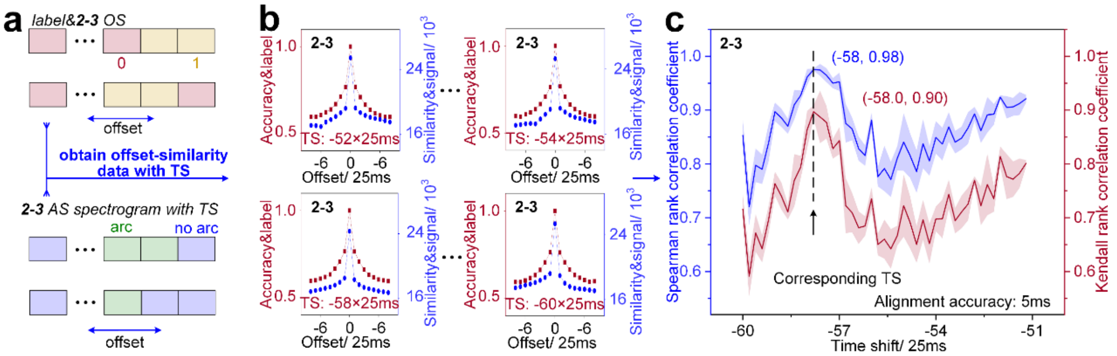

Supplementary Figure 26. **The alignment method on the basis of frequency domain similarity. a** Implement schematic of the alignment method. **b** Offset-accuracy and similarity curves under various time shifts (TSs). **c** Alignment results based on the method between optical signals (OS) and acoustic signals (AS).

Comment 6:

Provide timing benchmarks (training/inference cost) so readers can assess practicality for high - throughput in-situ tests.

Answer of comment 6:

Thanks very much for your valuable comment. Based on your comment, we have supplemented the analysis of the training inference time for the arc detection model in the "Supplementary Discussion 8. Cost evaluation of the arc detection model" section.

The supplemented contents are in “Supplementary Discussion 8. Cost evaluation of the arc detection model” section are shown as:

To assess the feasibility of deploying the arc detection model based on optical signals (OSs), acoustic signals (ISs), or acoustic signals (ASs) on an in-situ testing system for real-time monitoring, the inference time was measured via Python implementation, as detailed in Supplementary Table 16. The total inference time comprises two parts: data preprocessing and model inference. For arc detection using OS as input, the total time required for dynamic threshold adaptation preprocessing and inference with the RSNet model across 36,000 samples is 88.77 seconds. In contrast, IS-based arc detection involves neither preprocessing nor deep learning model inference, resulting in a substantially lower computational cost than the OS-based approach. For AS-based arc detection via the RDNet41 model, the total processing time for 36,000 samples is 893.8 s. All three detection methods exhibit inference times that are shorter than the corresponding test duration of 900 s, thereby fulfilling the real-time requirements for arc discrimination in the in-situ testing system. Moreover, for deployment scenarios demanding greater efficiency, implementing the models in “C” language can further reduce the computational cost of arc detection. Additionally, high-quality in-situ detection modules are costly. This case is expected to simultaneously equip high-speed cameras, infrared thermal imagers, and acoustic emission sensors with the ability to generate high-throughput statistics of the arc damage frequency under current-carried friction conditions, which would enhance the flexibility and utilization efficiency of in-situ detection modules in material data factories.

Supplementary Table 14. **The time cost of signal preprocessing**

(Not included in the time for data reading, data storage, and data segmentation)

Data preprocessing method	Time cost for processing each signal segment/s
Convert AS from time domain into the 1 M spectrogram form	3.453×10^{-2}
Convert AS from time domain into the 34 kHz spectrogram form	2.434×10^{-2}
Convert AS from time domain into the 1 MHz frequency domain form	4.156×10^{-3}
Convert OS based on the dynamic threshold adaptive	1.347×10^{-3}
Convert OS based on the histogram equalization	5.155×10^{-3}

Supplementary Table 15. **Training time costs of machine learning models**

(Not included in the time for data reading, data storage, and data segmentation)

Model	Samples number	Epoch	Training time per epoch/ s	Testing time per epoch/ s	Total training time/s
GRU	9000	200	4.157	4.034	1638
MLP		100	9.855×10^{-3}	3.621×10^{-2}	13.48
MNet2_1			8.225	3.619	1184
MNet2_2			8.532	3.755	1229
MNet2_4			8.411	3.645	1206
MNet2_8			8.623	3.710	1233
MNet2_16			8.364	3.566	1193
MNet2_32			8.650	3.660	1231
MNet2_64			8.999	4.124	1312
MNet3			9.190	3.954	1314
MNet4			9.109	4.006	1312
RANet		50	64.35	26.59	4547
RDNet13		100	8.878	3.732	1261
RDNet41			9.848	4.391	1424
RMNet			9.881	4.105	1399
RRNet10			11.16	4.683	1584
RRNet18			8.921	3.728	1265
RSNet (3 channels input)	10134	100	72.24	29.89	10213
RSNet (1 channels input)			28.08	11.34	3942
SVM	600	/	/	/	1.557×10^{-2}

Supplementary Table 16. **The inference time costs for the deployed model to detect the arc**

(Inference on the basis of Supplementary Tables 14 and 15)

Input signal	Model	Samples number	Data preprocessing time/ s	Inference time of model/ s	Total inference time/s
AS	MNet2_1	36000	876.2	14.48	890.7
AS	MNet2_2			15.02	891.3
AS	MNet2_4			14.58	890.8
AS	MNet2_8			14.84	891.1
AS	MNet2_16			14.26	890.5
AS	MNet2_32			14.64	890.9
AS	MNet2_64			16.50	892.7
AS	MNet3			15.82	892.1
AS	MNet4			16.02	892.3
AS	RDNet13			14.93	891.2
AS	RDNet41			17.56	893.8
AS	RMNet			16.42	892.7
AS	RRNet10			18.73	895.0
AS	RRNet18			14.88	891.1
OS	RSNet (3 channels input)		0 or 185.6	106.2	106.0 or 291.8
OS	RSNet (1 channels input)		48.49	40.28	88.77

Comment 7:

You use a simplified DenseNet-121 for shift estimation. Include an ablation that:

Varies network depth (e.g., DenseBlock counts).

Swaps in a lightweight CNN (e.g., MobileNet or ResNet-18) to show that DenseNet's dense connections are necessary. Report how alignment precision (in ms) degrades with smaller models.

Answer of comment 7:

Thanks very much for your valuable comment. Based on your comment, we have supplemented the impact of different kernel neural networks on alignment accuracy in the "2.3. Factors affecting alignment accuracy" sections. These networks include RDNet-41, RDNet13 modified from DenseNet-121, the self-developed M-Net series models without dense connectivity layers, RMNet modified from MobileNet and RRNet18 modified from ResNet18, etc.

The supplemented contents are in "2.3. Factors affecting alignment accuracy" section are shown as:

In systems with a constant semantic time shift (TS), synchronous hardware triggering is applied to ensure the simultaneous acquisition of heterogeneous signals. Simultaneously, the TS can be estimated once during an initial calibration using the alignment architecture and subsequently reused, resulting in minimal computational cost. In contrast, in systems with a varying semantic time shift—such as those affected by time-varying delays—the alignment architecture must be further optimized to reduce the computational cost. Fig. 3 illustrates the alignment accuracy between OS and AS in dataset **2-3**, evaluated across different kernel models (KMs), the number of samples (NS), and positive-to-negative

sample ratios. Fig. 3a presents the alignment accuracy for various KM sizes, including RRNet18 and RRNet10, with KM sizes ranging from 42.6 MB to 1.26×10⁻⁴ MB. The smallest model (MNet2_1) consists of only two layers and a single channel. As the KM size decreases, the alignment accuracy fluctuates under time resolutions of 5 ms and 10 ms, with a higher frequency of occurrence at 5 ms (Fig. 3a). Contrary to the intuitive expectation that the standard deviation (STD) of maximum testing accuracy (MTA) across different TSs systematically increases with reduced KM size, no consistent negative correlation between these two variables is observed. Fig. 3b shows the mean of the maximum training accuracy or the MTA across the KMs. Reducing the KM size corresponds to a clear transition from overfitting to underfitting. Despite the emergence of underfitting, the alignment accuracy remains unaffected. The corresponding bubble plot in Fig. 3c illustrates the relationships among the KMs, TS, and MTA. For each model, the MTA initially increases with TS, reaches a peak near the semantic TS, and subsequently decreases. Notably, this peak-like extremum remains pronounced even as the KM size decreases, demonstrating that alignment accuracy remains significantly unaffected by the KM size. The overall training process maintains relative stability despite potential underfitting tendencies in small kernel models, as corroborated by the epoch-accuracy and loss curves in Supplementary Fig. 22.

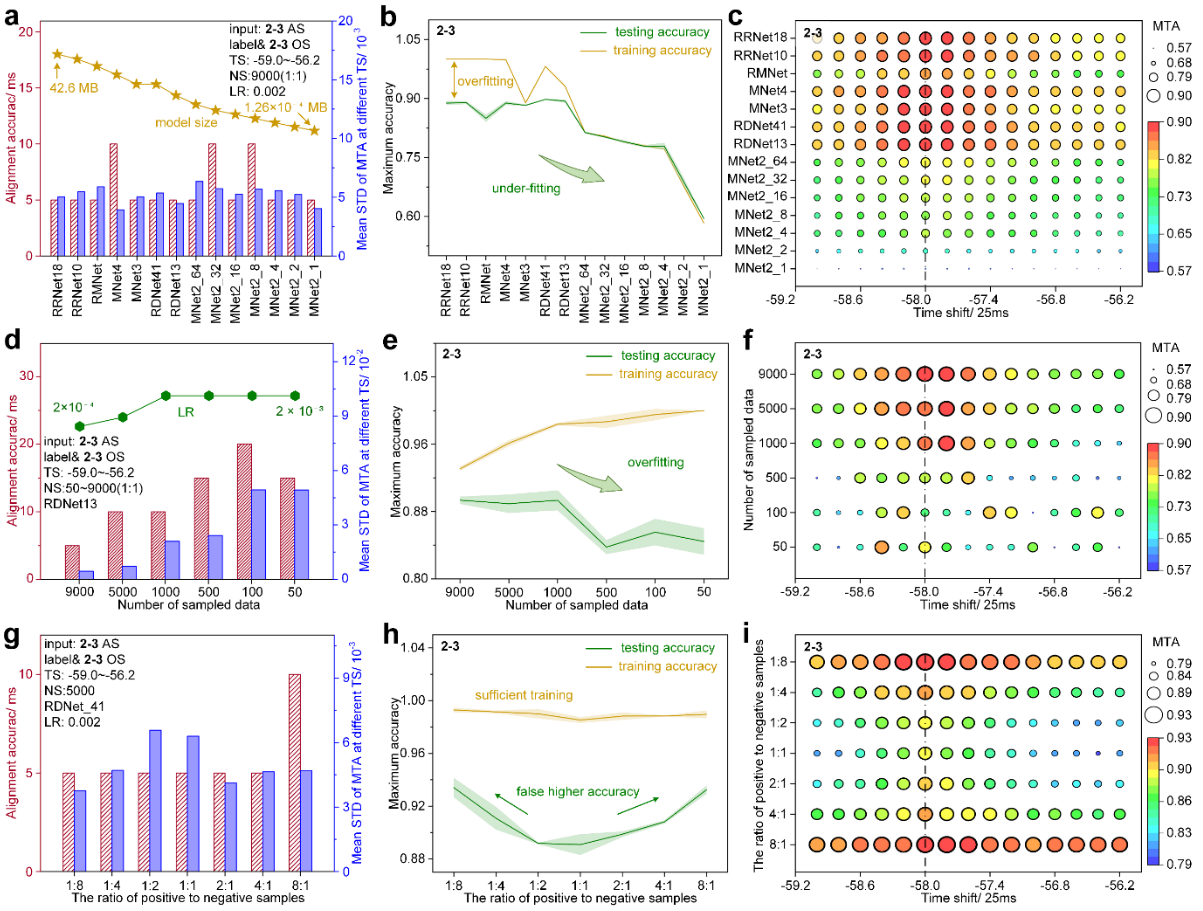

Figure 3. **Analysis of factors affecting alignment accuracy.** **a** Variance in alignment accuracy and standard deviation (STD) of the maximum testing accuracy (MTA) across different kernel models under the same learning rate (LR). **b** Maximum training and testing accuracy across different kernel models. **c** Bubble chart of the MTA across different kernel models and time shifts (TSs) (step size: 5 ms). **d** Alignment accuracy of the RDNet13 kernel models as the number of samples (NS) varies. **e** Corresponding NS–maximum accuracy curve. **f** Corresponding bubble chart of the MTA across different TSs (step size: 5 ms). and NS. **g** Variation in alignment accuracy with the proportion of positive to negative samples. **h** Maximum accuracy across different training sample proportions. **i** Corresponding bubble chart of MTA across different TSs (step size: 5 ms) and training sample proportions.

Reviewer #2

The manuscript, "Neural network-driven alignment of ultra-fast acoustic and optical signals," introduces a temporal alignment method for multimodal signals, including optical images, infrared images, and acoustic signals. The authors claim that the proposed method achieves 5 ms alignment accuracy and 90% discrimination accuracy. Although the proposed method may work appropriately for the specific experimental setup presented in the paper, the impact and significance of the manuscript are limited for the reasons below. Therefore, my recommendation is to reject the manuscript.

Comment 1:

The authors propose a temporal alignment method meticulously crafted for a measurement system they built in [3]. However, timing synchronization across different measurement signals can, in general, be easily achieved using hardware triggers, which are versatile for off-the-shelf measurement devices. It seems that the authors raise an unnecessary problem by not synchronizing the sensors in hardware and trying to solve it. Although it would be effective for their specific measurement system in which the sensors are not synchronized, I doubt the necessity and significance of the proposed method in a general sense.

Answer of comment 1:

Thanks very much for your valuable comment. Based on your comment, we have revised and supplemented the argument that the semantics of acoustic signals lag behind the semantics of synchronous optical signals in the "2.2. Alignment verification and applications" section and "Supplementary Discussion 5. Interpretability analysis of the acoustic signals" section. We have supplemented the limitation of cross-correlation and supervised learning algorithms based on cross-correlation algorithms in the "2.1. Alignment requirements and architecture" section to indicate the limitations of cross-correlation algorithms. And we also made corresponding revisions "abstract" section of the paper. The response to the generality of the proposed architecture is in section "**Answer of comment 2**".

The revised contents are in "Abstract" section are shown as:

Via cross-correlation algorithms or synchronized acquisition of signals, the alignment of heterogeneous data with unknown semantic time shifts and intermittent semantic variations cannot be solved. The shift is caused by different data acquisition principles of sensors, different response discrimination principles using heterogeneous data, etc.

The revised contents are in "1. Introduction" section are shown as:

Line 43, Revised Manuscript: Similarly, the goal of temporal signal alignment is to ensure semantic consistency between corresponding segments across signals. While hardware-based synchronization is the preferred approach, the differing acquisition principles of heterogeneous sensors and the varying discrimination based on heterogeneous data often limit its applicability. In such cases, cross-correlation analysis [43-45], is considered. However, cross-correlation analysis often requires prior domain knowledge, such as understanding the positive or negative correlation between signal features. To enhance applicability, supervised learning has been employed to derive transformation functions that generate effective features for alignment [46]. A significant limitation of this approach is the reliance on manually annotated semantic time shifts between signals as the gold standard for training[46]. Aligning heterogeneous data without domain knowledge is challenging [47], especially when semantic time shifts are unknown and semantic variations are transient [48, 49]. First, the unknown semantic time shifts hinder the extraction of manually annotated semantic time shifts and effective alignment features [46]. Furthermore, transient semantic variations further hinder the establishment of associations between heterogeneous data. Consequently, a robust and unsupervised alignment

method is urgently needed to overcome the associated challenges. Given the successful use of neural networks for alignment in transfer learning and multimodal domains [27–32, 37–42], a critical research question arises: can a novel neural network-driven architecture be designed to address the challenges of heterogeneous data alignment with unknown semantic time shifts and intermittent semantic variation?

The supplemented contents are in "2.1. Alignment requirements and architecture" section are shown as: where $x_1[n,t]$ and $x_2[n,t]$ denote the nth two-dimensional time series signal with a time shift (TS) t in the randomly sampled signal segment and the corresponding one-dimensional time series signal segment, respectively. The corresponding time length of signal segments $x_1[n,t]$ and $x_2[n,t]$ is T_s. In this case, $\mathbf{A}^{n,t}$ represents an OS or IS image, and $x_2[n,t]$ is a one-dimensional AS segment with amplitude $a_s^{n,t}$ and length s. The segmentation details of the signal segment and the symbol definition rules of Equation (1) are shown in Fig. 1a. For signal alignment, cross-correlation algorithms are commonly employed, as shown in Equation (2):

$$\begin{cases} t_c = \max_t \sum_{n=1}^{N} F_1^{\mathrm{tf}}(x_1[n,0]) \cdot F_2^{\mathrm{tf}}(x_2[n,t]) \\ T_c = t_c \cdot T_s \end{cases} \tag{2}$$

where T_c and t_c denote the semantic TS and semantic alignment parameters, respectively, between the sequences of signal segments $x_1[n,0]$ and $x_2[n,0]$ obtained via the cross-correlation algorithm, F_1^{tf} and F_2^{tf} denote the corresponding transformation functions of $x_1[n,t]$ and $x_2[n,t]$, respectively; and the search range for semantics is $t \cdot T_p$. However, in this case, defining F_1^{tf} and F_2^{tf} is challenging because of the unclear positive correlation between certain features of $x_1[n,t]$ and $x_2[n,t]$. Moreover, supervised learning is not applicable for determining F_1^{tf} and F_2^{tf} [46] because of the unknown and inherently ambiguous nature of the true semantic TS between $x_1[n,0]$ and $x_2[n,0]$. Addressing these challenges necessitates a robust unsupervised algorithm or architecture, the development of which demands further analytical and experimental investigation.

The supplemented contents are in "2.2. Alignment verification and applications" section are shown as: Line 225, Revised Manuscript: Moreover, interpretability analysis was performed via the arc detection model with AS as the input (Fig. 2e), as detailed in Supplementary Discussion 5. The interpretability analysis results and the comparative analysis between the reference time shift range and the semantic TS in alignment between OS and AS suggest that the semantics of AS lag behind synchronized OS semantics for arc detection tasks, which underscores the value of the proposed alignment architecture. The effectiveness of arc detection on the basis of the IS or AS was demonstrated on the basis of the effectiveness of the OS. Therefore, the effectiveness of arc detection via OS is verified in Supplementary Discussion 7. The training and inference costs of the arc detection models based on OS, IS, or AS are discussed in Supplementary Discussion 8.

The supplemented and revised contents are in "Supplementary Discussion 5. Interpretability analysis of the acoustic signals" section are shown as:

To analyze the principle of arc detection via acoustic signals (ASs), category activation mapping (CAM) [14] was performed from the last convolutional layer of the kernel model corresponding to the 53rd epoch in Fig. 2e, as shown in Supplementary Figs. 17a-b. The CAM is related to both the activation value and gradient, and the regions with significant CAM values indicate that the regions of the input image significantly affect the class discrimination of the model. In regions with relatively significant CAM values, those corresponding to no arc damage were more extensive than those corresponding to arc damage. Therefore, the arc damage may be closely related to the local features of the spectrogram, whereas the absence of arc damage is more closely

associated with the global features. The distribution of significant regions in the arc damage was related to the longitudinal texture of the spectrogram, although not entirely. In contrast, the significant areas corresponding to arc damage and no arc damage did not appear to be strongly related to frequency, unlike the clear correspondence between the arc response of the arc igniter and its corresponding spectrogram in Supplementary Fig. 9d. The arc damage in current carrying friction seems to be related to the spectrogram response in the range of 8-34 kHz, whereas the corresponding spectrogram without arc damage shows a relatively strong response in the range of 8-22 kHz. More CAM cloud maps are provided in Supplementary Movie 4 and Supplementary Movie 5. Owing to the inherent limitations of human perception, memory, and information processing, the brain, guided by evolutionary attention mechanisms, is best suited for recognizing high-contrast features, such as vertical stripes in spectrograms. Discriminating arc damage depends on coupling relationships in the AS spectrogram, which are difficult for the brain to identify. However, these complex and subtle patterns can be effectively recognized and accurately characterized by neural networks with strong expressive power. Although CAM does not provide researchers with particularly explicit domain knowledge, the proposed alignment architecture helps separate AS corresponding to arc damage from those without arc damage, which is challenging to accomplish solely through the brain. Supplementary Fig. 17c shows the normalized spectrogram corresponding to the AS in Supplementary Fig. 18, which corresponds to arc damage and slight friction(current: 8A). Supplementary Fig. 18 illustrates the energy variation when the power is switched on and off under the constant displacement loading mode. The micrometer head was rotated to slightly separate the current-carried friction pair. Initially, the power was switched off (0 A), then switched on (8 A), and finally, the power was turned off again (0 A). The variations in the voltage signal in the figure reflect this process. When voltage was applied to the friction pair, an increase in voltage fluctuation indicated arc damage. In contrast, an increase in the normal load was associated with the formation of minor surface protrusions, likely originating from transferred material, which contributed to elevated friction. Supplementary Fig. 17c presents representative spectrograms recorded under a current load of 8 A, including both time-stable patterns and those exhibiting pronounced temporal fluctuations, The relatively fluctuating spectrogram in Supplementary Fig. 17c exhibits a lower fluctuation frequency along the time axis than that in Supplementary Fig. 17a does, which may correspond to a higher frequency of collisions and frictional interactions between small surface protrusions under the constant minor load loading mode than under the slight friction condition (current: 8A) corresponding to Supplementary Fig. 18. Accordingly, the presence of vertical textures in the spectrograms corresponds to arc-induced damage on the surface of the friction pair. In contrast, the relatively temporal stable spectrograms observed in Supplementary Figs. 17b and 17c exhibit similar features, which may be indicative of friction-related damage. Moreover, in the arc generation, repeated collision and solidification of metals result in the tiny protrusions between microscopic protrusions on the friction pair surfaces, inducing unstable friction and significant fluctuations in the AS spectrogram (Supplementary Fig. 17a). In contrast, under situations without arc damage, tiny protrusions have been peeled off or flattened during friction, resulting in fine frictional contact, which effectively suppresses system fluctuations and results in a more stable AS spectrogram (Supplementary Fig.17b) along the time axis. Moreover, in this case, the delay between the OS and AS, caused by the inherent difference in the propagation speeds of light and sound, is approximately 0.5 ms, which has only a minor influence on the overall system. Consequently, the arc detection model with AS as input performs inference on the basis of the AS recorded after the arc event has occurred. This observation is consistent with the earlier finding that the semantics of AS tend to lag behind those of synchronous OS in the alignment process mentioned in Supplementary Discussion 4 (in Lines 368-371 of Supplementary Information), further indicating the reliability of the above statement and the significance of the proposed signal alignment architecture. As shown in the confusion matrix (Fig. 2f) of the arc detection

model based on the AS input, the false positive (FP) and false negative (FN) rates are relatively comparable. Since the labels for training the model are derived from the aligned OS, the FN rate may result from arc events that cause sufficiently strong acoustic responses detectable by the acoustic emission sensor, but are missed by the high-speed camera owing to its visual blind spots. In contrast, the FP rate may be attributed to arc events with weak or ambiguous acoustic signatures that are captured by the high-speed camera but fail to elicit a detectable response in the acoustic emission sensor.

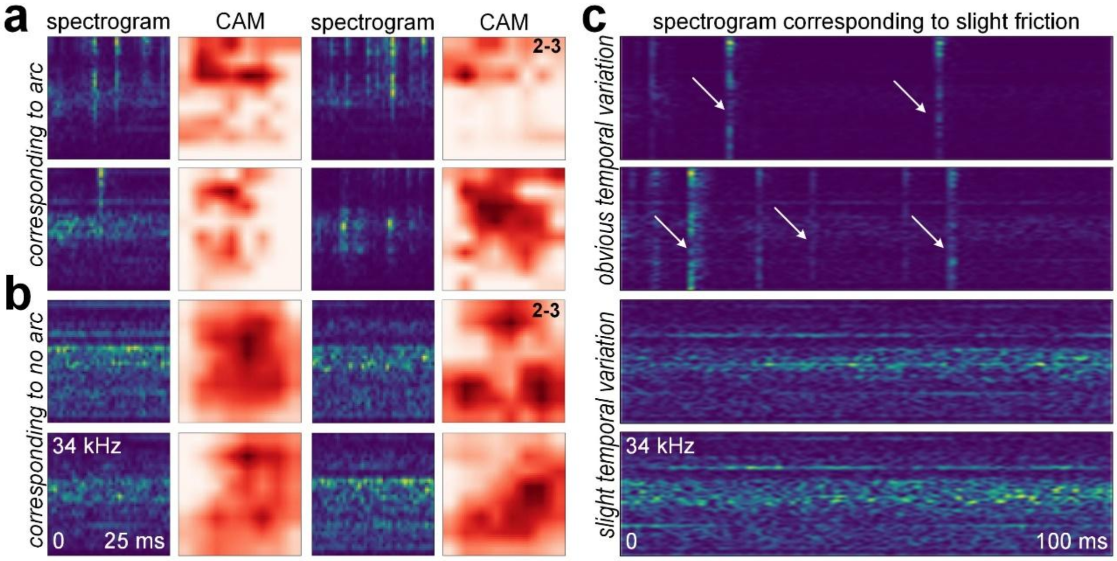

Supplementary Figure 17. **Interpretability analysis of typical acoustic signals. a-b** Normalized spectrograms of the acoustic signals selected via the signal alignment method and the calculated class activation maps (CAMs) under conditions of arc damage and no arc damage. **c** Typical normalized spectrogram of the acoustic signals under the condition of slight contact (current: 8 A) between two friction specimens (Supplementary Fig. 16.). (a, b and c show multiple sets of spectrograms with the same semantics to more fully demonstrate the common features of the signal.)

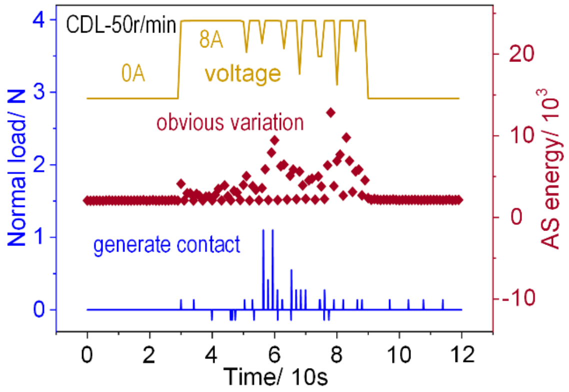

Supplementary Figure 18. **The variations in the acoustic signal (AS) energy and normal load with varying electrification conditions, occurring in the case of slight separation of the friction pair under the constant displacement loading (CDL) mode.**

Comment 2:

This paper demonstrates the effectiveness of the proposed method only for the one measurement system. There is no discussion about how this method could be used for other systems, so the method is missing generality. If the proposed method is only valid for the specific measurement system, it may not be appropriate for publication in Nature Communications.

Answer of comment 2:

Thanks very much for your valuable comment. Based on your comment, we have revised and supplemented datasets 3-3 for optical and acoustic signals corresponding to arc detection under inclined friction conditions, as well as datasets 3-1 and 3-2 for optical and acoustic signals corresponding to arc detection under different current carrying friction pair material conditions in "Supplementary Discussion 2. Datasets used for the alignment study" section. And corresponding alignment experiments were supplemented based on these datasets in the "Supplementary Discussion 4. Repetitive and supplementary alignment" section. We supplemented the alignment experiment of the dataset based on the preset time shift in the"2.4. Promotion of alignment architecture" section and the "Supplementary Discussion 11. Supplement of alignment architecture promotion" section.

The supplemented contents are in "2.4. Promotion of alignment architecture" section are shown as:

To further evaluate the applicability and reliability of the proposed alignment architecture, extensive experiments were performed on datasets with predetermined alignment parameters. For time series data, to accelerate the experimental process, both the AS spectrograms and the corresponding labels were synthetically generated on the basis of the arc response patterns of the arc igniter. The construction procedure is illustrated in Supplementary Fig. 27, and the detailed algorithm for generating data is provided in Supplementary Algorithm 3. On the basis of the gated recurrent unit (GRU) KM, alignment experiments were conducted on the generated spectrograms and corresponding labels (Fig. 4b), with the sample shift search range set from -8 to +8. In this setup, the frequency response intensity vectors at each time point in the spectrogram were used as feature vectors input into the time series model. The alignment results are shown in Fig. 4c, and the distribution of testing accuracy across different sample shifts is presented in Supplementary Fig. 28. Both the training accuracies and testing accuracies reached their maximum values at a sample shift of 0, indicating that the alignment architecture successfully achieved correct alignment between the time series data and the corresponding labels. To further validate the applicability of the proposed alignment architecture to tabular data, a synthetic dataset was constructed, referring to recent research [52], via the function $z = \sin x + y^2$, where x and y were sampled within the range of -0.5 --+0.5. Data points with $z > 0.0696$ were assigned a label of 1, whereas those with $z \leq 0.0696$ were assigned a label of 0 (Fig. 4d). The feature vectors composed of x and y, and the corresponding labels, were aligned via a support vector machine (SVM) KM (Fig. 4d). The alignment results shown in Fig. 4e demonstrate the favorable alignment performance of the architecture. In the multimodal era driven by transformer -based architectures, semantic vectors have become the primary medium for cross-modal information exchange. To evaluate the applicability of the proposed alignment framework in such contexts, alignment was performed between dataset **2-3** OS images and their corresponding 8-dimensional semantic feature vectors (mean, maximum, minimum, range, variance, skewness, kurtosis, and entropy) via the RANet KM (Fig. 4g). The alignment results are presented in Figs. 4h and 4i. While the training mean squared error (MSE) did not reach its minimum at a sample shift of 0, the testing MSE achieved its lowest value at this point. Moreover, the overall distribution of the testing MSE at a sample shift of 0 was lower than that at other shifts, indicating successful alignment between the OS images and their associated semantic representations. The sample shift–testing MSE curves corresponding to the three

alignment trials (Figs. 4g–i) are presented in Supplementary Fig. 29. Among the three, one instance exhibited suboptimal alignment performance, with an alignment resolution exceeding 8 sample shifts. This discrepancy may be attributed to internal balancing mechanisms among the outputs of the multioutput neural network. To increase the robustness of alignment in such challenging scenarios, multiple alignment runs are recommended to increase the reliability of the estimated semantic time shift. Additionally, supplementary alignment experiments are presented in Supplementary Discussion 11, including the alignment between images and labels, tabular data and labels, and input vectors and output values of artificially constructed functions. All the experiments achieved favorable alignment performance. In summary, at the task level, the proposed alignment architecture is applicable not only to regression tasks of KMs but also to classification tasks. At the model level, the architecture accommodates a wide range of model architectures, including traditional machine learning models, time series models, and convolutional neural networks. At the data level, the architecture supports alignment across diverse modalities, including tabular data, time series data, and image data, with corresponding semantic labels or feature vectors. Therefore, this alignment architecture is expected to be applicable to align heterogeneous data pairs in which one modality allows the extraction of semantic labels, semantic vectors, and continuous semantic values. Finally, the time cost of alignment based on the proposed architecture is analyzed in detail in Supplementary Discussion 12.

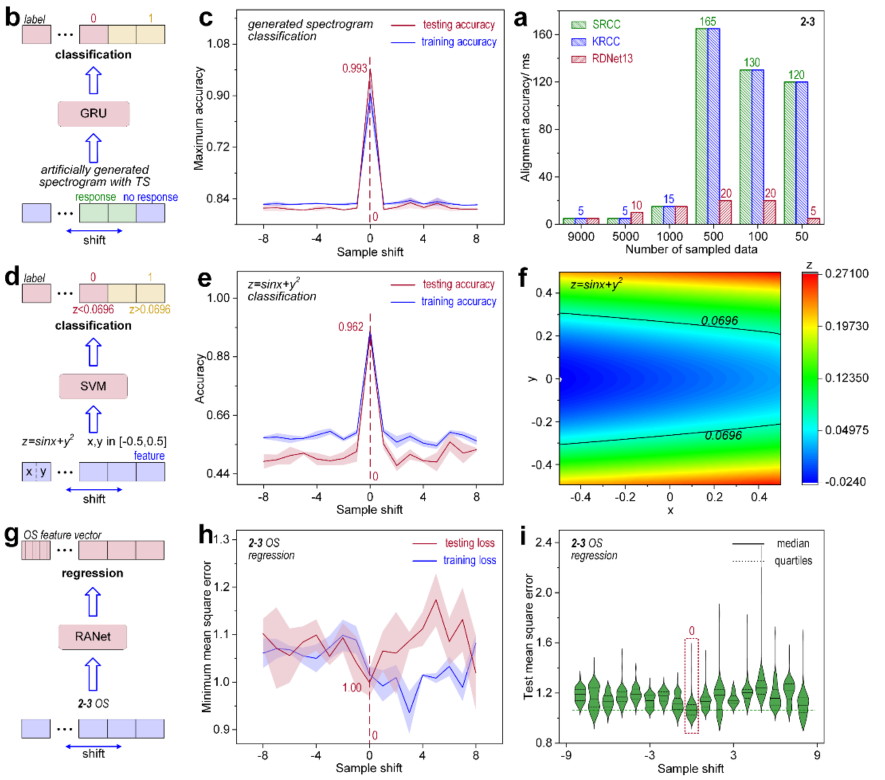

Figure 4. **Analysis of alignment architecture promotion. a** Comparison of alignment accuracy between the RDNet13 kernel model and frequency domain similarity, using the Spearman rank correlation coefficient (SRCC) or the Kendall rank correlation coefficient(KRCC). **b-c** Schematic diagrams and alignment results based on the gate recurrent unit (GRU) kernel model used to align the arc response data from the arc igniter with the corresponding labels. **d-e** Schematic diagrams and alignment results of aligning the input of artificially constructed functions and corresponding labels on the basis of the support vector machine (SVM) kernel model. **f** Cloud diagram of the corresponding constructed functions. **g-h** Schematic diagram and alignment results of aligning optical signals (OS) in dataset **2-3** and feature vectors of the OS via the RANet kernel model. **i** The corresponding distribution of testing accuracy across different sample shifts.

The supplemented contents are in "Supplementary Discussion 2. Datasets used for the alignment study" section are shown as:

Supplementary Table 3. **The dataset numbering and corresponding conditions of the orthogonal experiments**

Number	Monitor signal	Material	Load mode	Electric current / A	Rotational speed/ r/min	Time/ s
**1-1-1**	OS	CrZrCu alloy	CDL	2	50	1000
**1-1-2**	OS	CrZrCu alloy		2	150	
**1-1-3**	OS	CrZrCu alloy		8	50	
**1-1-4**	OS	CrZrCu alloy		8	150	
**1-2-1**	OS	CrZrCu alloy	CLL	2	50	
**1-2-2**	OS	CrZrCu alloy		2	150	
**1-2-3**	OS	CrZrCu alloy		8	50	
**1-2-4**	OS	CrZrCu alloy		8	150	
**1-3-1**	OS	CrZrCu alloy	CMLL	2	50	
**1-3-2**	OS	CrZrCu alloy		2	150	
**1-3-3**	OS	CrZrCu alloy		8	50	
**1-3-4**	OS	CrZrCu alloy		8	150	
**2-2**	OS,IS and AS	CrZrCu alloy	CLL	2、5、8	50、100、150	900
**2-3**	OS,IS and AS	CrZrCu alloy	CMLL			
**3-1**	OS and AS	CuBe alloy				
**3-2**	OS and AS	CuSn alloy				
**3-3**	OS and AS	CuZn alloy				

Line 180, Supplementary Information: To further assess the generalizability of the proposed architecture, the disc materials in experiments from **3-1** to **3-3** were replaced with CuBe, CuSn, and CuZn alloys.

Line 210, Supplementary Information: This discrepancy arises because the ASs were more sensitive to subtle variations and fluctuations in the friction load, whereas the low friction speed resulted in less pronounced variation between consecutive infrared frames. Four additional experiments were conducted on the basis of a more transient process to further support signal alignment. The experiments involved metal columnar projectiles impacting metal plates at a launch speed of approximately 58 m/s. The impact produced clear acoustic, optical, and infrared responses, allowing for effective cross-modal alignment, as shown in Supplementary Fig. 9c. Via the impact experiments, reference ranges for the TS among the OA, AS, and IS in datesets **2-2** and **2-3** were determined. The TS t_i^a between the AS and IS ranged from -59.50×25 ms to -54.25×25 ms, whereas the TS t_o^a between the AS and OS ranged from -60.00×25 ms to -52.50×25 ms, which indicated that the TS between the signals consisted of a major systematic error and a minor random error. In addition to impact response testing, the photoacoustic transient response event of the arc generated by the arc igniter can also serve as a reference for signal alignment. The corresponding acoustic response is shown in Supplementary Fig. 9d, where the spectrogram exhibits a pronounced signal in the 17–19 Hz range. According to seven arc response tests, the TS t_o^a between OSs and ASs in datasets from **3-1** to **3-3** was determined to be within the range of -48.00×25 ms to -52.00×25 ms. Although each experiment could be approximately synchronized on the basis of other physical events such as impacts, microexplosions, or arc responses, the alignment parameters derived from these auxiliary events may not completely coincide with the semantic alignment parameters between signals of the target response.

[REDACTED]

Supplementary Figure 9. **Reference time-shift range between the signals of the testing system. a** Discrimination criteria based on infrared signals (OSs) to determine whether the friction disc is rotating. **b** Spectrogram of the acoustic signals (AS) corresponding to the start and stop of the friction disk rotation. **c** Approximate range of the time shift between OS, infrared signals (IS) and AS for datasets **2-2** and **2-3** of the experiments, obtained from the impact test. **d** Approximate range of the time shift of the system signal for datasets from **3-1** to **3-3** of the experiments, obtained from the arc igniter test.

Supplementary Table 4. **Label information of optical and infrared signals**

Experiment number	Signal	Number	Labeling method	Arc response frequency
**2-2**	OS	36000	Manual annotation	256
**2-2**	IS	72000	Automatic annotation (Based on green unrecognized areas)	183
**2-3**	OS	36000	Manual annotation	4821 or 4713 (The same annotator annotates twice, inconsistency rate: 97.84%)
**2-3**	IS	72000	Automatic annotation (Based on green unrecognized areas)	2556
**3-1**	OS	36000	Manual annotation	5768
**3-2**				7652
**3-3**				1944(Inclined friction)

With respect to in-situ signal processing, semantic labels were assigned to the OS from datasets from **2-2** to **3-3** via manual annotation, and to the ISs from dataset **2-3** via automated annotation. The labels were annotated by the same annotator details of the labeling criteria are provided in Supplementary Table 4.

The supplemented contents are in “Supplementary Discussion 4. Repetitive and supplementary alignment” section are shown as:

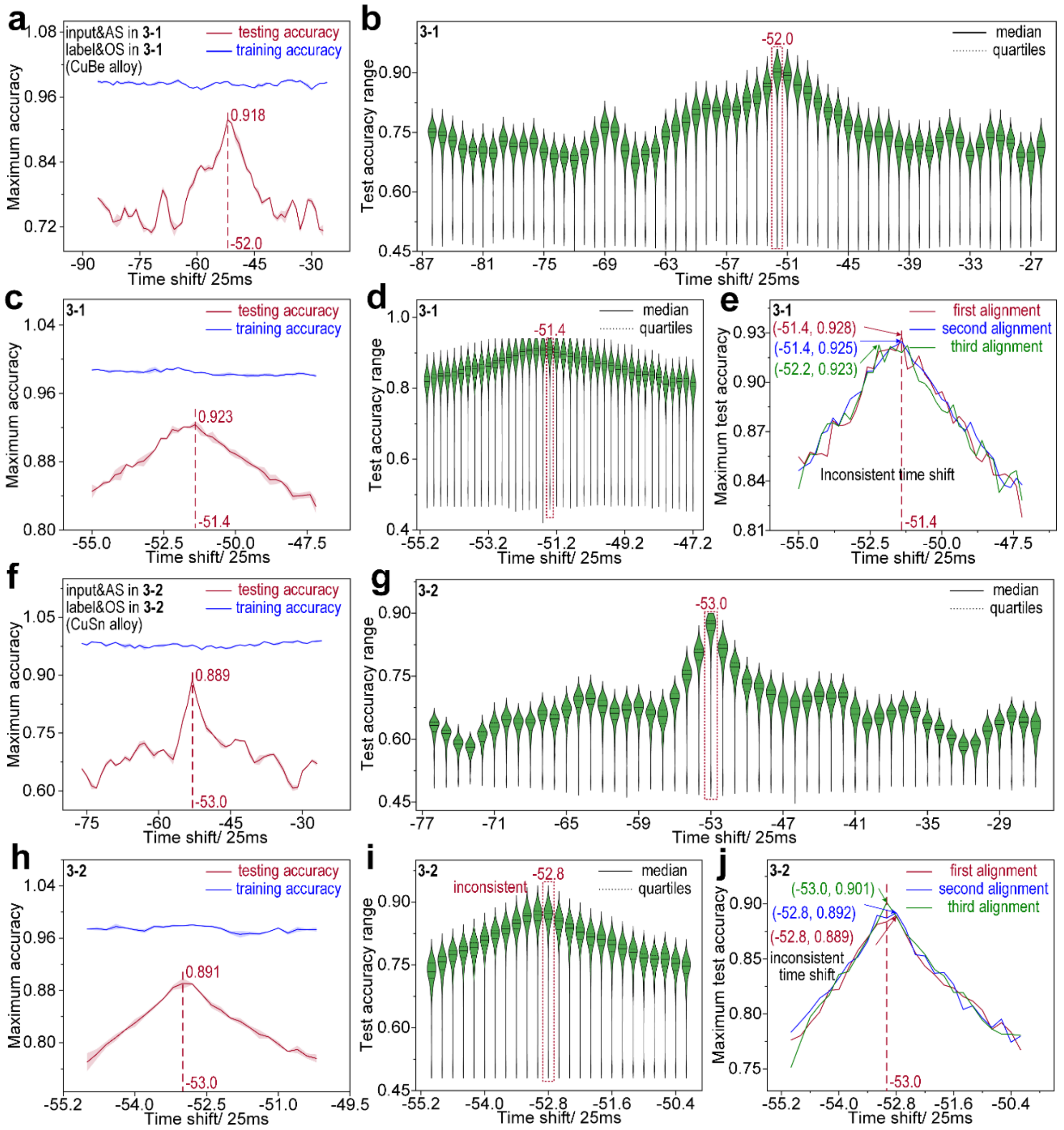

Supplementary Figure 15. **The time shift-maximum accuracy curve and accuracy distribution corresponding to the alignment of optical signals (OS) and acoustic signals (AS) in datasets 3-1 and 3-2. a-b** The time shift (step size: 25 ms)-maximum accuracy curve and time shift (step size: 25 ms)-testing accuracy distribution for dataset **3-1** alignment, respectively. **c-d** Time shift (step size: 5 ms)-maximum accuracy curve and time shift (step size: 5 ms)-testing accuracy distributions in the dataset **3-1** alignment, respectively. **e** Time shift–maximum testing accuracy curve corresponding to 3 repeated alignments in dataset **3-1**. **f-g** Time shift (step size: 25 ms)-maximum accuracy curve and time shift (step size: 25 ms)-testing accuracy distributions for dataset **3-2** alignments. **h-i** Time shift (step size: 5 ms)-maximum accuracy curve and time shift (step size: 5 ms)-testing accuracy distributions in the dataset **3-2** alignment, respectively. **j** Time shift–maximum testing accuracy curve corresponding to 3 repeated alignments in dataset **3-2**.

Line 360, Supplementary Information: To further demonstrate the effectiveness of the proposed architecture, alignment between the optical signal (OS) and acoustic signal (AS) from experiments with different current-carried friction pairs (**3–1** and **3–2**) and friction modes (**3–3**) was conducted. The friction pairs in dataset **3–1** are 7075 Al alloy and CuBe alloy, with the alignment results shown in Supplementary Figs. 15a–e. The semantic TS t_o^a for this dataset is -51.4×25 ms. In the dataset **3–2** , the friction pairs are 7075 Al alloy and CuSn alloy, with the alignment results presented in Supplementary Figs. 15f–j, and the semantic TS t_o^a is -53.0×25 ms.The alignment results of OS and AS for dataset **3-3** are shown in Supplementary Figs. 16c–h, with an alignment parameter t_o^a of -49.2×25 ms. For datasets from **3-1** to **3-3**, the obtained alignment parameter is close to the reference range from -51.00×25 ms to -49.00×25 ms derived from the photoacoustic response test. Notably, in addition to the alignment of dataset **3-3** (inclined friction in Supplementary Fig. 16a), the comparison of the alignment results from datasets **2-3**, **3-1**, and **3-2** in the four vertical friction tests (Supplementary Fig. 16b) with the corresponding reference range of TSs suggests that the semantics of the AS tend to lag behind those of the synchronous OS. Therefore, the acoustic response following the arc response may serve as a basis for detecting the arc response via the AS.

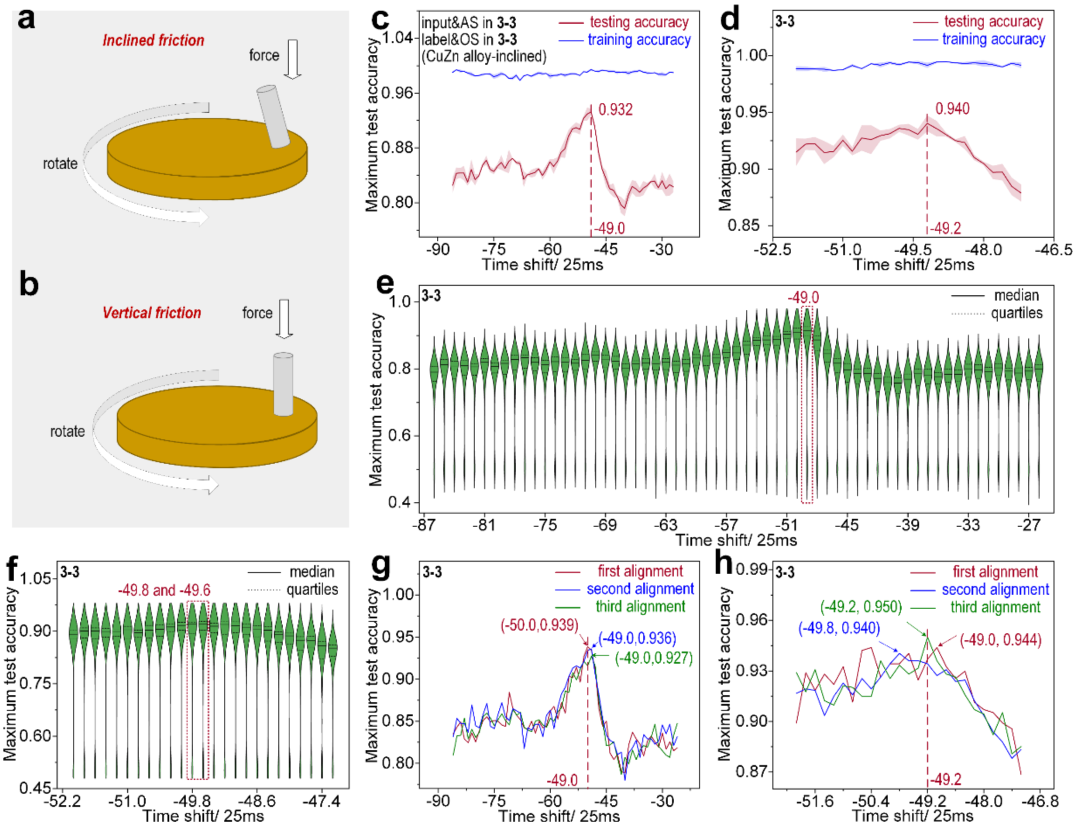

Supplementary Figure 16. **Friction form of the test specimens and alignment results of the optical signals (OS) and acoustic signals (AS) in dataset 3-3 of the experiments. a** Schematic diagram of inclined friction in dataset **3-3**. **b** Schematic diagram of vertical friction in other datasets. **c-d** Time shift (time step: 25 or 5 ms)-maximum accuracy curve for **3-3** sets of alignments. **e-f** Time shift (step: 25 or 5 ms)-testing accuracy distributions for **3-3** sets of alignments. **g-h** Time shift (step: 25 or 5 ms)-maximum testing accuracy curve corresponding to 3 repeated alignments in dataset **3-3**.

The supplemented contents are in "Supplementary Discussion 11. Supplement of alignment architecture promotion" section are shown as:

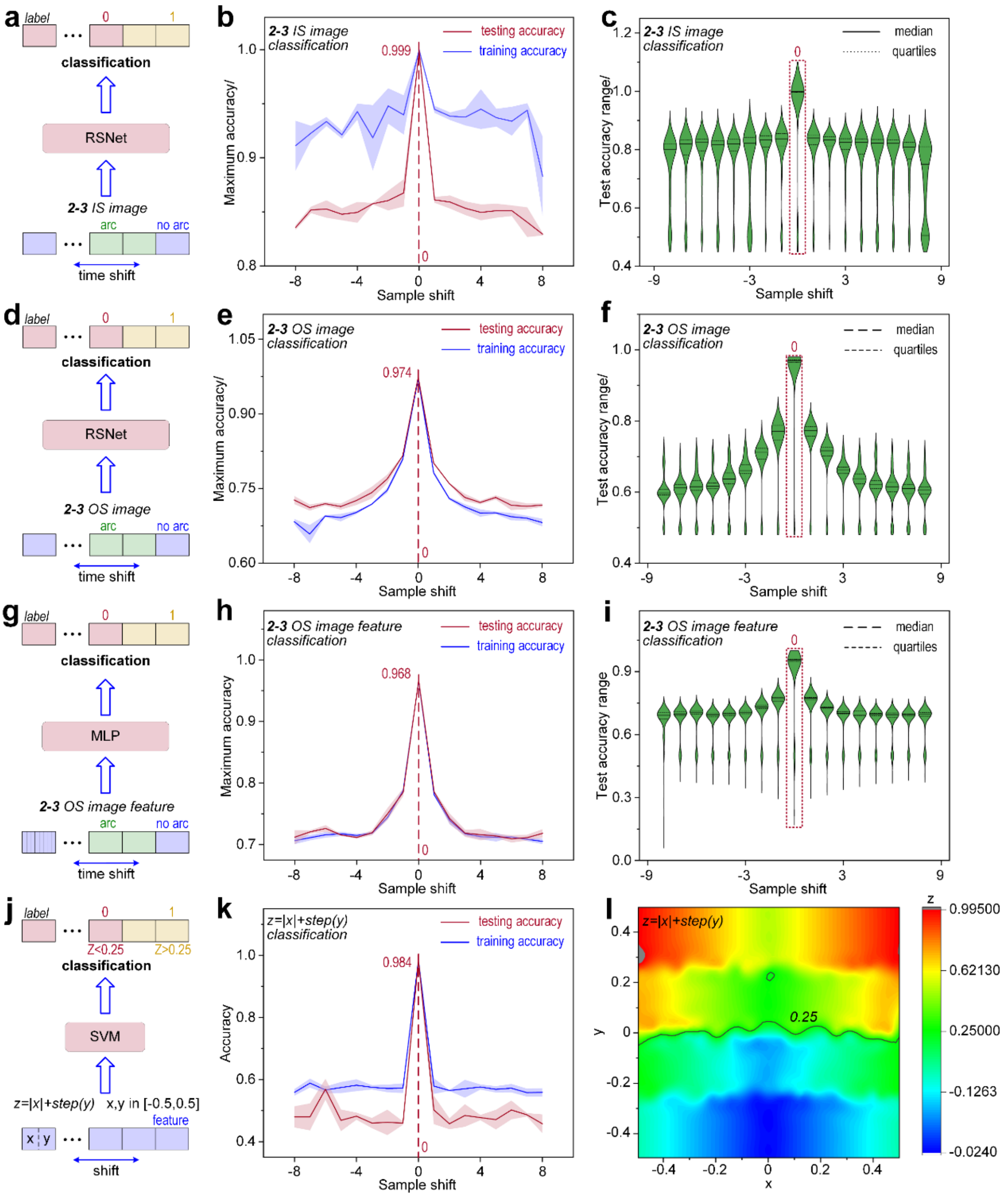

Supplementary Figure 30. **Promotion of the alignment architecture (supplements for Figure 4). a** Schematic diagram of aligning dataset **2-3** infrared signals (IS) with their labels via the RSNet kernel model. **b-c** Sample shift-maximum accuracy curve and testing accuracy distribution corresponding to a, respectively. **d** Schematic diagram of aligning dataset **2-3** optical signals (OS) with their labels via the RSNet kernel model. **e-f** Sample shift-maximum accuracy curves and accuracy distributions corresponding to d, respectively. **g** Schematic diagram of the alignment of dataset **2-3** feature vectors of the OS with their labels via multilayer perceptron (MLP) kernel model. **h-i** The shift-maximum accuracy curve and testing accuracy distribution corresponding to g, respectively. **j-k** Schematic diagram of aligning the input of artificially constructed functions with the corresponding output via the support vector machine (SVM) kernel model and corresponding alignment results. **l** Cloud diagram of the constructed functions in j.

For image data, alignment via the RSNet kernel model was performed between infrared signal images and corresponding labels in dataset **2-3** (Supplementary Figs. 30a-c), as well as between optical signal (OS) images and their corresponding labels in dataset **2-3** (Supplementary Figs. 30d-f), both of which achieved favorable alignment results. For tabular data, eight statistical features—mean, maximum, minimum, range, variance, skewness, kurtosis, and entropy—were extracted from dataset **2-3** OS images to form 8-dimensional feature vectors. The alignment between these vectors and their corresponding labels was performed via a multilayer perceptron (MLP) kernel model (Supplementary Figs. 30g–i). The model achieved maximum training and testing accuracy at a sample shift of 0, confirming the suitability of the proposed alignment architecture for structured tabular data. Additionally, the input vectors of the function $z = |x| + \text{step}(y)$ and corresponding labels based on the support vector machine (SVM) were successfully aligned (Supplementary Figs. 30j-l). Subsequently, alignments between the input vectors and outputs of the above two functions were performed via a regression-based kernel model (Supplementary Fig. 31). Both models receive a two-dimensional feature vector composed of x and y as inputs and predict the corresponding z values. Both the training and testing mean squared errors reached their minimum at a sample shift of 0 for the two models, demonstrating that the proposed alignment architecture is also applicable to regression tasks.

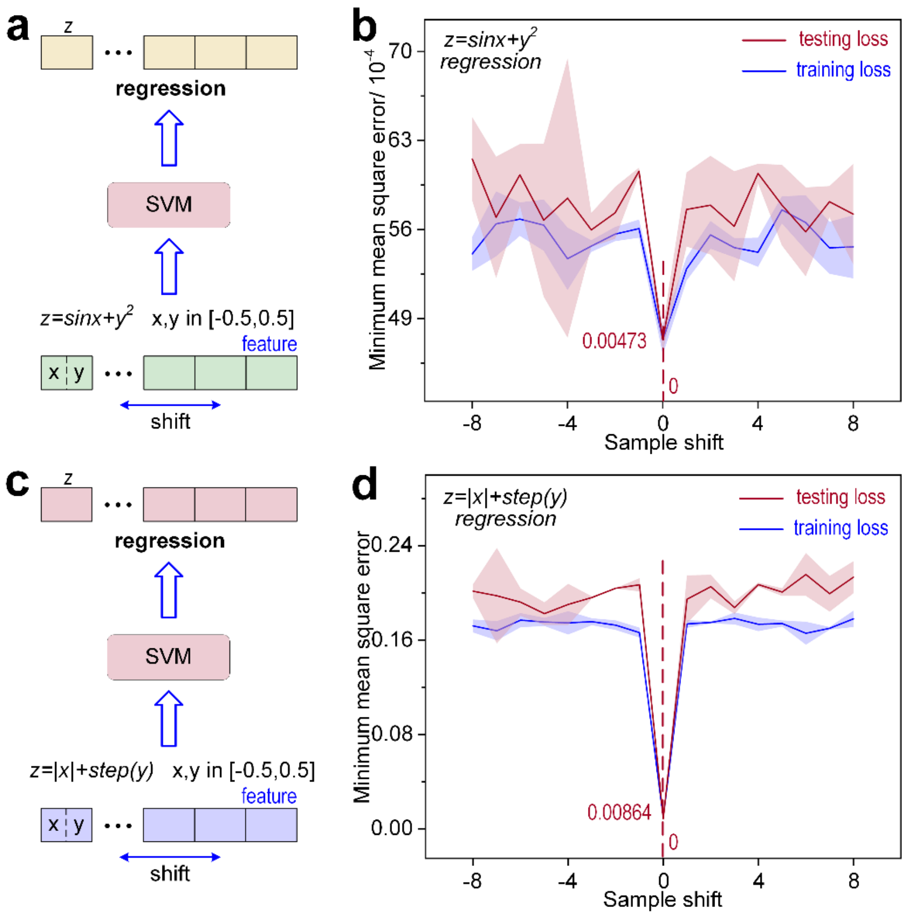

Supplementary Figure 31. **Promotion of the alignment architecture (supplements for Fig. 4). a-b** Schematic diagram of aligning the input of artificially constructed functions ($z = \sin x + y^2$) with the corresponding output via the support vector machine (SVM) kernel model and corresponding alignment results. **c-d** Schematic diagram of aligning the input of artificially constructed functions ($z = |x| + \text{step}(y)$) with the corresponding output via the SVM kernel model and corresponding alignment results.

Comment 3:

The authors train the DenseNet model for different time shifts and compare the validation accuracy to determine the time shift. This approach can be interpreted as a grid search with the neural network as a black box discriminator. I believe that the technical novelty and academic contribution from the ML perspective are quite low.

Answer of comment 3:

Thanks very much for your valuable comment. Despite your opposition to the publication of the paper, we still acknowledge the contribution of your suggestions to the improvement of this article. Your comment is very insightful, and it is our honor to communicate with you. As stated in answers of comments 1 and 2, we have demonstrated the generality of alignment architecture. In other words, machine learning models can be used to distinguish the degree of matching between input and output, which may be a universal property of machine learning. Although this is easy to understand, it differs from the conventional application of supervised learning and its derivative algorithms. Moreover, a paper that is easy to understand or implement may not necessarily mean that the core idea of the paper is easy to come up with. As we have read a study on metamaterials [1], which combines metamaterials with nonlinear gears, this idea is easy to understand for those who understand artificial intelligence and machinery, I think that such an idea is not far away from us but it is not easy to propose such an idea for the first time. Similarly, in the early days, the application of artificial intelligence in the field of material composition screening was also considered innovative [2]. Although, from our current perspective, this may not be very difficult., such research is rare in an era where artificial intelligence is not widely used. Given this, our research is suitable for the Nature Communications journal. We have made every effort to revise this paper and sincerely request that you give us a chance.

[1] Fang, X., Wen, J., Cheng, L., Yu, D., Zhang, H., & Gumbsch, P. Programmable gear-based mechanical metamaterials. *Nat. Mater.* **21**, 869-+ (2022).

[2] &Rickman, J.M., Chan, H.M., Harmer, M.P., Smeltzer, J.A., Marvel, C.J., Roy, A., Balasubramanian, G. Materials informatics for the screening of multi-principal elements and high-entropy alloys. *Nat. Commun*. **10**, 2618 (2019).

Reviewer #3

Comment 1:

The novelty of the manuscript is not clearly established. Further clarification needed how this approach is fundamentally different from existing neural network-based alignment and multimodal fusion methods.

Answer of comment 1:

Thanks very much for your valuable comment. Based on your comment, we have supplemented the description of cross-correlation and supervised learning algorithms based on cross-correlation algorithms in the in sections “1. Introduction” and “2.1. Alignment requirements and architecture” to indicate the limitations of cross-correlation algorithms. And we also made corresponding revisions to the “abstract” of the paper. We have also clarified the differences between the proposed architecture and existing neural network-based alignment, as well as the relationship between the proposed architecture and multimodal fusion methods in “1. Introduction” section.

Although the application scenarios of alignment in the field of multimodal fusion and transformation are

The revised contents are in “Abstract” section are shown as:

Via cross-correlation algorithms or synchronized acquisition of signals, the alignment of heterogeneous data with unknown semantic time shifts and intermittent semantic variations cannot be solved. The shift is caused by different data acquisition principles of sensors, different response discrimination principles using heterogeneous data, etc.

The revised contents are in “1. Introduction” section are shown as:

The term "alignment" is commonly used in three contexts: alignment between the source and target domains in transfer learning [27–29], alignment in multimodal transformation and fusion [30–32], and alignment of signals along the timeline [33–36]. With respect to transfer learning, the essence of domain generalization lies in aligning the features of the source and target domains to leverage information from the source domain, thereby enhancing model performance on tasks in the target domain [29,37-42]. With respect to multimodal domains, alignment is fundamental to the effective fusion of multimodal information [31]. In multimodal learning, alignment involves projecting data from different modalities into a shared semantic space, where semantically similar concepts are placed closer together, thus facilitating the integration of diverse information sources [32]. Similarly, the goal of temporal signal alignment is to ensure semantic consistency between corresponding segments across signals. While hardware-based synchronization is the preferred approach, the differing acquisition principles of heterogeneous sensors and the varying discrimination based on heterogeneous data often limit its applicability. In such cases, cross-correlation analysis [43-45], is considered. However, cross-correlation analysis often requires prior domain knowledge, such as understanding the positive or negative correlation between signal features. To enhance applicability, supervised learning has been employed to derive transformation functions that generate effective features for alignment [46]. A significant limitation of this approach is the reliance on manually annotated semantic time shifts between signals as the gold standard for training[46]. Aligning heterogeneous data without domain knowledge is challenging [47], especially when semantic time shifts are unknown and semantic variations are transient [48, 49]. First, the unknown semantic time shifts hinder the extraction of manually annotated semantic time shifts and effective alignment features [46]. Furthermore, transient semantic variations further hinder the establishment of associations between heterogeneous data. Consequently, a robust and unsupervised alignment method is urgently needed to overcome the associated challenges. Given the successful use of neural networks for alignment in transfer learning and multimodal domains [27–32, 37–42], a critical research question arises: can a novel neural network-driven architecture be designed to address the challenges of heterogeneous data

alignment with unknown semantic time shifts and intermittent semantic variation?

The supplemented contents are in "2.1. Alignment requirements and architecture" section are shown as: where $x_1[n,t]$ and $x_2[n,t]$ denote the nth two-dimensional time series signal with a time shift (TS) t in the randomly sampled signal segment and the corresponding one-dimensional time series signal segment, respectively. The corresponding time length of signal segments $x_1[n,t]$ and $x_2[n,t]$ is T_s. In this case, $\mathbf{A}^{n,t}$ represents an OS or IS image, and $x_2[n,t]$ is a one-dimensional AS segment with amplitude $a_s^{n,t}$ and length s. The segmentation details of the signal segment and the symbol definition rules of Equation (1) are shown in Fig. 1a. For signal alignment, cross-correlation algorithms are commonly employed, as shown in Equation (2):

$$\begin{Bmatrix} t_c = \max_t \sum_{n=1}^{N} F_1^{tf}(x_1[n,0]) \cdot F_2^{tf}(x_2[n,t]) \\ T_c = t_c \cdot T_s \end{Bmatrix} \tag{2}$$

where T_c and t_c denote the semantic TS and semantic alignment parameters, respectively, between the sequences of signal segments $x_1[n,0]$ and $x_2[n,0]$ obtained via the cross-correlation algorithm, F_1^{tf} and F_2^{tf} denote the corresponding transformation functions of $x_1[n,t]$ and $x_2[n,t]$, respectively; and the search range for semantics is $t \cdot T_p$. However, in this case, defining F_1^{tf} and F_2^{tf} is challenging because of the unclear positive correlation between certain features of $x_1[n,t]$ and $x_2[n,t]$. Moreover, supervised learning is not applicable for determining F_1^{tf} and F_2^{tf} [46] because of the unknown and inherently ambiguous nature of the true semantic TS between $x_1[n,0]$ and $x_2[n,0]$. Addressing these challenges necessitates a robust unsupervised algorithm or architecture, the development of which demands further analytical and experimental investigation.

Comment 2:

The paper does not clearly specify the threshold criteria for identifying arc-related acoustic events. Clear information on the amplitude, energy, or statistical thresholds used to distinguish arc events from background signals, and how these thresholds were determined.

Answer of comment 2:

Thanks very much for your valuable comment. Based on your comment, we have supplemented the corresponding threshold determination criteria in the "Methods" section, and also provided a detailed description of acoustic sensor parameter settings and signal processing in the "Methods" section.

The supplemented contents are in the "In situ test of current-carried friction" in the "Methods" section are shown as:

Optical signals were captured via a high-speed camera (Revealer X213) with a sampling frequency of 40 Hz, an exposure time of 25 ms, and a resolution of 1024 × 768 pixels. Simultaneously, infrared data were acquired with a thermal imager (Telops Spark M150), which recorded at 80 Hz over a temperature range of -20 to 350°C, yielding raw images at a resolution of 640 × 556 pixels. The acoustic signals were collected via a dual-channel system synchronized for data acquisition. The first channel incorporated a wideband acoustic emission sensor (PAC WSa; 1–1000 kHz) connected to a PAC 2/4/6 amplifier. The second channel consisted of an air-coupled sensor (PAC AF62; 1–34 kHz) coupled with a PAC 1281 amplifier. Both channels shared an upper computer (PAC PCI-2) and were controlled by the software (PAC AEwin for PCI2). The signals collected by both sensors were processed through an analog bandpass filter with a frequency range of 1 kHz to 3 MHz. The system supports two acquisition modes: complete waveform acquisition and threshold-based segmented waveform acquisition. For the current-carrying friction test, both airborne and wideband sensors were employed in full waveform acquisition mode, with a sampling frequency of 2 MHz and a preamplifier gain of 40 dB. In contrast,

for the acoustic signals energy comparison test (Supplementary Figs. 3, 10, and 18), to facilitate the extraction of energy information, airborne sensors were utilized in threshold-based acquisition mode, with a sampling frequency of 2 MHz, a preamplifier gain of 40 dB, a waveform length of “1k”, and a threshold of 10 dB (the minimum value supported by the system). In these experiments, optical, infrared, and acoustic signals were collected approximately synchronously via synchronized triggering software. The mechanical load signal was measured with a bidirectional force sensor (Huilizhi LZ-LW64) at a sampling frequency of 10 Hz. Current and voltage data were acquired from a programmable power supply (PSY DCPS3014) at a sampling frequency of 1 Hz. The optical, acoustic, and infrared signals were synchronized approximately via synchronous triggering software.

Comment 3:

The details of the synchronization process across acoustic, optical, and infrared sensors are not clearly described. A description of the sensor synchronization procedure and the process used to validate the ground truth time shift independently of the neural network would improve clarity.

Answer of comment 3:

Thanks very much for your valuable comment. Based on your comment, we have supplemented the approximate synchronization process of the signal and the usage details of the synchronization triggering software in the “Methods” section. And upload the synchronization trigger software to GitHub. The relevant descriptions of impact testing and arc response testing of arc igniters have been supplemented in the “Methods” section, both of which are used to determine the reference value of the time shift of the system. The corresponding figures and descriptions related to optical signals, infrared signals, and acoustic signals have been supplemented to the “Supplementary Discussion 2. Datasets used for the alignment study” section.

The supplemented contents are in the “In situ test of current-carried friction” in the “Methods” section are shown as:

Line 443, Revised Manuscript: The optical, acoustic, and infrared signals were synchronized approximately via synchronous triggering software.

The supplemented contents are in the “Impact in-situ test” in the “Methods” section are shown as:

The impact testing equipment was a small mini cannon, with an accelerated component driven by an electromagnetic coil. The projectile was made of bearing steel, with a diameter of 6 mm and a mass of 6.5 g. The cylindrical projectile was accelerated to 58 m/s by the mini cannon and subsequently impacted a flat specimen made of aluminum alloy with dimensions of 80 × 80 × 4 mm. In-situ signals during the impact process were approximately synchronously collected via high-speed cameras, infrared thermal imagers, and airborne acoustic emission sensors to capture three key moments of the impact. The time shift reference range between the signals was determined by comparing these three moments (Supplementary Fig. 9c), which were subsequently used to validate the alignment effect on the basis of the signals in datasets **2-2** and **2-3**. The measurement surfaces of the optical, infrared, and acoustic sensors were adjusted to focus on the impact location collectively.

The optical, acoustic, and infrared signals were synchronized approximately via synchronous triggering software. The experiment was conducted over four trials. In the first and second experiments, the exposure time of the high-speed camera was 25 ms, with sampling frequencies of 40 Hz for the high-speed camera and 80 Hz for the infrared thermal imager. In the third experiment, the exposure time of the high-speed camera was reduced to 12.5 ms, with sampling frequencies of 80 Hz for both the high-speed camera and the infrared thermal imager. In the fourth experiment, the exposure time of the high-speed camera was further reduced to

6.25 ms, with sampling frequencies of 160 Hz for both the high-speed camera and the infrared thermal imager. In these experiments, acoustic signals were collected via the airborne acoustic emission sensor in full waveform mode, with a sampling frequency of 2 MHz and a preamplifier gain of 40 dB. The signals were processed through an analog bandpass filter with a frequency range of 1 kHz to 3 MHz.

The supplemented contents are in the "In situ test of the arc response of the igniter" in the "Methods" section are shown as:

A dedicated calibration experiment was conducted to quantify the time shift between the optical and acoustic signals of the system. An arc was generated in a darkroom with the igniter from Supplementary Fig. 9d. The synchronous triggering software coordinated the high-speed camera and acoustic emission sensor to record the event. The measured time shift between the optical and acoustic recordings of the arc ignition served as the ground-truth reference for validating the temporal alignment of the signals in datasets from **3-1** to **3-3**. The experiment was conducted seven times, with an optical signal sampling frequency of 40 Hz and an exposure time of 25 ms. Acoustic signals were collected via an air-coupled acoustic emission sensor in full waveform acquisition mode, with a sampling frequency of 2 MHz and a preamplifier gain of 40 dB. The signals were processed through an analog bandpass filter with a frequency range of 1 kHz to 3 MHz.

The supplemented contents are in the "Synchronize triggering software" in the "Methods" section are shown as:

To bypass the hardware synchronization process and accelerate the acquisition of experimental data for verifying the alignment architecture, synchronization triggering software (the software and scripts involved in the Methods section are detailed at https://github.com/ChaofanLi-JinlinUniversity/Alignment-Architecture-of-Heterogeneous-Data) was used on the basis of preset click steps, to approximately synchronize the activation of the corresponding start collection buttons on the user interface, enabling the approximately synchronous acquisition of heterogeneous signals. This method reduces random errors in the time shift between signals compared with manually activating the start collection buttons. Simultaneously, to verify the alignment results of the alignment architecture, via synchronization triggering software, in situ impact tests and arc response tests of the igniter were conducted to obtain the reference time shift between heterogeneous signals.

The supplemented and revised contents are in the "Supplementary Discussion 2. Datasets used for the alignment study" section are shown as:

Line 190, Supplementary Information: To bypass the hardware-level synchronization of the in-situ monitoring module and accelerate validation of the alignment architecture, software-based synchronization triggering was employed to approximately synchronize the acquisition of the OS, IS, and AS.

Moreover, the reference time shift (TS) range among the OS, IS, and AS via software-based synchronization triggering was estimated on the basis of additional photoacoustic thermal response tests (Supplementary Fig. 9), providing a reference for evaluating the alignment performance of the proposed architecture. Physical events that could be simultaneously detected by high-speed cameras, infrared thermal imagers, and AE sensors, such as the start and stop of friction disc rotation, were considered reference points for signal alignment. The fixture holding the disc-shaped specimen is visible in the IS image, as shown in Supplementary Fig. 9a. In the comparison between the upper left image and the lower left image, the disc-shaped specimens started to rotate at 37.4500 s. Similarly, the comparison between the upper right image and the lower right image indicates that the friction ceased at 933.2125 s. Therefore, according to the IS, the total duration of friction was 895.7625 s. In parallel, the AS can also be compared to distinguish the onset and

[REDACTED]

Supplementary Figure 9. **Reference time-shift range between the signals of the testing system. a** Discrimination criteria based on infrared signals (OSs) to determine whether the friction disc is rotating. **b** Spectrogram of the acoustic signals (AS) corresponding to the start and stop of the friction disk rotation. **c** Approximate range of the time shift between OS, infrared signals (IS) and AS for datasets **2-2** and **2-3** of the experiments, obtained from the impact test. **d** Approximate range of the time shift of the system signal for datasets from **3-1** to **3-3** of the experiments, obtained from the arc igniter test.

cessation of disc rotation, allowing the friction duration to be identified, as shown in Supplementary Fig. 9b. The duration of friction determined from the AS was approximately 897.425 s, which was slightly longer than the 895.7625 s estimated from the IS. This discrepancy arises because the ASs were more sensitive to subtle variations and fluctuations in the friction load, whereas the low friction speed resulted in less pronounced variation between consecutive infrared frames. Four additional experiments were conducted on the basis of a more transient process to further support signal alignment. The experiments involved metal columnar projectiles impacting metal plates at a launch speed of approximately 58 m/s. The impact produced clear acoustic, optical, and infrared responses, allowing for effective cross-modal alignment, as shown in

Supplementary Fig. 9c. Via the impact experiments, reference ranges for the TS among the OA, AS, and IS in datasets **2-2** and **2-3** were determined. The TS t_i^a between the AS and IS ranged from -59.50×25 ms to -54.25×25 ms, whereas the TS t_o^a between the AS and OS ranged from -60.00×25 ms to -52.50×25 ms, which indicated that the TS between the signals consisted of a major systematic error and a minor random error. In addition to impact response testing, the photoacoustic transient response event of the arc generated by the arc igniter can also serve as a reference for signal alignment. The corresponding acoustic response is shown in Supplementary Fig. 9d, where the spectrogram exhibits a pronounced signal in the 17–19 Hz range. According to seven arc response tests, the TS t_o^a between OSs and ASs in datasets from **3-1** to **3-3** was determined to be within the range of -48.00×25 ms to -52.00×25 ms. Although each experiment could be approximately synchronized on the basis of other physical events such as impacts, microexplosions, or arc responses, the alignment parameters derived from these auxiliary events may not completely coincide with the semantic alignment parameters between signals of the target response.

Comment 4:

According to Nyquist theory, the sampling rate should be at least 2.5 times higher than the maximum AE frequency. The authors used a 2 MHz sampling rate, while the effective frequency goes up to 1 MHz. This may cause loss of important high-frequency information. It is recommended to perform tests with higher sampling rates to confirm whether any data was lost.

Answer of comment 4:

Thanks very much for your valuable comment. Based on your comment, we have supplemented the research on the impact of different frequency ranges of acoustic signal on alignment in “Supplementary Discussion 6. The impact of the spectrogram frequency range on alignment” section. Because the frequency range above 1MHz may not affect the validation of the alignment architecture in this case, we did not conduct additional friction testing. However, we have demonstrated the effectiveness of your proposed viewpoint through supplementary alignment experiments and have included your ideas in “Supplementary Discussion 6. The impact of the spectrogram frequency range on alignment” section to ensure the professionalism of the paper.

The supplemented contents are in the “Supplementary Discussion 6. The impact of the spectrogram frequency range on alignment” section are shown as:

Additionally, during the implementation of the alignment architecture, minimizing information loss to ensure accurate signal representation is essential. The airborne acoustic emission sensor collected acoustic signals at a sampling frequency of 2 MHz. Although the measurement range of the sensor is 1–34 kHz, the highest effective frequency of its spectrogram is 1 MHz according to the Nyquist theorem. Therefore, a spectrogram of the frequency range of 0–1 MHz was generated to align the optical signals and acoustic signals, and the results were compared to those based on spectrogram of the frequency range of 0–34 kHz, as shown in Supplementary Fig. 19. As illustrated in Supplementary Figs. 19a–c, both approaches yield comparable alignment accuracies. Supplementary Fig. 19d presents the maximum testing accuracies for the kernel model achieved at the aligned time shift for both the 34 kHz and 1 MHz spectrograms. The 1 MHz spectrogram yields an accuracy of 91.7%, which exceeds the 89.8% obtained on the basis of the 34 kHz spectrogram. Therefore, effective information is included in spectrograms of 34 kHz-1 MHz. Effective information typically leads to improved alignment accuracy, as higher tesing accuracy of the kernel model under aligned time shifts may reflect more reliable alignment. Additionally, for the processing of one-dimensional signals used in alignment architectures, on the basis of ensuring that the sampling frequency of the signal is at least 2.5 times the highest frequency of the spectrogram, it is necessary to explore whether there is effective information in a wider

frequency band, which may be beneficial for alignment.

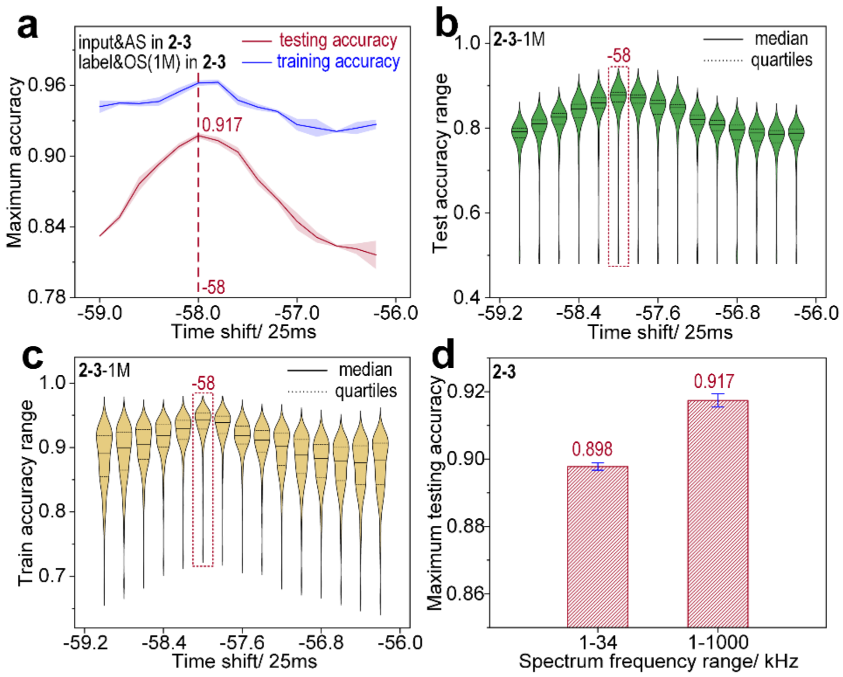

Supplementary Figure 19. **Influence of the frequency range of the spectrogram from the acoustic signal (AS) collected by the airborne acoustic emission sensor on the alignment. a** Time shift (time step: 5 ms)-highest accuracy curve of alignment between the AS spectrogram in the 1 MHz frequency range and the optical signal (OS). **b-c** Time shift (time step: 5 ms)-testing accuracy distribution and time shift (time step: 5 ms)-training accuracy distribution, respectively, under the same conditions. **d** Influence of the frequency range of the spectrogram on the maximum testing accuracy of the kernel model for the aligned parameters.

Comment 5:

The rationale behind the chosen combinations of load mode, current, and speed is not explained. Justification for selecting these specific parameters and their relevance to realistic or challenging test conditions would strengthen the study.

Answer of comment 5:

Thanks very much for your valuable comment. Based on your comment, we have supplemented the basis for parameter selection in “Supplementary Discussion 2. Datasets used for the alignment study” section.

The supplemented contents are in the “Supplementary Discussion 2. Datasets used for the alignment study” section are shown as:

To support the alignment and application research of multispectral acoustic signals, a series of orthogonal experiments were conducted with rotational speed, loading current, and loading mode as raw variables. In current-carried friction testing, the performance of friction pairs is typically evaluated through equivalent current-carried experiments. To ensure the applicability of the conclusions drawn in this study, the parameter settings for the orthogonal experiments closely matched the range supported by the developed testing instrument and were comparable to those of standard commercial systems (e.g., MS-MT9000). The wear radius of the experiment was uniformly set to 23 mm, with speeds of 50 r/min, 100 r/min, and 150 r/min. The loading voltage was uniformly set to 10 V, while the loading currents were 2 A, 5 A, and 8 A, respectively. The loading modes included constant displacement loading (CDL), constant load loading (CLL), and constant minor load loading (CMLL).

Comment 6:

The stability of background reference images over time is not addressed. It would be useful to explain how drift in background illumination or temperature was controlled during preprocessing.

Answer of comment 6:

Thanks very much for your valuable comment. Based on your comment, the comparison of the generalization performance of image detection models trained based on original images, dynamically threshold adaptive processing images, and histogram equalization processing results has been supplemented in the "Supplementary Discussion 7. Feasibility of arc detection via optical signals" section.

The supplemented and revised contents are in "Supplementary Discussion 7. Feasibility of arc detection via optical signals" section are shown as:

The effectiveness of arc detection via infrared signals or acoustic signals was previously evaluated via optical signals (OSs) as a reference. To further verify the reliability of the OS in arc detection, arc detection models were established with OSs as inputs. The dataset for these models consisted of OS data from datasets from **2-2** to **3-3**, along with manually annotated labels derived from the OS data (Supplementary Table 4). Details of the models and their corresponding training parameters are provided in Supplementary Table 5. Supplementary Fig. 20 shows the preprocessing results for the OS data and the performance of the corresponding arc detection models. Since OS images contain both infrared and visible spectral spectrum components, variations in light intensity and background temperature may lead to domain drift. Therefore, it is essential to explore preprocessing methods to mitigate domain shifts in OS images. Supplementary Figs. 20a-c show the original OS images from datasets **2-2** and **2-3**, the images processed via histogram equalization, and those processed via dynamic threshold adaptive methods. Supplementary Figs. 20d–20f show the epoch-loss and accuracy curves, along with the corresponding confusion matrices, for arc detection models trained on original images, images preprocessed via histogram equalization, and images preprocessed via dynamic threshold adaptive methods, respectively. Among the three models, the model trained on dynamically thresholded images demonstrates the most stable training process and achieves the highest testing accuracy, reaching 97.2%, outperforming the other two preprocessing approaches. Supplementary Fig. 20g compares OS images from datasets from 3-1 to 3-3 and 1-1-1, highlighting domain shifts caused by differences in background lighting and temperature. To evaluate the effectiveness of the three preprocessing methods in mitigating domain shift, the models that achieved the maximum testing accuracy on datasets 2-2 and 2-3 (Supplementary Fig. 20d: epoch 100, Supplementary Fig. 20e: epoch 62, Supplementary Fig. 20f: epoch 89) were tested on datasets from 3-1 to 3-3. The test results, shown in Supplementary Fig. 20h, indicate that the model trained on histogram-equalized images exhibits the weakest generalization performance. In contrast, the models trained on raw images and dynamically thresholded images demonstrate comparable generalization capabilities. Although the model trained on raw images performed well in this case, its corresponding generalization performance may be less reliable when it is applied to optical images with more significant domain shifts. The arc temperature (3,000–30,000 °C), which is sufficient to melt metal, significantly differs from the background environment. For OS data collected in a darkroom, the dynamic threshold adaptive preprocessing method effectively preserves key features at the interface between the arc and the background. Moreover, the resulting binary images, composed solely of black and white pixels, help to substantially mitigate the domain shift. Accordingly, dynamic threshold adaptive preprocessing is recommended to increase the robustness of arc detection under varying imaging conditions.

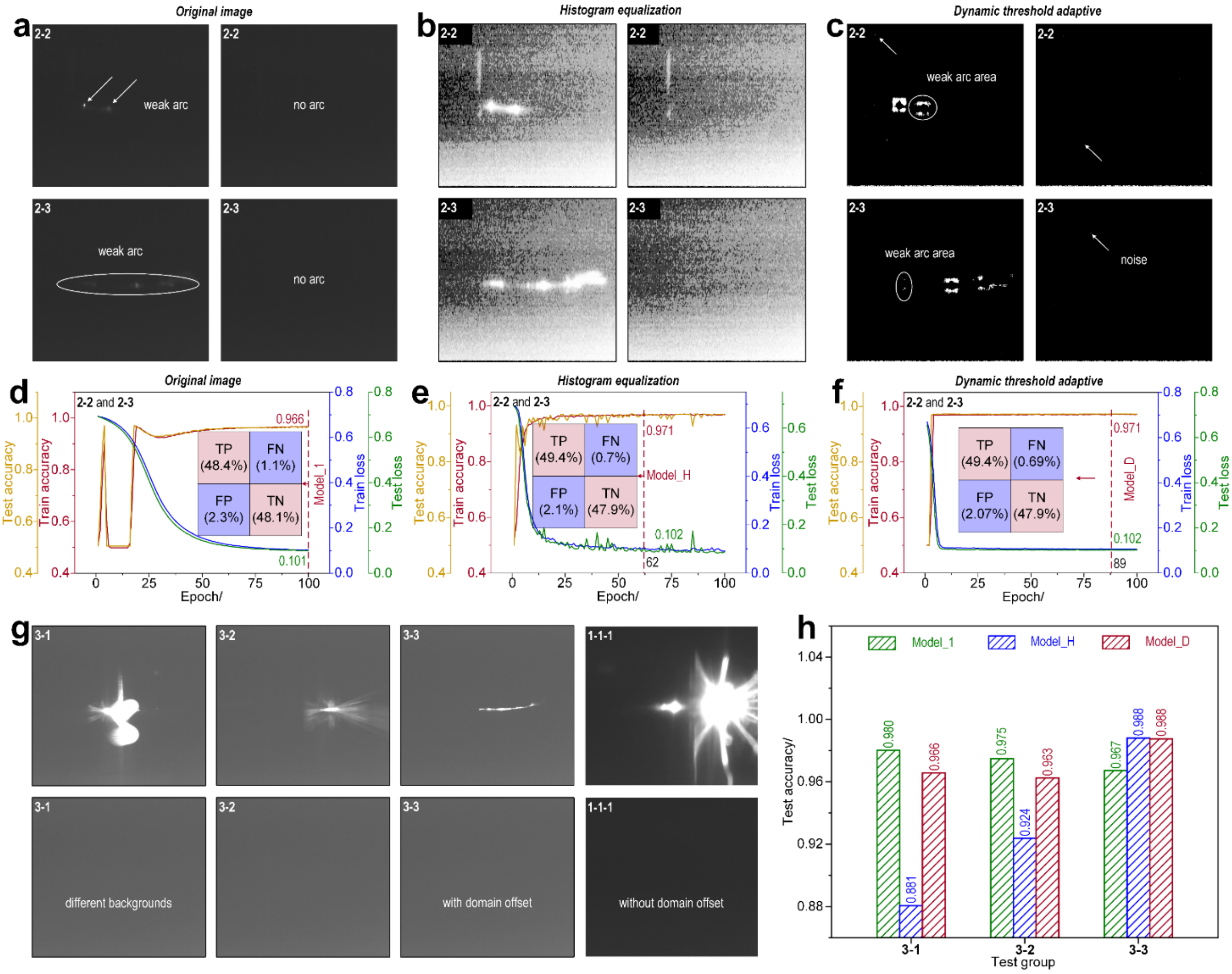

Supplementary Figure 20. **The effect of optical signal preprocessing on arc detection model performance with optical images as input**. **a** Raw images of typical optical signals from datasets **2-2** and **2-3**. **b-c** Images obtained via histogram equalization and dynamic threshold adaptive processing of these original images, respectively. **d-f** Training epoch-loss/accuracy curves of the arc detection model with the original image, the image processed via histogram equalization and the image processed via the dynamic threshold adaptive as inputs. The corresponding confusion matrix includes the true positive (TP) rate, false positive (FP) rate, true negative (TN) rate, and false negative (FN) rate. **g** Comparison of raw images in experiments between datasets from **2-2** to **3-3** and **1-1-1**. **h** Generalization performance of the model shown in d-f for image signals in datasets from **3-1** to **3-3**.

Comment 7:

It is not clear whether additional filtering was applied to the acoustic signals beyond bandpass filtering. Details on any temporal or spectral denoising methods used to handle cantilever vibrations or other noise sources would be important.

Answer of comment 7:

Thanks very much for your valuable comment. Based on your comment, we have supplemented the detailed description of the used for acoustic signals in the "Methods" section. We did not use any filters other than bandpass filters. We have also revised the corresponding content in the "Supplementary Discussion 2. Datasets used for the alignment study" section to indicate the reason for not using other filters. We initially considered

cantilever vibration because a wideband acoustic emission sensor connected to a fixture, which may hinder its collection of the response of the arc itself. Therefore, we introduced an air coupled acoustic emission sensor. However, later interpretability analysis based on acoustic signals showed that arc detection using acoustic signals is based on the acoustic response of the friction pair after the arc response rather than the arc response itself.

The supplemented and revised contents are in "In situ test of current-carried friction" section in "Methods" section are shown as:

Line 443, Revised Manuscript: The signals collected by both sensors were processed through an analog bandpass filter with a frequency range of 1 kHz to 3 MHz.

The supplemented and revised contents are in "Impact in-situ test" section in "Methods" section are shown as:

Line 464, Revised Manuscript: The signals were processed through an analog bandpass filter with a frequency range of 1 kHz to 3 MHz.

The supplemented and revised contents are in "In situ test of the arc response of the igniter" section in "Methods" section are shown as:

Line 474, Revised Manuscript: The signals were processed through an analog bandpass filter with a frequency range of 1 kHz to 3 MHz.

The supplemented and revised contents are in "Supplementary Discussion 2. Datasets used for the alignment study" section are shown as:

Line 257, Supplementary Information: The energy of background noise fluctuates within the range of 1000-6000, whereas the energy associated with the current-carried friction ranges from 25000-36000. The background noise contributes no more than 25% of the total AS energy, indicating minimal interference with the frictional acoustic response. Accordingly, no additional denoising was applied to the acquired ASs.

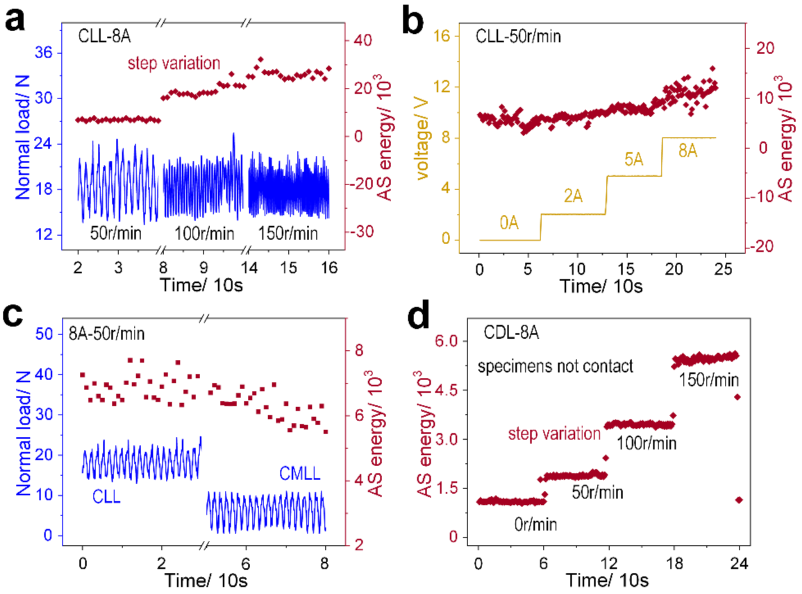

Supplementary Figure 10. **Analysis of the energy of the acoustic signals (AS). a-b** Energy variation of the AS at different speeds and currents under constant load loading (CLL) mode. **c** Energy of the AS under CLL and constant minor load loading (CMLL) modes. **d** The energy of the AS in the background of the system corresponding to different rotational speeds under constant displacement loading (CDL) mode.

Comment 8:

No information is provided about the repeatability of alignment results across multiple training runs. Reporting the variance in estimated time shifts across repeated model trainings would help assess consistency.

Answer of comment 8:

Thanks very much for your valuable comment. Based on your comment, we have supplemented the repeatability experiment of data alignment (the previous version) in "2.2. Alignment verification and applications" section and the "Supplementary Discussion 4. Repetitive and supplementary alignment" section. The number of repetitions for all alignment experiment (Including other experiments supplemented during this revision) is shown in Supplementary Table 5.

The supplemented contents are in "2.2. Alignment verification and applications" section are shown as:

Additionally, we repeated the above alignment, and the alignment between the OS and AS corresponding to different friction pair materials was analyzed as detailed in Supplementary Discussion 4. The experimental results indicate that the comprehensive results of repeated alignment are more reliable than those of single alignment are, and that determining the semantic TS via the distribution variation of testing accuracy across different TSs is more reliable than relying solely on the MTA. The subsequent alignment-related figures correspond to the comprehensive results of three repeated alignments and will not be repeated in the future.

The supplemented and revised contents are in Supplementary Table 5. are shown as:

Supplementary Table 5. **Training parameters of the kernel models**

Illustration	Task (number of repetitions)	Dataset(Proportion of positive and negative samples)	Model	Epoch	Learning rate	Batch
Fig. 2a	Alignment of label&IS and AS (1)	**2-3**:3600 samples(1:1)	RDNet41	100	2×10^{-4}	512
Figs. 2c-f Supplementary Fig. 13	Alignment of label&OS and AS (1)	**2-3**:9000 samples(1:1)				
Supplementary Figs. 14a-f Supplementary Figs. 19	Alignment of label&OS and AS (3)					
Figs. 2g, 2i	Alignment of label&OS and AS(transfer training final layer) (1)	**2-2**:500 samples(1:1)		1000	8×10^{-2}	
Supplementary Figs. 11h-j	Alignment of label&OS and AS(transfer training final layer) (3)					
Figs. 2g, 2 h	Alignment of label&OS and AS (training from start) (1)			100	2×10^{-4}	
Fig. 2g	Alignment of label&OS and AS (globally transfer training) (1)			500	1×10^{-6}	
Supplementary Figs. 15a-e	Alignment of label&OS and AS (3)	**3-1**:10000 samples(1:1)		100	2×10^{-4}	
Supplementary Figs. 15f-j		**3-2**:14000 samples(1:1)				
Supplementary Figs. 16c-h		**3-3**:3800 samples(1:1)				
Supplementary Fig. 20d	Arc detection based on OS (1)	**2-2**:508 samples(1:1)+ **2-3**:9626 samples(1:1)	RSNet1		1×10^{-6}	128
Supplementary Fig. 20e					1×10^{-5}	
Supplementary Fig. 20f						256
Figs. 3a-c	Alignment of label&OS and AS (3)	**2-3**:9000 samples(1:1)	RRNet18 RRNet10 RMNet MNet4 MNet3 RDNet41 RDNet13 MNet2_64 MNet2_32 MNet2_16 MNet2_8 MNet2_4 MNet2_2 MNet2_1		2×10^{-4}	512
Figs. 3d-f Fig. 4a		**2-3**:50-9000 samples(1:1)	RDNet13		2×10^{-4} 4×10^{-4} 2×10^{-3}	
Supplementary Fig. 24			MNet2_1		2×10^{-4} 2×10^{-2} 1×10^{-1} 5×10^{-1}	
Figs. 3g-i		**2-3**:5000 samples(1:8 - 8:1)	RDNet41		2×10^{-4}	
Supplementary Fig. 26 Fig. 4a		**2-3**:50-9000 samples(1:1)	/	/	/	/
Figs. 4b-c Supplementary Fig. 28	Alignment of label&AS and AS (3)	Generated data: 9000 samples(1:1)	GRU	200	1×10^{-2}	512
Supplementary Figs. 30a-c	Alignment of label&IS and IS (3)	**2-3**: 5000 samples(1:1)	RSNet1	100	2×10^{-3}	128
Supplementary Figs. 30d-f	Alignment of label&OS and OS (3)	**2-3**: 9000 samples(1:1)			1×10^{-5}	
Supplementary Figs. 30g-i	Alignment of label&OS and OS feature (3)		MLP		1×10^{-3}	1024
Figs. 4d-f Supplementary Figs. 30j-l	Alignment of label&function output and function input (3)	Generated data: 500(1:1)	SVM	/	/	/
Supplementary Fig. 31	Alignment of function output and function input (3)	Generated data: 500		/	/	/
Figs. 4g-I Supplementary Fig. 29	Alignment of feature vector&OS and OS (3)	**2-3**: 9000 samples(1:1)	RANet	50	1×10^{-4}	128

The supplemented contents are in the “Supplementary Discussion 4. Repetitive and supplementary alignment” section. are shown as:

To further assess the reliability of the alignment results produced by the proposed architecture, the alignment procedure was repeated, and the distributions of training and testing accuracy during kernel model training were analyzed. In the coarse alignment stage (step: 25 ms) of dataset **2-3** (Supplementary Fig. 14a), the alignment results from three independent replicates (data is not shown separately) were consistent with those shown in Fig. 2c. The relationship between the time shift (TS) and the mean of the maximum training and testing accuracies across the three alignment trials is shown in Supplementary Fig. 14a, which is consistent with the alignment results reported in Fig. 2c. The mean values of the maximum training and testing accuracies derived from multiple experiments provide a more reliable estimate than those from a single experiment. This is analogous to the improved accuracy observed in ensemble learning, where decision-making from multiple learners with equal weights outperforms that of a single learner. As shown in Supplementary Fig. 14b, the comprehensive distribution of testing accuracy across the three experiments follows the overall trend of mean maximum testing accuracy with TS, with testing accuracy consistently higher for semantic TSs than for other time shifts. The corresponding training accuracy distribution is shown in Supplementary Fig. 14c. Similarly, training accuracy under semantic TSs is consistently greater than that under other TS conditions. This can be attributed to the weaker correspondence between input signals and semantic labels in nonsemantic alignments, which reduces the proportion of functionally relevant components in the loss function that effectively drive model training, thereby slowing the training rate. Furthermore, although training accuracy generally remains higher than testing accuracy across all conditions, it exhibits less variability in response to TSs than testing accuracy does, which suggests that the kernel model perceives semantic alignment parameters more effectively in the testing set than in the training set. The pre-AlexNet era was defined by traditional machine learning models, such as support vector machines (SVMs). In contrast, neural networks that emerged following the AlexNet breakthrough are typically characterized by increased channel widths, which foster the development of diverse random features. This architectural approach expands the solution space and enhances representational power, enabling these networks to outperform earlier machine learning methods. While neural networks may exhibit better predictive variance than more industrially robust methods such as SVMs do, this variability can be constructively leveraged. When kernel models are trained across multiple epochs and under repetitive alignment conditions, the aggregated predictions form a stable, ensemble-like distribution at each TS. This concept parallels the idea that integrating observations from multiple perspectives leads to a more accurate interpretation. Thus, for semantic TSs, evaluations based on the full distribution of testing accuracy provide a more reliable metric than those relying solely on the maximum testing accuracy. The distribution provides a more comprehensive view of the training process, whereas the maximum testing accuracy typically reflects the credibility of the alignment results, although it is less robust. The comprehensive results of three repeated fine alignments (step size: 5 ms) for dataset **2–3** are shown in Supplementary Figs. 14d–f. Similarly, the results of three repeated coarse alignments (step size: 5 ms) for the **2–2** experiments are presented in Supplementary Figs. 14g–j. These comprehensive alignment results are consistent with previous findings. Supplementary Fig. 14i shows the TS–maximum testing accuracy curves for the three repeated alignments of the dataset **2–2** . The trends of the three curves at the semantic TS are consistent, but there are inconsistencies at other TS positions. In some cases, these inconsistencies may propagate to the semantic shift and affect the alignment outcome. Therefore, for subsequent alignments, the alignment experiment was repeated three times.

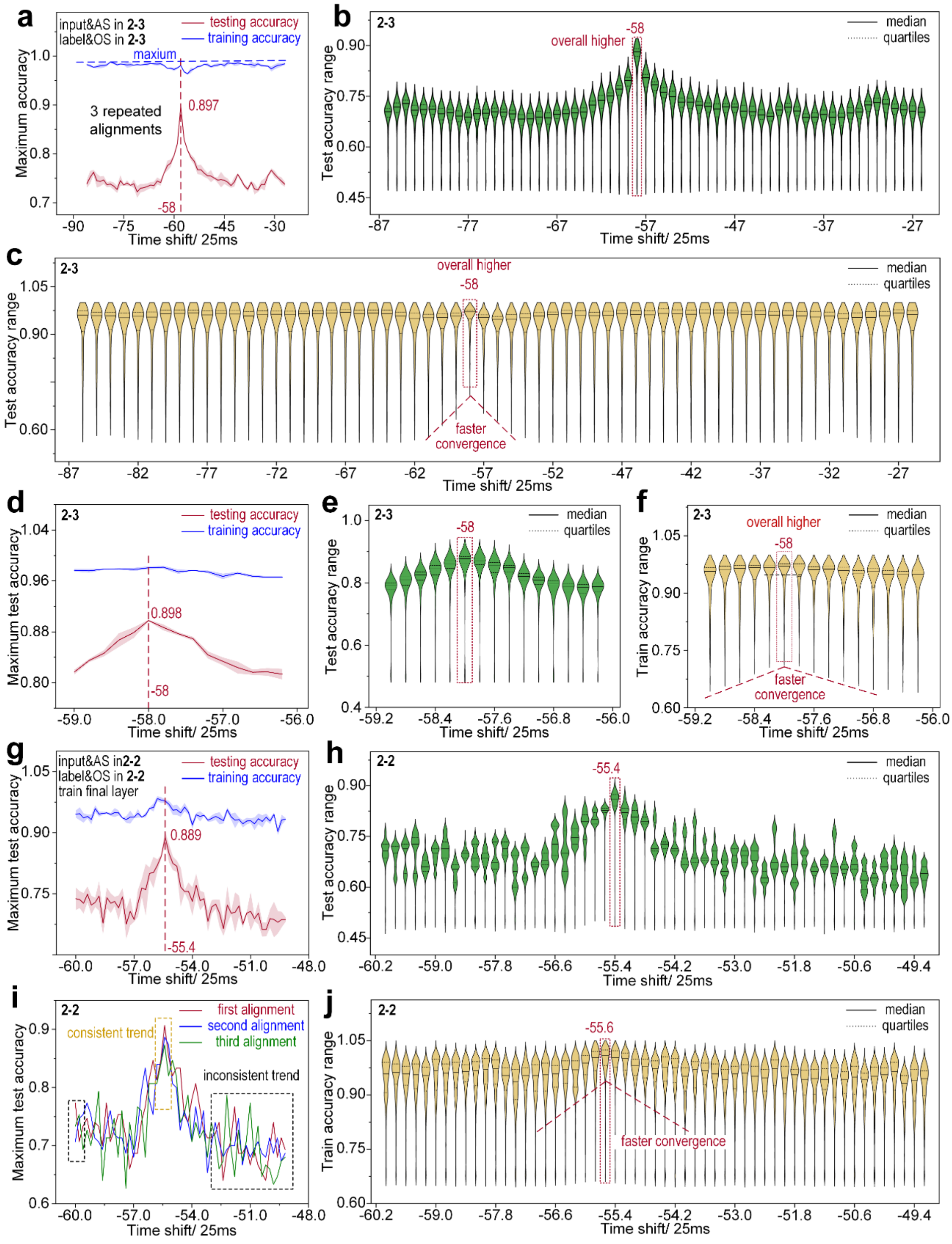

Supplementary Figure 14. **Shift–maximum accuracy curves and accuracy distributions for aligning optical signals (OS) and acoustic signals (AS) in datasets 2-2 and 2-3.** **a-b** Time shift(step size: 25 ms)-maximum training and testing accuracy curves and testing accuracy distributions corresponding to 3 alignments of **2-3** sets of OS and AS, respectively. **c** Time shift (step size: 25 ms)-train accuracy distributions in dataset **2-3**. **d** Time shift (step size: 5 ms)-maximum accuracy curves in dataset **2-3**. **e-f** Time shift (step: 5 ms)-testing accuracy distributions and training accuracy distributions, respectively, in dataset **2-3**. **g** Time shift (step: 5 ms)-maximum accuracy curves in dataset **2-3**. **h, j** Time shift (step: 5 ms)-testing accuracy distributions and training accuracy distributions, respectively, in dataset **2-2**. **i** Time shift (step: 5 ms)-maximum testing accuracy curve corresponding to the alignment repeated 3 times, in dataset **2-2**.

Comment 9:

There is no direct comparison to conventional alignment techniques such as cross-correlation or dynamic time warping. Including baseline performance comparisons would help demonstrate the advantage of the proposed method.

Answer of comment 9:

Thanks very much for your valuable comment. Based on your comment, we have supplemented the description and discussion of traditional cross-correlation algorithms and alignment methods based on cross-correlation supervised learning in the "2.1. Alignment requirements and architecture" section. Meanwhile, we have not yet read any heterogeneous data alignment methods for this scenario. Therefore, in sections "2.4. Promotion of alignment architecture" and "Supplementary Discussion 10. The alignment method based on frequency domain similarity", we have supplemented the proposed alignment method based on frequency domain signal similarity, and a comparison between this method and the alignment architecture to highlight the advantages of the proposed architecture.

The supplemented contents are in "2.1. Alignment requirements and architecture" section are shown as:

Line 97, Revised Manuscript: For signal alignment, cross-correlation algorithms are commonly employed, as shown in Equation (2):

$$\begin{cases} t_c = \max_t \sum_{n=1}^{N} F_1^{tf}(x_1[n,0]) \cdot F_2^{tf}(x_2[n,t]) \\ T_c = t_c \cdot T_s \end{cases} \quad (2)$$

where T_c and t_c denote the semantic TS and semantic alignment parameters, respectively, between the sequences of signal segments $x_1[n,0]$ and $x_2[n,0]$ obtained via the cross-correlation algorithm, F_1^{tf} and F_2^{tf} denote the corresponding transformation functions of $x_1[n,t]$ and $x_2[n,t]$, respectively; and the search range for semantics is $t \cdot T_p$. However, in this case, defining F_1^{tf} and F_2^{tf} is challenging because of the unclear positive correlation between certain features of $x_1[n,t]$ and $x_2[n,t]$. Moreover, supervised learning is not applicable for determining F_1^{tf} and F_2^{tf} [46] because of the unknown and inherently ambiguous nature of the true semantic TS between $x_1[n,0]$ and $x_2[n,0]$. Addressing these challenges necessitates a robust unsupervised algorithm or architecture, the development of which demands further analytical and experimental investigation.

The supplemented contents are in "2.4. Promotion of alignment architecture" section are shown as:

Given the limited research on algorithms closely resembling the architecture proposed in this study, an alternative alignment method based on frequency domain similarity (Supplementary Discussion 10) was developed for comparative analysis. This method was evaluated further to illustrate the relative advantages of the proposed architecture. On the basis of the same alignment parameters, a comparison was conducted between the alignment results produced by the frequency domain similarity–based method and those obtained via the neural network–based architecture (Figs. 3d–f), as summarized in Fig 4a. Although the neural network–based architecture achieves slightly lower alignment accuracy (10 ms) than the frequency domain similarity–based method under the number of samples of 5,000, the architecture remains robust under data-limited conditions. In contrast, the accuracy of the frequency domain similarity–based method substantially decreases (120–165 ms) when the number of samples is reduced to 500 or less, whereas the neural network–based architecture maintains superior precision (5–20 ms). The results indicate that, although frequency domain representations of arc responses in the AS modality exhibit measurable similarity, such patterns are not prominent in small-sample analyses and require large datasets for reliable detection. The computational costs of the respective alignment methods are reported in Supplementary Tables 17 and 18. The evolution of

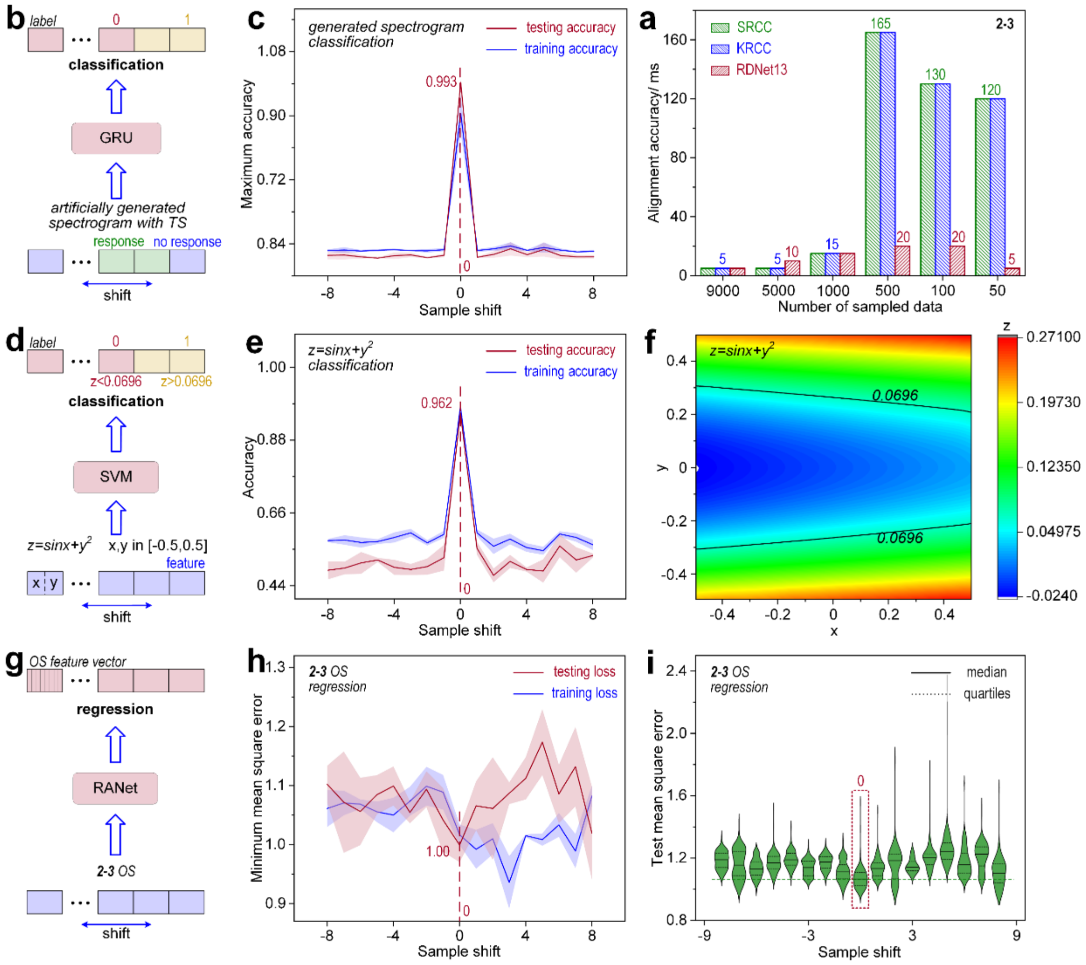

Figure 4. **Analysis of alignment architecture promotion.** **a** Comparison of alignment accuracy between the RDNet13 kernel model and frequency domain similarity, using the Spearman rank correlation coefficient (SRCC) or the Kendall rank correlation coefficient(KRCC). **b-c** Schematic diagrams and alignment results based on the gate recurrent unit (GRU) kernel model used to align the arc response data from the arc igniter with the corresponding labels. **d-e** Schematic diagrams and alignment results of aligning the input of artificially constructed functions and corresponding labels on the basis of the support vector machine (SVM) kernel model. **f** Cloud diagram of the corresponding constructed functions. **g-h** Schematic diagram and alignment results of aligning optical signals (OS) in dataset **2-3** and feature vectors of the OS via the RANet kernel model. **i** The corresponding distribution of testing accuracy across different sample shifts.

numerical modeling has undergone three major paradigm shifts. The first stage is defined by numerical fitting methods, such as least squares, which rely on predefined basis functions. The second stage introduces traditional machine learning algorithms, including support vector machines (SVMs), which eliminate the need for explicit basis functions but operate within limited solution spaces. The third and current stage is characterized by deep learning algorithms, which are initiated by models from the AlexNet era and are capable of learning complex patterns directly from data. Compared with earlier modeling paradigms, the training of deep learning models offers significantly expanded paths for identifying optimal solutions. Using AlexNet as a representative example, the process begins with weight initialization, followed by progressive feature transformation stages that enrich representation through dimensional expansion and spatial reduction of feature maps. Hierarchical connectivity across layers further enhances the expressive capacity of the model, enabling the learning of increasingly abstract features. During training, loss functions guide parameter updates by

reinforcing features that improve task performance, thereby systematically directing the model toward accurate classification or regression outcomes. The neural network–based alignment architecture reflects the characteristics of the third modeling paradigm, which involves solving for the target kernel model (KM) through multiple adaptive strategies involving feature aggregation and enrichment, thereby achieving high-quality alignment. In contrast, the frequency domain similarity–based algorithm resembles the paradigms of the first and second stages, employing relatively fixed rules that rely solely on frequency-domain vector similarities. This fundamental limitation explains its significantly lower accuracy than neural network–based alignment when working with a limited number of samples. However, algorithms that rely on relatively fixed approaches empowered by relevant domain knowledge to find solution functions are still recommended because they consume relatively low algorithmic costs. However, in the absence of such prior knowledge, neural networks offer clear advantages. Deep learning models with strong expressive capacity can flexibly adapt to end-to-end learning across diverse data types, including tabular data, images, semantic vectors, and time series. The primary trade-offs of neural network–based approaches include increased demand for data and computational resources. Nonetheless, when domain knowledge is unavailable, the cost associated with acquiring large-scale data and supporting sufficient computational power becomes a necessary investment.

The supplemented contents are in "Supplementary Discussion 10. The alignment method based on frequency domain similarity" section are shown as:

The implementation and results of the frequency domain similarity-based alignment method are shown in Supplementary Figs. 26a–c. To address the challenge of defining explicit correlation functions between heterogeneous data modalities, an approach leveraging intramodal similarity was proposed under the assumption that spectrograms corresponding to the same label category—such as arc response and no arc response—exhibit inherent similarity. As shown in Supplementary Fig. 26a, for the optical signal (OS) modality, similarity is quantified by computing the coincidence accuracy between the OS label sequence and the offset OS label sequence. For the acoustic signal (AS) modality, the time-domain signal of each segment is first transformed into the frequency domain. To ensure comparability, the amplitude of the frequency-domain signal is scaled such that the sum of the amplitudes remains constant. Inspired by the geometric principle that the square in a rectangle with equal perimeter yields the maximum area, the similarity between two signals is quantified via the dot product between the scaled frequency-domain vectors, where an increasing dot product denotes greater similarity between two signals. For the AS segment sequence, the original AS sequence corresponds to the time shift, whereas each offset AS sequence corresponds to the superposition of the time shift and offset. The offset-label coincidence accuracy curve, derived from the OS sequence, and the offset-frequency domain similarity curve, obtained from the AS sequence across different time shifts, are shown in Supplementary Fig. 26b. Suppose that a given time shift accurately reflects accurate semantic alignment. In that case, the positive correlation between the label coincidence accuracy and frequency domain similarity at that time shift should be stronger than that observed at other time shifts. The mathematical formulation of this algorithm is shown in Supplementary Equation (1):

$$\left\{\begin{aligned} S_y[t_{\rm of}] &= \sum_{n=1}^{N} M(y_1[n,0], y_1[n,t_{\rm of}])/N \\ S_{\rm fft}[t_{of},t] &= \sum_{n=1}^{N} k\cdot \mathbf{F}_{2,\rm norm}^{\rm fft}[n,t] \bullet \mathbf{F}_{2,\rm norm}^{\rm fft}[n,t_{\rm of}+t] \\ c_{\rm Sr}[t] &= 1-\frac{6\cdot \sum_{t_{\rm of}=-T_{\rm of}}^{T_{\rm of}} d_n^2(S_y[t_{\rm of}],S_{\rm fft}[t_{\rm of},t])}{N^3-N} \\ c_{\rm Kr}[t] &= \frac{C(S_y[t_{\rm of}],S_{\rm fft}[t_{\rm of},t])-D(S_y[t_{\rm of}],S_{\rm fft}[t_{\rm of},t])}{\frac{1}{2}\cdot N\cdot(N-1)} \\ t_{\rm Sr} &= \max_t c_{\rm Sr}[t] \\ t_{\rm Kr} &= \max_t c_{\rm Kr}[t] \\ T_{\rm Sr} &= t_{\rm Sr}\cdot T_p \\ T_{\rm Kr} &= t_{\rm Kr}\cdot T_p \end{aligned}\right\} t_{\rm of}\in Z,\ y_1[n,t]\in\{0,1\} \quad (1)$$

where $y_1[n,0]$, $S_y[t_{\rm of}]$, $\mathbf{F}_{2,\rm norm}^{\rm fft}[n,t]$, and $S_{\rm fft}[t_{of},t]$ denote the label obtained from signal $x_1[n,0]$, the label coincidence accuracy between sequences $\{y_1[0,0], y_1[1,0],\cdots, y_1[N-1,0]\}$ and sequences $\{y_1[0,t_{\rm of}], y_1[1,t_{\rm of}],\cdots, y_1[N-1,t_{\rm of}]\}$, the scaled frequency-domain vectors of signal $x_2[n,t]$ (where the sum of the elements of each vector is equal), and the frequency-domain similarity between sequences $\{\mathbf{F}_{2,\rm norm}^{\rm fft}(0,t),\ \mathbf{F}_{2,\rm norm}^{\rm fft}(1,t),\ \cdots,\ \mathbf{F}_{2,\rm norm}^{\rm fft}(N-1,t)\}$ and $\{\mathbf{F}_{2,\rm norm}^{\rm fft}(0,t+t_{\rm of}),\ \mathbf{F}_{2,\rm norm}^{\rm fft}(1,t+t_{\rm of}),\ \cdots,\ \mathbf{F}_{2,\rm norm}^{\rm fft}(N-1,t+t_{\rm of})\}$, respectively. $c_{\rm Sr}[t]$ and $c_{\rm Kr}[t]$ denote the Spearman rank coefficient and the Kendall rank coefficient, respectively. d^n denotes the rank difference between the matching degree sequences $\{S_y[-T_{\rm of}],\ S_y[-T_{\rm of}+1],\ \cdots,\ S_y[T_{\rm of}]\}$ and the similarity sequence $\{S_{\rm fft}[-T_{\rm of},t],\ S_{\rm fft}[-T_{\rm of}+1,t],\ \ldots,\ S_{\rm fft}[T_{\rm of},t]\}$. $C(S_y[t_{\rm of}],S_{\rm fft}[t_{\rm of},t])$ and $D(S_y[t_{\rm of}],S_{\rm fft}[t_{\rm of},t])$ denote the logarithms of the consistent and inconsistent elements between sequences $\{S_y[-T_{\rm of}],\ S_y[-T_{\rm of}+1],\ \cdots,\ S_y[T_{\rm of}]\}$ and $\{S_{\rm fft}[-T_{\rm of},t],\ S_{\rm fft}[-T_{\rm of}+1,t],\ \ldots,\ S_{\rm fft}[T_{\rm of},t]\}$, respectively. Here, $T_{\rm of}$ is set to 8. $t_{\rm Sr}$ and $t_{\rm Kr}$ denote the semantic alignment parameters between the semantic sequences of signals $x_1[n,0]$ and $x_2[n,0]$ calculated on the basis of the Spearman rank correlation coefficient and the Kendall rank correlation coefficient, respectively. $T_{\rm Sr}$ and $T_{\rm Kr}$ are the corresponding semantic time shifts, and the search range of the semantic time shift is $t\cdot T_p$. The pseudocode corresponding to the above algorithm is shown in Algorithm 2.

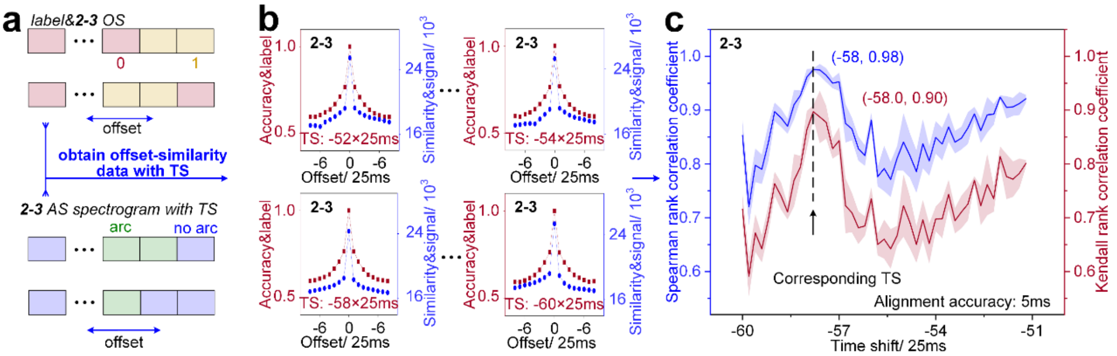

Supplementary Figure 26. **The alignment method on the basis of frequency domain similarity. a** Implement schematic of the alignment method. **b** Offset-accuracy and similarity curves under various time shifts (TSs). **c** Alignment results based on the method between optical signals (OS) and acoustic signals (AS).

The sampling methods and ratios employed in Supplementary Figs. 26b-c are consistent with those utilized in Figs. 3g-i, with 9,000 samples analyzed for the former. The time shift–level correlation coefficient curve generated by the proposed algorithm is presented in Supplementary Fig. 26c. Both the Spearman and the Kendall rank correlation coefficients reach their extrema at a time shift of -58.0×25 ms, indicating that the semantic time shift t_o^a is -58.0 × 25 ms with an alignment accuracy of 5 ms. This estimated semantic time shift lies within the expected reference range and is consistent with the outcome derived from the proposed

neural network-based alignment architecture. Furthermore, large-scale data analysis confirms that spectrograms exhibit substantial similarity within the arc response category, whereas those that display clear distinctions between arc and no-arc responses.

Comment 10:

All experiments are limited to aluminum and copper alloy friction pairs. Information on whether the model was evaluated on other materials or geometries would be useful to understand generalization.

Answer of comment 10:

Thanks very much for your valuable comment. Based on your comment, we have supplemented the optical and acoustic signal datasets from 3-1 to 3-3 corresponding to different material friction pairs and inclined friction conditions in "Supplementary Discussion 2. Datasets used for the alignment study" section, and supplemented the corresponding alignment experiments in "Supplementary Discussion 4. Repetitive and supplementary alignment" section to demonstrate the generality of the proposed architecture.

The supplemented contents are in "Supplementary Discussion 2. Datasets used for the alignment study" section are shown as:

Line 180, Revised Manuscript: To further assess the generalizability of the proposed architecture, the disc materials in experiments from **3-1** to **3-3** were replaced with CuBe, CuSn, and CuZn alloys.

Supplementary Table 3. **The dataset numbering and corresponding conditions of the orthogonal experiments**

Number	Monitor signal	Material	Load mode	Electric current / A	Rotational speed/ r/min	Time/ s
**1-1-1**	OS	CrZrCu alloy	CDL	2	50	1000
**1-1-2**	OS	CrZrCu alloy		2	150	
**1-1-3**	OS	CrZrCu alloy		8	50	
**1-1-4**	OS	CrZrCu alloy		8	150	
**1-2-1**	OS	CrZrCu alloy	CLL	2	50	
**1-2-2**	OS	CrZrCu alloy		2	150	
**1-2-3**	OS	CrZrCu alloy		8	50	
**1-2-4**	OS	CrZrCu alloy		8	150	
**1-3-1**	OS	CrZrCu alloy	CMLL	2	50	
**1-3-2**	OS	CrZrCu alloy		2	150	
**1-3-3**	OS	CrZrCu alloy		8	50	
**1-3-4**	OS	CrZrCu alloy		8	150	
**2-2**	OS,IS and AS	CrZrCu alloy	CLL	2、5、8	50、100、150	900
**2-3**	OS,IS and AS	CrZrCu alloy	CMLL			
**3-1**	OS and AS	CuBe alloy				
**3-2**	OS and AS	CuSn alloy				
**3-3**	OS and AS	CuZn alloy				

The supplemented contents are in "Supplementary Discussion 4. Repetitive and supplementary alignment" section are shown as:

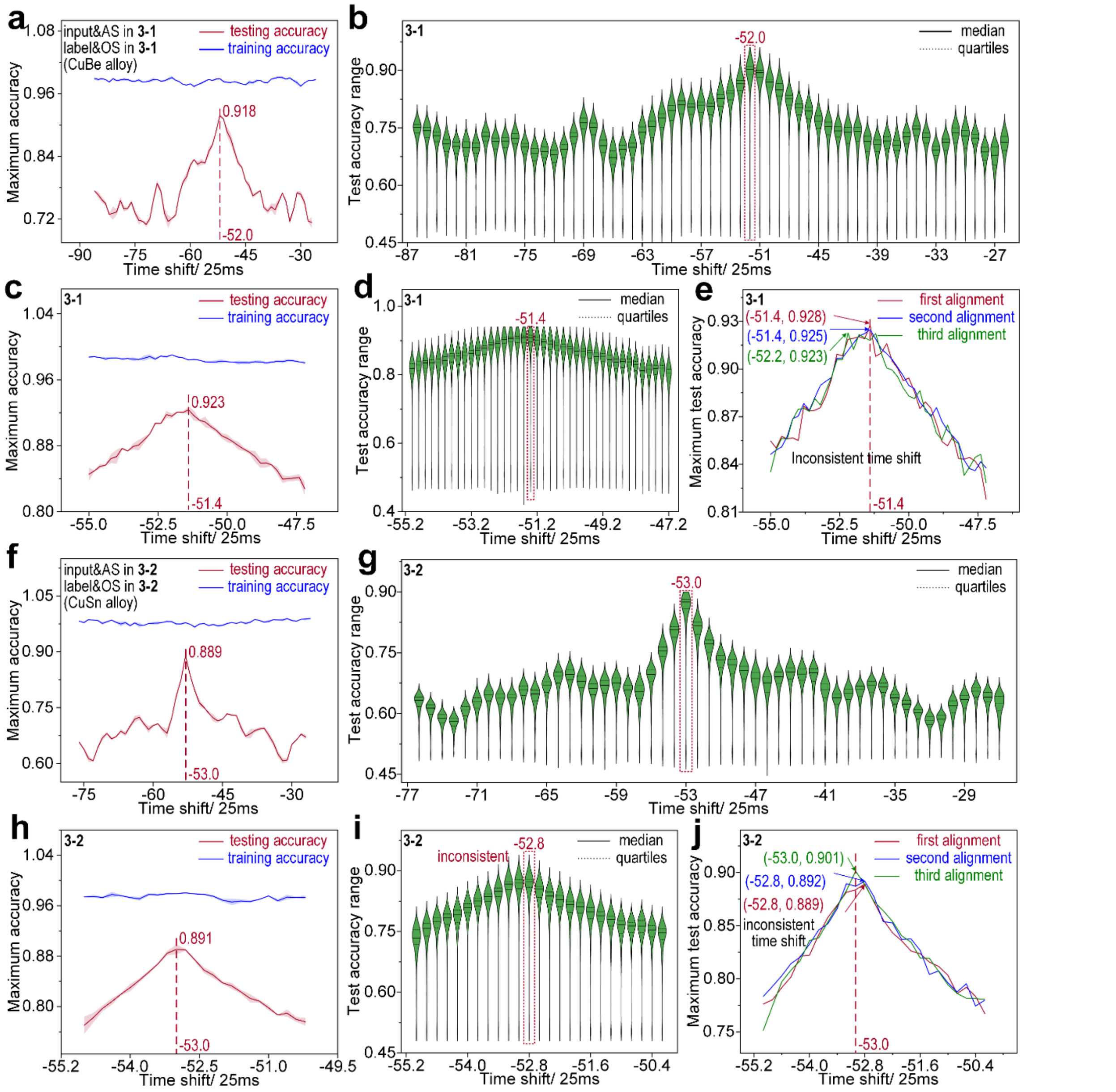

Supplementary Figure 15. **The time shift-maximum accuracy curve and accuracy distribution corresponding to the alignment of optical signals (OS) and acoustic signals (AS) in datasets 3-1 and 3-2.** **a-b** The time shift (step size: 25 ms)-maximum accuracy curve and time shift (step size: 25 ms)-testing accuracy distribution for dataset **3-1** alignment, respectively. **c-d** Time shift (step size: 5 ms)-maximum accuracy curve and time shift (step size: 5 ms)-testing accuracy distributions in the dataset **3-1** alignment, respectively. **e** Time shift–maximum testing accuracy curve corresponding to 3 repeated alignments in dataset **3-1**. **f-g** Time shift (step size: 25 ms)-maximum accuracy curve and time shift (step size: 25 ms)-testing accuracy distributions for dataset **3-2** alignments. **h-i** Time shift (step size: 5 ms)-maximum accuracy curve and time shift (step size: 5 ms)-testing accuracy distributions in the dataset **3-2** alignment, respectively. **j** Time shift–maximum testing accuracy curve corresponding to 3 repeated alignments in dataset **3-2**.

To further demonstrate the effectiveness of the proposed architecture, alignment between the optical signal (OS) and acoustic signal (AS) from experiments with different current-carried friction pairs (**3–1** and **3–2**) and

friction modes (**3–3**) was conducted. The friction pairs in dataset **3–1** are 7075 Al alloy and CuBe alloy, with the alignment results shown in Supplementary Figs. 15a–e. The semantic TS t_o^a for this dataset is -51.4×25 ms. In the dataset **3–2** , the friction pairs are 7075 Al alloy and CuSn alloy, with the alignment results presented in Supplementary Figs. 15f–j, and the semantic TS t_o^a is -53.0×25 ms.The alignment results of OS and AS for dataset **3-3** are shown in Supplementary Figs. 16c–h, with an alignment parameter t_o^a of -49.2×25 ms. For datasets from **3-1** to **3-3**, the obtained alignment parameter is close to the reference range from -51.00×25 ms to -49.00×25 ms derived from the photoacoustic response test. Notably, in addition to the alignment of dataset **3-3** (inclined friction in Supplementary Fig. 16a), the comparison of the alignment results from datasets **2-3**, **3-1**, and **3-2** in the four vertical friction tests (Supplementary Fig. 16b) with the corresponding reference range of TSs suggests that the semantics of the AS tend to lag behind those of the synchronous OS. Therefore, the acoustic response following the arc response may serve as a basis for detecting the arc response via the AS.

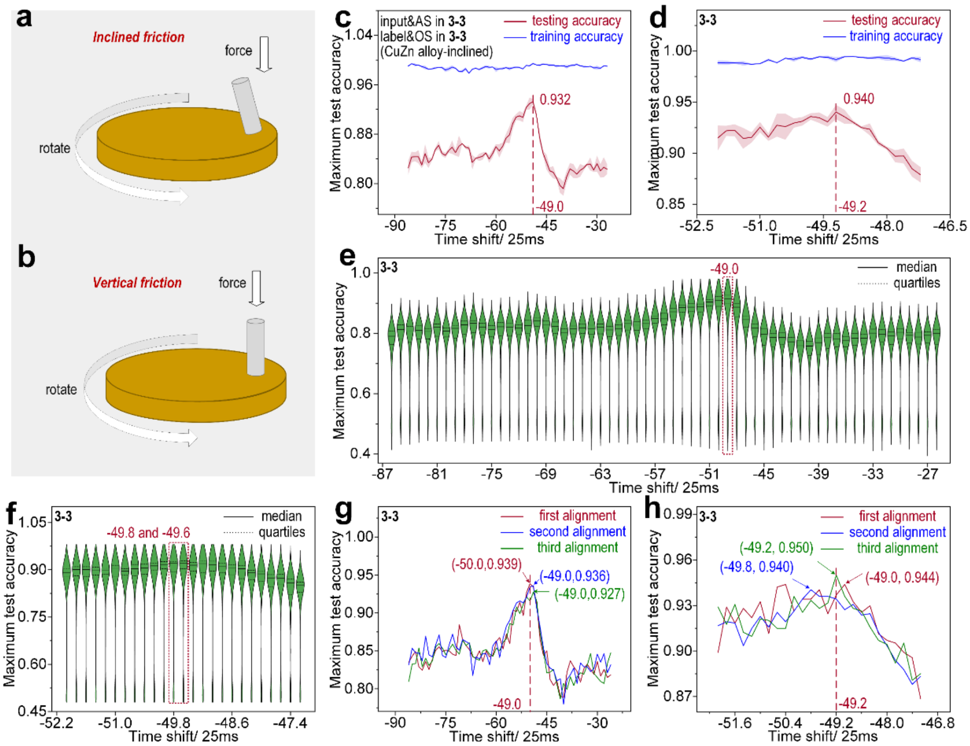

Supplementary Figure 16. **Friction form of the test specimens and alignment results of the optical signals (OS) and acoustic signals (AS) in dataset 3-3 of the experiments. a** Schematic diagram of inclined friction in dataset **3-3**. **b** Schematic diagram of vertical friction in other datasets. **c-d** Time shift (time step: 25 or 5 ms)-maximum accuracy curve for **3-3** sets of alignments. **e-f** Time shift (step: 25 or 5 ms)-testing accuracy distributions for **3-3** sets of alignments. **g-h** Time shift (step: 25 or 5 ms)-maximum testing accuracy curve corresponding to 3 repeated alignments in dataset **3-3**.

Comment 11:

The paper does not address whether the measured time shifts include physical delays such as acoustic propagation time. Clarification on whether these delays were measured and accounted for would improve interpretation.

Answer of comment 11:

Thanks very much for your valuable comment. Based on your comment, we have supplemented the

evaluation of physical in the "Supplementary Discussion 5. Interpretability analysis of the acoustic signals" section. If observing more transient phenomena, physical delays will significantly affect the semantic time shift between signals. Therefore, we have supplemented the reasons for the semantic time shift between signals from different sources in the "Abstract" section.

The supplemented and revised contents are in "Abstract" section are shown as:

Via cross-correlation algorithms or synchronized acquisition of signals, the alignment of heterogeneous data with unknown semantic time shifts and intermittent semantic variations cannot be solved. The shift is caused by different data acquisition principles of sensors, different response discrimination principles using heterogeneous data, etc.

The supplemented and revised contents are in "Supplementary Discussion 5. Interpretability analysis of the acoustic signals" section are shown as:

Line 440, Supplementary Information: Moreover, in this case, the delay between the OS and AS, caused by the inherent difference in the propagation speeds of light and sound, is approximately 0.5 ms, which has only a minor influence on the overall system.

Comment 12:

Class activation maps are shown qualitatively without detailed analysis. Quantification of which frequency bands contributed most to arc detection would improve interpretability.

Answer of comment 12:

Thanks very much for your valuable comment. Based on your comment, we have supplemented the more detailed analysis of the class activation mapping in the "Supplementary Discussion 5. Interpretability analysis of the acoustic signals" section, including the quantified frequency range. Meanwhile, we also supplemented Supplementary Movie 4 and Movie 5 to showcase more class activation maps.

The supplemented and revised contents are in "Supplementary Discussion 5. Interpretability analysis of the acoustic signals" section are shown as:

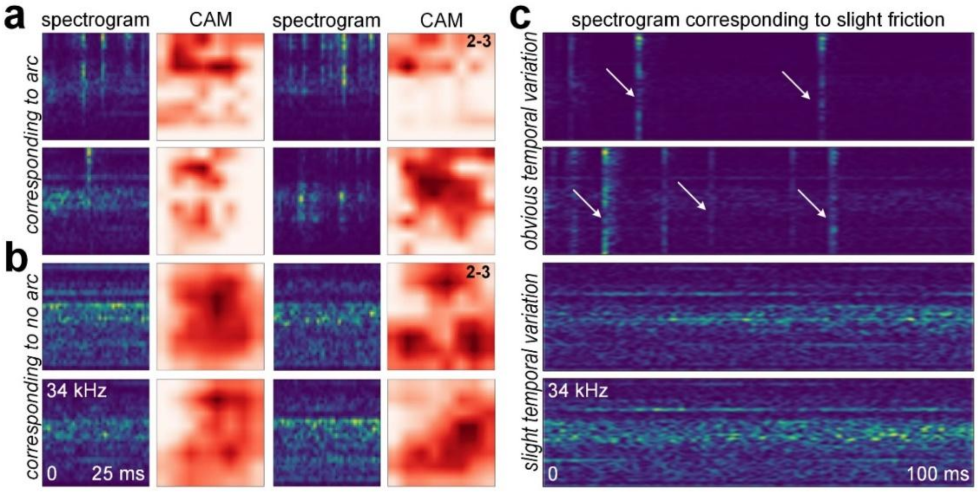

Supplementary Figure 17. **Interpretability analysis of typical acoustic signals. a-b** Normalized spectrograms of the acoustic signals selected via the signal alignment method and the calculated class activation maps (CAMs) under conditions of arc damage and no arc damage. **c** Typical normalized spectrogram of the acoustic signals under the condition of slight contact (current: 8 A) between two friction specimens (Supplementary Fig. 16.). (a, b and c show multiple sets of spectrograms with the same semantics to more fully demonstrate the common features of the signal.)

Line 411, Supplementary Information: The arc damage in current carrying friction seems to be related to the spectrogram response in the range of 8-34 kHz, whereas the corresponding spectrogram without arc damage shows a relatively strong response in the range of 8-22 kHz. More CAM cloud maps are provided in Supplementary Movie 4 and Supplementary Movie 5.

Comment 13:

Computational demands and real-time feasibility are not discussed. Information on the computational time required for alignment and prediction, and whether the approach is feasible for real-time monitoring, would be relevant.

Answer of comment 13:

Thanks very much for your valuable comment. Based on your comment, we have supplemented the time cost estimation of the alignment model in Supplementary Tables 17 and 18. Additionally, we have also supplemented the analysis of the training inference time for the arc detection model in the "Supplementary Discussion 8. Cost evaluation of the arc detection model" section and discussion of the time cost of the architecture in the "Supplementary Discussion 12. Cost evaluation of alignment architecture" section.

The supplemented contents are in "Supplementary Discussion 8. Cost evaluation of the arc detection model" section are shown as:

To assess the feasibility of deploying the arc detection model based on optical signals (OSs), acoustic signals (ISs), or acoustic signals (ASs) on an in-situ testing system for real-time monitoring, the inference time was measured via Python implementation, as detailed in Supplementary Table 16. The total inference time comprises two parts: data preprocessing and model inference. For arc detection using OS as input, the total time required for dynamic threshold adaptation preprocessing and inference with the RSNet model across 36,000 samples is 88.77 seconds. In contrast, IS-based arc detection involves neither preprocessing nor deep learning model inference, resulting in a substantially lower computational cost than the OS-based approach. For AS-based arc detection via the RDNet41 model, the total processing time for 36,000 samples is 893.8 s. All three detection methods exhibit inference times that are shorter than the corresponding test duration of 900 s, thereby fulfilling the real-time requirements for arc discrimination in the in-situ testing system. Moreover, for deployment scenarios demanding greater efficiency, implementing the models in "C" language can further reduce the computational cost of arc detection.

Supplementary Table 14. **The time cost of signal preprocessing**

(Not included in the time for data reading, data storage, and data segmentation)

Data preprocessing method	Time cost for processing each signal segment/s
Convert AS from time domain into the 1 M spectrogram form	3.453×10^{-2}
Convert AS from time domain into the 34 kHz spectrogram form	2.434×10^{-2}
Convert AS from time domain into the 1 MHz frequency domain form	4.156×10^{-3}
Convert OS based on the dynamic threshold adaptive	1.347×10^{-3}
Convert OS based on the histogram equalization	5.155×10^{-3}

Supplementary Table 15. **Training time costs of machine learning models**

(Not included in the time for data reading, data storage, and data segmentation)

Model	Samples number	Epoch	Training time per epoch/ s	Testing time per epoch/ s	Total training time/s
GRU	9000	200	4.157	4.034	1638
MLP		100	9.855×10^{-3}	3.621×10^{-2}	13.48
MNet2_1			8.225	3.619	1184
MNet2_2			8.532	3.755	1229
MNet2_4			8.411	3.645	1206
MNet2_8			8.623	3.710	1233
MNet2_16			8.364	3.566	1193
MNet2_32			8.650	3.660	1231
MNet2_64			8.999	4.124	1312
MNet3			9.190	3.954	1314
MNet4			9.109	4.006	1312
RANet		50	64.35	26.59	4547
RDNet13		100	8.878	3.732	1261
RDNet41			9.848	4.391	1424
RMNet			9.881	4.105	1399
RRNet10			11.16	4.683	1584
RRNet18			8.921	3.728	1265
RSNet (3 channels input)	10134	100	72.24	29.89	10213
RSNet (1 channels input)			28.08	11.34	3942
SVM	600	/	/	/	1.557×10^{-2}

Supplementary Table 16. **The inference time costs for the deployed model to detect the arc**

(Inference on the basis of Supplementary Tables 14 and 15)

Input signal	Model	Samples number	Data preprocessing time/ s	Inference time of model/ s	Total inference time/s
AS	MNet2_1	36000	876.2	14.48	890.7
AS	MNet2_2			15.02	891.3
AS	MNet2_4			14.58	890.8
AS	MNet2_8			14.84	891.1
AS	MNet2_16			14.26	890.5
AS	MNet2_32			14.64	890.9
AS	MNet2_64			16.50	892.7
AS	MNet3			15.82	892.1
AS	MNet4			16.02	892.3
AS	RDNet13			14.93	891.2
AS	RDNet41			17.56	893.8
AS	RMNet			16.42	892.7
AS	RRNet10			18.73	895.0
AS	RRNet18			14.88	891.1
OS	RSNet (3 channels input)		0 or 185.6	106.2	106.0 or 291.8
OS	RSNet (1 channels input)		48.49	40.28	88.77

The supplemented "Supplemented Tables 17 and 18" are shown as:

Supplementary Table 17. **The time costs of alignment based on RDNet13**

(Inference on the basis of Supplementary Tables 14 and 15)

Samples number	Data preprocessing time/ s	Training time per search steps/s	Search steps	Total time/ s
9000	1095	1261	15	20010
5000	608.5	716.5	15	11356
1000	121.7	139.2	15	2209.7
500	60.85	65.97	15	1050.4
100	12.17	12.50	15	199.67
50	6.085	7.037	15	111.64

Supplementary Table 18. **The time costs of alignment on the basis of frequency domain similarity**
(Inference on the basis of Supplementary Tables 14 and 15)

Samples number	Data preprocessing time/ s	The time for caculate rank correlation coefficient for per search step/s	Search steps	Total time/ s
9000	187.0	230.9	45	10578
5000	103.9	133.3	45	6102.4
1000	20.78	28.84	45	1318.6
500	10.39	15.34	45	700.69
100	2.078	3.162	45	144.37
50	1.039	1.806	45	82.309

The supplemented contents are in "Supplementary Discussion 12. Cost evaluation of alignment architecture" section are shown as:

The computational time required for training a kernel model is provided in Supplementary Table 15. In a fixed semantic time-shift system, the one-time alignment cost of the proposed architecture remains acceptable. In typical machine learning workflows, identifying semantic features that facilitate the extraction of critical information can enhance the performance of traditional, lightweight models. For systems that require frequent alignment, the support vector machine-based kernel model is computationally feasible when it is applied to small sample sizes within limited search ranges. Computational power is widely recognized as the cornerstone of the Fourth Industrial Revolution. In this context, the demand for quantum computing has grown increasingly urgent [15-17], driven by the application of artificial intelligence in the fields of science and industry. Similarly, for systems that require continuous alignment, deploying alignment models built upon a more complex kernel model demands substantial computational resources. The alignment requirements can vary significantly across systems. For systems with low real-time demands, fast kernel model convergence, or offline alignment needs, the applicability and efficiency of the proposed architecture should be evaluated on a case-by-case basis.

Reviewer #4

This article proposes a model for the current carrying friction scenario, which is still meaningful for this scenario. The experimental design, application of neural network methods, and final result verification of the article are all quite rigorous. The overall writing and structural arrangement are also relatively reasonable, but there are still the following issues that need further consideration. Overall, this manuscript is well-written and the proposed method is clearly implemented.

Comment 1:

In the current field of current carrying friction for monitoring acousto-optic electromagnetic signals, is there a method to detect the signal, or is there any problem if it is not aligned? This should be supplemented in the introduction.

Answer of comment 1:

Firstly, thank you for your appreciation of our work. Thanks very much for your valuable comment. Based on your comment, we have revised and supplemented the corresponding contents in the "1. Introduction" section to indicate that aligning the input and output of the model is a prerequisite for obtaining high-quality detection models and the limitation of classic alignment method including cross-correlation and supervised learning algorithms based on cross-correlation algorithms. As shown in the time shift-test accuracy curve presented in this article, if the optical signal and acoustics are not aligned, the performance of the acoustic signal measurement model trained based on such data will decrease.

The revised contents are in "1. Introduction" section are shown as:

Line 34, Revised Manuscript: However, whether the input and output of the model are aligned semantically is a prerequisite for the effective application of the optimization function.

The term "alignment" is commonly used in three contexts: alignment between the source and target domains in transfer learning [27–29], alignment in multimodal transformation and fusion [30–32], and alignment of signals along the timeline [33–36]. With respect to transfer learning, the essence of domain generalization lies in aligning the features of the source and target domains to leverage information from the source domain, thereby enhancing model performance on tasks in the target domain [29,37-42]. With respect to multimodal domains, alignment is fundamental to the effective fusion of multimodal information [31]. In multimodal learning, alignment involves projecting data from different modalities into a shared semantic space, where semantically similar concepts are placed closer together, thus facilitating the integration of diverse information sources [32]. Similarly, the goal of temporal signal alignment is to ensure semantic consistency between corresponding segments across signals. While hardware-based synchronization is the preferred approach, the differing acquisition principles of heterogeneous sensors and the varying discrimination based on heterogeneous data often limit its applicability. In such cases, cross-correlation analysis [43-45], is considered. However, cross-correlation analysis often requires prior domain knowledge, such as understanding the positive or negative correlation between signal features. To enhance applicability, supervised learning has been employed to derive transformation functions that generate effective features for alignment [46]. A significant limitation of this approach is the reliance on manually annotated semantic time shifts between signals as the gold standard for training[46]. Aligning heterogeneous data without domain knowledge is challenging [47], especially when semantic time shifts are unknown and semantic variations are transient [48, 49]. First, the unknown semantic time shifts hinder the extraction of manually annotated semantic time shifts and effective alignment features [46]. Furthermore, transient semantic variations further hinder the establishment of associations between heterogeneous data.

Comment 2:

Since it is aimed at the current carrying friction scenario, the title of the paper should be limited, otherwise it is unknown whether this data alignment method is applicable or its generalization performance can be extended in other scenarios.

Answer of comment 2:

Thanks very much for your valuable comment. Based on your comment, we supplemented the alignment experiment of the dataset based on the preset time shift in the"2.4. Promotion of alignment architecture" section and the "Supplementary Discussion 11 Supplement of alignment architecture promotion" section no illustrate the generality of the alignment architecture proposed in this article. At the same time, we have also made corresponding modifications to the title to more accurately describe the application scenarios of the proposed architecture and the challenges we have solved.

The revised contents are in "title" section are shown as:

Machine Learning-driven Alignment Architecture of Heterogeneous Data with Transient Varying Semantics

The supplemented contents are in "2.4. Promotion of alignment architecture" section are shown as:

To further evaluate the applicability and reliability of the proposed alignment architecture, extensive experiments were performed on datasets with predetermined alignment parameters. For time series data, to accelerate the experimental process, both the AS spectrograms and the corresponding labels were synthetically generated on the basis of the arc response patterns of the arc igniter. The construction procedure is illustrated in Supplementary Fig. 27, and the detailed algorithm for generating data is provided in Supplementary Algorithm 3. On the basis of the gated recurrent unit (GRU) KM, alignment experiments were conducted on the generated spectrograms and corresponding labels (Fig. 4b), with the sample shift search range set from -8 to +8. In this setup, the frequency response intensity vectors at each time point in the spectrogram were used as feature vectors input into the time series model. The alignment results are shown in Fig. 4c, and the distribution of testing accuracy across different sample shifts is presented in Supplementary Fig. 28. Both the training accuracies and testing accuracies reached their maximum values at a sample shift of 0, indicating that the alignment architecture successfully achieved correct alignment between the time series data and the corresponding labels. To further validate the applicability of the proposed alignment architecture to tabular data, a synthetic dataset was constructed, referring to recent research [52], via the function $z = \sin x + y^2$, where x and y were sampled within the range of -0.5 --+0.5. Data points with $z > 0.0696$ were assigned a label of 1, whereas those with $z \leq 0.0696$ were assigned a label of 0 (Fig. 4d). The feature vectors composed of x and y, and the corresponding labels, were aligned via a support vector machine (SVM) KM (Fig. 4d). The alignment results shown in Fig. 4e demonstrate the favorable alignment performance of the architecture. In the multimodal era driven by transformer -based architectures, semantic vectors have become the primary medium for cross-modal information exchange. To evaluate the applicability of the proposed alignment framework in such contexts, alignment was performed between dataset **2-3** OS images and their corresponding 8-dimensional semantic feature vectors (mean, maximum, minimum, range, variance, skewness, kurtosis, and entropy) via the RANet KM (Fig. 4g). The alignment results are presented in Figs. 4h and 4i. While the training mean squared error (MSE) did not reach its minimum at a sample shift of 0, the testing MSE achieved its lowest value at this point. Moreover, the overall distribution of the testing MSE at a sample shift of 0 was lower than that at other shifts, indicating successful alignment between the OS images and their associated semantic representations. The sample shift–testing MSE curves corresponding to the three alignment trials (Figs. 4g–i) are presented in Supplementary Fig. 29. Among the three, one instance exhibited suboptimal alignment performance, with an alignment resolution exceeding 8 sample shifts. This discrepancy

may be attributed to internal balancing mechanisms among the outputs of the multioutput neural network. To increase the robustness of alignment in such challenging scenarios, multiple alignment runs are recommended to increase the reliability of the estimated semantic time shift. Additionally, supplementary alignment experiments are presented in Supplementary Discussion 11, including the alignment between images and labels, tabular data and labels, and input vectors and output values of artificially constructed functions. All the experiments achieved favorable alignment performance. In summary, at the task level, the proposed alignment architecture is applicable not only to regression tasks of KMs but also to classification tasks. At the model level, the architecture accommodates a wide range of model architectures, including traditional machine learning models, time series models, and convolutional neural networks. At the data level, the architecture supports alignment across diverse modalities, including tabular data, time series data, and image data, with corresponding semantic labels or feature vectors. Therefore, this alignment architecture is expected to be applicable to align heterogeneous data pairs in which one modality allows the extraction of semantic labels, semantic vectors, and continuous semantic values. Finally, the time cost of alignment based on the proposed architecture is analyzed in detail in Supplementary Discussion 12.

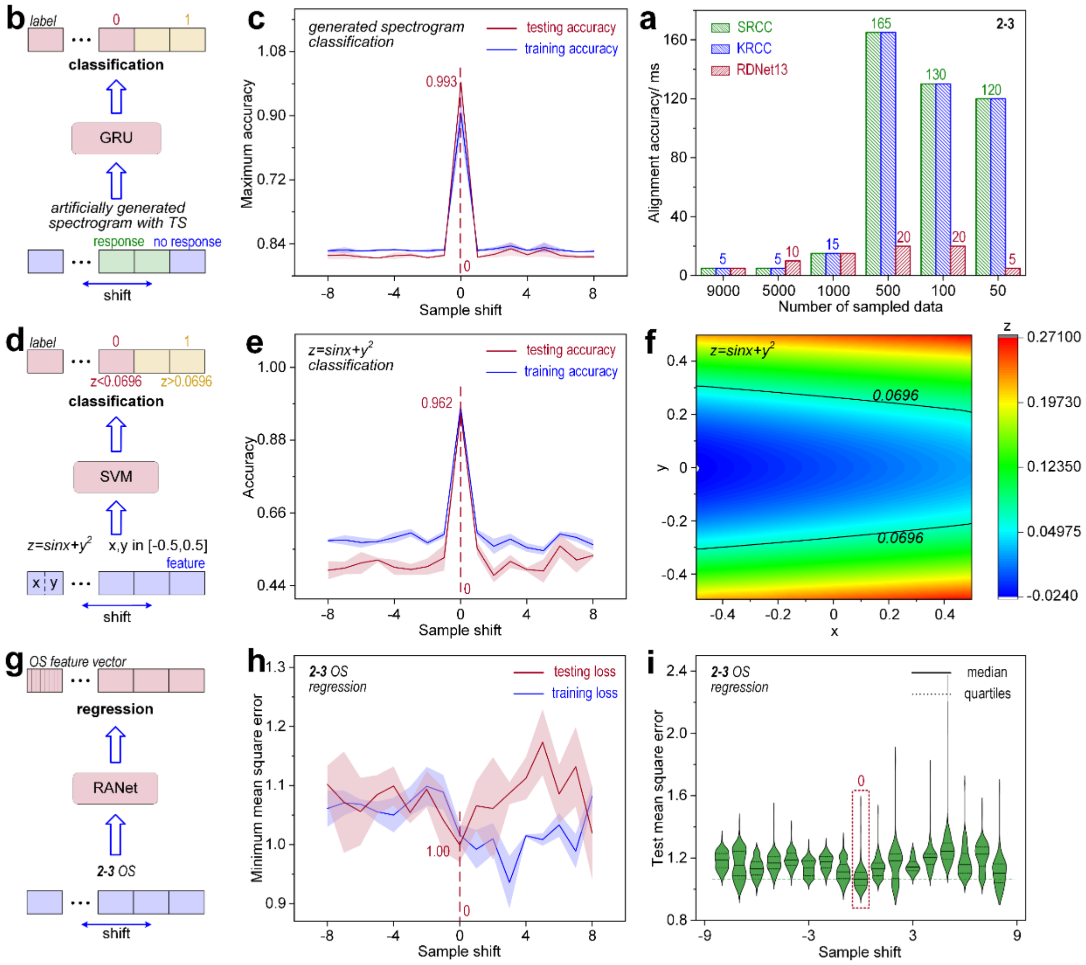

Figure 4. **Analysis of alignment architecture promotion. a** Comparison of alignment accuracy between the RDNet13 kernel model and frequency domain similarity, using the Spearman rank correlation coefficient (SRCC) or the Kendall rank correlation coefficient(KRCC). **b-c** Schematic diagrams and alignment results based on the gate recurrent unit (GRU) kernel model used to align the arc response data from the arc igniter with the corresponding labels. **d-e** Schematic diagrams and alignment results of aligning the input of artificially constructed functions and corresponding labels on the basis of the support vector machine (SVM) kernel model. **f** Cloud diagram of the corresponding constructed functions. **g-h** Schematic diagram and alignment results of aligning optical signals (OS) in dataset **2-3** and feature vectors of the OS via the RANet kernel model. **i** The corresponding distribution of testing accuracy across different sample shifts.

The supplemented contents are in "Supplementary Discussion 11. Supplement of alignment architecture promotion" section are shown as:

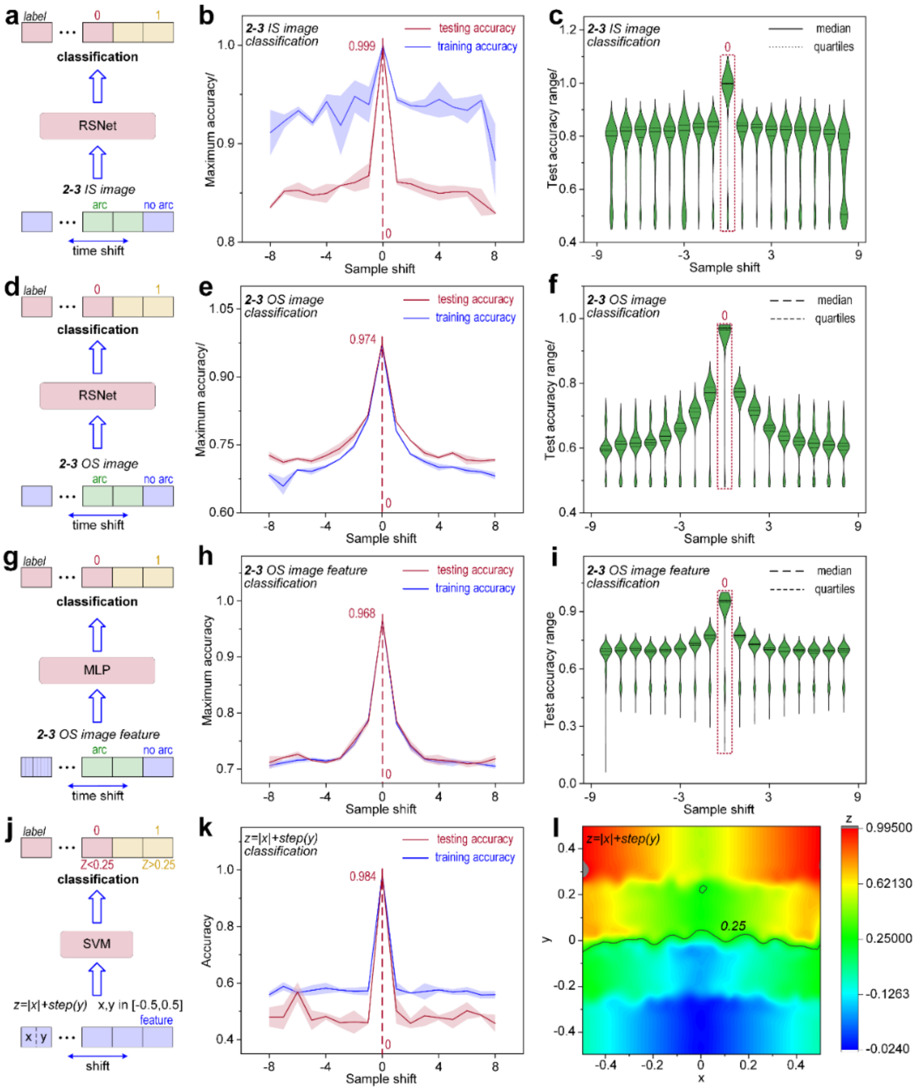

Supplementary Figure 30. **Promotion of the alignment architecture (supplements for Figure 4). a** Schematic diagram of aligning dataset **2-3** infrared signals (IS) with their labels via the RSNet kernel model. **b-c** Sample shift-maximum accuracy curve and testing accuracy distribution corresponding to a, respectively. **d** Schematic diagram of aligning dataset **2-3** optical signals (OS) with their labels via the RSNet kernel model. **e-f** Sample shift-maximum accuracy curves and accuracy distributions corresponding to d, respectively. **g** Schematic diagram of the alignment of dataset **2-3** feature vectors of the OS with their labels via multilayer perceptron (MLP) kernel model. **h-i** The shift-maximum accuracy curve and testing accuracy distribution corresponding to g, respectively. **j-k** Schematic diagram of aligning the input of artificially constructed functions with the corresponding output via the support vector machine (SVM) kernel model and corresponding alignment results. **l** Cloud diagram of the constructed functions in j.

For image data, alignment via the RSNet kernel model was performed between infrared signal images and corresponding labels in dataset **2-3** (Supplementary Figs. 30a-c), as well as between optical signal (OS) images and their corresponding labels in dataset **2-3** (Supplementary Figs. 30d-f), both of which achieved favorable alignment results. For tabular data, eight statistical features—mean, maximum, minimum, range, variance, skewness, kurtosis, and entropy—were extracted from dataset **2-3** OS images to form 8-dimensional feature

vectors. The alignment between these vectors and their corresponding labels was performed via a multilayer perceptron (MLP) kernel model (Supplementary Figs. 30g–i). The model achieved maximum training and testing accuracy at a sample shift of 0, confirming the suitability of the proposed alignment architecture for structured tabular data. Additionally, the input vectors of the function $z = |x| + \mathrm{step}(y)$ and corresponding labels based on the support vector machine (SVM) were successfully aligned (Supplementary Figs. 30j-l). Subsequently, alignments between the input vectors and outputs of the above two functions were performed via a regression-based kernel model (Supplementary Fig. 31). Both models receive a two-dimensional feature vector composed of x and y as inputs and predict the corresponding z values. Both the training and testing mean squared errors reached their minimum at a sample shift of 0 for the two models, demonstrating that the proposed alignment architecture is also applicable to regression tasks.

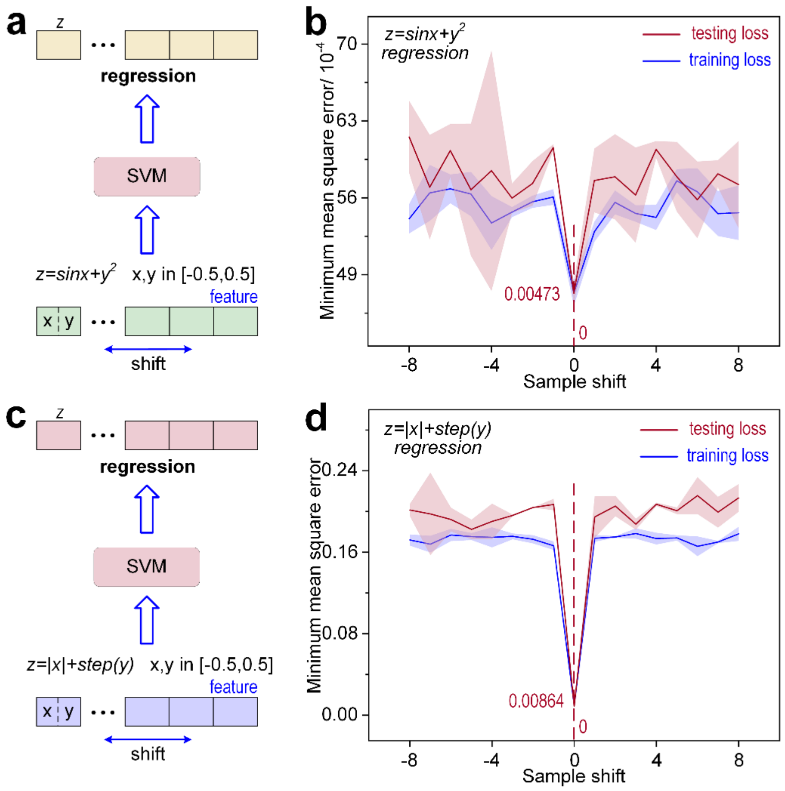

Supplementary Figure 31. **Promotion of the alignment architecture (supplements for Fig. 4). a-b** Schematic diagram of aligning the input of artificially constructed functions ($z = \sin x + y^2$) with the corresponding output via the support vector machine (SVM) kernel model and corresponding alignment results. **c-d** Schematic diagram of aligning the input of artificially constructed functions ($z = |x| + \mathrm{step}(y)$) with the corresponding output via the SVM kernel model and corresponding alignment results.

Comment 3:

The article mentions that the proposed method requires "relatively high-quality training data" to work effectively, which poses a challenge to the engineering practicality of the proposed method. It is suggested to supplement or explain this part slightly.

Answer of comment 3:

Thanks very much for your valuable comment. In the previous version, this vague explanation involved explaining the reason for the failure of alignment between infrared signals and acoustic signals. Based on your comment, we supplemented the alignment experiments with different numbers of sample as well as reasons

for failed alignment of infrared signals and acoustics in the “2.3. Factors affecting alignment accuracy” section.

The supplemented contents are in “2.3. Factors affecting alignment accuracy” section are shown as:

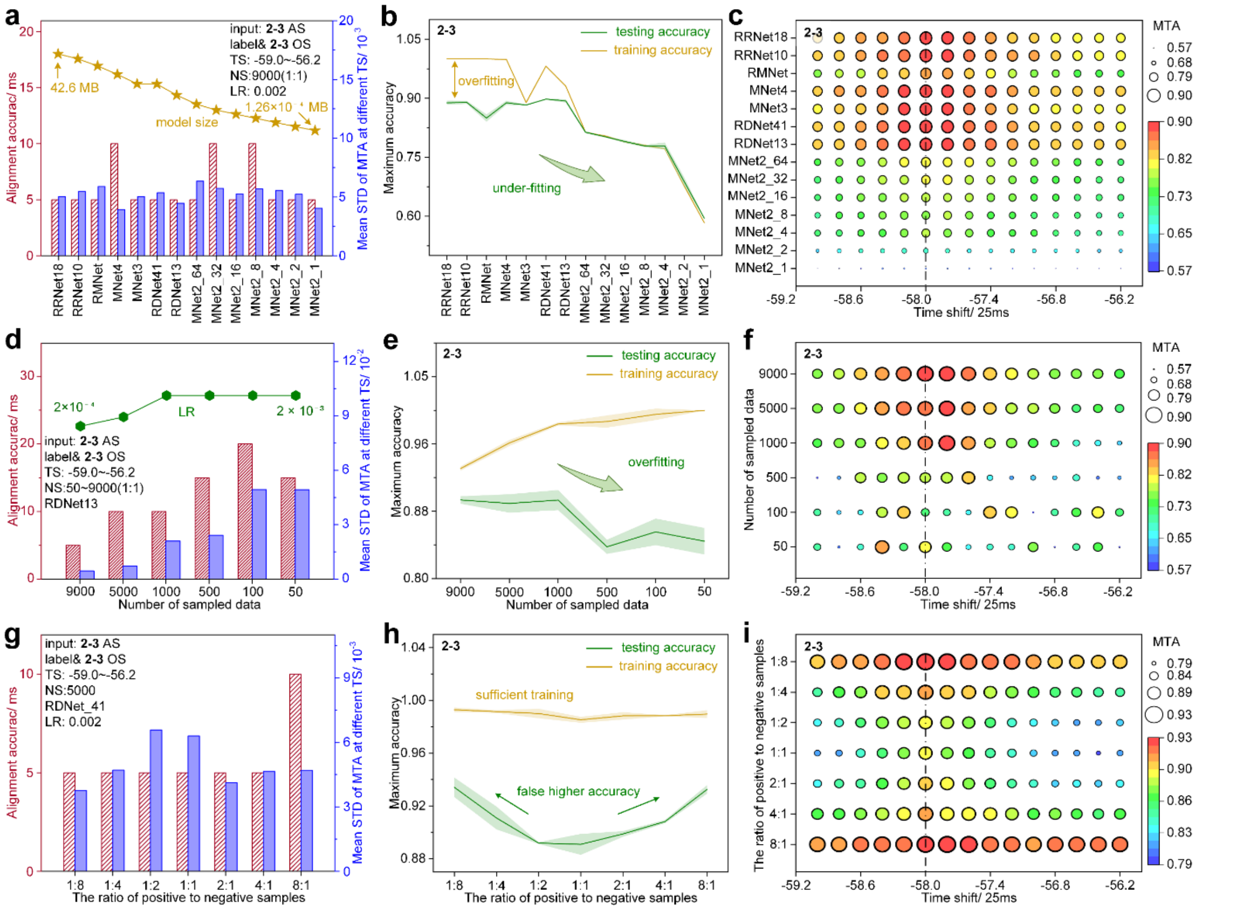

Figure 3. **Analysis of factors affecting alignment accuracy. a** Variance in alignment accuracy and standard deviation (STD) of the maximum testing accuracy (MTA) across different kernel models under the same learning rate (LR). **b** Maximum training and testing accuracy across different kernel models. **c** Bubble chart of the MTA across different kernel models and time shifts (TSs) (step size: 5 ms). **d** Alignment accuracy of the RDNet13 kernel models as the number of samples (NS) varies. **e** Corresponding NS–maximum accuracy curve. **f** Corresponding bubble chart of the MTA across different TSs (step size: 5 ms). and NS. **g** Variation in alignment accuracy with the proportion of positive to negative samples. **h** Maximum accuracy across different training sample proportions. **i** Corresponding bubble chart of MTA across different TSs (step size: 5 ms) and training sample proportions.

Figs. 3d–3f illustrate the impact of the NS on alignment accuracy. When the same kernel model is employed for alignment across varying NSs, the models trained with fewer samples exhibit more pronounced underfitting. This underfitting is reflected in the reduced testing accuracy, which in turn undermines the credibility of the alignment. Therefore, the KM was trained using learning rates that increased from 2×10^{-4} at an NS of 9000 to 2×10^{-3} at an NS of 50. The learning rate is negatively correlated with the NS (Fig. 3d). The alignment accuracy deteriorates from 5 ms at an NS of 9000 to 20 ms at an NS of 50. Concurrently, the average STD of the MTA across TSs increases as the NS decreases. With decreasing NS, the kernel model in the semantic TS exhibits increased overfitting and diminished generalization capability, resulting in decreased credibility of the alignment result, as shown in Fig. 3e. As shown in Fig. 3f, as the NS decreases, the trend of peak-shaped variation in the MTA near the semantic TS becomes less pronounced. In other words, the correlation—whether

positive or negative—between the MTA on either side of the semantic TS and the TS weakens as the NS decreases. The typical epoch-loss and accuracy curves of the kernel model corresponding to Fig. 3f are shown in Supplementary Fig. 23. The KM exhibits an unstable training process when the NS falls below 500. Under stable training conditions, a higher STD in the MTA across TSs reflects a greater sensitivity of accuracy to temporal variation, which is advantageous for alignment. However, in the case of unstable training, an increased STD (Fig. 2d) does not contribute to improved alignment, because the variability primarily arises from training instability rather than from meaningful temporal sensitivity. Additionally, when aligning the OS with the IS via the RDNet13 kernel and a limited NS of 50, an alignment accuracy of 15 ms was achieved, as shown in Fig. 3d. As illustrated in Fig. 1f, the arc events extracted from both the IS and OS demonstrate a temporally consistent distribution. The failed alignment between the IS and AS, as shown in Fig. 2a, is likely due to inaccuracies in the local time axis of the IS captured by the thermal imager, rather than the limited number of IS images (1800) associated with the arc. The effect of NS on alignment accuracy based on the extremely lightweight kernel model Mnet2_1 is similar to that of RDNet13, as presented in Supplementary Discussion 9. Moreover, the maximum degradation in alignment resolution within the range of 50-9000 samples is limited to just 15 ms. Therefore, in the initial stages of the large-scale search for alignment parameters, adopting a coarse alignment strategy based on a small NS and lightweight KMs is a cost-effective and practical solution.

Comment 4:

The current method seems to have no technical issues in achieving monitoring data alignment, but how economical is it? Should this be discussed in the article, such as the cost of the entire monitoring system.

Answer of comment 4:

Thanks very much for your valuable comment. Based on your comment, we supplemented the price estimate for the entire testing system in "Supplementary Discussion 1. Instrument and alignment requirements" section. In addition, high-speed cameras, infrared thermal imaging, and acoustic emission sensors are universal equipment that can also be used for other monitoring tasks in flexible material data factories. And the parameters used in this article are not the highest parameters that there devices can support, such as sampling frequency.

The supplemented contents are in "Supplementary Discussion 1. Instrument and alignment requirements" section are shown as:

Supplementary Table 2. **The estimated cost of the revised in-situ testing system**

Modules of the testing system	The estimated cost
Self-developed current-carrying friction testing instrument	50,000 RMB
High-speed camera (Revealer X213)	400,000 RMB
Infrared thermal imaging instrument (Telops Spark M150)	600,000-800,000 RMB
AE sensor and supporting acquisition system (PAC Wsa/AF62)	150,000 RMB
Total	1,200,000-1,400,000 RMB

To clearly demonstrate the significance and applicability of the heterogeneous data alignment architecture, a case study on high-throughput detection of the arc damage frequency under current-carried friction conditions, using optical signals (OSs), infrared signals (ISs) and acoustic signals (ASs), is introduced. The abbreviations and full names of the technical terms used in this paper are provided in Supplementary Table 1

Reviewer #5

The manuscript introduces a neural network-based method for aligning ultra-fast optical signals (images) with one-dimensional acoustic signals, applied in the context of arc damage detection during current-carrying friction tests. The authors integrate a high-speed optical imaging system, an infrared (IR) thermal camera, and acoustic emission (AE) sensors into a tribological test rig and conduct orthogonal experiments varying load, current, and speed. The proposed alignment approach uses a deep convolutional network (a modified DenseNet-121) to determine the time offset between optical and acoustic signal streams. By feeding time-shifted acoustic data into the network and using optical-signal-derived labels, they identify the time shift that yields the highest model validation accuracy as the correct alignment parameter. Using this method, the authors report that they can align the optical and acoustic signals with ~5 ms accuracy.

Comment 1:

While the manuscript presents an interesting multimodal alignment framework, the overall presentation suffers from a lack of clarity. Key concepts such as the alignment challenge, deep learning workflow, and result interpretation are not introduced or developed in a systematic and reproducible manner. Figures are inconsistently labelled, and several abbreviations are used without prior definition. For improved readability and reproducibility, I strongly recommend the authors revise the structure to include: (i) a clearly defined problem statement early on; (ii) a step-by-step methodology section including model architecture, training procedure, and data labelling; and (iii) consistently labelled and explained figures with defined acronyms. Further details are provided in the specific comments below.

Answer of comment 1:

Thanks very much for your valuable comment. Based on your comment and official requirements of the journal, we have thoroughly revised the entire text. The detailed items for specific modifications are summarized as follows:

1. For the challenge of alignment, we have supplemented the relevant content related to the alignment challenge and optimized the logic in the "1. Introduction" section and the "2.1. Alignment requirements and architecture" section. For the scenario involving the alignment of heterogeneous signals with unknown time shifts and transiently varying semantics, we reviewed other classical alignment methods that do not apply to this scenario. We proposed a heterogeneous data alignment architecture driven by machine learning to address this challenge.
2. For the workflow, we have supplemented equations, pseudocode, figures, and definitions for labels to clearly demonstrate the workflow in the "2.1. Alignment requirements and architecture" section. Sections "2.2. Alignment verification and applications" and "2.3. Factors affecting alignment accuracy" are instantiation of section 2.1, while section "2.4. Promotion of alignment architecture" is an extension of section 2.1.
3. For the explanation of the results, in the last paragraph of the "2.2. Alignment verification and applications" section, we have supplemented the reasons why the maximum testing accuracy at semantic time shifts is higher than that at other time shifts. For the "2. Results and discussion" section, we also adopted a writing style of presenting and discussing the results at the same time for supplementation and modification.
4. For the reproducibility of the results, we have supplemented the "Methods" section.
5. For the citation of figures, we have checked and corrected the citation in the revised paper.
6. For unclear abbreviations, we have supplemented "Supplementary Table 1. Abbreviations and full names of the technical terms" for readers to inquire about their meanings. Meanwhile, we have indicated the full name of the abbreviation where abbreviation appears for the first time in each section.

The specific modifications are shown in the revised manuscript, supplementary information and subsequent “**Answer of comment**” section.

Comment 2:

The manuscript should more clearly articulate the motivation and technical challenge of aligning acoustic signals with optical/infrared images. It would help readers unfamiliar with sensor fusion to understand why alignment is necessary despite simultaneous data acquisition, and what specific temporal or physical desynchronizations this method aims to resolve.

Answer of comment 2:

Thanks very much for your valuable comment. Based on your comment, we have supplemented the challenges of alignment, motivation for architecture, and sources of time shift in the “Abstract” section, the “1. Introduction” section, and the “2.1. Alignment requirements and architecture” section.

The revised contents are in “Abstract” section are shown as:

Via cross-correlation algorithms or synchronized acquisition of signals, the alignment of heterogeneous data with unknown semantic time shifts and intermittent semantic variations cannot be solved. The shift is caused by different data acquisition principles of sensors, different response discrimination principles using heterogeneous data, etc.

The revised contents are in “1. Introduction” section are shown as:

The term "alignment" is commonly used in three contexts: alignment between the source and target domains in transfer learning [27–29], alignment in multimodal transformation and fusion [30–32], and alignment of signals along the timeline [33–36]. With respect to transfer learning, the essence of domain generalization lies in aligning the features of the source and target domains to leverage information from the source domain, thereby enhancing model performance on tasks in the target domain [29,37-42]. With respect to multimodal domains, alignment is fundamental to the effective fusion of multimodal information [31]. In multimodal learning, alignment involves projecting data from different modalities into a shared semantic space, where semantically similar concepts are placed closer together, thus facilitating the integration of diverse information sources [32]. Similarly, the goal of temporal signal alignment is to ensure semantic consistency between corresponding segments across signals. While hardware-based synchronization is the preferred approach, the differing acquisition principles of heterogeneous sensors and the varying discrimination based on heterogeneous data often limit its applicability. In such cases, cross-correlation analysis [43-45], is considered. However, cross-correlation analysis often requires prior domain knowledge, such as understanding the positive or negative correlation between signal features. To enhance applicability, supervised learning has been employed to derive transformation functions that generate effective features for alignment [46]. A significant limitation of this approach is the reliance on manually annotated semantic time shifts between signals as the gold standard for training[46]. Aligning heterogeneous data without domain knowledge is challenging [47], especially when semantic time shifts are unknown and semantic variations are transient [48, 49]. First, the unknown semantic time shifts hinder the extraction of manually annotated semantic time shifts and effective alignment features [46]. Furthermore, transient semantic variations further hinder the establishment of associations between heterogeneous data. Consequently, a robust and unsupervised alignment method is urgently needed to overcome the associated challenges. Given the successful use of neural networks for alignment in transfer learning and multimodal domains [27–32, 37–42], a critical research question arises: can a novel neural network-driven architecture be designed to address the challenges of heterogeneous data

alignment with unknown semantic time shifts and intermittent semantic variation?

The supplemented contents are in "2.1. Alignment requirements and architecture" section are shown as:

Line 102, Revised Manuscript: However, in this case, defining F_1^{tf} and F_2^{tf} is challenging because of the unclear positive correlation between certain features of $x_1[n,t]$ and $x_2[n,t]$. Moreover, supervised learning is not applicable for determining F_1^{tf} and F_2^{tf} [46] because of the unknown and inherently ambiguous nature of the true semantic TS between $x_1[n,0]$ and $x_2[n,0]$. Addressing these challenges necessitates a robust unsupervised algorithm or architecture, the development of which demands further analytical and experimental investigation.

Comment 3:

The manuscript lacks an explanation for why a DenseNet-121-based CNN was chosen for the alignment and classification tasks. This leaves the impression that the choice might be arbitrary. The authors should clarify why this architecture is suitable or discuss comparisons to other models. Without this, readers may question whether a simpler or traditional method could have achieved similar results. Given the novelty of the alignment idea, it would strengthen the paper to justify the use of a deep CNN.

Answer of comment 3:

Thanks very much for your valuable comment. Based on your comment, we have supplemented the impact of different kernel neural networks on alignment accuracy in the "2.3. Factors affecting alignment accuracy" sections.

The supplemented contents are in "2.3. Factors affecting alignment accuracy" section are shown as:

In systems with a constant semantic time shift (TS), synchronous hardware triggering is applied to ensure the simultaneous acquisition of heterogeneous signals. Simultaneously, the TS can be estimated once during an initial calibration using the alignment architecture and subsequently reused, resulting in minimal computational cost. In contrast, in systems with a varying semantic time shift—such as those affected by time-varying delays—the alignment architecture must be further optimized to reduce the computational cost. Fig. 3 illustrates the alignment accuracy between OS and AS in dataset **2-3**, evaluated across different kernel models (KMs), the number of samples (NS), and positive-to-negative sample ratios. Fig. 3a presents the alignment accuracy for various KM sizes, including RRNet18 and RRNet10, with KM sizes ranging from 42.6 MB to 1.26×10^{-4} MB. The smallest model (MNet2_1) consists of only two layers and a single channel. As the KM size decreases, the alignment accuracy fluctuates under time resolutions of 5 ms and 10 ms, with a higher frequency of occurrence at 5 ms (Fig. 3a). Contrary to the intuitive expectation that the standard deviation (STD) of maximum testing accuracy (MTA) across different TSs systematically increases with reduced KM size, no consistent negative correlation between these two variables is observed. Fig. 3b shows the mean of the maximum training accuracy or the MTA across the KMs. Reducing the KM size corresponds to a clear transition from overfitting to underfitting. Despite the emergence of underfitting, the alignment accuracy remains unaffected. The corresponding bubble plot in Fig. 3c illustrates the relationships among the KMs, TS, and MTA. For each model, the MTA initially increases with TS, reaches a peak near the semantic TS, and subsequently decreases. Notably, this peak-like extremum remains pronounced even as the KM size decreases, demonstrating that alignment accuracy remains significantly unaffected by the KM size. The overall training process maintains relative stability despite potential underfitting tendencies in small kernel models, as corroborated by the epoch-accuracy and loss curves in Supplementary Fig. 22.

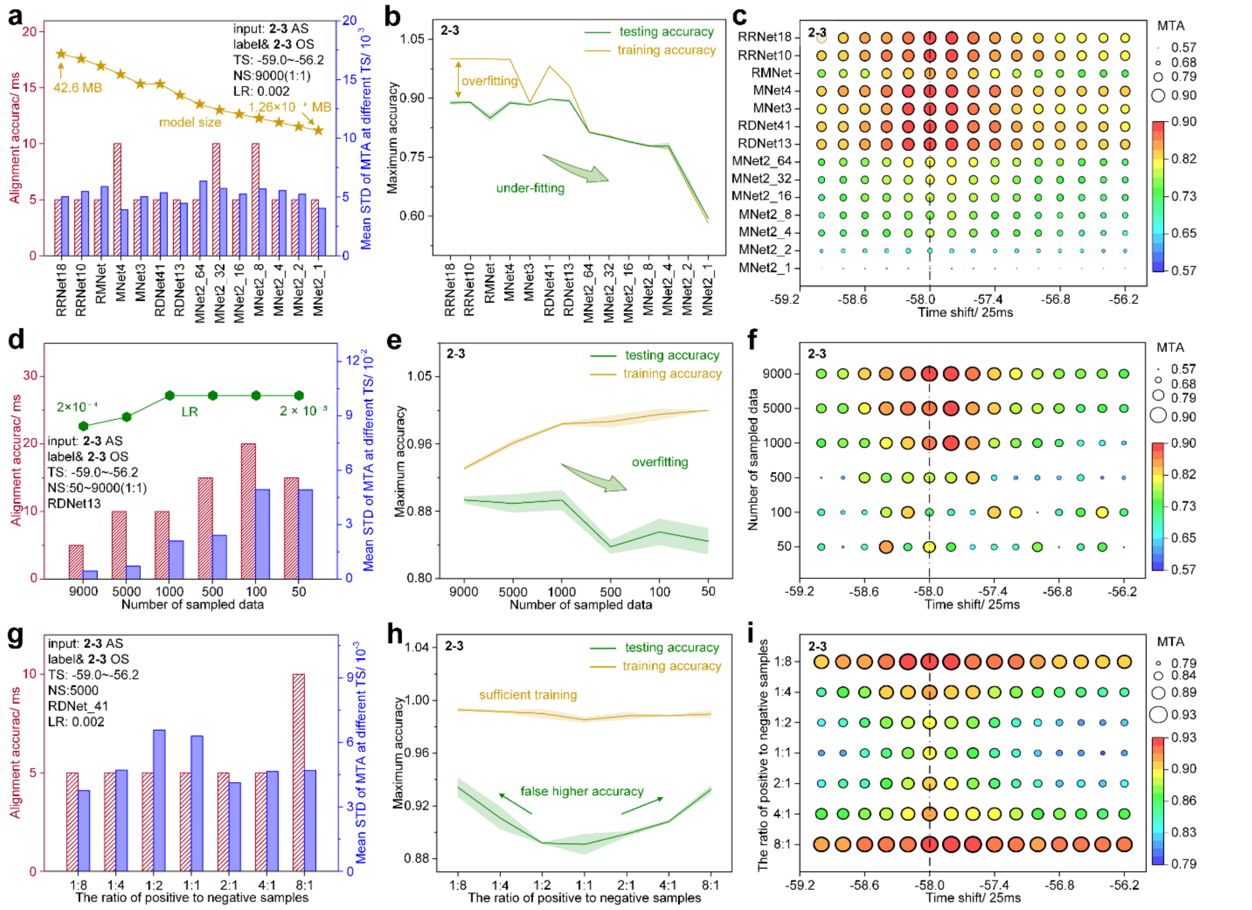

Figure 3. **Analysis of factors affecting alignment accuracy. a** Variance in alignment accuracy and standard deviation (STD) of the maximum testing accuracy (MTA) across different kernel models under the same learning rate (LR). **b** Maximum training and testing accuracy across different kernel models. **c** Bubble chart of the MTA across different kernel models and time shifts (TSs) (step size: 5 ms). **d** Alignment accuracy of the RDNet13 kernel models as the number of samples (NS) varies. **e** Corresponding NS–maximum accuracy curve. **f** Corresponding bubble chart of the MTA across different TSs (step size: 5 ms). and NS. **g** Variation in alignment accuracy with the proportion of positive to negative samples. **h** Maximum accuracy across different training sample proportions. **i** Corresponding bubble chart of MTA across different TSs (step size: 5 ms) and training sample proportions.

Comment 4:

While the authors suggest that the optimal time shift is determined by identifying the maximum classification accuracy of a DenseNet model, the methodology is under-specified. It remains unclear how acoustic data are pre-processed into segments, how labels are assigned across shifts, and whether DenseNet is trained separately for each shift or evaluated using a sliding window strategy. Furthermore, the rationale and validity behind using peak classification accuracy as a surrogate for signal alignment require clearer justification and empirical support. I recommend the authors provide a structured description of: (i) training data generation, (ii) model input/output, (iii) shift-wise evaluation protocol, and (iv) how maximum accuracy maps to optimal alignment.

Answer of comment 4:

Thanks very much for your valuable comment. The workflow is indeed difficult to express clearly through textual description alone. Based on your comment, we have supplemented Fig.1a, Equations 1 and 3, and Supplementary Algorithm 1 as well as related descriptions of these contents in “2.1. Alignment requirements and architecture” section. These contents include cutting methods for acoustic signals, label assignment rules, input and output of kernel models, shift-wise evaluation protocol and the mapping relationship between maximum test accuracy and optimal alignment. For more detailed signal processing methods, please refer to the “Methods” section. For the principle of alignment, we have supplemented the principle of the alignment architecture in the “2.2. Alignment verification and applications” section.

The supplemented contents are in “2.1. Alignment requirements and architecture” section are shown as:

To illustrate the significance of aligning heterogeneous signals, the arc damage frequency is detected under current-carried friction conditions via optical signals (OSs), infrared signals (ISs), and acoustic signals (ASs), as presented in Supplementary Discussion 1. The abbreviations and full names of the technical terms used in this paper are provided in Supplementary Table 1 and will not be repeated in the following text. The alignment between AS and OS or IS serves as a prerequisite for extracting meaningful information from the AS. Data segmentation is essential for accurate signal alignment. OS and IS images are temporally integrated spectral information over fixed intervals, inherently limiting flexibility for temporal segmentation. In contrast, AS, which is a high-frequency one-dimensional waveform, supports fine-grained and precise temporal segmentation. Accordingly, the AS can be adaptively segmented to align with the fixed temporal windows of OS or IS images. The AS was segmented according to the acquisition time T_s of each image. The OS or IS images were subsequently randomly sampled into the N group at a 1:1 ratio of the arc response to non-arc response classes, and the corresponding AS segments were extracted. The resulting signal segments are represented by Equation (1):

$$\left.\begin{cases} x_1[n,t] = \mathbf{A}^{n,t} \\ x_2[n,t] = \{a_1^{n,t}, a_2^{n,t}, \cdots, a_s^{n,t}\} \end{cases}\right\} n \in \{0, 2, \cdots, N-1\} \quad (1)$$

where $x_1[n,t]$ and $x_2[n,t]$ denote the nth two-dimensional time series signal with a time shift (TS) t in the randomly sampled signal segment and the corresponding one-dimensional time series signal segment, respectively. The corresponding time length of signal segments $x_1[n,t]$ and $x_2[n,t]$ is T_s. In this case, $\mathbf{A}^{n,t}$ represents an OS or IS image, and $x_2[n,t]$ is a one-dimensional AS segment with amplitude $a_s^{n,t}$ and length s. The segmentation details of the signal segment and the symbol definition rules of Equation (1) are shown in Fig. 1a. For signal alignment, cross-correlation algorithms are commonly employed, as shown in Equation (2):

$$\begin{cases} t_c = \max_t \sum_{n=1}^{N} F_1^{tf}(x_1[n,0]) \cdot F_2^{tf}(x_2[n,t]) \\ T_c = t_c \cdot T_s \end{cases} \quad (2)$$

where T_c and t_c denote the semantic TS and semantic alignment parameters, respectively, between the sequences of signal segments $x_1[n,0]$ and $x_2[n,0]$ obtained via the cross-correlation algorithm, F_1^{tf} and F_2^{tf} denote the corresponding transformation functions of $x_1[n,t]$ and $x_2[n,t]$, respectively; and the search range for semantics is $t \cdot T_p$. However, in this case, defining F_1^{tf} and F_2^{tf} is challenging because of the unclear positive correlation between certain features of $x_1[n,t]$ and $x_2[n,t]$. Moreover, supervised learning is not applicable for determining F_1^{tf} and F_2^{tf} [46] because of the unknown and inherently ambiguous nature of the true semantic TS between $x_1[n,0]$ and $x_2[n,0]$. Addressing these challenges necessitates a robust unsupervised algorithm or architecture, the development of which demands further analytical and experimental investigation.

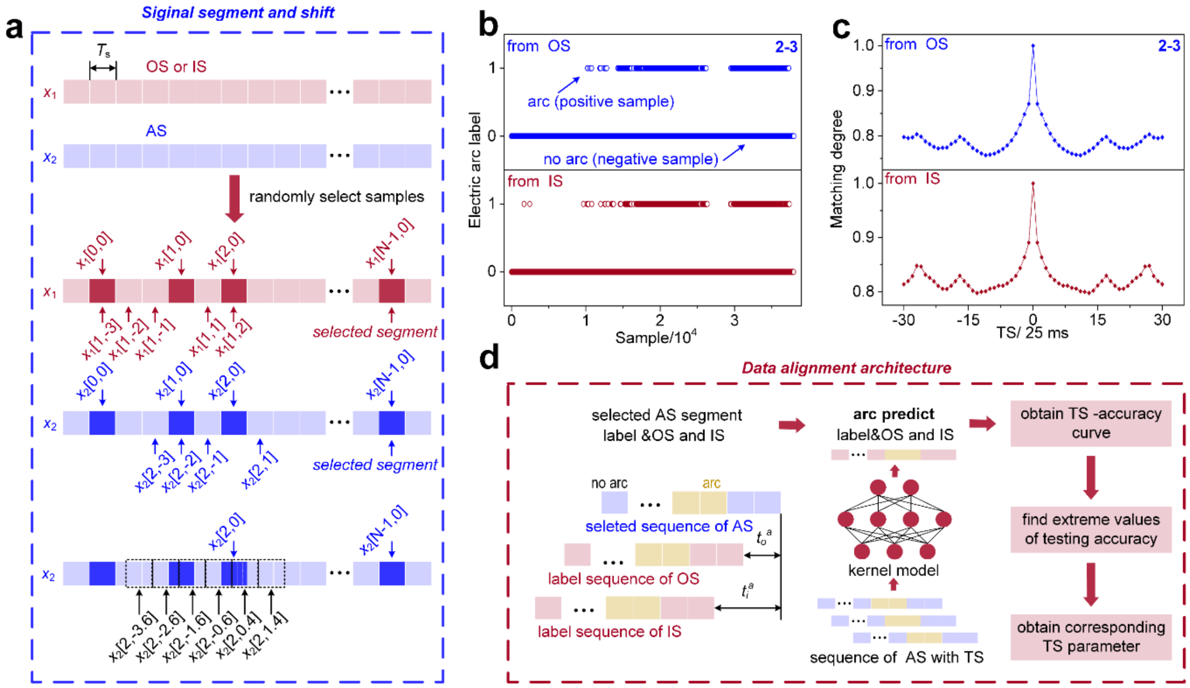

Figure 1. **The alignment architecture for heterogeneous signals. a** Process of random sampling and symbol definition of signal segments. **b** Label obtained from infrared signals (IS) and optical signals (OS) to determine arc damage, **c** Time shift (TS)-accuracy curve of the labels. **d** The alignment architecture and its application flow.

The labels ("1" denotes a positive sample corresponding to the arc, and "0" denotes negative samples corresponding to the absence of arc) obtained from the IS and OS (Fig. 1b) were subsequently subjected to a time-shift process to generate time-shifted labels. These labels were compared with the original label to construct a TS-matching degree curve (Fig. 1c). The accuracy was optimal (100%) in the absence of TS, but declined sharply with increasing TS near 0 TS. As the TS increased further, the accuracy entered a nonmonotonic, fluctuating regime. Assuming that the AS was related to arc damage and that the correlation could be captured by an artificial intelligence model, the AS segment with the TS can be used as the input of the model to monitor arc damage. For such a model, high dataset quality is achieved when the TS between AS inputs and labels derived from OS or IS closely approximates the true semantic TS, which improves the ability of the model to capture consistent patterns across training and testing sets, resulting in higher testing accuracy, similar to the trend in Fig. 1c. Therefore, during model training, the maximum testing accuracy corresponded to a specific alignment parameter between the source signal of the label and the AS. The obtained alignment parameter may correspond to, or closely approximate, the actual TS on the time axis, ensuring semantic alignment between the two signals. The implementation and application workflow of the above algorithm architecture are shown in Fig. 1d. Here, T_0^a denotes the semantic TS between the OS sequence and the AS sequence, whereas T_i^a denotes the semantic TS between the IS sequence and the AS sequence. The formal representation of the algorithm architecture is provided in Equation (3), and the corresponding pseudocode for implementing the architecture is shown in Supplementary Algorithm 1.

$$\left.\begin{cases} \theta_t = \min_{\theta_t} \sum_{n_{tr}=1}^{N_{tr}} L(F_{\theta_t}(F_2^{stft}[n_{tr}, t]), y_1[n_{tr}, 0]) \\ t_n = \max_{t} \sum_{n_{te}=1}^{N_{te}} M(F_{\theta_t}(F_2^{stft}[n_{te}, t]), y_1[n_{te}, 0])/N_{te} \\ N = N_{te} + N_{tr} \\ T_n = t_n \cdot T_s \end{cases}\right\} y_1[n, t] \in \{0,1\} \tag{3}$$

where θ_t, F_2^{stft}, $y_1[n, t]$, and L denote the parameters of the neural network F_{θ_t} with a TS parameter t, the time-frequency spectrogram of signal $x_2[n_{tr}, t]$ after short-time Fourier transform, the label corresponding to signal $x_1[n, t]$, and the cross entropy loss function, respectively. $F_2^{stft}[n_{tr}, t]$ and $y_1[n_{tr}, 0]$ are used as input–output pairs for training neural networks, whereas $F_2^{stft}[n_{te}, t]$ and $y_1[n_{te}, 0]$ are used for testing. N_{te} and N_{tr} denote the number of samples in the training and testing sets, respectively. The matching function M returns a value of 1 if two elements are identical, and 0 otherwise. T_n and t_n denote the semantic TS and semantic alignment parameters, respectively, between the sequence of signal $x_1[n, 0]$ and that of signal $x_2[n, 0]$, as determined by the neural network-driven data alignment architecture. The search range for the semantic TS is defined by $t \cdot T_s$. While initially developed for aligning OS or IS with AS, the proposed architecture is broadly applicable to a range of problems involving the alignment of two heterogeneous data streams with unknown initial semantic time shifts and intermittent semantic variations over time. The successful application of this architecture is conditional on the possession of semantic labels for one of the two heterogeneous data streams. The observed semantic TS arises from a combination of factors. The semantic TS typically arises from two main factors: the inherently distinct physical principles of the sensors (e.g., the vastly different propagation speeds of light and sound), and the data processing strategies, where optimal response identification may depend on data from before, during, or after the event. The proposed algorithm architecture aims to solve the ultimate data alignment problem, which is particularly applicable to alignments that cannot be solved through synchronous signal acquisition. Additionally, for alignment exploration under different TSs, the AS must be repeatedly segmented with different initial TSs. Choosing the length of each segment T_s as an integer multiple of the semantic TS search step size is advantageous for reducing the number of segments.

The supplemented contents are in "2.2. Alignment verification and applications" section are shown as:

Additionally, we repeated the above alignment, and the alignment between the OS and AS corresponding to different friction pair materials was analyzed as detailed in Supplementary Discussion 4. The experimental results indicate that the comprehensive results of repeated alignment are more reliable than those of single alignment are, and that determining the semantic TS via the distribution variation of testing accuracy across different TSs is more reliable than relying solely on the MTA. The subsequent alignment-related figures correspond to the comprehensive results of three repeated alignments and will not be repeated in the future. The higher training or testing accuracy under semantic TS than under other TS conditions can be attributed to the weaker correspondence between input signals and semantic labels in non-semantic alignments, which reduces the proportion of functionally relevant components in the loss function that effectively drive model training, thereby slowing the training rate.

The supplemented "Supplementary Algorithm 1. Neural network-driven data alignment algorithm architecture" are shown as:

Supplementary Algorithm 1. **Neural network-driven data alignment algorithm architecture**

Input: the spectrogram list of AS and label list obtained from OS or IS .

initialization, including search ranges of time shift parameter t, model training parameters and number of samples N, etc.
for t in range list:
    randomly extract equal proportional labels of "0" and "1" from the label list to form a list $\{y_1[0,0], y_1[1,0], \cdots, y_1[N-1,0]\}$.
    extract the corresponding spectrogram list of AS $\{F_2^{\mathrm{stft}}[0,t], F_2^{\mathrm{stft}}[1,t], \cdots, F_2^{\mathrm{stft}}[N-1,t]\}$.
    establish a dataset and the dataset is divided into training and testing sets.
    train the model F_{θ_t} based on Equation (3).
    calculate the maximum testing accuracy of the model F_{θ_t} in training.
    obtain the time shift parameter t in the maximum testing accuracy.
calculate the time shift T_{n} between signal $x_1[n,0]$ and $x_2[n,0]$ based on Equation (3).

Comment 5:

The manuscript could be more explicit about how exactly the labels were extracted. For example, was this done by manual frame inspection or via an automated threshold algorithm? It is implied that the "unrecognized green area" in IR images was used as the arc indicator.

Answer of comment 5:

Thanks very much for your valuable comment. Based on your comment, we have supplemented "Supplementary Table 4. Label information of optical and infrared signals" in the "Supplementary Discussion 2. Datasets used for the alignment study" section to indicate the labeling method of the signal.

The supplemented contents are in "Supplementary Discussion 2. Datasets used for the alignment study" section are shown as:

Supplementary Table 4. **Label information of optical and infrared signals**

Experiment number	Signal	Number	Labeling method	Arc response frequency
**2-2**	OS	36000	Manual annotation	256
**2-2**	IS	72000	Automatic annotation (Based on green unrecognized areas)	183
**2-3**	OS	36000	Manual annotation	4821 or 4713 (The same annotator annotates twice, inconsistency rate: 97.84%)
**2-3**	IS	72000	Automatic annotation (Based on green unrecognized areas)	2556
**3-1**	OS	36000	Manual annotation	5768
**3-2**				7652
**3-3**				1944(Inclined friction)

Comment 6:

It would be helpful to include a brief discussion of the computational cost. Training multiple CNNs for different shifts (or training one and sliding the input) can be computationally expensive. Is the method feasible for real-time or large-scale use?

Answer of comment 6:

Thanks very much for your valuable comment. Based on your comment, we have supplemented the time cost estimation of the alignment model in Supplementary Tables 17 and 18. Additionally, we have also supplemented the training and testing time costs of machine learning models in the "Supplementary Discussion 8. Cost evaluation of the arc detection model" section and discussion of the time cost of the architecture in the "Supplementary Discussion 12. Cost evaluation of alignment architecture" section.

The supplemented contents are in “Supplementary Discussion 8. Cost evaluation of the arc detection model” section are shown as:

Supplementary Table 15. **Training time costs of machine learning models**

(Not included in the time for data reading, data storage, and data segmentation)

Model	Samples number	Epoch	Training time per epoch/ s	Testing time per epoch/ s	Total training time/s
GRU	9000	200	4.157	4.034	1638
MLP		100	9.855×10^{-3}	3.621×10^{-2}	13.48
MNet2_1			8.225	3.619	1184
MNet2_2			8.532	3.755	1229
MNet2_4			8.411	3.645	1206
MNet2_8			8.623	3.710	1233
MNet2_16			8.364	3.566	1193
MNet2_32			8.650	3.660	1231
MNet2_64			8.999	4.124	1312
MNet3			9.190	3.954	1314
MNet4			9.109	4.006	1312
RANet		50	64.35	26.59	4547
RDNet13		100	8.878	3.732	1261
RDNet41			9.848	4.391	1424
RMNet			9.881	4.105	1399
RRNet10			11.16	4.683	1584
RRNet18			8.921	3.728	1265
RSNet (3 channels input)	10134	100	72.24	29.89	10213
RSNet (1 channels input)			28.08	11.34	3942
SVM	600	/	/	/	1.557×10^{-2}

The supplemented “Supplemented Tables 17 and 18” are shown as:

Supplementary Table 17. **The time costs of alignment based on RDNet13**

(Inference on the basis of Supplementary Tables 14 and 15)

Samples number	Data preprocessing time/ s	Training time per search steps/s	Search steps	Total time/ s
9000	1095	1261	15	20010
5000	608.5	716.5	15	11356
1000	121.7	139.2	15	2209.7
500	60.85	65.97	15	1050.4
100	12.17	12.50	15	199.67
50	6.085	7.037	15	111.64

Supplementary Table 18. **The time costs of alignment on the basis of frequency domain similarity**

(Inference on the basis of Supplementary Tables 14 and 15)

Samples number	Data preprocessing time/ s	The time for caculate rank correlation coefficient for per search step/s	Search steps	Total time/ s
9000	187.0	230.9	45	10578
5000	103.9	133.3	45	6102.4
1000	20.78	28.84	45	1318.6
500	10.39	15.34	45	700.69
100	2.078	3.162	45	144.37
50	1.039	1.806	45	82.309

The supplemented contents are in “Supplementary Discussion 12. Cost evaluation of alignment architecture” section are shown as:

The computational time required for training a kernel model is provided in Supplementary Table 15. In a fixed semantic time-shift system, the one-time alignment cost of the proposed architecture remains acceptable. In typical machine learning workflows, identifying semantic features that facilitate the extraction of critical information can enhance the performance of traditional, lightweight models. For systems that require frequent alignment, the support vector machine-based kernel model is computationally feasible when it is applied to small sample sizes within limited search ranges. Computational power is widely recognized as the cornerstone of the Fourth Industrial Revolution. In this context, the demand for quantum computing has grown increasingly urgent [15-17], driven by the application of artificial intelligence in the fields of science and industry. Similarly, for systems that require continuous alignment, deploying alignment models built upon a more complex kernel model demands substantial computational resources. The alignment requirements can vary significantly across systems. For systems with low real-time demands, fast kernel model convergence, or offline alignment needs, the applicability and efficiency of the proposed architecture should be evaluated on a case-by-case basis.

Comment 7:

The paper does not explicitly state which sensor's data was ultimately used for the alignment and detection models. One may infer that the airborne sensor was used for arc detection, as it would better capture arc sounds, but this should be clearly stated.

Answer of comment 7:

Thanks very much for your valuable comment. Based on your comment, we have clarified the relevant content in “2.2. Alignment verification and applications” section.

The supplemented contents are in “2.2. Alignment verification and applications” section are shown as:

Line 190,Revised Manuscript: For the aligned AS and OS, the kernel model corresponding to the AS collected by the airborne acoustic sensor, without lg transformation, achieved the maximum MTA among the four input configurations. Therefore, subsequent alignment of the AS and OS, as well as arc detection model using AS would be based on the spectrogram of the AS collected by the airborne sensor without lg transformation.

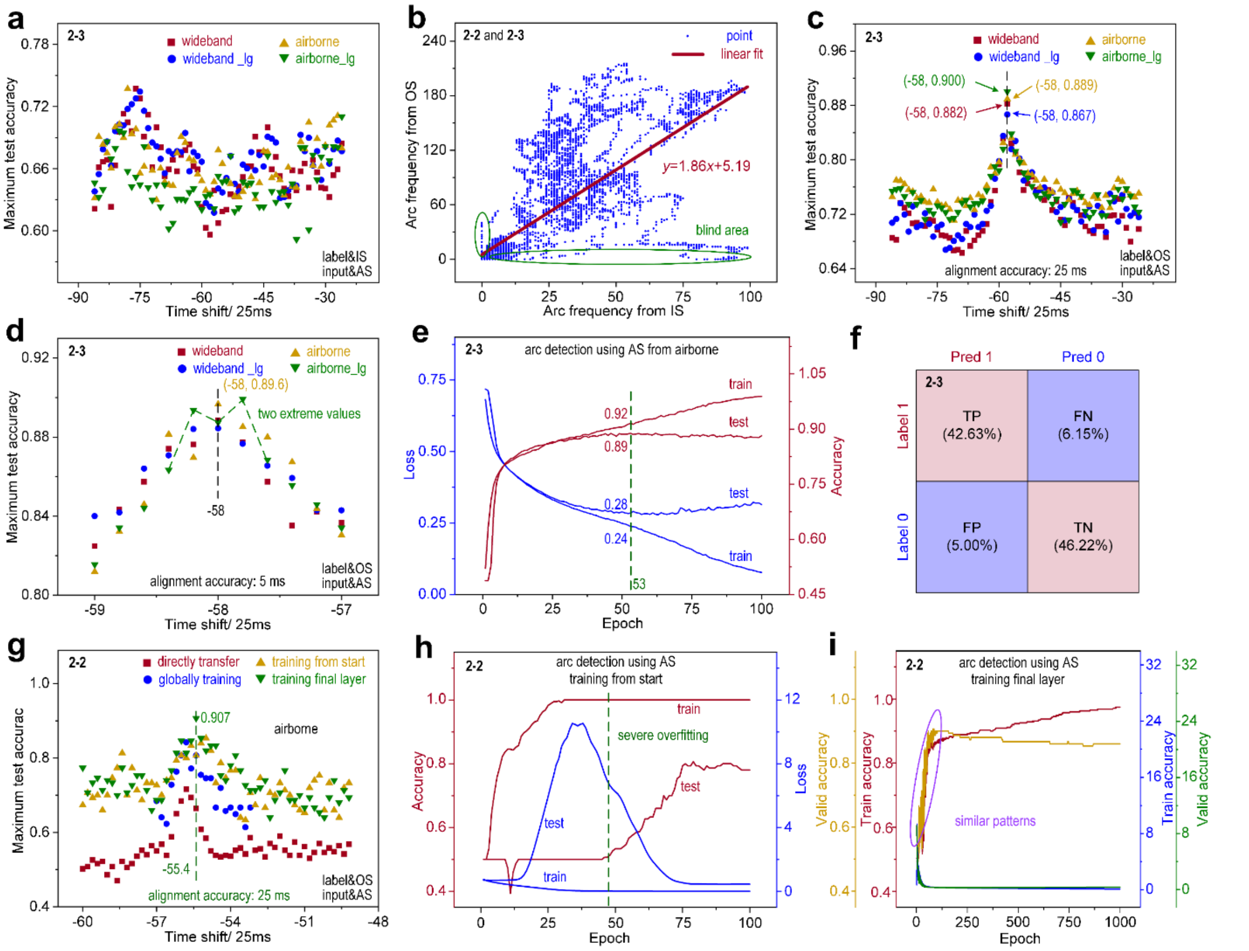

Figure 2. **Verification and application of the data alignment architecture. a** Alignment results (step size: 25 ms) of infrared signals (IS) and acoustic signals (AS). **b** Comparison of the arc frequency every 10 s calculated from optical signals (OS) and IS, **c-d** Alignment results (step size: 25 and 5 ms) of OS and AS in dataset **2-3**. **e** Epoch-accuracy and loss curves of the kernel model with aligned AS as the input and labels obtained from the IS as the output. **f** The confusion matrix of the highest testing accuracy in Fig. 2e, including the true positive (TP) rate, false positive (FP) rate, true negative (TN) rate, and false negative (FN) rate. **g Alignment results (step size: 5 ms) of OS and AS data in dataset 2-2**. **h-i** Epoch-accuracy and loss curves of the model in Fig. 2g.

Comment 8:

Table 1 presents a well-designed set of 14 orthogonal experiments, yet only results from experiments 2-2 and 2-3 are presented and discussed. The rationale for selecting these specific experiments should be explicitly stated. Are they representative cases or particularly informative for alignment validation? If space constraints limit full reporting, a brief summary of results across all 14 conditions (e.g., in supplementary files) would

enhance the transparency and reproducibility of the work.

Answer of comment 8:

Thanks very much for your valuable comment. Based on your comment, we have supplemented relevant explanations in the "Supplementary Discussion 2. Datasets used for the alignment study" section.

The supplemented contents are in "Supplementary Discussion 2. Datasets used for the alignment study" section are shown as:

Supplementary Table 3. **The dataset numbering and corresponding conditions of the orthogonal experiments**

Number	Monitor signal	Material	Load mode	Electric current / A	Rotational speed/ r/min	Time/ s
**1-1-1**	OS	CrZrCu alloy	CDL	2	50	1000
**1-1-2**	OS	CrZrCu alloy		2	150	
**1-1-3**	OS	CrZrCu alloy		8	50	
**1-1-4**	OS	CrZrCu alloy		8	150	
**1-2-1**	OS	CrZrCu alloy	CLL	2	50	
**1-2-2**	OS	CrZrCu alloy		2	150	
**1-2-3**	OS	CrZrCu alloy		8	50	
**1-2-4**	OS	CrZrCu alloy		8	150	
**1-3-1**	OS	CrZrCu alloy	CMLL	2	50	
**1-3-2**	OS	CrZrCu alloy		2	150	
**1-3-3**	OS	CrZrCu alloy		8	50	
**1-3-4**	OS	CrZrCu alloy		8	150	
**2-2**	OS,IS and AS	CrZrCu alloy	CLL	2、5、8	50、100、150	900
**2-3**	OS,IS and AS	CrZrCu alloy	CMLL			
**3-1**	OS and AS	CuBe alloy				
**3-2**	OS and AS	CuSn alloy				
**3-3**	OS and AS	CuZn alloy				

Line 185, Supplementary Information: Signal segments obtained via threshold-based acquisition were deemed unsuitable, as this mode failed to capture the entire test duration and risked the loss of critical information. Although not central to the study, it is necessary to explain that the ASs for experiments **1-1-1** and **1-3-4** were acquired via threshold-based triggering rather than continuous waveform recording. As a result, the captured segments covered only approximately 10% of the total test duration, omitting substantial information and rendering the data incompatible with the alignment framework proposed in this study. Accordingly, these ASs are not included in Supplementary Table 3. To bypass the hardware-level synchronization of the in-situ monitoring module and accelerate validation of the alignment architecture, software-based synchronization triggering was employed to approximately synchronize the acquisition of the OS, IS, and AS.

Comment 9:

The terms "positive sample" and "negative sample" are used when describing the dataset and model training, but the manuscript does not explicitly define them. I infer that "positive" refers to an acoustic signal segment during which an arc was occurring, and "negative" refers to the absence of arc activity. This should be explicitly defined in the manuscript.

Answer of comment 9:

Thanks very much for your valuable comment. Based on your comment, we have supplemented relevant descriptions in the main text and Fig. 1b in the "2.1. Alignment requirements and architecture" section.

Meanwhile, we have also supplemented corresponding descriptions in the “Supplementary Discussion 2. Datasets used for the alignment study” section.

The supplemented and revised contents are in “2.1. Alignment requirements and architecture” section are shown as:

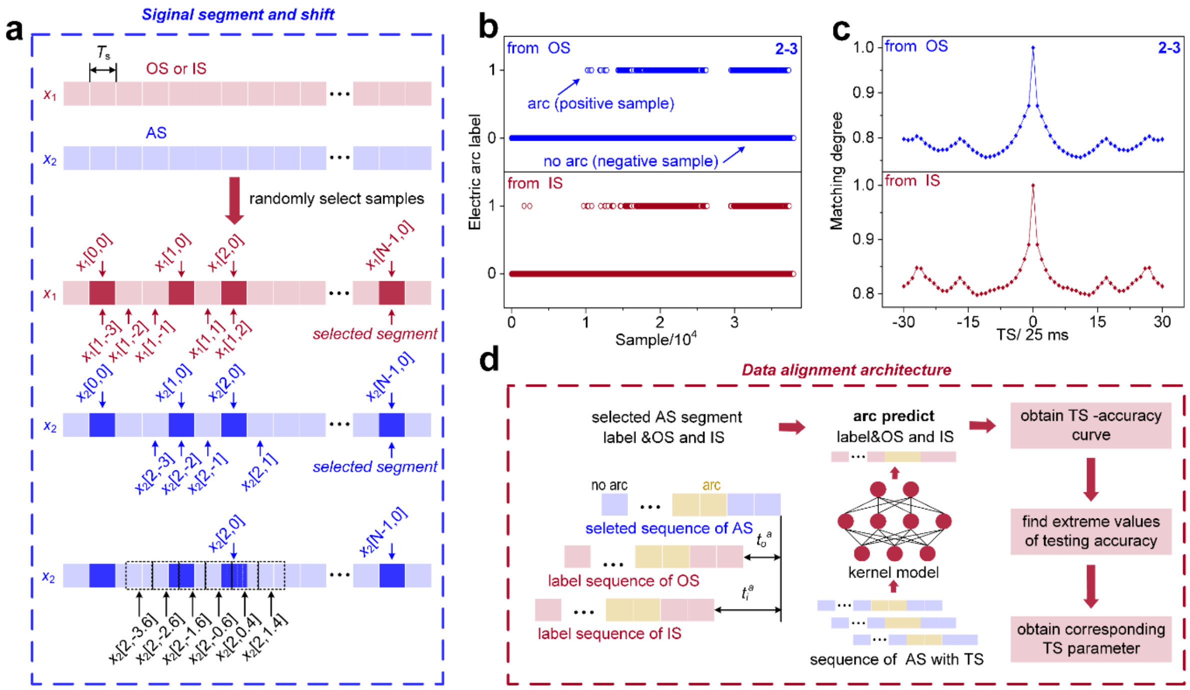

Figure 1. **The alignment architecture for heterogeneous signals. a** Process of random sampling and symbol definition of signal segments. **b** Label obtained from infrared signals (IS) and optical signals (OS) to determine arc damage, **c** Time shift (TS)-accuracy curve of the labels. **d** The alignment architecture and its application flow.

Line 113, Revised Manuscript: The labels (“1” denotes a positive sample corresponding to the arc, and “0” denotes negative samples corresponding to the absence of arc)

The supplemented and revised contents are in “Supplementary Discussion 2. Datasets used for the alignment study” section are shown as:

Line 238, Supplementary Information: The label “1” denotes the presence of an arc (positive sample), whereas the label “0” denotes its absence (negative sample).

Comment 10:

Several undefined abbreviations are used in figures without definition (e.g., OS, AS, IS, AES, AE, SFT, CAM, etc.). While domain experts might infer some of these, it burdens the reader and can lead to misinterpretation. This is especially problematic in figures meant to stand on their own. All abbreviations should be defined either in the legend, the caption, or in the main text.

Answer of comment 10:

Thanks very much for your valuable comment. Based on your comment, we have supplemented “Supplementary Table 1. Abbreviations and full names of the technical terms” for readers to inquire about their meanings. Meanwhile, we have indicated the full name of the abbreviation where abbreviation appears for the first time in text and figures in each section.

The supplemented “Supplementary Table 1. Abbreviations and full names of the technical terms” are shown as:

Supplementary Table 1. **Abbreviations and full names of the technical terms**

Abbreviations	Meanings	Abbreviations	Meanings
AE	Acoustic emission	MSE	Mean squared error
AS	Acoustic signal	MTA	Maximum testing accuracy
C	Capacitor	NS	Number of samples
CAM	Category activation mapping	OS	Optical signal
CDL	Constant displacement loading	PKL	Pickle
CLL	Constant load loading	PNG	Portable network graphics
CMLL	Constant minor load loading	R	Resistor
CSV	Comma-separated values	RAM	Random access memory
FN	False negative	RANet	Revised AlexNet
FP	False positive	RDNet13	Revised DenseNet13
GRU	Gate recurrent unit	RDNet41	Revised DenseNet41
IS	Infrared signal	RMB	Renminbi
KM	Kernel model	RMNet	Revised MobileNet V3 small
KRCC	Kendall rank correlation coefficient	RRNet10	Revised ResNet10
MLP	Multi-layer perceptron	RRNet18	Revised ResNet18
LR	Learning rate	RSNet1	Revised Squeezenet 1.0
MB	Megabyte	SRCC	Spearman rank correlation coefficient
MNet2_1	MiniNet2_1	STD	Standard deviation
MNet2_2	MiniNet2_2	SVM	Support vector machines
MNet2_4	MiniNet2_4	TIFF	Tag image file format
MNet2_8	MiniNet2_8	TN	True negative
MNet2_16	MiniNet2_16	TP	True positive
MNet2_32	MiniNet2_32	TS	Time shift
MNet2_64	MiniNet2_64	V	Velocity
MNet3	MiniNet3	U	Voltage
MNet4	MiniNet4		

Comment 11:

The manuscript uses some vague terms that require more precise definition. For instance, the terms "relatively stationary" and "relatively non-stationary" appear as axis labels in Fig. 4, but are not introduced or explained anywhere in the manuscript.

Answer of comment 11:

Thanks very much for your valuable comment. Based on your comment, we have revised the unclear terms in Supplementary Fig. 17c (Fig.4c from the previous version) in "Supplementary Discussion 5. Interpretability analysis of the acoustic signals" Section.

The revised contents are in the "Supplementary Discussion 5. Interpretability analysis of the acoustic signals" section are shown:

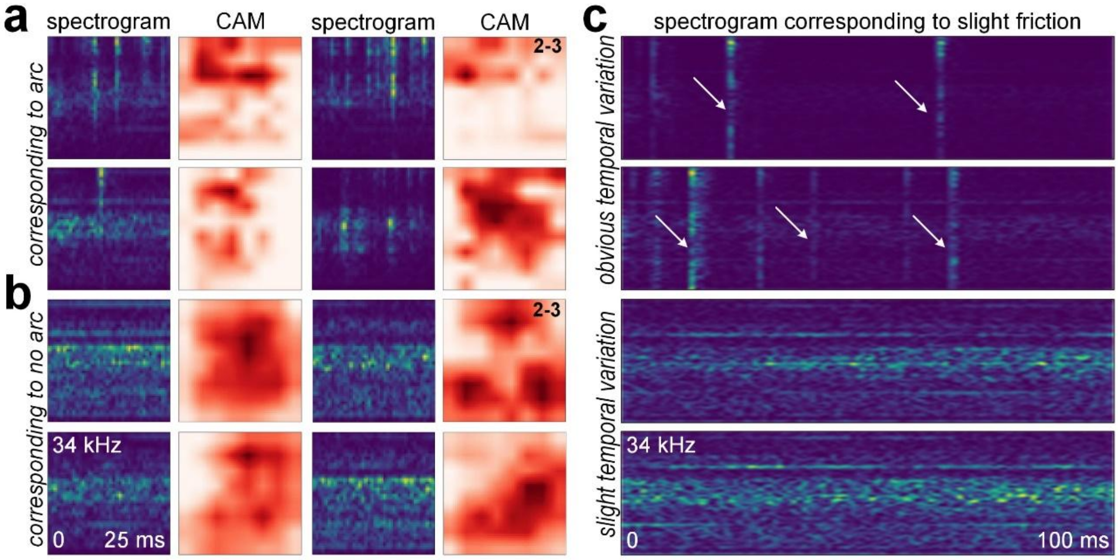

Supplementary Figure 17. **Interpretability analysis of typical acoustic signals. a-b** Normalized spectrograms of the acoustic signals selected via the signal alignment method and the calculated class activation maps (CAMs) under conditions of arc damage and no arc damage. **c** Typical normalized spectrogram of the acoustic signals under the condition of slight contact (current: 8 A) between two friction specimens (Supplementary Fig. 16.). (a, b and c show multiple sets of spectrograms with the same semantics to more fully demonstrate the common features of the signal.)

Comment 12:

While the manuscript touches briefly on limitations (e.g., IR camera issues), the discussion could be expanded. It would be helpful to mention the scope and limitations of the proposed method more explicitly in the discussion or conclusion section.

Answer of comment 12:

Thanks very much for your valuable comment. Based on your comment, we supplemented the alignment experiments with different numbers of sample as well as reasons for failed alignment of infrared and acoustic signals in the "2.3. Factors affecting alignment accuracy" section.

The supplemented contents are in "2.3. Factors affecting alignment accuracy" section are shown as:

Figs. 3d–3f illustrate the impact of the NS on alignment accuracy. When the same kernel model is employed for alignment across varying NSs, the models trained with fewer samples exhibit more pronounced underfitting. This underfitting is reflected in the reduced testing accuracy, which in turn undermines the credibility of the alignment. Therefore, the KM was trained using learning rates that increased from 2×10^{-4} at an NS of 9000 to 2×10^{-3} at an NS of 50. The learning rate is negatively correlated with the NS (Fig. 3d). The alignment accuracy deteriorates from 5 ms at an NS of 9000 to 20 ms at an NS of 50. Concurrently, the average STD of the MTA across TSs increases as the NS decreases. With decreasing NS, the kernel model in the semantic TS exhibits increased overfitting and diminished generalization capability, resulting in decreased credibility of the alignment result, as shown in Fig. 3e. As shown in Fig. 3f, as the NS decreases, the trend of peak-shaped variation in the MTA near the semantic TS becomes less pronounced. In other words, the correlation—whether positive or negative—between the MTA on either side of the semantic TS and the TS weakens as the NS decreases. The typical epoch-loss and accuracy curves of the kernel model corresponding to Fig. 3f are shown in Supplementary Fig. 23. The KM exhibits an unstable training process when the NS falls below 500. Under

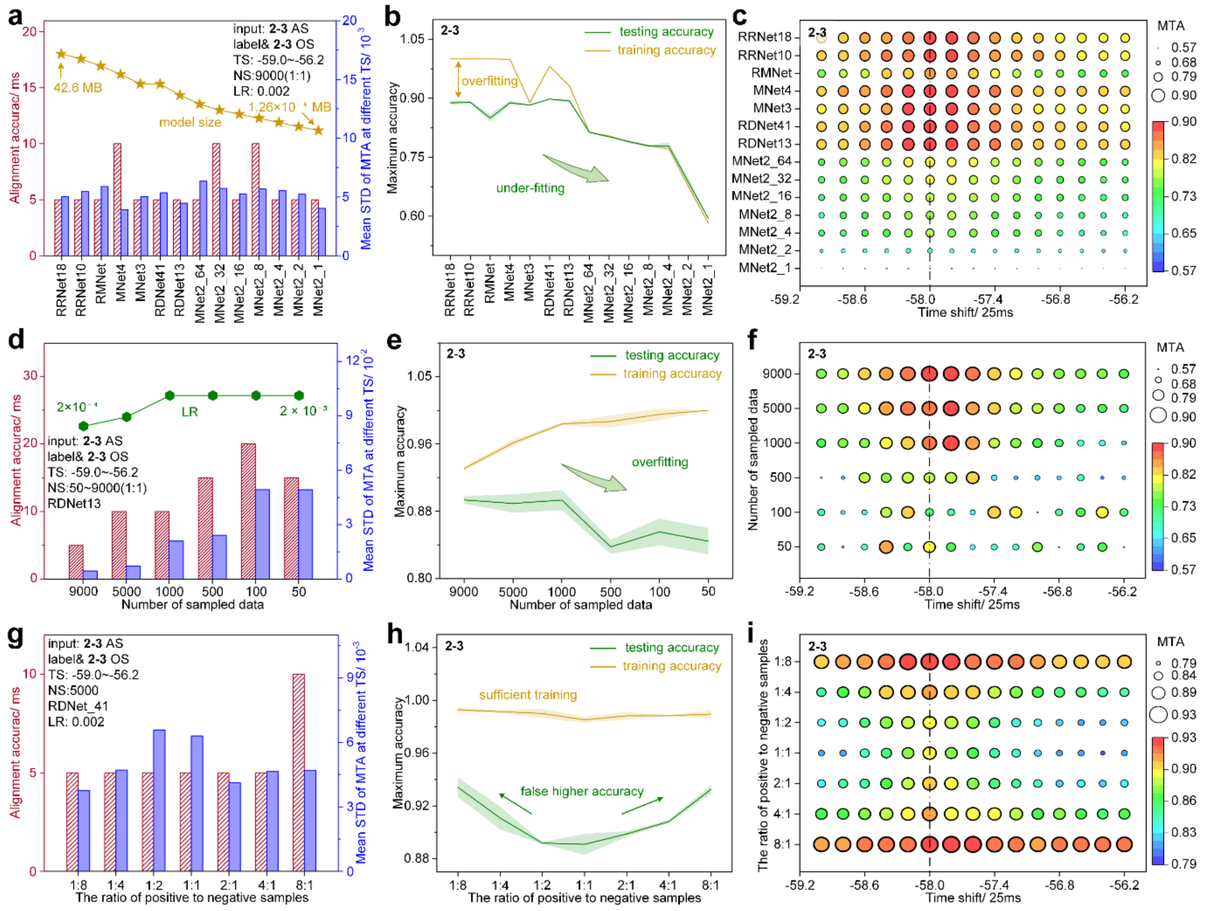

Figure 3. **Analysis of factors affecting alignment accuracy.** **a** Variance in alignment accuracy and standard deviation (STD) of the maximum testing accuracy (MTA) across different kernel models under the same learning rate (LR). **b** Maximum training and testing accuracy across different kernel models. **c** Bubble chart of the MTA across different kernel models and time shifts (TSs) (step size: 5 ms). **d** Alignment accuracy of the RDNet13 kernel models as the number of samples (NS) varies. **e** Corresponding NS–maximum accuracy curve. **f** Corresponding bubble chart of the MTA across different TSs (step size: 5 ms). and NS. **g** Variation in alignment accuracy with the proportion of positive to negative samples. **h** Maximum accuracy across different training sample proportions. **i** Corresponding bubble chart of MTA across different TSs (step size: 5 ms) and training sample proportions.

stable training conditions, a higher STD in the MTA across TSs reflects a greater sensitivity of accuracy to temporal variation, which is advantageous for alignment. However, in the case of unstable training, an increased STD (Fig. 2d) does not contribute to improved alignment, because the variability primarily arises from training instability rather than from meaningful temporal sensitivity. Additionally, when aligning the OS with the IS via the RDNet13 kernel and a limited NS of 50, an alignment accuracy of 15 ms was achieved, as shown in Fig. 3d. As illustrated in Fig. 1f, the arc events extracted from both the IS and OS demonstrate a temporally consistent distribution. The failed alignment between the IS and AS, as shown in Fig. 2a, is likely due to inaccuracies in the local time axis of the IS captured by the thermal imager, rather than the limited number of IS images (1800) associated with the arc. The effect of NS on alignment accuracy based on the extremely lightweight kernel model Mnet2_1 is similar to that of RDNet13, as presented in Supplementary Discussion 9. Moreover, the maximum degradation in alignment resolution within the range of 50-9000 samples is limited to just 15 ms. Therefore, in the initial stages of the large-scale search for alignment parameters, adopting a coarse alignment strategy based on a small NS and lightweight KMs is a cost-effective and practical solution.

Comment 13:

From page 5, line 7 onward, several figure references appear incorrect. For example, Fig. 2f and 2g should likely be Fig. 1f and 1g based on the surrounding discussion. Similarly, Fig. 3h (page 9, line 13) appears to be mislabeled and should be Fig. 3i. These should be corrected to ensure accurate cross-referencing.

Answer of comment 13:

Thanks very much for your valuable comment. Based on your comment, we have corrected the references of Fig. 1d (previous Fig. 2i), Supplemented Fig. 3 (previous Fig. 1j), Supplemented Fig. 10 (previous Figs. 1f-i), and Supplemented Fig. 18 (previous Fig. 1k). Meanwhile, we also checked the figure citations throughout the entire text.

The revised contents are in "2.1. Alignment requirements and architecture" section are shown as:

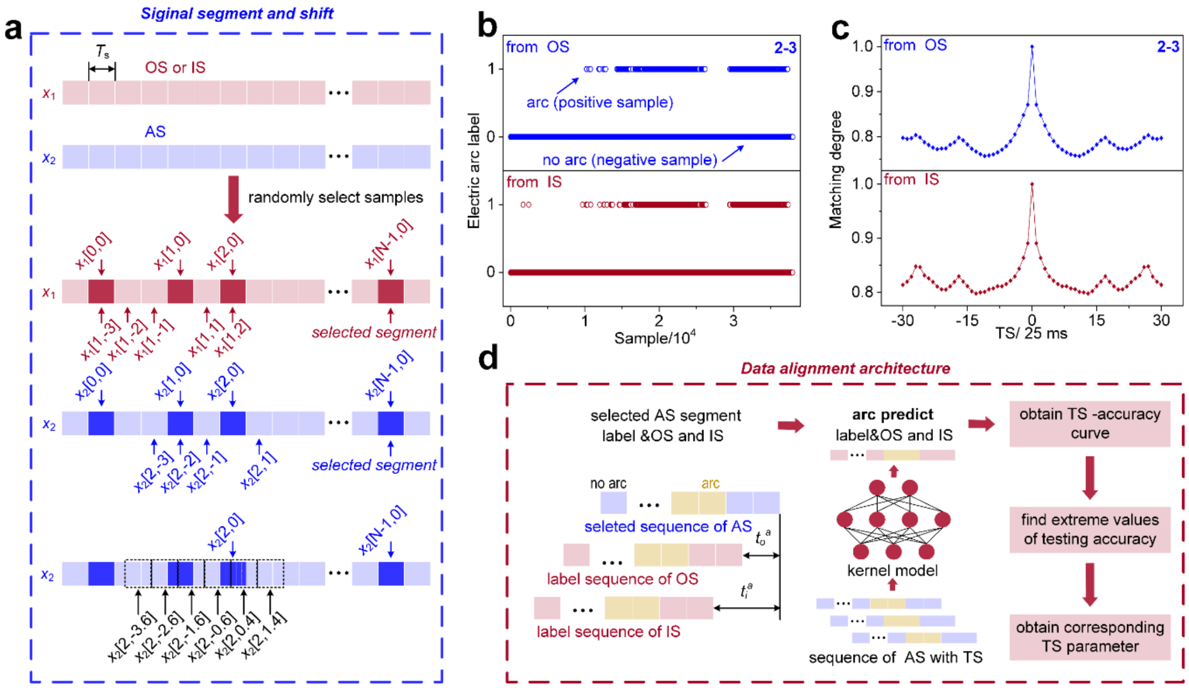

Figure 1. The alignment architecture for heterogeneous signals. a Process of random sampling and symbol definition of signal segments. **b** Label obtained from infrared signals (IS) and optical signals (OS) to determine arc damage, **c** Time shift (TS)-accuracy curve of the labels. **d** The alignment architecture and its application flow.

Line 125, Revised Manuscript: The implementation and application workflow of the above algorithm architecture are shown in Fig. 1d.

The revised contents are in "Supplementary Discussion 1. Instrument and alignment requirements" section are shown as:

Line 60, Supplementary Information: Supplementary Fig. 3 shows the energy variation under friction and no-friction conditions controlled by a micrometer head.

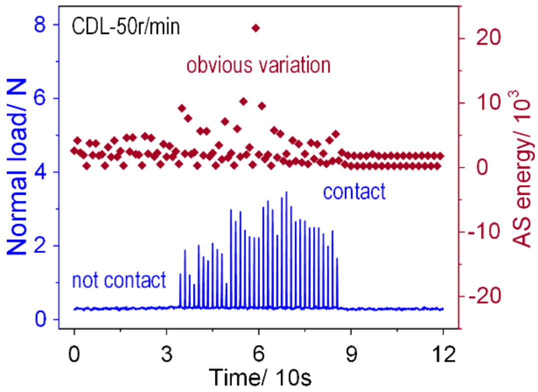

Supplementary Figure 3. **The variations in the acoustic signal (AS) energy feature (obtained from an airborne acoustic emission sensor) and normal load with contact conditions controlled by micrometer heads.**

The revised contents are in "Supplementary Discussion 2. Datasets used for the alignment study" section are shown as:

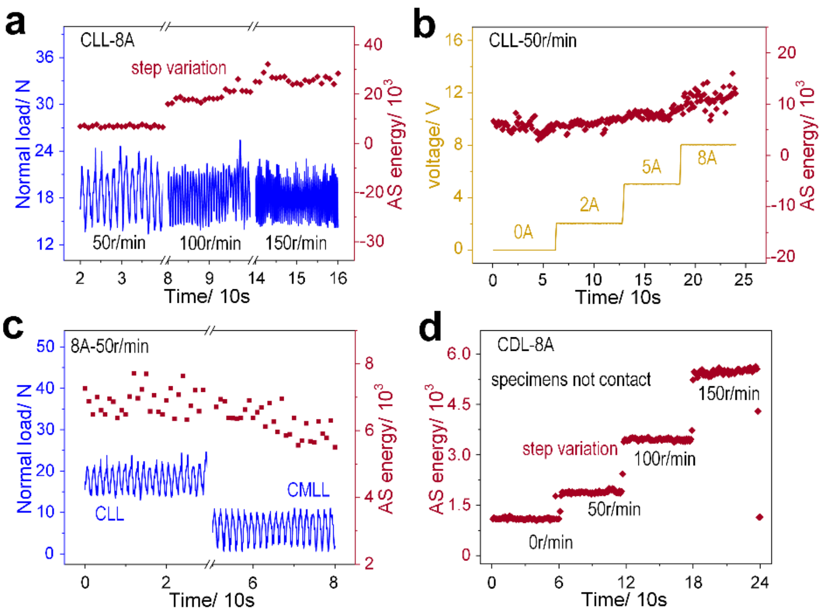

Supplementary Figure 10. **Analysis of the energy of the acoustic signals (AS). a-b** Energy variation of the AS at different speeds and currents under constant load loading (CLL) mode. **c** Energy of the AS under CLL and constant minor load loading (CMLL) modes. **d** The energy of the AS in the background of the system corresponding to different rotational speeds under constant displacement loading (CDL) mode.

Line 248, Supplementary Information: The energy variations under different working conditions are compared, as shown in Supplementary Figs. 10a-c. As shown in Supplementary Fig. 10a, the energy increases with increasing rotational speed, exhibiting a stepwise trend across different speed levels. Supplementary Fig. 10b illustrates the trend in energy variation under different current conditions. As the current increases, the energy also increases without any step variation, and the energy distribution becomes dispersed. Supplementary Fig. 10c shows the signal energy under various normal loads, with more significant normal loads corresponding to higher energy. The experimental conditions described above suggest the presence of significant domain shifts in AS under varying friction conditions. Supplementary Fig. 10d presents the energy variation of background noise unrelated to the current-carried friction of the pin and disk specimens under different rotational speed

conditions.

The revised contents are in "Supplementary Discussion 5. Interpretability analysis of the acoustic signals" section are shown as:

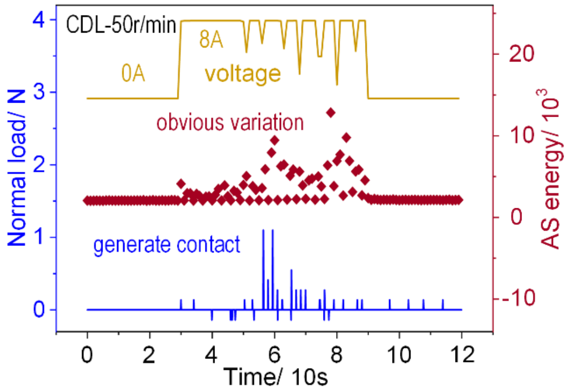

Supplementary Figure 18. **The variations in the acoustic signal (AS) energy and normal load with varying electrification conditions, occurring in the case of slight separation of the friction pair under the constant displacement loading (CDL) mode.**

Line 420, Supplementary Information: Supplementary Fig. 17c shows the normalized spectrogram corresponding to the AS in Supplementary Fig. 18, which corresponds to arc damage and slight friction(current: 8A). Supplementary Fig. 18 illustrates the energy variation when the power is switched on and off under the constant displacement loading mode. The micrometer head was rotated to slightly separate the current-carried friction pair. Initially, the power was switched off (0 A), then switched on (8 A), and finally, the power was turned off again (0 A). The variations in the voltage signal in the figure reflect this process. When voltage was applied to the friction pair, an increase in voltage fluctuation indicated arc damage. In contrast, an increase in the normal load was associated with the formation of minor surface protrusions, likely originating from transferred material, which contributed to elevated friction. Supplementary Fig. 17c presents representative spectrograms recorded under a current load of 8 A, including both time-stable patterns and those exhibiting pronounced temporal fluctuations, The relatively fluctuating spectrogram in Supplementary Fig. 17c exhibits a lower fluctuation frequency along the time axis than that in Supplementary Fig. 17a does, which may correspond to a higher frequency of collisions and frictional interactions between small surface protrusions under the constant minor load loading mode than under the slight friction condition (current: 8A) corresponding to Supplementary Fig. 18.

Comment 14:

The vertical axes in both Figures 4a and 4b are labelled as "corresponding to no arc," yet the text suggests that these plots include arc-related events. This inconsistency should be resolved by aligning the figure axes labels with the textual descriptions or clarifying exceptions.

Answer of comment 14:

Thanks very much for your valuable comment. Based on your comment, we have corrected Supplementary Figs. 17a-b (formerly Figs. 4a-b) in the "Supplementary Discussion 5. Interpretability analysis of the acoustic signals" section.

The revised contents are in "Supplementary Discussion 5. Interpretability analysis of the acoustic signals" section are shown as:

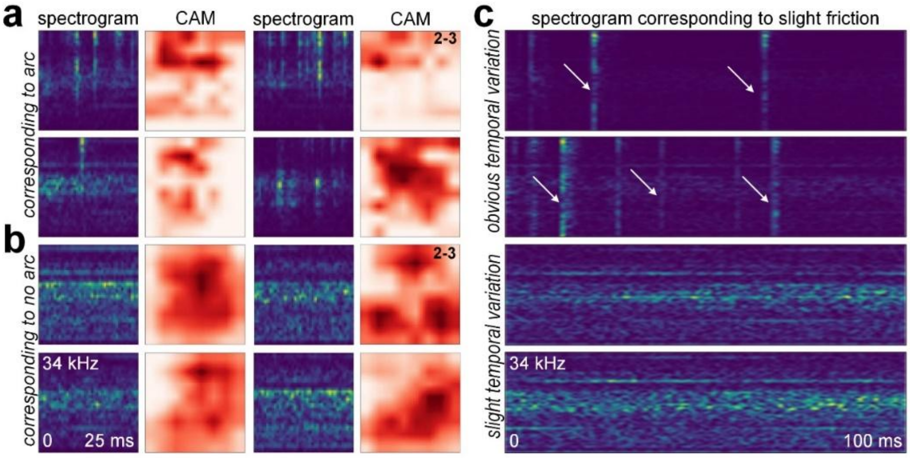

Supplementary Figure 17. **Interpretability analysis of typical acoustic signals. a-b** Normalized spectrograms of the acoustic signals selected via the signal alignment method and the calculated class activation maps (CAMs) under conditions of arc damage and no arc damage. **c** Typical normalized spectrogram of the acoustic signals under the condition of slight contact (current: 8 A) between two friction specimens (Supplementary Fig. 16.). (a, b and c show multiple sets of spectrograms with the same semantics to more fully demonstrate the common features of the signal.)

Comment 15:

Figures 4a, 4b, and 4c contain two rows of images, but the distinction between the rows is not clearly explained in the captions or main text. This makes interpretation difficult. The authors should clarify the meaning and purpose of the two rows to improve reader understanding.

Answer of comment 15:

Thanks very much for your valuable comment. Based on your comment, we have supplemented corresponding explanations in Supplementary Figures 17 (formerly Figs. 4a-c) in the "Supplementary Discussion 5. Interpretability analysis of the acoustic signals" section.

The revised contents are in "Supplementary Discussion 5. Interpretability analysis of the acoustic signals" section are shown as:

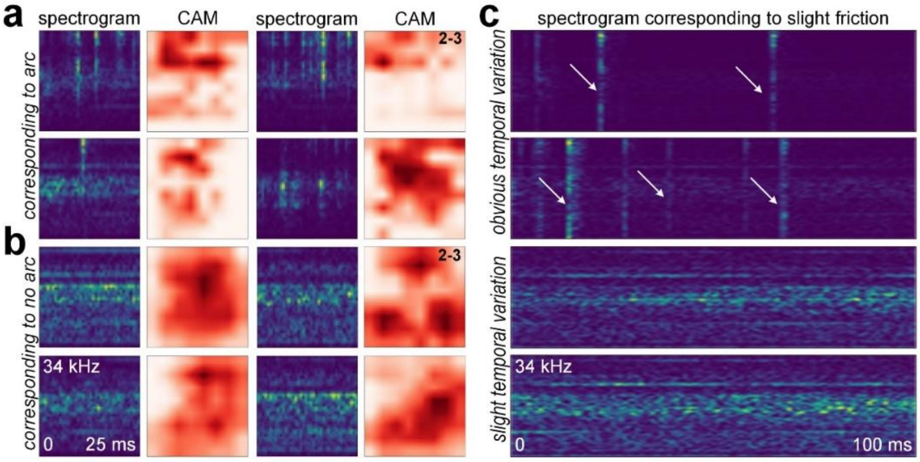

Supplementary Figure 17. **Interpretability analysis of typical acoustic signals. a-b** Normalized spectrograms of the acoustic signals selected via the signal alignment method and the calculated class activation maps (CAMs) under conditions of arc damage and no arc damage. **c** Typical normalized spectrogram of the acoustic signals under the condition of slight contact (current: 8 A) between two friction specimens (Supplementary Fig. 16.). (a, b and c show multiple sets of spectrograms with the same semantics to more fully demonstrate the common features of the signal.)

Comment 16:

The figure references on Page 14 are inconsistent and confusing. Subfigures labelled as Fig. 6e, 6f, 6h, and 6i are mentioned, but Fig. 6 itself does not appear to exist. Similarly, Fig. 5 is never referenced, making it unclear whether it has been omitted or misnumbered. The entire figure sequence should be checked for consistency.

Answer of comment 16:

Thanks very much for your valuable comment. Based on your comment, we have corrected references of Supplementary Figure 21 (previous Figs. 1f-i). Meanwhile, we also checked the image citations throughout the entire text.

The revised contents are in "Supplementary Discussion 7. Feasibility of arc detection via optical signals" section are shown as:

Line 519, Supplementary Information: The arc detection model trained on dynamically thresholded images was subsequently applied to determine whether the OS in datasets from **1-1-1** to **1-3-4** corresponded to arc damage. The test results are shown in Supplementary Fig. 21. Supplementary Figs. 21a-c display the number of arc damage events detected in each OS set from datasets from **1-1-1** to **1-3-4** . The frequency and distribution of arc damage events on the time axis are shown in Supplementary Figs. 21d-f. For the datasets from **1-1-1** to **1-1-4** in Supplementary Figs. 21a and 21d, the number of arc damage events was not considered meaningful because of the variations in the axial runout errors of the two clamped specimens and the difficulty in maintaining a consistent load distribution for each dataset. In contrast, the spatial distribution of arc damage exhibited an alternating pattern, which was consistent with the expected behavior. In the dataset of datasets **1-1-3**, the relatively low frequency of arc events may be attributed to the minimal axial runout between the clamped specimens. Supplementary Figs. 21b and 21e show the arc numbers and temporal distributions of arc damage events under the constant load loading mode, respectively. Supplementary Figs. 21c and 21f present the corresponding results for the constant minor load loading mode. .

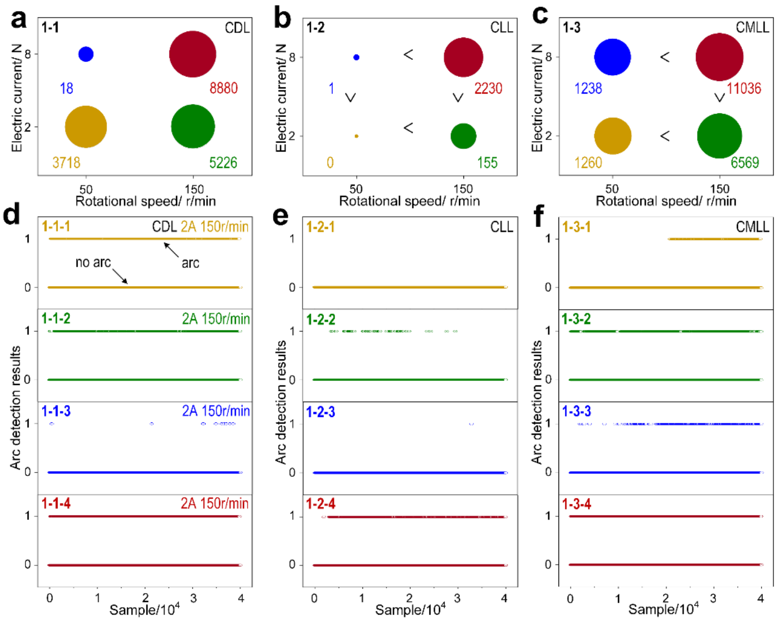

Supplementary Figure 21. **Application of the arc detection model with optical images processed by a dynamic threshold adaptive as input**. **a-c** Arc damage frequency calculated via the arc damage detection model using optical signals for datasets from **1-1-1** to **1-1-4** under constant displacement loading (CDL) mode, datasets from **1-2-1** to **1-2-4** under constant load loading (CLL) mode, and datasets from **1-3-1** to **1-3-4** under constant minor load loading (CMLL) mode. **d-f** Distributions of the corresponding arc damage on the time axis.

Overall Assessment:

The manuscript presents strong scientific content and proposes a novel method for multimodal signal alignment using deep learning. However, its clarity is hindered by structural and presentation issues. Revising key elements such as inconsistent terminology, unclear figure labelling, and missing methodological context will significantly enhance the manuscript's readability. At present, the need to interpret undefined terms or locate missing figure components detracts from the core contributions. Addressing these concerns will not only improve the manuscript's quality but also allow readers and reviewers to better engage with and appreciate the work.

Answer of overall assessment:

Thank you for your recognition and detailed guidance. We have supplemented, revised, and reorganize the relevant content of the entire paper according to your comments one by one to improve the clarity and readability of the paper. These supplement and modifications include inconsistencies in terminology, unclear figure labelling, methodological context, the full name of the abbreviation of the term, figure citation, etc. The specific modifications are shown in the revised manuscript, supplementary information, and the above "Answer of comment".

Reviewer #6

Signal/image alignment is an very important and challenging issue in the community of AI applications.

This manuscript attmepts to tackle the issue of aligning 1D acoustic signal and 2D images. However, a few concerns need to be addressed before its publication.

Comment 1:

The big issue is lack of novelty. Section '4. Signal alignment method' should be the key part for this research work in terms of novelty. However, the main idea of the proposed method is an dedicated experiment design for data acquisition. Then a deep learning network is used to further improve the alignment accuracy of signals. The novelty is minor.

Answer of comment 1:

Thanks very much for your valuable comment. We fully agree with your viewpoint. The research focus of deep learning has shifted from previous backbone model research to the challenging field of multimodal research. Based on your comment, we have thoroughly integrated the paper to highlight alignment in the main text, and content related to the device has been moved to the supplementary materials. The specific modifications are shown in the main text and supplementary information.

Regarding the universality of the align architecture, we have added alignment experiments based on a dataset with a determined offset in the "2.4. Promotion of alignment architecture" sections and the "Supplementary Discussion 11. Supplement of alignment architecture promotion" section to demonstrate the universality of the proposed alignment method. The experimental results suggest that machine learning may serve as an effective discriminator for the matching degree between input and output.

Regarding the innovation of alignment architecture, we have supplemented the limitation of cross-correlation and supervised learning algorithms based on cross-correlation algorithms in "1. Introduction" section.

The revised contents are in "1. Introduction" section are shown as:

The term "alignment" is commonly used in three contexts: alignment between the source and target domains in transfer learning [27–29], alignment in multimodal transformation and fusion [30–32], and alignment of signals along the timeline [33–36]. With respect to transfer learning, the essence of domain generalization lies in aligning the features of the source and target domains to leverage information from the source domain, thereby enhancing model performance on tasks in the target domain [29,37-42]. With respect to multimodal domains, alignment is fundamental to the effective fusion of multimodal information [31]. In multimodal learning, alignment involves projecting data from different modalities into a shared semantic space, where semantically similar concepts are placed closer together, thus facilitating the integration of diverse information sources [32]. Similarly, the goal of temporal signal alignment is to ensure semantic consistency between corresponding segments across signals. While hardware-based synchronization is the preferred approach, the differing acquisition principles of heterogeneous sensors and the varying discrimination based on heterogeneous data often limit its applicability. In such cases, cross-correlation analysis [43-45], isconsidered. However, cross-correlation analysis often requires prior domain knowledge, such as understanding the positive or negative correlation between signal features. To enhance applicability, supervised learning has been employed to derive transformation functions that generate effective features for alignment [46]. A significant limitation of this approach is the reliance on manually annotated semantic time shifts between signals as the gold standard for training[46]. Aligning heterogeneous data without domain knowledge is challenging [47], especially when semantic time shifts are unknown and semantic variations are transient [48, 49]. First, the unknown semantic time shifts hinder the extraction of manually annotated semantic time shifts and effective alignment features [46]. Furthermore, transient semantic variations further hinder the establishment of

associations between heterogeneous data. Consequently, a robust and unsupervised alignment method is urgently needed to overcome the associated challenges. Given the successful use of neural networks for alignment in transfer learning and multimodal domains [27–32, 37–42], a critical research question arises: can a novel neural network-driven architecture be designed to address the challenges of heterogeneous data alignment with unknown semantic time shifts and intermittent semantic variation?

The supplemented contents are in "2.4. Promotion of alignment architecture" section are shown as:

To further evaluate the applicability and reliability of the proposed alignment architecture, extensive experiments were performed on datasets with predetermined alignment parameters. For time series data, to accelerate the experimental process, both the AS spectrograms and the corresponding labels were synthetically generated on the basis of the arc response patterns of the arc igniter. The construction procedure is illustrated in Supplementary Fig. 27, and the detailed algorithm for generating data is provided in Supplementary Algorithm 3. On the basis of the gated recurrent unit (GRU) KM, alignment experiments were conducted on the generated spectrograms and corresponding labels (Fig. 4b), with the sample shift search range set from -8 to +8. In this setup, the frequency response intensity vectors at each time point in the spectrogram were used as feature vectors input into the time series model. The alignment results are shown in Fig. 4c, and the distribution of testing accuracy across different sample shifts is presented in Supplementary Fig. 28. Both the training accuracies and testing accuracies reached their maximum values at a sample shift of 0, indicating that the alignment architecture successfully achieved correct alignment between the time series data and the corresponding labels. To further validate the applicability of the proposed alignment architecture to tabular data, a synthetic dataset was constructed, referring to recent research [52], via the function $z = \sin x + y^2$, where x and y were sampled within the range of -0.5 --+0.5. Data points with $z > 0.0696$ were assigned a label of 1, whereas those with $z \leq 0.0696$ were assigned a label of 0 (Fig. 4d). The feature vectors composed of x and y, and the corresponding labels, were aligned via a support vector machine (SVM) KM (Fig. 4d). The alignment results shown in Fig. 4e demonstrate the favorable alignment performance of the architecture. In the multimodal era driven by transformer -based architectures, semantic vectors have become the primary medium for cross-modal information exchange. To evaluate the applicability of the proposed alignment framework in such contexts, alignment was performed between dataset **2-3** OS images and their corresponding 8-dimensional semantic feature vectors (mean, maximum, minimum, range, variance, skewness, kurtosis, and entropy) via the RANet KM (Fig. 4g). The alignment results are presented in Figs. 4h and 4i. While the training mean squared error (MSE) did not reach its minimum at a sample shift of 0, the testing MSE achieved its lowest value at this point. Moreover, the overall distribution of the testing MSE at a sample shift of 0 was lower than that at other shifts, indicating successful alignment between the OS images and their associated semantic representations. The sample shift–testing MSE curves corresponding to the three alignment trials (Figs. 4g–i) are presented in Supplementary Fig. 29. Among the three, one instance exhibited suboptimal alignment performance, with an alignment resolution exceeding 8 sample shifts. This discrepancy may be attributed to internal balancing mechanisms among the outputs of the multioutput neural network. To increase the robustness of alignment in such challenging scenarios, multiple alignment runs are recommended to increase the reliability of the estimated semantic time shift. Additionally, supplementary alignment experiments are presented in Supplementary Discussion 11, including the alignment between images and labels, tabular data and labels, and input vectors and output values of artificially constructed functions. All the experiments achieved favorable alignment performance. In summary, at the task level, the proposed alignment architecture is applicable not only to regression tasks of KMs but also to classification tasks. At the model level, the architecture accommodates a wide range of model architectures, including traditional machine

learning models, time series models, and convolutional neural networks. At the data level, the architecture supports alignment across diverse modalities, including tabular data, time series data, and image data, with corresponding semantic labels or feature vectors. Therefore, this alignment architecture is expected to be applicable to align heterogeneous data pairs in which one modality allows the extraction of semantic labels, semantic vectors, and continuous semantic values. Finally, the time cost of alignment based on the proposed architecture is analyzed in detail in Supplementary Discussion 12.

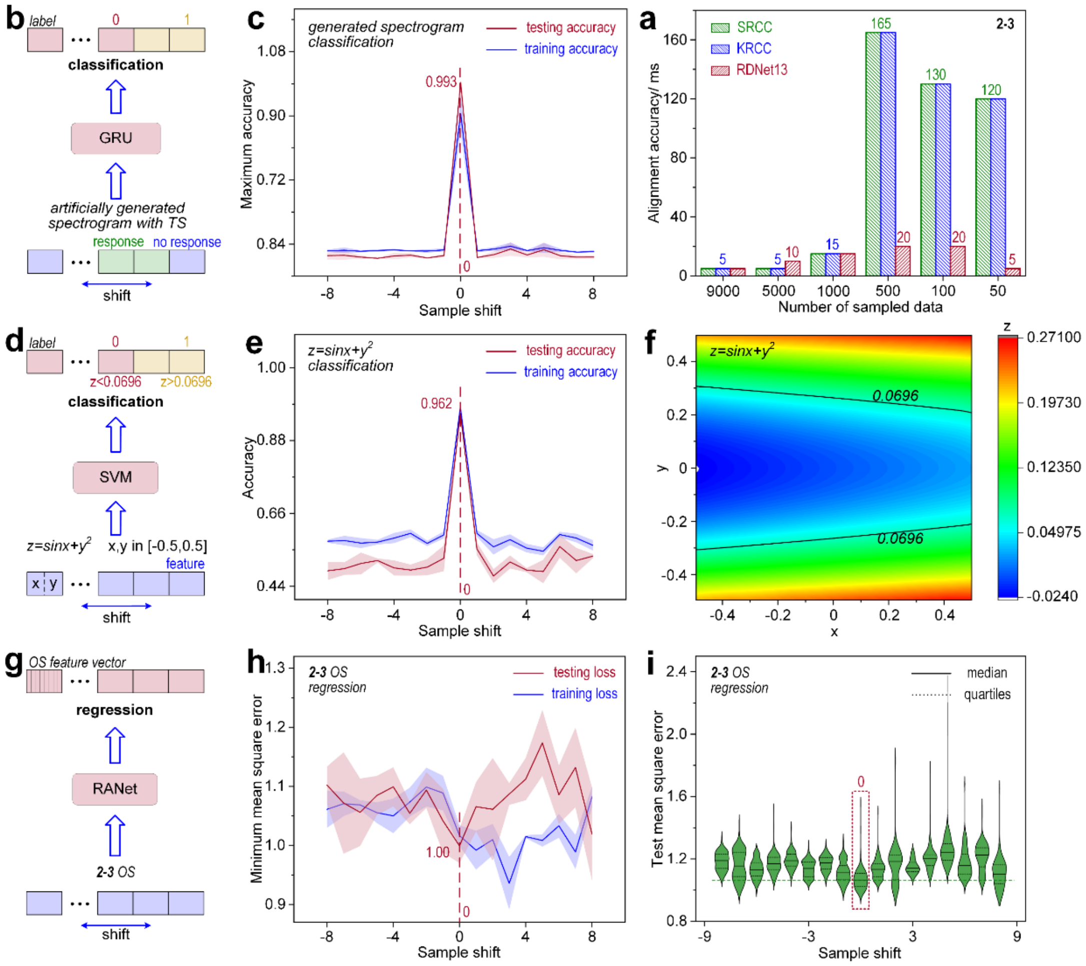

Figure 4. **Analysis of alignment architecture promotion. a** Comparison of alignment accuracy between the RDNet13 kernel model and frequency domain similarity, using the Spearman rank correlation coefficient (SRCC) or the Kendall rank correlation coefficient(KRCC). **b-c** Schematic diagrams and alignment results based on the gate recurrent unit (GRU) kernel model used to align the arc response data from the arc igniter with the corresponding labels. **d-e** Schematic diagrams and alignment results of aligning the input of artificially constructed functions and corresponding labels on the basis of the support vector machine (SVM) kernel model. **f** Cloud diagram of the corresponding constructed functions. **g-h** Schematic diagram and alignment results of aligning optical signals (OS) in dataset **2-3** and feature vectors of the OS via the RANet kernel model. **i** The corresponding distribution of testing accuracy across different sample shifts.

The supplemented contents are in "Supplementary Discussion 11. Supplement of alignment architecture promotion" section are shown as:

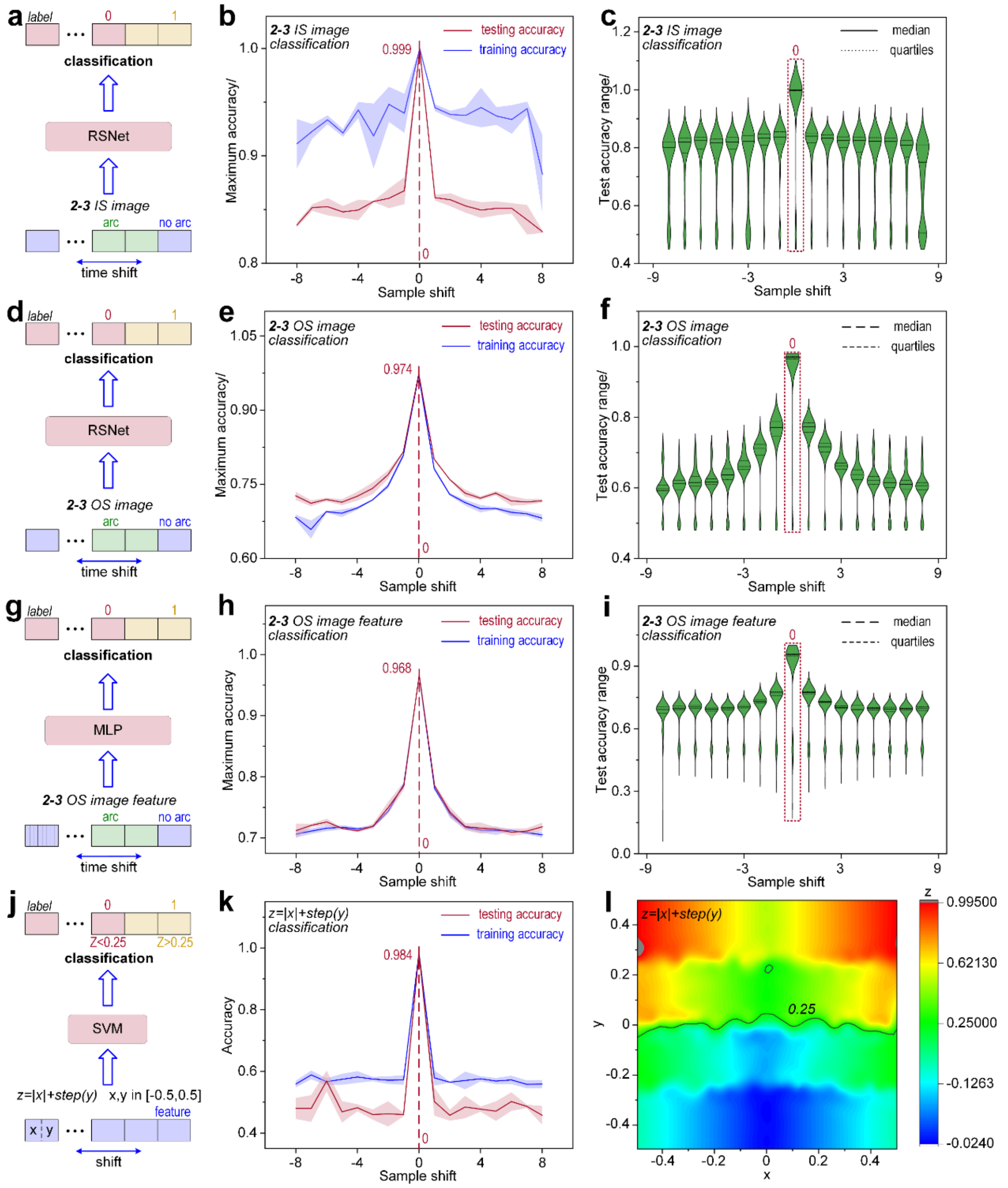

Supplementary Figure 30. **Promotion of the alignment architecture (supplements for Figure 4). a** Schematic diagram of aligning dataset **2-3** infrared signals (IS) with their labels via the RSNet kernel model. **b-c** Sample shift-maximum accuracy curve and testing accuracy distribution corresponding to a, respectively. **d** Schematic diagram of aligning dataset **2-3** optical signals (OS) with their labels via the RSNet kernel model. **e-f** Sample shift-maximum accuracy curves and accuracy distributions corresponding to d, respectively. **g** Schematic diagram of the alignment of dataset **2-3** feature vectors of the OS with their labels via multilayer perceptron (MLP) kernel model. **h-i** The shift-maximum accuracy curve and testing accuracy distribution corresponding to g, respectively. **j-k** Schematic diagram of aligning the input of artificially constructed functions with the corresponding output via the support vector machine (SVM) kernel model and corresponding alignment results. **l** Cloud diagram of the constructed functions in j.

For image data, alignment via the RSNet kernel model was performed between infrared signal images and

corresponding labels in dataset **2-3** (Supplementary Figs. 30a-c), as well as between optical signal (OS) images and their corresponding labels in dataset **2-3** (Supplementary Figs. 30d-f), both of which achieved favorable alignment results. For tabular data, eight statistical features—mean, maximum, minimum, range, variance, skewness, kurtosis, and entropy—were extracted from dataset **2-3** OS images to form 8-dimensional feature vectors. The alignment between these vectors and their corresponding labels was performed via a multilayer perceptron (MLP) kernel model (Supplementary Figs. 30g–i). The model achieved maximum training and testing accuracy at a sample shift of 0, confirming the suitability of the proposed alignment architecture for structured tabular data. Additionally, the input vectors of the function $z = |x| + \mathrm{step}(y)$ and corresponding labels based on the support vector machine (SVM) were successfully aligned (Supplementary Figs. 30j-l). Subsequently, alignments between the input vectors and outputs of the above two functions were performed via a regression-based kernel model (Supplementary Fig. 31). Both models receive a two-dimensional feature vector composed of x and y as inputs and predict the corresponding z values. Both the training and testing mean squared errors reached their minimum at a sample shift of 0 for the two models, demonstrating that the proposed alignment architecture is also applicable to regression tasks.

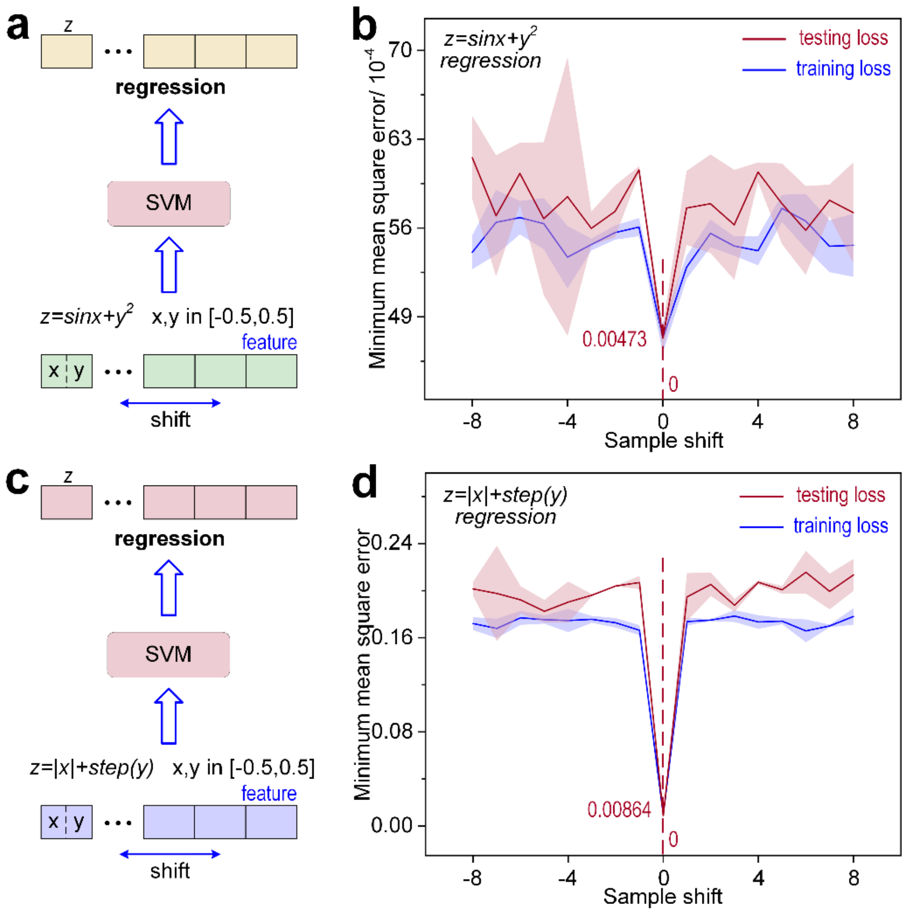

Supplementary Figure 31. **Promotion of the alignment architecture (supplements for Fig. 4). a-b** Schematic diagram of aligning the input of artificially constructed functions ($z = \sin x + y^2$) with the corresponding output via the support vector machine (SVM) kernel model and corresponding alignment results. **c-d** Schematic diagram of aligning the input of artificially constructed functions ($z = |x| + \mathrm{step}(y)$) with the corresponding output via the SVM kernel model and corresponding alignment results.

Comment 2:

The manuscript title is not good reflection of the research work presented in this manuscript. For me, the main contribution of this manusript is the experimental work, and the knowledge contribution on alignment of acoustic signals and optical images is minor.

Answer of comment 2:

Thanks very much for your valuable comment. Based on your comment, we have revised the title to define the applicable scenarios of the proposed alignment architecture. we have also supplement research on the factors affecting alignment accuracy to enrich the research content related to alignment architecture in the “2.3. Factors affecting alignment accuracy” section. Finally, to demonstrate the advantages of the proposed architecture, we supplemented the alignment method based on frequency domain similarity and the comparative study between this method and the alignment architecture in the “2.4. Promotion of alignment architecture” section and the “Supplementary Discussion 10. The alignment method based on frequency domain similarity” section.

The innovation of the paper has been explained in the response to Comment 1. Meanwhile, a cross disciplinary research paper should provide value to a wider range of readers. We support your viewpoint, so we have made public the website corresponding to our dataset in section “Data availability” for specialized readers in the fields of artificial intelligence and multimodality.

The revised contents are in “title” section are shown as:

Machine Learning-driven Alignment Architecture of Heterogeneous Data with Transient Varying Semantics

The supplemented contents are in “2.3. Factors affecting alignment accuracy” section are shown as:

In systems with a constant semantic time shift (TS), synchronous hardware triggering is applied to ensure the simultaneous acquisition of heterogeneous signals. Simultaneously, the TS can be estimated once during an initial calibration using the alignment architecture and subsequently reused, resulting in minimal computational cost. In contrast, in systems with a varying semantic time shift—such as those affected by time-varying delays—the alignment architecture must be further optimized to reduce the computational cost. Fig. 3 illustrates the alignment accuracy between OS and AS in dataset **2-3**, evaluated across different kernel models (KMs), the number of samples (NS), and positive-to-negative sample ratios. Fig. 3a presents the alignment accuracy for various KM sizes, including RRNet18 and RRNet10, with KM sizes ranging from 42.6 MB to 1.26×10^{-4} MB. The smallest model (MNet2_1) consists of only two layers and a single channel. As the KM size decreases, the alignment accuracy fluctuates under time resolutions of 5 ms and 10 ms, with a higher frequency of occurrence at 5 ms (Fig. 3a). Contrary to the intuitive expectation that the standard deviation (STD) of maximum testing accuracy (MTA) across different TSs systematically increases with reduced KM size, no consistent negative correlation between these two variables is observed. Fig. 3b shows the mean of the maximum training accuracy or the MTA across the KMs. Reducing the KM size corresponds to a clear transition from overfitting to underfitting. Despite the emergence of underfitting, the alignment accuracy remains unaffected. The corresponding bubble plot in Fig. 3c illustrates the relationships among the KMs, TS, and MTA. For each model, the MTA initially increases with TS, reaches a peak near the semantic TS, and subsequently decreases. Notably, this peak-like extremum remains pronounced even as the KM size decreases, demonstrating that alignment accuracy remains significantly unaffected by the KM size. The overall training process maintains relative stability despite potential underfitting tendencies in small kernel models, as corroborated by the epoch-accuracy and loss curves in Supplementary Fig. 22.

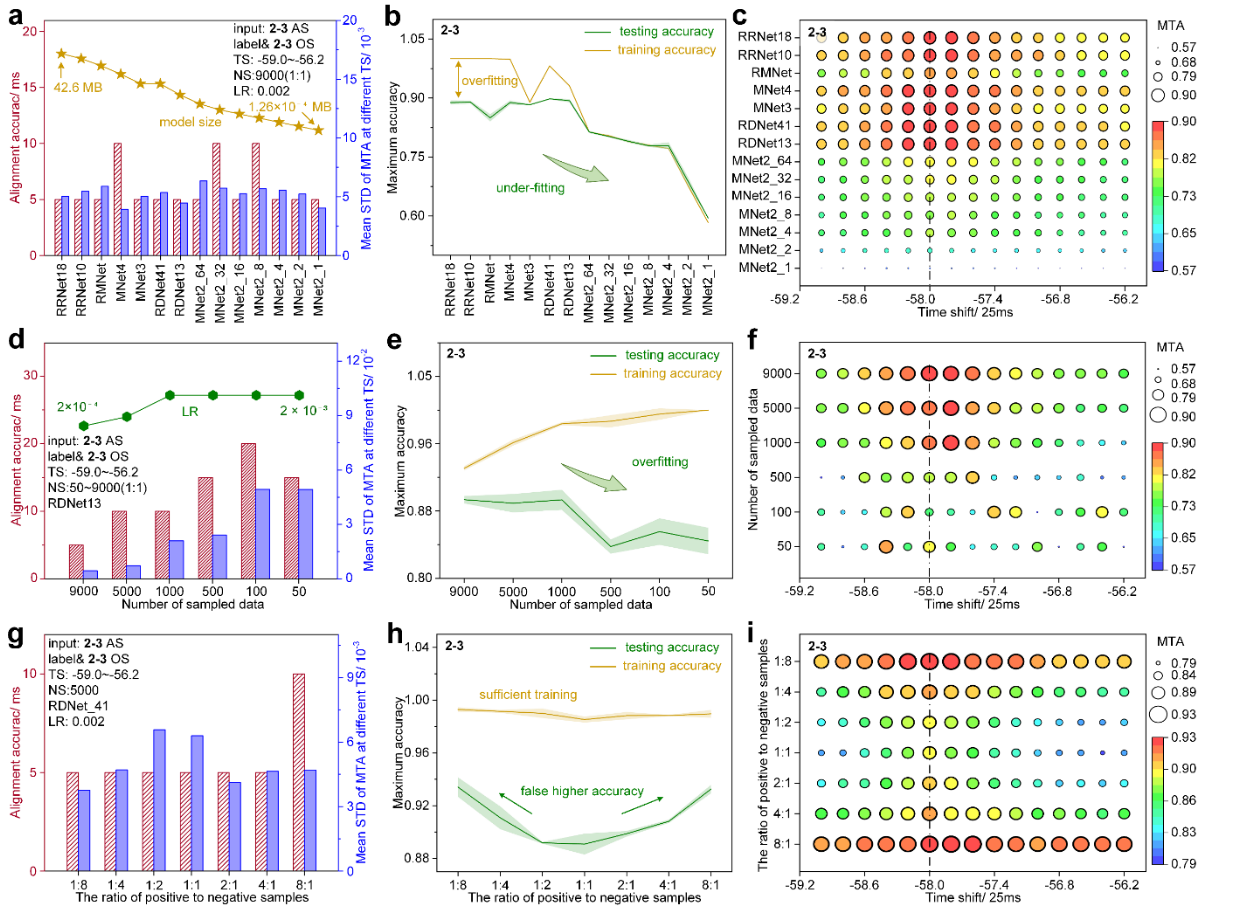

Figure 3. **Analysis of factors affecting alignment accuracy. a** Variance in alignment accuracy and standard deviation (STD) of the maximum testing accuracy (MTA) across different kernel models under the same learning rate (LR). **b** Maximum training and testing accuracy across different kernel models. **c** Bubble chart of the MTA across different kernel models and time shifts (TSs) (step size: 5 ms). **d** Alignment accuracy of the RDNet13 kernel models as the number of samples (NS) varies. **e** Corresponding NS–maximum accuracy curve. **f** Corresponding bubble chart of the MTA across different TSs (step size: 5 ms). and NS. **g** Variation in alignment accuracy with the proportion of positive to negative samples. **h** Maximum accuracy across different training sample proportions. **i** Corresponding bubble chart of MTA across different TSs (step size: 5 ms) and training sample proportions.

Figs. 3d–3f illustrate the impact of the NS on alignment accuracy. When the same kernel model is employed for alignment across varying NSs, the models trained with fewer samples exhibit more pronounced underfitting. This underfitting is reflected in the reduced testing accuracy, which in turn undermines the credibility of the alignment. Therefore, the KM was trained using learning rates that increased from 2×10^{-4} at an NS of 9000 to 2×10^{-3} at an NS of 50. The learning rate is negatively correlated with the NS (Fig. 3d). The alignment accuracy deteriorates from 5 ms at an NS of 9000 to 20 ms at an NS of 50. Concurrently, the average STD of the MTA across TSs increases as the NS decreases. With decreasing NS, the kernel model in the semantic TS exhibits increased overfitting and diminished generalization capability, resulting in decreased credibility of the alignment result, as shown in Fig. 3e. As shown in Fig. 3f, as the NS decreases, the trend of peak-shaped variation in the MTA near the semantic TS becomes less pronounced. In other words, the correlation—whether positive or negative—between the MTA on either side of the semantic TS and the TS weakens as the NS decreases. The typical epoch-loss and accuracy curves of the kernel model corresponding to Fig. 3f are shown

in Supplementary Fig. 23. The KM exhibits an unstable training process when the NS falls below 500. Under stable training conditions, a higher STD in the MTA across TSs reflects a greater sensitivity of accuracy to temporal variation, which is advantageous for alignment. However, in the case of unstable training, an increased STD (Fig. 2d) does not contribute to improved alignment, because the variability primarily arises from training instability rather than from meaningful temporal sensitivity. Additionally, when aligning the OS with the IS via the RDNet13 kernel and a limited NS of 50, an alignment accuracy of 15 ms was achieved, as shown in Fig. 3d. As illustrated in Fig. 1f, the arc events extracted from both the IS and OS demonstrate a temporally consistent distribution. The failed alignment between the IS and AS, as shown in Fig. 2a, is likely due to inaccuracies in the local time axis of the IS captured by the thermal imager, rather than the limited number of IS images (1800) associated with the arc. The effect of NS on alignment accuracy based on the extremely lightweight kernel model Mnet2_1 is similar to that of RDNet13, as presented in Supplementary Discussion 9. Moreover, the maximum degradation in alignment resolution within the range of 50-9000 samples is limited to just 15 ms. Therefore, in the initial stages of the large-scale search for alignment parameters, adopting a coarse alignment strategy based on a small NS and lightweight KMs is a cost-effective and practical solution.

Figs. 3g–i illustrate the effect of the positive-to-negative sample ratio on alignment accuracy. Fig. 3g presents the alignment accuracy and the mean STD of MTA across TSs for sample ratios ranging from 1:8 to 8:1. Stratified sampling is applied to divide the training and testing sets. The alignment accuracy is 5 ms for most sample ratios; however, an increased imbalance in the sample ratio leads to a slight degradation in alignment performance. As the sample imbalance increases, the mean STD of MTA across TSs decreases, corresponding to a reduction in alignment accuracy. Fig. 3h presents the relationships between the sample ratio and the maximum training accuracy and MTA. With increasing sample imbalance, the fluctuation in maximum training accuracy remains small, which is attributable to the sufficient expressive power of the KM. As the degree of sample imbalance increases, the MTA also increases. This phenomenon arises because the loss function penalizes samples from the majority class more heavily than those from the minority class do, resulting in higher predictive accuracy for overrepresented samples than for minority-class samples. Consequently, when the majority class dominates the testing set, the overall testing accuracy may appear artificially inflated, failing to reflect actual model performance and compromising the evaluation of alignment accuracy. It is therefore advisable to maintain a positive-to-negative sample ratio as close to 1:1 as practicable. In scenarios with a limited NS, transfer learning algorithms may be employed to compensate for data scarcity. As shown in Fig. 3i, owing to the relatively stable training process of the KM algorithm, the trend of mountain-shaped variation in accuracy remains well-preserved even under increasingly imbalanced sample ratios. However, as the sample imbalance intensifies, the difference in the MTA at different TSs diminishes, which is consistent with the trend in the STD of MTA observed in Fig. 3g. The quantitative strength of the mountain-peak trend can be characterized by a weighted mean of the rank correlation coefficients between the TSs and MTA on both sides of the maximum value of MTA. This metric serves as a direct indicator, where a larger value corresponds to a more distinct peak-shaped feature.

For scenarios that demand real-time alignment and involve a broad search range of semantic TSs, a two-level alignment strategy can be employed. At the first level, alignment is performed via a lightweight KM trained on a small NS, with coarse TS step size to search for alignment parameters and reduce the computational cost. Given the potentially limited expressive capacity of the KM relative to the data complexity, hyperparameter optimization is necessary to maximize the testing accuracy and increase the prominence of the peak-shaped variation trend of the MTA across different TSs. At the second-level alignment, a narrower search range is defined around the semantic TS identified during the first-level coarse alignment, thereby reducing

the computational cost of fine-level alignment. The fine-level alignment is performed via a higher-complexity KM trained on a larger NS with a finer time shift step to search for alignment parameters. The expressive capacity of the KM should be sufficient relative to the sample complexity, and its hyperparameters should be optimized to ensure training stability and to maximize testing accuracy. Additionally, in fine alignment, the samples used in coarse alignment can be leveraged to reduce the number of preprocessing groups required in fine alignment. Meanwhile, the sampling step can be performed before the data preprocessing step, and preprocessing only the sampled samples can also reduce the time consumption of the alignment framework.

The supplemented contents are in "2.4. Promotion of alignment architecture" section are shown as:

Given the limited research on algorithms closely resembling the architecture proposed in this study, an alternative alignment method based on frequency domain similarity (Supplementary Discussion 10) was developed for comparative analysis. This method was evaluated further to illustrate the relative advantages of the proposed architecture. On the basis of the same alignment parameters, a comparison was conducted between the alignment results produced by the frequency domain similarity–based method and those obtained via the neural network–based architecture (Figs. 3d–f), as summarized in Fig 4a. Although the neural network–based architecture achieves slightly lower alignment accuracy (10 ms) than the frequency domain similarity–based method under the number of samples of 5,000, the architecture remains robust under data-limited conditions. In contrast, the accuracy of the frequency domain similarity–based method substantially decreases (120–165 ms) when the number of samples is reduced to 500 or less, whereas the neural network–based architecture maintains superior precision (5–20 ms). The results indicate that, although frequency domain representations of arc responses in the AS modality exhibit measurable similarity, such patterns are not prominent in small-sample analyses and require large datasets for reliable detection. The computational costs of the respective alignment methods are reported in Supplementary Tables 17 and 18. The evolution of numerical modeling has undergone three major paradigm shifts. The first stage is defined by numerical fitting methods, such as least squares, which rely on predefined basis functions. The second stage introduces traditional machine learning algorithms, including support vector machines (SVMs), which eliminate the need for explicit basis functions but operate within limited solution spaces. The third and current stage is characterized by deep learning algorithms, which are initiated by models from the AlexNet era and are capable of learning complex patterns directly from data. Compared with earlier modeling paradigms, the training of deep learning models offers significantly expanded paths for identifying optimal solutions. Using AlexNet as a representative example, the process begins with weight initialization, followed by progressive feature transformation stages that enrich representation through dimensional expansion and spatial reduction of feature maps. Hierarchical connectivity across layers further enhances the expressive capacity of the model, enabling the learning of increasingly abstract features. During training, loss functions guide parameter updates by reinforcing features that improve task performance, thereby systematically directing the model toward accurate classification or regression outcomes. The neural network–based alignment architecture reflects the characteristics of the third modeling paradigm, which involves solving for the target kernel model (KM) through multiple adaptive strategies involving feature aggregation and enrichment, thereby achieving high-quality alignment. In contrast, the frequency domain similarity–based algorithm resembles the paradigms of the first and second stages, employing relatively fixed rules that rely solely on frequency-domain vector similarities. This fundamental limitation explains its significantly lower accuracy than neural network–based alignment when working with a limited number of samples. However, algorithms that rely on relatively fixed approaches empowered by relevant domain knowledge to find solution functions are still recommended because they consume relatively low algorithmic costs. However, in the absence of such prior knowledge,

neural networks offer clear advantages. Deep learning models with strong expressive capacity can flexibly adapt to end-to-end learning across diverse data types, including tabular data, images, semantic vectors, and time series. The primary trade-offs of neural network–based approaches include increased demand for data and computational resources. Nonetheless, when domain knowledge is unavailable, the cost associated with acquiring large-scale data and supporting sufficient computational power becomes a necessary investment.

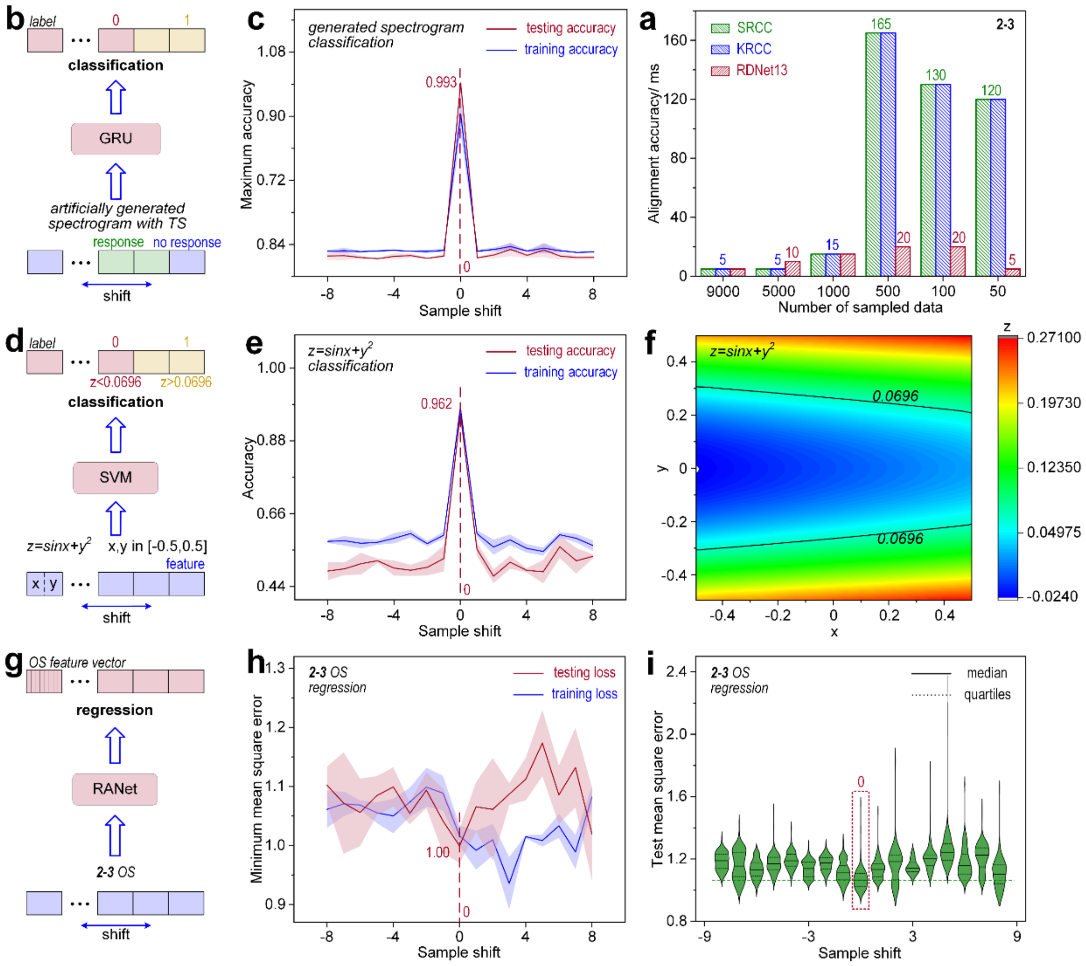

Figure 4. **Analysis of alignment architecture promotion. a** Comparison of alignment accuracy between the RDNet13 kernel model and frequency domain similarity, using the Spearman rank correlation coefficient (SRCC) or the Kendall rank correlation coefficient(KRCC). **b-c** Schematic diagrams and alignment results based on the gate recurrent unit (GRU) kernel model used to align the arc response data from the arc igniter with the corresponding labels. **d-e** Schematic diagrams and alignment results of aligning the input of artificially constructed functions and corresponding labels on the basis of the support vector machine (SVM) kernel model. **f** Cloud diagram of the corresponding constructed functions. **g-h** Schematic diagram and alignment results of aligning optical signals (OS) in dataset **2-3** and feature vectors of the OS via the RANet kernel model. **i** The corresponding distribution of testing accuracy across different sample shifts.

The supplemented contents are in "Data availability" section are shown as:

The authors declare that the main data supporting the findings of this study are available within the article and its Supplementary Information files. Source Data are provided with this paper. Extra data are available from the corresponding author upon request. The datasets used for data alignment, as well as training and testing of the arc detection models have been deposited in the public repository (https://www.scidb.cn/en/s/iMnaii).

The supplemented contents are in "Supplementary Discussion 10. The alignment method based on frequency domain similarity" section are shown as:

The implementation and results of the frequency domain similarity-based alignment method are shown in Supplementary Figs. 26a–c. To address the challenge of defining explicit correlation functions between heterogeneous data modalities, an approach leveraging intramodal similarity was proposed under the assumption that spectrograms corresponding to the same label category—such as arc response and no arc response—exhibit inherent similarity. As shown in Supplementary Fig. 26a, for the optical signal (OS) modality, similarity is quantified by computing the coincidence accuracy between the OS label sequence and the offset OS label sequence. For the acoustic signal (AS) modality, the time-domain signal of each segment is first transformed into the frequency domain. To ensure comparability, the amplitude of the frequency-domain signal is scaled such that the sum of the amplitudes remains constant. Inspired by the geometric principle that the square in a rectangle with equal perimeter yields the maximum area, the similarity between two signals is quantified via the dot product between the scaled frequency-domain vectors, where an increasing dot product denotes greater similarity between two signals. For the AS segment sequence, the original AS sequence corresponds to the time shift, whereas each offset AS sequence corresponds to the superposition of the time shift and offset. The offset-label coincidence accuracy curve, derived from the OS sequence, and the offset-frequency domain similarity curve, obtained from the AS sequence across different time shifts, are shown in Supplementary Fig. 26b. Suppose that a given time shift accurately reflects accurate semantic alignment. In that case, the positive correlation between the label coincidence accuracy and frequency domain similarity at that time shift should be stronger than that observed at other time shifts. The mathematical formulation of this algorithm is shown in Supplementary Equation (1):

$$\left\{\begin{array}{l} S_y[t_{\mathrm{of}}] = \sum_{n=1}^{N} M(y_1[n,0], y_1[n,t_{\mathrm{of}}])/N \\ S_{\mathrm{fft}}[t_{of},t] = \sum_{n=1}^{N} k \cdot \mathbf{F}_{2,\mathrm{norm}}^{\mathrm{fft}}[n,t] \bullet \mathbf{F}_{2,\mathrm{norm}}^{\mathrm{fft}}[n,t_{\mathrm{of}}+t] \\ c_{\mathrm{Sr}}[t] = 1 - \frac{6\cdot\sum_{t_{\mathrm{of}}=-T_{\mathrm{of}}}^{T_{\mathrm{of}}} d_n^2(S_y[t_{\mathrm{of}}], S_{\mathrm{fft}}[t_{\mathrm{of}},t])}{N^3-N} \\ c_{\mathrm{Kr}}[t] = \frac{C(S_y[t_{\mathrm{of}}],S_{\mathrm{fft}}[t_{\mathrm{of}},t]) - D(S_y[t_{\mathrm{of}}],S_{\mathrm{fft}}[t_{\mathrm{of}},t]}{\frac{1}{2}\cdot N\cdot(N-1)} \\ t_{\mathrm{Sr}} = \max_t c_{\mathrm{Sr}}[t] \\ t_{\mathrm{Kr}} = \max_t c_{\mathrm{Kr}}[t] \\ T_{\mathrm{Sr}} = t_{\mathrm{Sr}} \cdot T_p \\ T_{\mathrm{Kr}} = t_{\mathrm{Kr}} \cdot T_p \end{array}\right\} t_{\mathrm{of}} \in Z, y_1[n,t] \in \{0,1\} \quad (1)$$

where $y_1[n,0]$, $S_y[t_{\mathrm{of}}]$, $\mathbf{F}_{2,\mathrm{norm}}^{\mathrm{fft}}[n,t]$, and $S_{\mathrm{fft}}[t_{of},t]$ denote the label obtained from signal $x_1[n,0]$, the label coincidence accuracy between sequences $\{y_1[0,0], y_1[1,0], \cdots, y_1[N-1,0]\}$ and sequences $\{y_1[0,t_{\mathrm{of}}], y_1[1,t_{\mathrm{of}}], \cdots, y_1[N-1,t_{\mathrm{of}}]\}$, the scaled frequency-domain vectors of signal $x_2[n,t]$ (where the sum of the elements of each vector is equal), and the frequency-domain similarity between sequences $\{\mathbf{F}_{2,\mathrm{norm}}^{\mathrm{fft}}(0,t)$, $\mathbf{F}_{2,\mathrm{norm}}^{\mathrm{fft}}(1,t)$, $\cdots$, $\mathbf{F}_{2,\mathrm{norm}}^{\mathrm{fft}}(N-1,t)\}$ and $\{\mathbf{F}_{2,\mathrm{norm}}^{\mathrm{fft}}(0,t+t_{\mathrm{of}})$, $\mathbf{F}_{2,\mathrm{norm}}^{\mathrm{fft}}(1,t+t_{\mathrm{of}})$, $\cdots$, $\mathbf{F}_{2,\mathrm{norm}}^{\mathrm{fft}}(N-1,t+t_{\mathrm{of}})\}$, respectively. $c_{\mathrm{Sr}}[t]$ and $c_{\mathrm{Kr}}[t]$ denote the Spearman rank coefficient and the Kendall rank coefficient, respectively. d^n denotes the rank difference between the matching degree sequences $\{S_y[-T_{\mathrm{of}}]$, $S_y[-T_{\mathrm{of}}+1]$, $\cdots$, $S_y[T_{\mathrm{of}}]\}$ and the similarity sequence $\{S_{\mathrm{fft}}[-T_{\mathrm{of}},t]$, $S_{\mathrm{fft}}[-T_{\mathrm{of}}+1,t]$, $\ldots$, $S_{\mathrm{fft}}[T_{\mathrm{of}},t]\}$. $C(S_y[t_{\mathrm{of}}], S_{\mathrm{fft}}[t_{\mathrm{of}},t])$ and $D(S_y[t_{\mathrm{of}}], S_{\mathrm{fft}}[t_{\mathrm{of}},t])$ denote the logarithms of the consistent and inconsistent elements between sequences $\{S_y[-T_{\mathrm{of}}]$, $S_y[-T_{\mathrm{of}}+1]$, $\cdots$, $S_y[T_{\mathrm{of}}]\}$ and $\{S_{\mathrm{fft}}[-T_{\mathrm{of}},t]$, $S_{\mathrm{fft}}[-T_{\mathrm{of}}+1,t]$, $\ldots$, $S_{\mathrm{fft}}[T_{\mathrm{of}},t]\}$, respectively. Here, T_{of} is set to 8. t_{Sr} and t_{Kr} denote the

semantic alignment parameters between the semantic sequences of signals $x_1[n,0]$ and $x_2[n,0]$ calculated on the basis of the Spearman rank correlation coefficient and the Kendall rank correlation coefficient, respectively. T_{Sr} and T_{Kr} are the corresponding semantic time shifts, and the search range of the semantic time shift is $\mathrm{t}\cdot T_p$. The pseudocode corresponding to the above algorithm is shown in Algorithm 2.

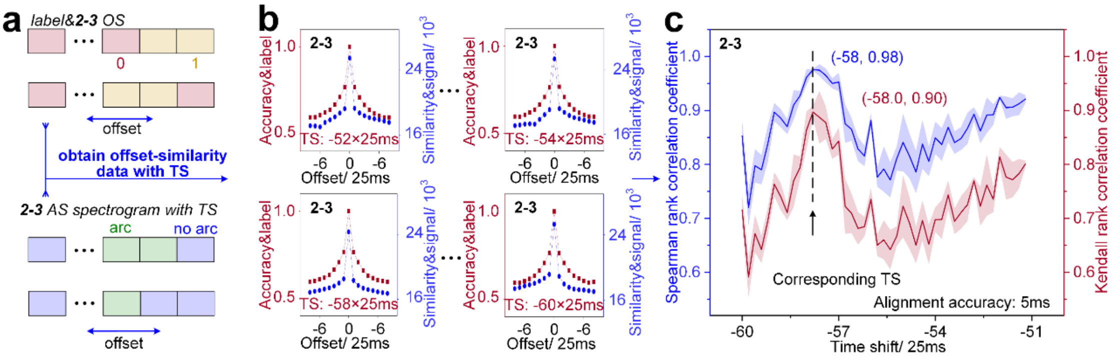

Supplementary Figure 26. **The alignment method on the basis of frequency domain similarity. a** Implement schematic of the alignment method. **b** Offset-accuracy and similarity curves under various time shifts (TSs). **c** Alignment results based on the method between optical signals (OS) and acoustic signals (AS).

Supplementary Algorithm 2. **The frequency domain similarity-driven data alignment method**

Input: the list of normalized frequency domain signal of AS and label list.

1 initialization, including the search ranges of time shift parameter t, ranges of offset parameter t_{of} and number of samples N, etc.

2 **for** t in range list:

3 randomly extract equal proportional labels of "0" and "1" from the label list to form a list $\{y_1[0,0],\ y_1[1,0],\ \cdots,\ y_1[N-1,0]\}$.

4 extract the corresponding list of scaled frequency domain signal of AS $\{\mathbf{F}_{2,\mathrm{norm}}^{\mathrm{fft}}(0,t),\ \mathbf{F}_{2,\mathrm{norm}}^{\mathrm{fft}}(1,t),\ \cdots,\ \mathbf{F}_{2,\mathrm{norm}}^{\mathrm{fft}}(N-1,t)\}$.

5 **for** t_{of} in list $\{-T_{\mathrm{of}},\ -T_{\mathrm{of}}+1,\ \cdots,\ T_{\mathrm{of}}\}$:

6 calculate the coincidence accuracy $S_y[t_{\mathrm{of}}]$ between label list $\{y_1[0,0],\ y_1[1,0],\ \cdots,\ y_1[N-1,0]\}$ and offset label list $\{y_1[0,t_{\mathrm{of}}],\ y_1[1,t_{\mathrm{of}}],\ \cdots,\ y_1[N-1,t_{\mathrm{of}}]\}$ according to Supplementary Equation (1).

7 **for** t_{of} in list $\{-T_{\mathrm{of}},\ -T_{\mathrm{of}}+1,\ \cdots,\ T_{\mathrm{of}}\}$:

8 calculate the degree of similarity $S_{\mathrm{fft}}[t_{\mathrm{of}},t]$ between frequency domain signal list $\{\mathbf{F}_{2,\mathrm{norm}}^{\mathrm{fft}}(0,t),\ \mathbf{F}_{2,\mathrm{norm}}^{\mathrm{fft}}(1,t),\ \cdots,\ \mathbf{F}_{2,\mathrm{norm}}^{\mathrm{fft}}(N-1,t)\}$ and offset frequency domain signal list $\{\mathbf{F}_{2,\mathrm{norm}}^{\mathrm{fft}}(0,t+t_{of}),\ \mathbf{F}_{2,\mathrm{norm}}^{\mathrm{fft}}(1,t+t_{of}),\ \cdots,\ \mathbf{F}_{2,\mathrm{norm}}^{\mathrm{fft}}(N-1,t+t_{of})\}$ according to Supplementary Equation (1).

9 calculate the Spearman rank coefficient $c_{\mathrm{Sr}}[t]$ and Kendall rank coefficient $c_{\mathrm{Kr}}[t]$ for list $\{S_y[-T_{\mathrm{of}}],\ S_y[-T_{\mathrm{of}}+1],\ \cdots,\ S_y[T_{\mathrm{of}}]\}$ and $\{S_{\mathrm{fft}}[-T_{\mathrm{of}},t],\ S_{\mathrm{fft}}[-T_{\mathrm{of}}+1,t],\ \ldots,\ S_{\mathrm{fft}}[T_{\mathrm{of}},t]\}$ based on Supplementary Equation (1).

11 calculate the semantic time shift T_{Sr} and T_{Kr} between signal $x_1[n,0]$ and $x_2[n,0]$ based on Supplementary Equation (1).

The sampling methods and ratios employed in Supplementary Figs. 26b-c are consistent with those

utilized in Figs. 3g-i, with 9,000 samples analyzed for the former. The time shift–level correlation coefficient curve generated by the proposed algorithm is presented in Supplementary Fig. 26c. Both the Spearman and the Kendall rank correlation coefficients reach their extrema at a time shift of -58.0×25 ms, indicating that the semantic time shift t_o^a is -58.0 × 25 ms with an alignment accuracy of 5 ms. This estimated semantic time shift lies within the expected reference range and is consistent with the outcome derived from the proposed neural network-based alignment architecture. Furthermore, large-scale data analysis confirms that spectrograms exhibit substantial similarity within the arc response category, whereas those that display clear distinctions between arc and no-arc responses.

Comment 3:

The writing of the manuscript needs to be improved significantly. A more structural way will help audiences to capture the key information.

Answer of comment 3:

Thanks very much for your valuable comment. Based on your comment and official requirements of the journal, we have thoroughly reorganized the paper. The "2. Results and discussion" section the paper mainly consists of four parts. The first part introduces the alignment architecture, the second part discusses the application of alignment architecture, the third part introduces the factors that affect alignment accuracy, and the fourth part introduces the promotion of alignment architecture. And we have placed the alignment architecture related content in the main position of the paper, and moved other content related to the topic to the supplementary materials to improve the clarity of the paper. And we have placed the alignment architecture related content in the main position of the paper, and moved the device related content to the supplementary materials to improve the clarity of the paper. And we have supplemented a "methods" section to enhance the reproducibility of the paper. The specific modifications are shown in the revised manuscript and supplementary information.

Again, we are so sorry to bring you so much trouble. All the detailed changes are marked in the revised manuscript. At last, thank you for your review and your comments again.

Best regards.

On behalf of all co-authors.

Response Letter

Dear editors and reviewers,

We declare that the **submitted manuscript is the revised and resubmitted version of NCOMMS-25-26757A.** Thank you very much for the opportunity to revise our manuscript and for the thoughtful comments provided. We are grateful that, based on the positive assessments from Reviewers 1, 3, 4, 5, and 6 in the previous round, the editor offered an "accept in principle" decision. The constructive suggestions from all reviewers have been invaluable in improving our work.

In this revision, we have carefully addressed the remaining concerns. Specifically, the points raised by Reviewer 2 regarding the nature and contribution of our method have been clarified in detail below. Following suggestions of Reviewer 5, we have also updated our public code repository by supplementing a requirements.txt file and improving the environment setup and script execution instructions to enhance reproducibility. Reviewers 1, 3, 4, and 6 had no further comments, and we thank them for their positive feedback and valuable input in the previous round, which helped us strengthen the manuscript. Also, we have also revised and supplemented the relevant documents according to the requirements of “Author_Checklist”. Additionally, we have thoroughly reviewed the entire manuscript and corrected some errors to further improve the clarity and rigorism.

We believe the revisions have addressed all remaining comments and hope the manuscript is now suitable for publication in Nature Communications. A point-by-point response to each reviewer's comments is provided below.

Reviewer #2

Comment:

I appreciate the author’s careful and detailed responses. The authors’ replies to Comments 1 and 2 are clear and sufficiently address my concerns.

However, I do not agree with the authors’ response to Comment 3. In my view, the proposed method is essentially a “brute-force alignment” approach, one of the most fundamental and inefficient approaches in sequence alignment. The authors’ method treats the time shift as an unknown variable and searches for the optimal shift using a neural network as a predictor. In addition, dividing the measurement data into training and test sets to evaluate performance is a similar concept to cross-validation. While the experimental results may demonstrate a certain level of practical usefulness for the specific measurement scenarios considered, I believe that the contribution is incremental. Therefore, this work should be published in an application-focused journal rather than Nature Communications.

Answer of comment:

We appreciate the reviewer's continued attention and recognition of the clarity of our responses to comments 1 and 2. In response to the concerns raised incomment 3 regarding the nature of the method, contribution size, and journal matching, we provide the following clarification based on the content of the paper:

(1) Regarding the difference between ‘brute-force alignment’:

The reviewer classified our method as a 'brute force search' approach. We would like to elaborate on the essence of this method from a more macro perspective.

Firstly, the alignment architecture we proposed is essentially a continuation and evolution of the idea of cross-correlation algorithms. When domain knowledge is unknown and the positive and negative correlations between signal features are unclear, defining an appropriate transformation function becomes extremely difficult, as shown in Equation 2 in the main text. The architecture we propose utilizes the feature of neural

networks being able to self-sense the matching degree between input and output data, thereby achieving alignment without prior knowledge. Therefore, rather than calling our method a completely new 'brute force search', it is more accurate to say that it is a natural evolution of cross-correlation algorithms in the era of machine learning.

Secondly, in practical applications, we do not need to traverse all data. As shown in Figures 3d-3f, our algorithm can maintain effective alignment accuracy (15-20 ms) even with extremely small sample sizes (as low as 50 samples). Figure 4a illustrates this advantage more intuitively: when the sample size is reduced to below 500, the accuracy of traditional frequency domain similarity methods drops sharply to 120-165 ms, while our neural network architecture can still maintain high accuracy of 5-20 ms. This indicates that our method is not "inefficient", but an efficient solution designed specifically for practical scenarios where data is scarce and prior knowledge is lacking.

Thirdly, to address the issue of computational overhead, we systematically proposed a two-level alignment strategy in Section 2.3 (Factors affecting alignment accuracy) of the paper to reduce computational costs. This strategy includes:

First level coarse alignment: Use lightweight kernel models (such as MNet1_1, only 1.26×10^{-4} MB) and small samples (as few as 50 samples) for large-scale search to quickly identify candidate alignment regions

Second level fine alignment: using complex models and high sample sizes for fine alignment within a narrowed search range

(2) Regarding core contributions and innovation:

Based on the above understanding, we believe that the core contribution of this work lies in combining the ideas of classical cross-correlation algorithms with the expressive power of machine learning, and proposing a universal alignment architecture that can still work effectively in situations where domain knowledge is unknown and ground truth values are missing. The generality of this framework is reflected at multiple levels (as shown in Figure 4 in Section 2.4):

Diversity of kernel models: supports multiple kernel model such as GRU, SVM, RANet, etc.

Universality of data types: suitable for aligning table data, time series, and image data with semantic vectors and labels.

Comprehensive task types: suitable for classification and regression tasks.

The core phenomenon we discovered - that neural networks can self sense the matching degree between input and output data - provides new ideas for subsequent research. This is not only an application for specific measurement scenarios, but also an extension and deepening of the idea of cross-correlation algorithms.

(3) Regarding whether it is suitable for publication in Nature Communications:

We respectfully believe that this work is highly relevant to the scope of Nature Communications journal, as demonstrated in Section 2.4 and Figure 4 of the paper, which demonstrate its generality. This architecture can serve as the ultimate strategy for aligning data along the timeline and a prerequisite steps for effective fusions of multimodal information (its applicability can also be extended to related interdisciplinary fields of artificial intelligence), especially in scenarios where domain knowledge is scarce (Figure 4a) and traditional methods are difficult to apply. Our work provides a successful example of combining modern artificial intelligence methods with classical principles in various fields and breaking through the performance limits of traditional methods by demonstrating how classical cross-correlation ideas can be used to overcome the inherent limitations of traditional methods through neural networks, such as the need for manual design of transformation functions and poor performance in small samples. We believe that the enlightening value of this paradigm level is exactly what Nature Communications hopes to convey to the vast scientific research community.

I hope the above clarification can address the concerns of the reviewers.

Reviewer #4

Comment:

The authors have clearly addressed all my concerns, I have no further comments.

Answer of comment:

We thank the reviewer for their positive feedback and valuable suggestions, which have helped improve our manuscript.

Reviewer #5

Comment:

The repository provides the essential code components needed to reproduce the paper's main results, including scripts for data alignment, arc detection modeling, class activation mapping, and data preprocessing. A README file is included with hardware and software requirements clearly listed (Python 3.9.6, PyTorch 2.2.2, etc.), and the authors have linked the corresponding dataset on an external repository. The code is organized into logical folders, and a demo case is provided that allows users to quickly verify the core alignment methodology.

The installation instructions are somewhat minimal—there's no requirements.txt file provided, and the guidance essentially tells users to "use pip" without specific commands. The reliance on Sublime Text for running scripts is a bit unusual compared to standard command-line approaches. The authors note that more detailed documentation will be added after publication, which suggests the current state is preliminary.

Answer of comment:

We thank the reviewer for the positive assessment of our code and the valuable guidance. Following the reviewer's suggestion, we have now supplemented a requirements.txt file to the repository, which lists all necessary Python packages along with their recommended versions. The installation instructions in the README have been thoroughly updated with clear commands for environment setup and code execution. We have also clarified that while editors like PyCharm or Sublime Text can be used to view or modify the code, execution is independent of the editor and follows standard command-line practices. Additionally, we have included contact information for the code developer in the README to facilitate communication and welcome further suggestions. After publication, we will also add the link to the paper in the README to ensure easy access for readers. We believe these revisions have addressed the reviewer's concerns and improved the overall usability of the code. The relevant modifications are shown in our public storage repository (https://github.com/ChaofanLi-JinlinUniversity/Alignment-Architecture-of-Heterogeneous-Data).

Reviewer #6

Comment:

The issues raised in my previous review have been addressed.

Answer of comment:

We thank the reviewer for their positive feedback and valuable suggestions, which have helped improve our manuscript.

At last, thank you for your review and your comments again.

Best regards.

On behalf of all co-authors.
